# Hydroxychloroquine in patients with mainly mild to moderate coronavirus disease 2019: open label, randomised controlled trial

**DOI:** 10.1136/bmj.m1849

**Published:** 2020-05-14

**Authors:** Wei Tang, Zhujun Cao, Mingfeng Han, Zhengyan Wang, Junwen Chen, Wenjin Sun, Yaojie Wu, Wei Xiao, Shengyong Liu, Erzhen Chen, Wei Chen, Xiongbiao Wang, Jiuyong Yang, Jun Lin, Qingxia Zhao, Youqin Yan, Zhibin Xie, Dan Li, Yaofeng Yang, Leshan Liu, Jieming Qu, Guang Ning, Guochao Shi, Qing Xie

**Affiliations:** 1 Department of Pulmonary and Critical Care Medicine, Ruijin Hospital, Shanghai Jiao Tong University School of Medicine, Shanghai, China; 2 Institute of Respiratory Diseases, School of Medicine, Shanghai Jiao Tong University, Shanghai, China; 3 Department of Infectious Disease, Ruijin Hospital, Shanghai Jiao Tong University School of Medicine, Shanghai 200025, China; 4 Department of Respiratory Medicine, No 2 People’s Hospital of Fuyang City, Fuyang, Anhui, China; 5 Department of Respiratory Medicine, Suizhou Hospital, Hubei University of Medicine, Suizhou, Hubei, China; 6 Department of Respiratory and Critical Care Medicine, Xiangyang No 1 People’s Hospital, Hubei University of Medicine, Xiangyang, Hubei, China; 7 Department of Infectious Diseases, Central Hospital of Ezhou, Ezhou, Hubei, China; 8 Department of Cardiovascular Medicine, Yunmeng People’s Hospital, Xiaogan, Hubei, China; 9 Department of Respiratory Medicine, First People’s Hospital of Jingzhou City, Jingzhou, Hubei, China; 10 Department of Infectious Diseases, Xiaogan Hospital Affiliated to Wuhan University of Science and Technology, Xiaogan, Hubei, China; 11 Department of Emergency Medicine, Ruijin Hospital, Shanghai Jiao Tong University School of Medicine, Shanghai, China; 12 Department of Respiratory Medicine, Putuo Hospital, Shanghai University of Traditional Chinese Medicine, Shanghai, China; 13 Department of Respiratory Medicine, Hubei Space Hospital of Xiaogan, Xiaogan, Hubei, China; 14 Department of Gastroenterology, Zhongnan Hospital of Wuhan University, Wuhan, Hubei, China; 15 Department of Infectious Disease, The Sixth People’s Hospital of Zhengzhou, Zhengzhou, Henan, China; 16 Department of Infectious Disease, Wuhan No 7 Hospital, Wuhan, Hubei, China; 17 Departments of Respiratory Medicine, Xiaogan Hospital Affiliated to Wuhan University of Science and Technology, Xiaogan, Hubei, China; 18 Department of Respiratory Medicine, The Third People’s Hospital of Yichang, Yichang, Hubei, China; 19 Department of Respiratory Medicine, Xiao Gan First People’s Hospital, Xiaogan, Hubei Province, China; 20 Clinical Research Centre, Ruijin Hospital, Shanghai Jiao Tong University School of Medicine, Shanghai, China; 21 Shanghai National Research Centre for Endocrine and Metabolic Diseases, State Key Laboratory of Medical Genomics, Shanghai Institute for Endocrine and Metabolic Diseases, Ruijin Hospital, Shanghai Jiao Tong University School of Medicine, Shanghai, China

## Abstract

**Objective:**

To assess the efficacy and safety of hydroxychloroquine plus standard of care compared with standard of care alone in adults with coronavirus disease 2019 (covid-19).

**Design:**

Multicentre, open label, randomised controlled trial.

**Setting:**

16 government designated covid-19 treatment centres in China, 11 to 29 February 2020.

**Participants:**

150 patients admitted to hospital with laboratory confirmed covid-19 were included in the intention to treat analysis (75 patients assigned to hydroxychloroquine plus standard of care, 75 to standard of care alone).

**Interventions:**

Hydroxychloroquine administrated at a loading dose of 1200 mg daily for three days followed by a maintenance dose of 800 mg daily (total treatment duration: two or three weeks for patients with mild to moderate or severe disease, respectively).

**Main outcome measure:**

Negative conversion of severe acute respiratory syndrome coronavirus 2 by 28 days, analysed according to the intention to treat principle. Adverse events were analysed in the safety population in which hydroxychloroquine recipients were participants who received at least one dose of hydroxychloroquine and hydroxychloroquine non-recipients were those managed with standard of care alone.

**Results:**

Of 150 patients, 148 had mild to moderate disease and two had severe disease. The mean duration from symptom onset to randomisation was 16.6 (SD 10.5; range 3-41) days. A total of 109 (73%) patients (56 standard of care; 53 standard of care plus hydroxychloroquine) had negative conversion well before 28 days, and the remaining 41 (27%) patients (19 standard of care; 22 standard of care plus hydroxychloroquine) were censored as they did not reach negative conversion of virus. The probability of negative conversion by 28 days in the standard of care plus hydroxychloroquine group was 85.4% (95% confidence interval 73.8% to 93.8%), similar to that in the standard of care group (81.3%, 71.2% to 89.6%). The difference between groups was 4.1% (95% confidence interval –10.3% to 18.5%). In the safety population, adverse events were recorded in 7/80 (9%) hydroxychloroquine non-recipients and in 21/70 (30%) hydroxychloroquine recipients. The most common adverse event in the hydroxychloroquine recipients was diarrhoea, reported in 7/70 (10%) patients. Two hydroxychloroquine recipients reported serious adverse events.

**Conclusions:**

Administration of hydroxychloroquine did not result in a significantly higher probability of negative conversion than standard of care alone in patients admitted to hospital with mainly persistent mild to moderate covid-19. Adverse events were higher in hydroxychloroquine recipients than in non-recipients.

**Trial registration:**

ChiCTR2000029868.

## Introduction

Coronavirus disease 2019 (covid-19), caused by severe acute respiratory syndrome coronavirus 2 (SARS-CoV-2), spread to most countries in the world within five months of initial reports in China. As of 22 April 2020, more than 2.5 million infections and 178 thousand deaths had been reported.^1^

Several drugs, including remdesivir, favipiravir, ribavirin, lopinavir-ritonavir (used in combination), and chloroquine or hydroxychloroquine, have been highlighted on the basis of promising in vitro results and therapeutic experiences from two other coronavirus diseases—severe acute respiratory syndrome and Middle East respiratory syndrome.^2^ However, none of these promising results has yet been translated into clinical benefits in patients with covid-19, with a failed trial of lopinavir-ritonavir being the most recently reported.^3^

Chloroquine and its hydroxyl analogue hydroxychloroquine, best known as antimalarial drugs, are prominent on the list of potential covid-19 treatments, owing to potent antiviral activity against SARS-CoV-2 in the in vitro studies and news reports of promising results from some ongoing trials.^4^ ^5^ ^6^ Despite their unclear benefits, chloroquine and hydroxychloroquine are both recommended for off label use in the treatment of covid-19 by the Chinese national guideline,^7^ and the US Food and Drug Administration recently authorised them for emergency use.^8^ US president Donald Trump also recently recommended use of hydroxychloroquine. Such a presidential endorsement stimulated an rapid increase in demand for hydroxychloroquine, which obscured the negative aspects of this drug. Deaths due to chloroquine overdoses have been reported in Nigeria among people self-treating for apparent covid-19.^9^ Retinopathy and gastrointestinal and cardiac side effects are well documented with the use of chloroquine or hydroxychloroquine in the treatment of malarial and rheumatic diseases.^10^ Hydroxychloroquine is preferred in clinical applications owing to its lower toxicity, particularly retinal toxicity,^10^ and three times the potency against SARS-CoV-2 infection compared with chloroquine in a recent in vitro study.^5^ No convincing evidence from well designed clinical trials exists to support the use of chloroquine or hydroxychloroquine with good efficacy and safety for the treatment of covid-19. Rapidly conducting such trials with high quality is challenging in the face of a dangerous coronavirus outbreak, in which healthcare workers have an overwhelming amount of work and the highest risk of developing covid-19.^11^

Having encountered many challenges, we conducted a multicentre, open label, randomised controlled trial to assess the efficacy and safety of hydroxychloroquine sulfate in adult patients with covid-19.

## Methods

### Trial oversight

The principal investigators (QX, GS, GN, and JQ) designed and initiated the study after approval of the protocol by the institutional review board in Ruijin Hospital on 6 February 2020. The protocol and approval documents are available in the online supplement. All patients gave written informed consent. The principal investigators invited hospitals with the capability to provide the current standard of care for covid-19 to participate in the study. Minimum requirements for the standard of care included the provision of intravenous fluids, supplemental oxygen, regular laboratory testing, SARS-CoV-2 testing, haemodynamic monitoring, and intensive care, as well as the ability to deliver concomitant medications. The trial was conducted urgently during the outbreak of covid-19 in compliance with the Declaration of Helsinki, Good Clinical Practice guidelines, and local regulatory requirements. Shanghai Pharmaceuticals Holding donated the investigated drug, hydroxychloroquine but was not involved in the study design, accrual, analyses of data, or preparation of the manuscript. A contract research organisation, R&G PharmaStudies, was hired to assist in the study design, data collection and cleaning, and statistical analyses. Data were recorded by clinical research coordinators, followed by queries from clinical research associates. The study statistician then entered confirmed data into the OpenClinica database for statistical analyses performed by and reviewed by the senior statistician in accordance with Good Clinical Practice guidelines. Data collection forms and statistical analysis plans are available in the online supplement.

An independent data and safety monitoring committee (IDMC) periodically reviewed the progress and oversight of the study. The interim analysis took place on 14 March, and the results were presented to the IDMC. The rapid decline in eligible new cases of covid-19 in China at that time precluded recruitment of our targeted number of patients. After a two round extensive review of the efficacy and safety data generated from the interim analysis, the IDMC endorsed an early termination of the trial. Members of the committee all agreed that the data from the trial are important for clinicians, the public, and the government to avoid inappropriate use of hydroxychloroquine in the clinical management of covid-19, particularly in areas with overwhelming patient numbers. The report of the trial could be an important resource to facilitate better design of future trials. The results of this clinical trial are reported in accordance with CONSORT (Consolidated Standards of Reporting Trials) guidelines.

### Trial design, randomisation, and procedures

This study was a multicentre, randomised, parallel, open label trial of hydroxychloroquine in patients admitted to hospital with covid-19. Patients were enrolled by the site investigators in 16 government designated covid-19 treatment centres in three provinces in China (Hubei, Henan, and Anhui). No placebo was used, and drugs were not masked. We applied stratified random sampling to stratify all eligible patients according to disease severity (mild/moderate or severe), followed by random assignment (in a 1:1 ratio) in each stratum to ensure a balanced distribution of disease severity between treatment (hydroxychloroquine plus standard of care) and control (standard of care only) groups. LL designed the randomisation rules together with the principal investigators, and an independent statistician who was not involved in data analysis implemented them. Equal numbers of cards with each group assignment number randomly generated by computer were placed in sequentially numbered envelopes that were opened as the patients were enrolled. All patients were managed with standard of care aligned with the indications from the updated national clinical practice guidelines for covid-19 in China. Patients in the treatment group were given hydroxychloroquine within 24 hours after randomisation, with a loading dose of 1200 mg daily for three days followed by a maintenance dose of 800 mg daily for the remaining days (total treatment duration was two weeks for patients with mild to moderate disease and three weeks for those with severe disease). The dose of hydroxychloroquine was adjusted when adverse events were related to hydroxychloroquine, as judged by investigators. Details of dose adjustment are provided in the study protocol available in the online supplement. Patients, investigators, and statisticians were not masked to treatment assignment. Laboratory technicians who did virological, chemical, and other routine measurements were unaware of treatment information.

### Patients

Inclusion criteria were age 18 years or older, ongoing SARS-CoV-2 infection confirmed in upper or lower respiratory tract specimens with real time reverse transcriptase polymerase chain reaction (RT-PCR), willingness to participate, and consent not to be enrolled in other clinical trials during the study period. Pneumonia on computed tomography of the chest was not mandatory for inclusion.

Exclusion criteria were age below 18 years; severe conditions including malignancies, heart, liver, or kidney disease or poorly controlled metabolic diseases; unsuitability for oral administration; pregnancy or lactation; allergy to hydroxychloroquine; inability to cooperate with investigators due to cognitive impairments or poor mental status; severe hepatic impairment (for example, Child Pugh grade C, alanine aminotransferase more than fivefold the upper limit); and severe renal impairment (estimated glomerular filtration rate ≤30 mL/min/1.73 m^2^) or receipt of continuous renal replacement therapy, haemodialysis, or peritoneal dialysis. In the original protocol, patients with severe covid-19 were excluded. Considering that the anti-inflammatory property of hydroxychloroquine might favour disease regression, we decided to include patients with severe covid-19 (change approved by the ethics committee on 17 February 2020).

The definition of disease severity of covid-19 was based on the fifth version of the Chinese guideline for the management of covid-19.^12^ Mild disease includes patients with mild symptoms but no manifestation of pneumonia on imaging. Moderate disease includes patients with fever, cough, sputum production, and other respiratory tract or non-specific symptoms along with manifestation of pneumonia on imaging but no signs of severe pneumonia defined as the presence of SaO_2_/SpO_2_ below 94% on room air or a PaO_2_ to FiO_2_ ratio of 300 or lower.

### Assessment

Specimens from the upper respiratory tract, lower respiratory tract, or both were obtained from each patient on screening (day –3 to day 1) and during treatment and post-treatment follow-up at scheduled visits on days 4, 7, 10, 14, 21, and 28. The local Centre for Disease Control and Prevention or authorised health institutions or hospitals at each site measured SARS-CoV-2 by using assays approved by the National Medical Products Administration. Measurements were performed according to the recommendations of the National Institute for Viral Disease Control and Prevention (China) (http://ivdc.chinacdc.cn/kyjz/202001/t20200121_211337.html). Methods for extraction and amplification of total RNA through RT-PCR were similar to those described elsewhere.^13^ Rather than quantitative data (cycle threshold value) reported from the RT-PCR assay, we collected only qualitative data reported from our trial sites. On the basis of a national recommendation, we defined a cycle threshold value less than 37 as a positive test result and a cycle threshold value of 40 or more as a negative test. Cycle threshold values between 37 and 40 confirmed by retesting were reported as unclassified.

In addition to SARS-CoV-2 testing, patients were assessed on each scheduled visit for vital signs, C reactive protein, erythrocyte sedimentation rate, tumour necrosis factor α, interleukin 6, complete blood cell count with differential, blood chemistry, coagulation panel, pulse oximetry, and respiratory symptoms. Records of administration of hydroxychloroquine and adverse events were reviewed daily to ensure fidelity to the protocol and, more importantly, patient safety. Computed tomography of the chest was assessed on screening and at the last visit of the treatment period (day 14 for patients with mild to moderate disease and day 21 for severe disease). Computed tomography examinations of the chest could be exempted by the investigators if the participants could provide qualified examination results within three days before the start of the study. More details on data collection are provided in the protocol available in the supplement.

### Outcome

The primary outcomes for this trial were whether patients had negative conversion of SARS-CoV-2 by 28 days and whether patients with severe covid-19 had clinical improvement by 28 days. However, as the trial was stopped early and only two patients with severe disease were enrolled, results on clinical improvement are not presented. We defined negative conversion of SARS-CoV-2 as two consecutive reports of a negative result for SARS-CoV-2 at least 24 hours apart without a subsequent report of a positive result by the end of the study. We considered the date of the first negative report as the date of negative conversion. In the original protocol, the primary endpoint was prespecified as the “Negative conversion rate by Day 10” (approved by the ethics committee on 6 February 2020). However, with the increasing knowledge of covid-19 from our clinical practice, we realised that the duration of SARS-CoV-2 in respiratory samples of many patients was longer than 10 days, recently highlighted by a detailed virological study.^14^ We therefore modified our primary outcome to test whether patients had a negative conversion of SARS-CoV-2 by 28 days (approved by the ethics committee on 17 February 2020). Probability of negative conversion at day 4, 7, 10, 14, or 21 was specified as a secondary outcome in the protocol, but this does not appear on the trial registration list. The listed secondary outcome in the trial registration was adverse events coded using the latest version of Medical Dictionary for Regulatory Activities coding dictionary, recorded in standard medical terminology and graded according to the National Cancer Institute Common Terminology Criteria for Adverse Events.

Other prespecified secondary outcomes not listed in the trial registration but included in the protocol were the probabilities of alleviation of clinical symptoms; improvement of C reactive protein, erythrocyte sedimentation rate, tumour necrosis factor α, interleukin 6, and absolute blood lymphocyte count; improvement of lung lesions on chest radiology; all cause death; and disease progression in patients with mild to moderate disease. The time frame for these secondary outcomes was from randomisation to 28 days. Prespecified secondary outcomes for patients with severe disease are not listed here but are included in the protocol. Owing to the early termination of our study, we could not justify the results from these analyses with an underpowered sample size and therefore decided not to emphasise them in this paper to avoid misinterpretation. The only one presented here is the alleviation of clinical symptoms within 28 days, which is an important outcome of interest prespecified in our protocol. The definition of the alleviation of clinical symptoms was resolving from fever to an axillary temperature of 36.6°C or below, normalisation of SpO_2_ (>94% on room air), and disappearance of respiratory symptoms including nasal congestion, cough, sore throat, sputum production, and shortness of breath.

### Statistical analysis

In the original protocol, the target number of enrolments was set to 200 without type I error control. In the updated protocol (approved by the ethics committee on 10 February 2020), we considered type I and type II error control and recalculated the sample size. The sample size was calculated on the basis of the alternative hypothesis of a 30% increase in the rate of conversion to negative for SARS-CoV-2 as defined by virus nucleic acid negativity. With the assumption that time to conversion follows an exponential distribution, if the median time to conversion with hydroxychloroquine can be reduced from 10 days to seven days, a total of 248 events would provide a power of 80% to detect a hazard ratio of 0.7 (standard of care *v* hydroxychloroquine) with a log rank test. Additionally, we assumed a 75 day accrual period and a seven day follow-up after enrolment of the last patient, so about 360 patients (180 per group) would be randomised in the study. An interim analysis was planned when around 150 patients were treated for at least seven days. We applied the O’Brien-Fleming cumulative α spending function by Lan-DeMets algorithm to control for family-wise type I error=0.05.

We estimated the overall probability of negative conversion by analysing time to negative conversion of SARS-CoV-2 using the Kaplan-Meier method in the intention to treat population and compared groups with a log rank test. We considered patients who did not reach the negative conversion of SARS-CoV-2 (see definition in Outcome section) by the cut-off date of the analysis (14 March 2020) to be right censored at the last visit date. All these patients remained in hospital and may have reached negative conversion after our last visit date, but the specific timing of the event is unknown. We applied similar approaches to the analysis of alleviation of symptoms. We used the rate difference between groups to show the treatment effect size and estimated 95% confidence intervals by an approximate normal distribution and the standard error by the bootstrap method (n=1000). We used a Cox model to estimate the hazard ratio. Hazard ratios greater than one indicate that the rate of negative conversion of virus or alleviation of symptoms was higher in the hydroxychloroquine plus standard of care group than in the standard of care only group. Safety analyses were based on the patients’ actual exposure to treatment. We used SAS version 9.4 for data analyses.

### Patient and public involvement

No patients were involved in setting the research question or the outcome measures, nor were they involved in developing plans for recruitment, design, or implementation of the study. No patients were asked to advise on interpretation or writing up of results. There are no plans to disseminate the results of the research to study participants or the relevant patient community.

## Results

### Patients

**Fig 1:**
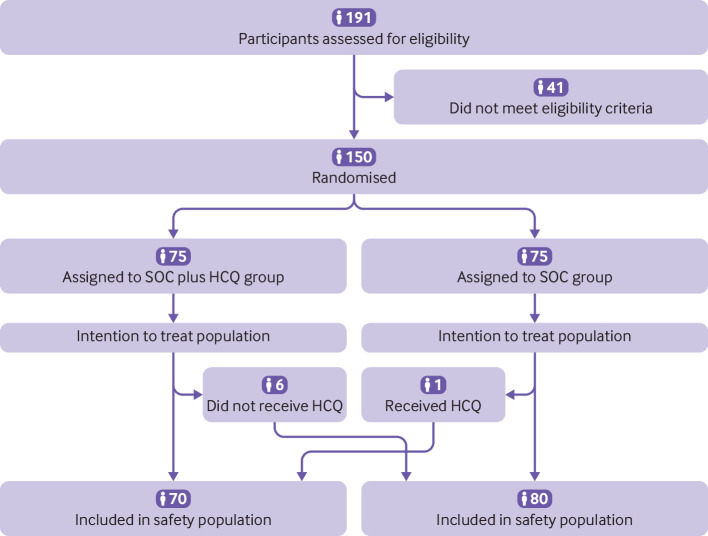
Screening, randomisation, and follow-up of trial participants

Of 191 patients admitted to hospital with covid-19 from 11 to 29 February 2020, 41 did not meet the eligibility criteria. The remaining 150 patients were randomised: 75 patients were assigned to standard of care and 75 patients to standard of care plus hydroxychloroquine (fig 1). The mean age of the patients was 46 years, and 82 (55%) were male. The mean duration from onset of symptoms to randomisation was 16.6 (SD 10.5; range 3-41) days, and 90 (60%) patients received concomitant drug treatment before randomisation. Among these, 52 (35%) patients received antiviral treatment (table 1). Almost all (148; 99%) patients had mild to moderate covid-19, and only 2 (1%) patients had severe disease on screening. Table 1 shows baseline demographic, epidemiological, and clinical characteristics of the patients in the two groups.

**Table 1:** Baseline demographic and clinical characteristics of patients in intention to treat population. Values are numbers (percentages) unless stated otherwise

Characteristics	SOC plus HCQ (n=75)	SOC (n=75)	Total (n=150)
Mean (SD) age, years	48.0 (14.1)	44.1 (15.0)	46.1 (14.7)
Male sex	42 (56)	40 (53)	82 (55)
Mean (SD) body mass index*	23.9 (3.24) (n=74)	23.2 (3.0) (n=71)	23.5 (3.2) (n=145)
Mean (SD) days from disease onset to randomisation	16.0 (9.9) (n=73)	17.1 (11.1) (n=74)	16.6 (10.5) (n=147)
Exposure history:			
Hubei province exposure	50/72 (69)	53/71 (75)	103/143 (72)
Contact with patients with confirmed covid-19	39/72 (54)	32/71 (45)	71/143 (50)
Others	1/72 (1)	1/71 (1)	2/143 (1)
No exposure	2/72 (3)	9/71 (13)	11/143 (8)
Unknown	5/72 (7)	5/71 (7)	10/143 (7)
Drug treatment before randomisation:	47 (63)	43 (57.3)	90 (60)
Antiviral agents	28 (37)	24 (32.0)	52 (35)
Arbidol	12 (16)	8 (11)	20 (13)
Lopinavir-ritonavir	18 (24)	14 (19)	32 (21)
Oseltamivir	3 (4)	3 (4)	6 (4)
Entecavir	1 (1)	0	1 (1)
Virazole	3 (4)	6 (8)	9 (6)
Ganciclovir	0	2 (3)	2 (1)
Disease severity:			
Mild	15 (20)	7 (9)	22 (15)
Moderate	59 (79)	67 (89)	126 (84)
Severe	1 (1)	1 (1)	2 (1)
Coexisting conditions:	28 (37)	17 (23)	45 (30)
Diabetes	12 (16)	9 (12)	21 (14)
Hypertension	6 (8)	3 (4)	9 (6)
Others	21 (28)	10 (13)	31 (21)
Vital signs—mean (SD):			
Body temperature, °C	36.9 (0.47) (n=72)	36.8 (0.48) (n=75)	36.8 (0.5) (n=147)
Pulse, beats/min	82.75 (8.0) (n=73)	82.5 (9.4) (n=71)	82.6 (8.7) (n=144)
Respiratory rate, breaths/min	19.6 (1.3) (n=73)	19.7 (1.7) (n=70)	19.6 (1.5) (n=143)
Systolic blood pressure, mm Hg	126.3 (13.2) (n=70)	123.5 (11.2) (n=69)	124.9 (12.3) (n=139)
Diastolic blood pressure, mm Hg	79.1 (8.5) (n=70)	76.8 (8.0) (n=69)	77.9 (8.3) (n=139)
Pulse oximetry, %	97.4 (1.6)	97.3 (1.6) (n=73)	97.4 (1.6) (n=148)
Symptoms:			
Fever	43/72 (60)	40 (53)	83/157 (53)
Cough	35/68 (51)	26/68 (38)	61/136 (45)
Sputum production	11/68 (16)	4/68 (6)	15/136 (11)
Shortness of breath	15/68 (22)	4/68 (6)	19/136 (14)
Nasal congestion	0	0	0
Pharynx discomfort	2/68 (3)	4/68 (6)	6/136 (4)
Fatigue	5/68 (7)	1/68 (1)	6/136 (4)
Laboratory parameters—mean (SD):			
White cell count, ×10^9^/L	5.59 (1.9)	5.6 (1.8)	5.6 (1.8)
Lymphocyte count, ×10^9^/L	1.46 (0.6)	1.6 (0.5)	1.5 (0.57)
Neutrophil count, ×10^9^/L	3.55 (1.6)	4.2 (6.2)	3.9 (4.51)
Platelet count, ×10^9^/L	214.8 (68.1)	211.7 (71.6)	213.2 (69.7)
Haemoglobin, g/L	128.8 (17.5)	129.1 (17.1)	129.0 (17.3)
Aspartate aminotransferase, U/L	25.0 (13.5)	26 (14.7)	25.5 (14.1)
Alanine aminotransferase, U/L	31.4 (26.3)	32.7 (25.2) (n=74)	32.1 (25.7) (n=149)
γ-glutamyl transpeptidase. U/L	46.9 (61.8) (n=73)	44.0 (51.8) (n=73)	45.4 (56.9) (n=146)
Total bilirubin, μmol/L	11.6 (8.4) (n=74)	12.8 (7.7) (n=73)	12.2 (8.1) (n=147)
Albumin, g/L	39.9 (4.5) (n=74)	40.4 (4.4) (n=74)	40.1 (4.4) (n=148)
Lactate dehydrogenase, U/L	203.9 (65.2) (n=66)	190.9 (49.5) (n=67)	197.4 (58.0) (n=133)
Creatine kinase, U/L	74.4 (110.1) (n=67)	71.0 (52.6) (n=68)	72.7 (85.7) (n=135)
Creatine kinase isoenzyme-MB, U/L	8.0 (4.2) (n=46)	6.8 (3.9) (n=44)	7.4 (4.0) (n=90)
Creatinine, μmol/L	71.2 (38.4) (n=74)	63.9 (16.0) (n=74)	67.5 (29.5) (n=148)
Blood urea nitrogen, mmol/L	3.5 (1.0) (n=44)	3.1 (0.7) (n=39)	3.3 (0.9) (n=83)
Urea, mmol/L	4.0 (3.0) (n=31)	3.8 (1.2) (n=32)	4.0 (2.2) (n=63)
International normalised ratio	1.0 (0.1) (n=73)	1.0 (0.1) (n=74)	1.0 (0.1) (n=147)
C reactive protein, mg/L	9.9 (13.3) (n=73)	7.4 (12.8) (n=74)	8.6 (13.1) (n=147)
Erythrocyte sedimentation rate, mm/h	30.6 (28.6) (n=72)	25.4 (21.7) (n=71)	28.0 (25.4) (n=143)
Tumour necrosis factor α, pg/mL	4.9 (4.1) (n=7)	4.8 (3.6) (n=7)	4.8 (3.7) (n=14)
Interleukin 6, pg/mL	12.9 (36.3) (n=31)	8.9 (13.0) (n=29)	11.0 (27.4) (n=60)

covid-19=coronavirus disease 2019; HCQ=hydroxychloroquine; SOC=standard of care.

To convert values for creatinine to mg/dL, divide by 88.4. To convert values for total bilirubin to mg/dL, divide by 17.1.

* Weight in kilograms divided by square of height in metres.

By 14 March 2020 (the cut-off date for data analysis), the median duration of follow-up was 21 (range 2-33) days in the standard of care group and 20 (3-31) days in the standard of care plus hydroxychloroquine group; nine patients in each group had completed 28 days of follow-up. Of the 75 patients assigned to receive standard of care plus hydroxychloroquine, six patients did not receive any dose of hydroxychloroquine (three withdrew consent and three refused to be treated with hydroxychloroquine). Concomitant treatments, including antiviral agents, antibiotics, and systemic glucocorticoid therapy, were similar in the two groups (table 2). One patient with moderate disease in the hydroxychloroquine group progressed to severe covid-19, and no patients died during follow-up.

**Table 2:** Treatments after randomisation in patients in intention to treat population. Values are numbers (percentages)

Treatments	SOC plus HCQ (n=75)	SOC (n=75)	Total (n=150)
Antiviral agents	47 (63)	48 (64)	95 (63)
Arbidol	37 (49)	33 (44)	70 (47)
Virazole	13 (17)	15 (20)	28 (19)
Lopinavir–ritonavir	13 (17)	12 (16)	25 (17)
Oseltamivir	8 (11)	9 (12)	17 (11)
Entecavir	1 (1)	1 (1)	2 (1)
Antibiotics	32 (43)	27 (36)	59 (39)
Systemic glucocorticoid treatment	6 (8)	4 (5)	10 (7)

HCQ=hydroxychloroquine; SOC=standard of care.

### Primary outcome

**Fig 2:**
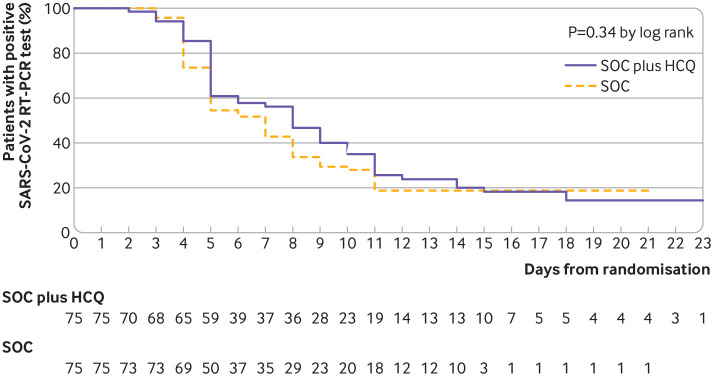
Kaplan-Meier curves of time to negative conversion of SARS-CoV-2 on real time reverse transcription polymerase chain reaction (RT-PCR) test in standard of care (SOC) plus hydroxychloroquine (HCQ) versus SOC in intention to treat population. Data are shown for 75 patients assigned to SOC plus HCQ and 75 assigned to SOC. Overall probability of negative conversion by 28 days was 85.4% (95% CI 73.8% to 93.8%) in SOC plus HCQ group and 81.3% (71.2% to 89.6%) in SOC group (P=0.34). Difference between groups was 4.1% (95% CI –10.3% to 18.5%). Median time to negative conversion was 8 (95% CI 5 to 10) days in SOC plus HCQ group and 7 (5 to 8) days SOC group (hazard ratio 0.85, 95% CI 0.58 to 1.23; P=0.34 by log rank test). Data from patients who did not have negative conversion were censored (tick marks) at last visit date

A total of 109 (73%) patients (56 standard of care; 53 standard of care plus hydroxychloroquine) had negative conversion well before 28 days, and the remaining 41 (27%) patients (19 standard of care; 22 standard of care plus hydroxychloroquine) were censored as they did not reach negative conversion of virus. The maximum duration for a patient with positive SARS-CoV-2 was 23 days by the cut-off date of our analysis. Overall, the probability of negative conversion of SARS-CoV-2 among patients who were assigned to receive standard of care plus hydroxychloroquine was 85.4% (95% confidence interval 73.8% to 93.8%) by 28 days, similar to that of the standard of care group (81.3%, 71.2% to 89.6%). The difference in the probability of negative conversion between standard of care plus hydroxychloroquine and standard of care alone was 4.1% (95% confidence interval –10.3% to 18.5%). The median time to negative conversion was also similar in the standard of care plus hydroxychloroquine group (8 (95% confidence interval 5 to 10) days) to that in the standard of care group (7 (5 to 8) days) (hazard ratio 0.85, 95% confidence interval 0.58 to 1.23; P=0.34 by log rank test; fig 2).

### Safety

Six patients who were assigned to the standard of care plus hydroxychloroquine group but did not receive hydroxychloroquine treatment were classified as hydroxychloroquine non-recipients in the safety population. One patient in the standard of care group wrongly received 14 days of hydroxychloroquine treatment with an accumulative dose of 11 600 mg. This patient was classified as a hydroxychloroquine recipient in the safety population (fig 1). We compared safety endpoints between hydroxychloroquine recipients and non-recipients (table 3). In hydroxychloroquine recipients, the median duration of hydroxychloroquine treatment was 14 (range 1-22) days. Between randomisation and the final visit, 21 (30%) patients in the standard of care plus hydroxychloroquine group reported adverse events, compared with 7 (9%) patients in the standard of care group (table 3). No serious adverse events were reported in the standard of care group. Two patients in the hydroxychloroquine group reported serious adverse events due to disease progression and upper respiratory infection. The patient with upper respiratory infection was discharged after finishing the 14 days of treatment with hydroxychloroquine and developed throat drying and pharyngalgia requiring readmission without evidence of pneumonia on computed tomography of the chest during the extended follow-up period.

**Table 3:** Summary of adverse events in safety population. Values are numbers (percentages)

Adverse events*	SOC plus HCQ (n=70)	SOC (n=80)
Any adverse event	21 (30)	7 (9)
Serious adverse event	2 (3)	0
Disease progression	1 (1)	0
Upper respiratory tract infection	1 (1)	0
Non-serious adverse event	19 (27)	7 (9)
Diarrhoea	7 (10)	0
Vomiting	2 (3)	0
Nausea	1 (1)	0
Abdominal discomfort	1 (1)	0
Thirst	1 (1)	0
Abdominal bloating	0	1 (1)
Sinus bradycardia	1 (1)	0
Hypertension	1 (1)	0
Orthostatic hypotension	1 (1)	0
Hypertriglyceridaemia	1 (1)	0
Decreased appetite	1 (1)	0
Fatigue	1 (1)	0
Fever	0	1 (1)
Dyspnoea	1 (1)	0
Flush	1 (1)	0
Liver abnormality	0	1 (1)
Kidney injury	1 (1)	0
Coagulation dysfunction	1 (1)	0
Hepatic steatosis	0	1 (1)
Otitis externa	0	1 (1)
Blurred vision	1 (1)	0
Decreased white blood cells	1 (1)	0
Increased alanine aminotransferase	1 (1)	1 (1)
Increased serum amylase	1 (1)	0
Decreased neutrophil count	1 (1)	0
Increased serum amyloid A	0	1 (1)

HCQ=hydroxychloroquine; SOC=standard of care.

* Multiple occurrences of same adverse event in one patient were counted.

The most common adverse event in the standard of care plus hydroxychloroquine group was diarrhoea, reported in 7 (10%) patients, which was not reported in the standard of care group. Hydroxychloroquine was discontinued in one patient owing to blurred vision and was adjusted to give a lower dose in one patient who reported thirst. These two adverse events were both transient with a duration of one to two days.

### Secondary outcome

**Fig 3:**
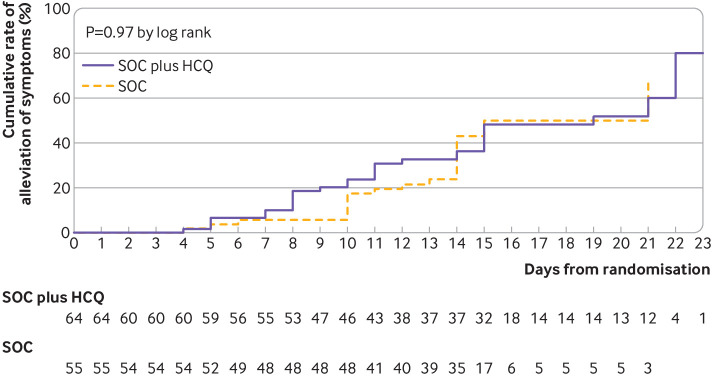
Kaplan-Meier curves of time to alleviation of clinical symptoms with standard of care (SOC) plus hydroxychloroquine (HCQ) versus SOC alone in intention to treat population. Data shown are for 55 patients with symptoms assigned to SOC plus HCQ and 64 assigned to SOC. Probability of alleviation of symptoms by 28 days was similar (P=0.97) in patients with SOC with (59.9%, 95% confidence interval 45.0% to 75.3%) and without HCQ (66.6%, 39.5% to 90.9%). Difference between groups was –6.6% (95% CI –41.3% to 28.0%). Median time to alleviation of clinical symptoms was similar in SOC plus HCQ group and SOC group (19 *v* 21 days; hazard ratio 1.01, 95% confidence interval 0.59 to 1.74; P=0.97 by log rank test). Data from patients who did not have alleviation of symptoms were censored (tick marks) at last visit date

Probability of negative conversion by a specific time point—4, 7, 10, 14, or 21 days—was also similar in the two groups (supplementary table A). The probability of alleviation of symptoms by 28 days was similar in patients with standard of care with hydroxychloroquine (59.9%, 95% confidence interval 45.0% to 75.3%) and without hydroxychloroquine (66.6%, 39.5% to 90.9%). The difference between groups was –6.6% (–41.3% to 28.0%). The median time to alleviation of clinical symptoms was similar in the standard of care plus hydroxychloroquine group to that in the standard of care group (19 *v* 21 days; hazard ratio 1.01, 0.59 to 1.74; P=0.97 by log rank test; fig 3).

## Discussion

This study (conducted during the outbreak of covid-19 in China) is the first randomised controlled trial evaluating administration of hydroxychloroquine in patients with covid-19. The findings do not provide evidence to support an increase in the probability of negative conversion of SARS-CoV-2 conferred by the addition of hydroxychloroquine administration to the current standard of care in patients admitted to hospital with mainly persistent mild to moderate covid-19.

### Comparison with other studies

The negative results on the antiviral efficacy of hydroxychloroquine obtained in this trial are contradictory to the encouraging in vitro results and the recently reported promising results from a non-randomised trial in 36 patients with covid-19.^4^ ^5^ ^6^ Participants in our trial had mainly mild to moderate disease, with a median 16 day delay between symptom onset and hydroxychloroquine treatment, so the negative results of our trial are applicable only to patients with persistently mild to moderate covid-19. Covid-19 has overwhelmed hospital systems during the pandemic, and many of these patients may be treated in the community and may finally be admitted to hospital because of a need for oxygen or owing to rapid deterioration of the disease. Data from our trial do not provide evidence to support the use of hydroxychloroquine in this population, particularly considering the increased adverse events (discussed below). Our trial could not assess the antiviral efficacy of hydroxychloroquine at an earlier stage, such as within 48 hours of onset of the illness, the golden window for antiviral treatment in influenza.^15^ However, conducting such a trial is challenging in patients admitted to hospital and will be easier in outpatient or community settings. The fact that antiviral treatment was not restricted in our trial should also be considered when interpreting our results. Seeing the antiviral effects of hydroxychloroquine when compared with a “pure” control arm would be more conclusive. In the early dangerous outbreak of covid-19 in China, however, setting a restriction on drugs that could be potentially useful was not ethical. Nevertheless, the use of antiviral drugs before and after randomisation was balanced between the hydroxychloroquine and standard of care groups and thus might have little effect on our primary endpoint. Moreover, use of a higher dose of hydroxychloroquine than was used in our trial is not likely to have additional antiviral effects, because the dosage of hydroxychloroquine that we chose achieves the 50% effective concentration (EC_50_) of hydroxychloroquine against SARS-CoV-2,^5^ although we did not monitor the concentration of hydroxychloroquine in our study. Another important message from our trial is that the Kaplan-Meier curve crossed over with time, suggesting a potential for a non-constant hazard ratio of negative conversion conferred by exposure to hydroxychloroquine. Therefore, the hazard ratios presented in our trial should be interpreted as weighted results over time rather than as a definitive constant. We will investigate this in the future. Further trials might also need to combat this problem when using the Cox regression method. Taken together, future studies could take advantage of our results to design trials in a more selective population, at the earliest stage possible (<48 hours from illness onset), and using more sensitive endpoints, such as viral load shedding.

Hydroxychloroquine in our trial was given at a loading dose of 1200 mg daily for three days followed by a maintenance dose of 800 mg daily for a total treatment duration of two weeks or three weeks for patients with mild to moderate or severe disease, respectively. Serious adverse events occurred in two patients, and both were reported in hydroxychloroquine recipients. The overall frequency of adverse events was significantly higher in hydroxychloroquine recipients than in non-recipients. Gastrointestinal events, particularly diarrhoea, were most commonly reported, as they were in another study using a high dose of hydroxychloroquine.^16^ Transient blurred vision was reported in one patient whose symptoms recovered two days after discontinuation of hydroxychloroquine. Early development of retinal damage with a daily dose of 800 to 1200 mg was detected using sensitive retinal screening tests.^17^ Therefore, the retinal damage could be underestimated in our trial. Events of cardiac arrhythmia, such as prolonged QT interval,^18^ were not observed in our trial, possibly because of the relatively mild to moderate disease of patients investigated or the short term period of follow-up. However, with increasing interest in the combined use of hydroxychloroquine and azithromycin worldwide, physicians should be cautious about the increased risk of prolongation of the QT interval and fatal ventricular arrhythmia with azithromycin and other antimicrobial agents.^19^ ^20^ Drug-drug interaction should be taken into consideration when assessing safety and efficacy endpoints in future trials of hydroxychloroquine.^10^ The effects of hydroxychloroquine in causing increased concentrations of digitoxin and metoprolol would be particularly relevant in patients with severe covid-19 and would therefore require close monitoring.^21^

### Strengths and limitations of study

This study provides the first and timely evidence about the benefit-risk profile of hydroxychloroquine derived from a multicentre randomised controlled trial, which was started during the most challenging time of the covid-19 outbreak in China. In such a situation, our study has several limitations. Firstly, the open label, as opposed to double blind, design introduces the possibility of biased investigator determined assessments and unbalanced dosage adjustment. Urgent production of placebos mimicking hydroxychloroquine and the management of a multicentre, placebo controlled trial remain challenging during the pandemic. Secondly, the use of sequential envelopes is inferior to the interactive web response management system for randomisation. Thirdly, the setting of our trial among patients admitted to hospital precluded us from enrolling patients at the early stage of disease. In addition, we cannot provide evidence on the effect of hydroxychloroquine on disease progression or regression because 148/150 (99%) patients in our trial had mild to moderate disease.

Fourthly, the results of our main prespecified outcomes are not entirely conclusive, being based on an underpowered sample size due to the lack of enough eligible patients to enrol. The recruitment of eligible patients was unexpectedly difficult, with many clinical trials launched in the same period in response to the urgent call by the national health authorities for the exploration of effective treatment against covid-19. The rapid decline in eligible new cases owing to the successful containment of covid-19 in the middle of March 2020 in China precluded further recruitment to reach our targeted sample size. The premature termination of our trial also led to increased censoring of data on our primary outcome. We therefore reanalysed the probability of negative conversion in the two groups by using follow-up data to 27 April 2020 (supplementary table B), and the results were consistent with our reported results.

Fifthly, ensuring fidelity to the protocol by site investigators under highly challenging circumstances in the covid-19 treatment centres was difficult. Hiring a contract research organisation, as we did in our trial, can greatly support the conduct and oversight of trials. Sixthly, population quarantine of Wuhan and neighbouring cities, nationwide travel restrictions, and isolation of cases and contacts were also barriers to collecting and transferring data and paper files. Several prespecified secondary endpoints, including the changes on computed tomography of the chest, were therefore not finished by the cut-off date for analysis. Finally, the specimens collected in our trial for virus RNA determination were mostly from the upper respiratory tract rather than bronchoalveolar lavage fluid, which could introduce false negative results.^22^ However, the prespecified definition for negative conversion of virus was two consecutive negative results at least 24 hours apart, which can reduce false negativity.

### Conclusion and policy implications

The results of our trial did not show additional benefits of virus elimination from adding hydroxychloroquine to the current standard of care in patients with mainly persistent mild to moderate covid-19. Adverse events, particularly gastrointestinal events, were more frequently reported in patients receiving hydroxychloroquine, who were given a loading dose of 1200 mg daily for three days followed by a maintenance dose of 800 mg daily for the remaining days for a total treatment duration of two weeks in patients with mild to moderate disease and three weeks in those with severe disease. Overall, these data do not support the addition of hydroxychloroquine to the current standard of care in patients with persistent mild to moderate covid-19 for eliminating the virus. Our trial may provide initial evidence for the benefit-risk profile of hydroxychloroquine and serve as a resource to support further research.

**What is already known on this topic**

The pandemic of coronavirus disease 2019 (covid-19) imposes a substantial burden on individuals, communities, healthcare facilities, markets, and governments globally
No specific treatment has been approved for covid-19, and no vaccine exists to prevent infection with SARS-CoV-2
During the urgent pandemic, media headlines encourage the use of drugs without solid evidence but ignore the side effects of these drugs

**What this study adds**

In this randomised clinical trial of patients with mainly persistent mild to moderate covid-19, exposure to hydroxychloroquine led to a similar probability of virus elimination to the current standard of care
Adverse events, mostly gastrointestinal, were significantly higher in patients who received hydroxychloroquine
Overall, the results do not support the use of hydroxychloroquine in patients with persistent mild to moderate covid-19

## Web extra

Extra material supplied by authors

**Web appendix:** Supplement

**Web appendix:** Supplementary tables

## Notes

Contributors: WT, ZC, MH, and ZW contributed equally to this paper and share joint first authorship. QX, GS, GN, and JQ contributed equally to this paper and share joint corresponding authorship. QX and GS were co-chief investigators of this trial. QX, GS, GN, and JQ led the development of the research question and study design and obtained the funding. WT, GS, QX, GN, JQ, and LL coordinated the operational delivery of the study protocol to the coordinating centres. LL led the process of data cleaning and analysis and took responsibility for the results in this manuscript and future analysis. ZC led and produced the first draft of this manuscript. MH, ZW, JC, WS, YW, WX, SL, EC, WC, XW, JY, JL, QZ, YY, ZX, DL, and YY represented the collaborating coordinating centres responsible for their centres’ participation in the trial. All authors provided critical review and final approval of the manuscript. The corresponding author attests that all listed authors meet authorship criteria and that no others meeting the criteria have been omitted. QX and GS are the guarantors.

Ethical approval: The trial was approved by the Institutional Review Board of Ruijin Hospital, Shanghai Jiao Tong University School of Medicine (KY2020–29). All patients gave written informed consent.

Data sharing: Anonymised datasets can be made available on reasonable request after approval from the trial management committee and after signing of a data access agreement. Proposals should be directed to the corresponding author.

The lead authors (the manuscript’s guarantors) affirm that the manuscript is an honest, accurate, and transparent account of the study being reported; that no important aspects of the study have been omitted; and that any discrepancies from the study as planned (and, if relevant, registered) have been explained.

Dissemination to participants and related patient and public communities: There are no plans to disseminate the results of the research to study participants or the relevant patient community.
