# Supplementary material for: Hydroxychloroquine in patients with mainly mild to moderate coronavirus disease 2019: open label, randomised controlled trial
Source: BMJ. 2020 May 14;369:m1849. doi: 10.1136/bmj.m1849 (PMC7221473; doi:10.1136/bmj.m1849)
Supplement: Supplementary file 1 — Web appendix: Supplement [file weit056844.ww1.pdf]

This supplement contains the following items:

Hydroxychloroquine in patients mainly with COVID-19 mild to moderate symptoms: an open-label, randomized, controlled trial (ChiCTR2000029868)

a. Original protocol, final protocol, summary of changes

b. Original SAP (No amendment of SAP until the study completion)

c. Final Case Report Form for mild/moderate and severe type

d. Final Patient consent form

Original Clinical Study Protocol: KY2020-29

Version date. 6 February 2020

This is the translated version of the original protocol

originally written in Chinese.

Clinical research protocol of hydroxychloroquine in the treatment of mild to moderate COVID-19 (Version 1.0)

1. Background

Since December 2019, Wuhan City, Hubei Province has found multiple cases of patients with pneumonia infected by a novel coronavirus (2019-nCoV). With the spread of the epidemic, other cases in China and abroad have also been found. [1] An important problem in the current diagnosis and treatment procedure is the lack of effective antiviral drugs. Early use of drugs with potential antiviral effects and clinically proven safety in human use is one of the fastest solutions to this problem.

Hydroxychloroquine (HCQ) is a 4-aminoquinoline antimalarial drug. It was artificially synthesized in 1944 and was first used for antimalarial treatment. Then it was used for systemic lupus erythematosus (SLE) in 1955 and in 1988. Rheumatoid arthritis has been widely recognized clinically for its role in regulating immunity and its safety and effectiveness. Hydroxychloroquine has similar effects to chloroquine (CQ), but the side effects are significantly reduced. [2] Not only in rheumatoid immune disease but also in antiviral process has HCQ shown obvious therapeutic effects, possibly by inhibiting TNF-α, IL-6 to treat acute respiratory distress syndrome (ARDS) caused by viral infection [3]. In special viral infections, such as HIV infection, chloroquine can reduce the inflammatory response in AIDS patients. [4] In related studies on SARS and MERS, HCQ has also shown possible antiviral efficacy at high doses. [5,6] Its antiviral action mechanism is to increase the pH of organelles such as lysosomes, interfere with intracellular iron metabolism, and prevent the virus from engulfing and shelling. At the same time, by increasing the pH of the Golgi apparatus, post-translational modification of viral proteins is inhibited, and intracellular pathogenic microorganisms are killed. [6,7] In related research on SARS virus, it can also prevent virus transmission by interfering with glycosylation of cellular receptors. [5] The safety of the drug and the distribution concentration in the tissue also perform well. [8, 9]

This study is intended to provide evidence for the therapeutic effect of high-dose hydroxychloroquine in patients with mild to moderate 2019-nCoV pneumonia. This project intends to conduct a prospective, multicenter, randomized controlled clinical trial to evaluate the drug viral negative rate, clinical symptom relief, improvement of laboratory test indicators, and prevention of increased disease severity are expected to confirm that the drug can be used as an antiviral therapy for patients with mild to moderate 2019-nCoV pneumonia.

2. Study content and methods

2.1 Primary objective

To evaluate the efficacy and safety of high dose of HCQ sulfate in patients with 2019-nCoV respiratory infection.

2.2 Study content

2.2.1 Design

A prospective, multicenter, randomized, open-label clinical trial. A total of 200 subjects will be randomized to oral hydroxychloroquine treatment group or control group in a 1:1 ratio.

2.2.2 Study population

Inclusion criteria

Patients will be enrolled in the trial if all of the following criteria are met:

1. ≥14 years old
2. Meet criteria for 2019-nCoV pneumonia (Laboratory tests for upper and lower respiratory tract RT-PCR confirmed 2019-nCoV nucleic acid test positive combining CT imaging examination)
3. Onset time ≤12 days
4. Sign the informed consent
5. No participation in other clinical researches within 28 days

Exclusion criteria

Patients will not be enrolled if one or more of the following criteria are met:

1. <14 years old
2. Meet the criteria of severe 2019-nCoV pneumonia
3. Combining other serious medical diseases such as malignant tumors, heart, liver and kidney diseases, metabolic diseases, etc.
4. Not suitable for gastrointestinal administration
5. (For female patients) ongoing pregnancy or lactation, or childbearing potential but unwillingness to adopt abstinence or contraception measures during the study
6. Allergic to the ingredients of hydroxychloroquine tablets
7. Mental illness, or other reasons for non-cooperation in the investigator’s opinion

2.2.3 Procedures:

Oral hydroxychloroquine group

Day 1: Oral hydroxychloroquine 2 tablets (250mg / tablet) twice a day;

Day 2 - Day 3: Oral hydroxychloroquine 2 tablets (250mg / tablet) twice a day;

Day 4 - Day 10: Oral hydroxychloroquine (250mg / tablet) 1 tablet once a day.

Control group

Supportive and symptomatic treatment for 2019-nCoV pneumonia.

2.2.4 Concomitant Treatments

Permitted

Appropriate supportive treatment(s) for 2019-nCoV pneumonia, that in the investigator's opinion will not interfere with the measurements contributing to the efficacy outcomes

Appropriate treatment(s) for any other underlying disease, that in the investigator's opinion will not interfere with the measurements contributing to the efficacy outcomes

If one or more treatments interfering with the measurements contributing to the primary efficacy outcomes are deemed necessary, the patient should not be randomized or should be discontinued from the study.

Not permitted

Antiviral drugs

2.2.5 Early Termination

The trail can be terminated early in particular individual if any of the following circumstance occurs:

- Treatment needs to be changed due to disease progression during study period after comprehensive assessment by investigators.
- Serious adverse events lead to disease progression
- Patient is no longer willing to be treated with the investigated drug
- Severe liver disease (e.g. Child Pugh grade C, AST>5 times upper limit)
- Patients with severe renal impairment (estimated glomerular filtration rate ≤30 mL/min/1.73 m2) or receiving continuous renal replacement therapy, hemodialysis, peritoneal dialysis

1.2.6 Study endpoints

Primary endpoint

Negative conversion rate of Sars-CoV-2 on day 10 of treatment (upper and lower respiratory tract nucleic acid detection negative rate)

Secondary endpoints

- Oral hydroxychloroquine treatment is superior to supportive therapy in change from baseline to day 4, day 7, day 10 of clinical symptoms (criteria for improvement of clinical symptoms: body temperature returned to normal, SaO2 returned to normal, respiratory symptoms were reduced by 50% from baseline);

- Oral hydroxychloroquine treatment is superior to supportive therapy in change from baseline to day 4, day 7, day 10 of laboratory indicators (CRP decreased by 50% from baseline, erythrocyte sedimentation decreased by 50%, and absolute peripheral lymphocyte count returned to normal);

- Oral hydroxychloroquine treatment is superior to supportive therapy in change from baseline to day 10 of improving rate of imaging (chest CT lesion absorption on day 10 of treatment is> 50% above baseline);

- Oral hydroxychloroquine treatment is not inferior to supportive therapy in change from baseline to day 4, day 7, day 10 of clinical exacerbation rate (the occurrence of severe / critical ill 2019-nCoV pneumonia).

Definition for severe and critically severe 2019-nCoV pneumonia：

A. Severe—Patients with 2019-nCoV pneumonia and at least one of the following：
    1. Respiratory distress，RR≥30 breaths per minute
    2. SpO2 ≤93% on room air
    3. Partial arterial oxygen pressure (PaO_2) / Fraction of inspired oxygen (FiO_2) ≤300mmHg (1mmHg=0.133kPa).

B. Critically severe—Patients with severe 2019-nCoV pneumonia and at least one of the following：
    1. Presence of lung failure requiring mechanical ventilation
    2. Presence of shock；
    3. Complicated with other organ failures requiring ICU monitoring

- Percentage of withdrawal from the study

1.2.7 Safety objectives

- Incidence of adverse events (Given that hydroxychloroquine is a commonly used anti-rheumatic and malaria parasite drug, the relevant adverse reactions have been fully documented, as shown in the following table):

Side-effects of tested drug

	HCQ sulphate
Eye	Long-term or high dose administration could increase the risk of irreversible retinopathy and visual impairment
Hearing	tinnitus
Musculoskeletal system	Sensory dyskinesia、Skeletal myopathy or neuromyopathy leads to progressive proximal muscle atrophy, diminished tendon reflexes,
Gastrointestinal system	Nausea, vomiting, diarrhea, bloating and burning sensation in the retrosternal area
Skin and Subcutaneous tissue	Rash, itching, skin and mucous membranes pigmentation disorders, hair bleaching or loss
hematological system	Bone marrow suppression, anemia, aplastic anemia, neutrophilic granulocytosis, leukopenia, thrombocytopenia
Nervous system	Dizziness, nystagmus, neurological aphasia、headache, epilepsy, ataxia and extrapyramidal diseases
Psychic side effects	Nervous, irritability, nightmare, psychosis, suicidal behavior
Cardiovascular system	Cardiomyopathy with possibility of heart failure and QT interval prolongation
Liver	Abnormality of liver function parameters
Immune system	Urticaria, angioedema, bronchospasm
Metabolism	Loss of appetite、hypoglycemia、porphyria、weight loss

- Percentage of withdrawal from the study

1.2.8 Statistical methods

Statistical analysis will be performed using SPSS or SAS software. The measurement data that follows the normal distribution will be described as mean ± standard deviation. The comparison between groups uses analysis of variance. The comparison of rank data uses Kruskal-Wallis-H rank sum test. The correlation analysis between measurement data uses Pearson. Correlation test was performed by Spearman method. $P < 0.05$ was considered statistically significant.

1.2.9 Time and Events Schedule (mild/moderate subgroup)

Phase		Screening/Baseline			
		Visit 1	Visit 2	Visit 3	Visit 4
Visits		Day -1~0	Day 4	Day 7	Day 10
General	Sign an informed consent	▲			
	Demographics	▲			
	Medical record	▲	▲	▲	▲
Clinical Symptoms	Maximum body temperature（℃）	▲	▲	▲	▲
	Visual score of respiratory symptoms	▲	▲	▲	▲
	SaO2（without oxygen intake）	▲	▲	▲	▲
	Urine pregnancy test (women of childbearing age)	▲			▲
Laboratory tests	C-reactive protein (CRP)	▲	▲	▲	▲
	ESR	▲	▲	▲	▲
	Peripheral blood lymphocyte count	▲	▲	▲	▲
	Imaging examination	▲			▲
Viral nucleic acid test	Upper / lower respiratory tract sampling	▲			▲
Disease severity assessment	Assess whether developed into severe or critical pneumonia	▲	▲	▲	▲
Inclusion/Exclusion criteria	Review inclusion / exclusion criteria	▲			
	Record adverse events		▲	▲	▲
	Record concomitant treatments	▲	▲	▲	▲
	Assign participants drug number	▲			
	Distributing or recalling research drugs	▲	▲	▲	▲

1.3 Risk and benefits

The current study is an open-label randomized trial for the evaluation of efficacy and safety of HCQ sulphate in the treatment of 2019-nCov respiratory infection. HCQ sulphate has been approved for clinical application as anti-inflammation and anti-microorganisms with good reported efficacy and safety profiles. We therefore anticipate that the administration of HCQ will not bring additional major risks.

Potential benefits：

1. Participants could benefit from the treatment group because the tested drug planned for investigation in this study is thought to offer potential benefits to individual subjects, based upon previous pre-clinical and several retrospective case-series investigations. With regard to control group, all patients were treated according to the latest guideline and therefore receive the best treatment at current stage.
2. For each participant, either assigned into treatment or control group, all the expenses for examination and treatment are covered by the sponsors.

Confidential:

Record of personal information will be kept private without publication according to legislation and laws. Results from this study, but not your identity, may be shared with local medical providers, government health organizations or international health workers to help them better understand 2019-nCoV infection.

References：

1.新型冠状病毒感染的肺炎诊疗方案（试行第五版）中华结核和呼吸杂志

2.Rainsford KD, et al. Therapy and pharmacological properties of hydroxychloroquine and chloroquine in treatment of systemic lupus erythematosus, rheumatoid arthritis and related diseases. Inflammopharmacology. 2015 Oct;23(5):231-69

3. A Savarino, JR Boelaert, A Cassone, et al. Effects of chloroquine on viral infections: drug against today's diseases? The Lancet Infectious Disease 2003.

4. Andrea Sarvarino, Lart Luca Shytaj. Chloroquine and beyond:exploring anti-rheumatic drugs to reduce immune hyperactivation in HIV/AIDS. Retrovirology 2015. 12-51.

5. MJ Vincent, E Bergeron, S Benjanne, et al. Chloroquine is a potent inhibitor of SARS coronavirus infection and spread. Virology Journal. 2005: 2:69.

6. J Dyall, CM Coleman, BJ Hart, et al. Repurposing of clinically developed drugs for treatment of middle east respiratory syndrome coronavirus infection. Antimicrobial agents and chemotherapy. 2014:58(8):4885-93.

7. Liang Shen, Junwei Niu, Chunghua Wang, et al. High throughput screening and identification of potent broad-spectrum inhibitors of coronaviruses. Journal of Virology June 2019 Volume 93 Issue 12

8. Mcchesney EW. Animal toxicity and pharmacokinetics of hydroxychloroquine sulfate. The American Journal of Medicine. 1983, 75(1): 11-18.

9. Mcchesney EW et al. Tissue distribution of chloroquine, hydroxychloroquine, and desethylchloroquine in the rat. Toxicol Appl Pharmacol. 1967, 10(3): 501-513

Original Clinical Study Protocol: KY2020-29

Version date. 6 February 2020

This is the original version of the protocol in Chinese.

羟氯喹治疗轻中度武汉新冠状病毒肺炎的临床研究方案

（V1.0 版）

一、研究背景：

2019 年 12 月以来，湖北省武汉市陆续发现了多例新型冠状病毒感染的肺炎患者，随着疫情的蔓延，我国其他地区及境外也相继发现了此类病例。【1】目前严峻的诊治形式中的重要环节-有效的抗冠状病毒药物的缺乏。尽早使用有潜在抗病毒效应且临床已经证明其在人体使用中的安全性的药物是解决这一问题的最快方案之一。

羟氯喹（hydroxychloroquine，HCQ）属 4-氨基喹啉类抗疟药，1944 年人工合成，最初用于抗疟治疗，1955 年开始用于治疗系统性红斑狼疮（ SLE ），1988 年开始用于治疗类风湿关节炎，其调节免疫的作用及安全性和有效性已经得到了临床的广泛认可。羟氯喹与氯喹（CQ）有类似的效果，但副作用明显降低。【2】不仅在风湿免疫病，在抗病毒方面，氯喹也有明显的治疗作用，可能通过抑制 TNFα、IL-6，治疗病毒感染引起的急性呼吸窘迫综合征（ARDS）【3】。在特殊病毒感染方面，如艾滋病病毒感染方面，氯喹可减轻艾滋病患者的炎症反应。【4】在冠状病毒 SARS 和中东呼吸道病毒的相关研究中，HCQ 也显示了高剂量使用可能存在的抗病毒疗效。【5,6】其抗病毒的作用机制在于通过提高溶酶体等细胞器的 PH 值，干扰细胞内铁代谢，阻止病毒的内吞、脱壳。同时通过升高高尔基体的 PH 值，抑制病毒蛋白翻译后的修饰，杀死胞内的病原微生物。【6,7】在 SARS 病毒的相关研究中，也可以通过干扰细胞受体的糖基化，阻止病毒传播。【5】其药物的安全性和组织中的分布浓度也有很好的表现。【8，9】

本研究拟在确诊的新冠状病毒肺炎的轻中度患者中，验证高剂量羟氯喹的治疗作用，本项目拟开展前瞻性、多中心、随机对照临床试验，评价该药物在新型冠状病毒肺炎的病毒转阴率、临床症状缓解、实验室检查指标的改善、预防疾病严重度增加的作用，预期可证实该药物可作为新型冠状病毒肺炎的轻中度患者抗病毒治疗用药。

二、研究内容和方法

（一）总体目标

本项目拟以高剂量羟氯喹治疗轻中度新型冠状病毒肺炎进行临床研究；对 200 例轻中度新型冠状病毒肺炎患者进行治疗有效性和安全性进行评价的临床试验，全面评价其在新型冠状病毒肺炎的临床作用。

（二）研究内容和方法：

1．1 研究目的：评价高剂量羟氯喹治疗轻中度新型冠状病毒肺炎的疗效和安全性。

1．2 试验内容

1．2．1 设计：按照 100 比 100 例，前瞻性、多中心、随机、开放对照研究。

1．2．2 研究人群

入组标准：

1）14 周岁以上。

2） 符合新型冠状病毒肺炎的诊断标准，【1】上和下呼吸道实验室检测 RT-PCR 证实 2019-nCov 核酸检测阳性，可以配合胸部 CT 影像学检查。

3）发病时间≦12 天。

4）签署知情同意书。

5）28 天内不参与其他研究药物的临床研究

排除标准

1）年龄＜14 岁患者；

2）符合重度新型冠状病毒肺炎的诊断标准的患者【1】

3）合并其他严重内科疾病如恶性肿瘤、心肝肾疾病，代谢性疾病等；

3）不宜胃肠道给药者；

4）妊娠期或哺乳期妇女；

5）对本品成分过敏者；

6）精神状态不能配合观察者或认知障碍者。

1、2、3 分组方法：

试验组：第一天口服羟氯喹（250mg/片）2 片，每日 2 次；第二天和第三天口服羟氯喹（250mg/片）2 片，每日 2 次；第四-第十天：（250mg/片）1 片，每日 1 次。

对照组：支持对症治疗

治疗周期：10 天

1.2.4 合并用药

治疗新型冠状病毒肺炎的支持对症药物，但不允许合并使用其他抗病毒药物。

1．2．5 提前终止

符合以下任何一种情况的均可提前结束研究，并记录结束时间

①研究期间出现疾病恶化，进展到重度肺炎，需要改变治疗

②药物不良事件的出现

③患者不愿意继续使用研究药物

④严重肝病(如 Child Pugh 评分≥C, AST>5 倍以上)

⑤已知严重肾功能损害的患者(估计为肾小球)滤过率≤30 mL/min/1.73 m^2)或连续肾脏替代疗法，血液透析，腹膜透析

1.2.6 研究终点

主要终点

1) 治疗第 10 天的新型冠状病毒检测转阴率（上、下呼吸道核酸检测转阴率）

次要终点

1）评估时间点的临床症状改善率：评估时间为治疗前，治疗第 4 天，治疗第 7 天，治疗第 10 天）

临床症状改善的标准：体温恢复正常，SaO_2 恢复正常，呼吸道症状较基线减轻 50%，

2）评估时间点的实验室指标改善率：评估时间为治疗前，治疗第 4 天，治疗第 7 天，治疗第 10 天）

C 反应蛋白（CRP）较基线下降 50%，血沉较基线下降 50%，外周血淋巴细胞绝对计数恢复正常。

3）影像学好转率：治疗第 10 天胸部 CT 病灶吸收较基线>50%

3）临床恶化率：转为重型/危重型新型冠状病毒肺炎的发生。（评估时间为治疗第 4 天，治疗第 7 天，治疗第 10 天）

注：重型和危重型新型冠状病毒肺炎的定义【1】:

A：重型——符合下列任何一条：

1.呼吸窘迫，RR≥30 次/分；

2.静息状态下，指氧饱和度≤93%;

3. 动 脉 血 氧 分 压 （ PaO2 ） / 吸 氧 浓 度 （ FiO2 ） ≤300mmHg （1mmHg=0.133kPa）。

B：危重型——符合以下情况之一者：

1.出现呼吸衰竭，且需要机械通气；

2.出现休克；

3.合并其他器官功能衰竭需 ICU 监护治疗。

安全性终点

①不良事件发生率（鉴于羟氯喹是临床常用的抗风湿及疟原虫药物，目前相关的不良反应已充分记载，具体如下表格）：

	羟氯喹
眼睛	长期或者高剂量服用会引发不可逆的视网膜损伤以及视觉障碍。
听力	耳鸣
肌肉骨胳系统	感觉运动障碍、骨骼肌肌病或神经肌病导致进行性弱和近端肌群萎缩、肌腱反射抑制和神经传导异常
胃肠系统	恶心、呕吐、腹泻、腹痛
皮肤和皮下组织等	皮疹、瘙痒、皮肤黏膜色素紊乱、毛色改变、脱发等
血液系统	骨髓衰竭、贫血、再生障碍性贫血、粒细胞增多、白细胞减少、血小板减少等
中枢神经系统	眩晕、眼球震颤、神经性失语、头痛、头晕、癫痫、共济失调和锥体外系疾病
精神类副作用	紧张、易怒、噩梦、精神病、自杀行为
心血管系统	心肌病并可能导致心力衰竭、QT 间期延长
肝脏	肝功能指标异常
免疫系统	荨麻疹、血管水肿、支气管痉挛
新陈代谢	食欲下降、低血糖、卟啉症、体重下降

②退出研究的比例

1．2．7 统计学处理：

采用 SPSS 或 SAS 软件进行统计学分析。遵循正态分布的计量资料，以均数±标准差描述，组间比较采用方差分析，等级资料的比较采用 Kruskal-Wallis-H 秩和检验，计量资料之间的相关分析采用 Pearson，等级资料相关分析采用 Spearman 法进行相关性检验，$P<0.05$ 为差异有统计学意义。

1．3 临床研究流程图

研究阶段		筛选入组/基线			
		访视 1	访视 2	访视 3	访视 4
访视		第-1 天~0 天	治疗第 4 天	治疗第 7 天	治疗第 10 天
一般情况	签署知情同意书	▲			
	人口学资料	▲			
	病史记录	▲	▲	▲	▲
临床症状	日最高体温（℃）	▲	▲	▲	▲
	呼吸道症状可视性评分	▲	▲	▲	▲
	SaO_2（不吸氧状态下）	▲	▲	▲	▲
	尿妊娠试验（育龄妇女）	▲			▲
实验室检查	C 反应蛋白（CRP）	▲	▲	▲	▲
	血沉	▲	▲	▲	▲
	外周血淋巴细胞绝对计数	▲	▲	▲	▲
	影像学	▲			▲
病毒核酸检测	上/下呼吸道采样	▲			▲
疾病严重度评估	评价是否转为重型或危重型肺炎	▲	▲	▲	▲
入排标准	审核入组/排除标准	▲			
	记录不良事件		▲	▲	▲
	记录合并用药	▲	▲	▲	▲
	分配受试者药物编号	▲			
	发放或回收研究药物	▲	▲	▲	▲

三．　受试者的风险与获益风险：

本项目是一项随机对照开放性研究，羟氯喹作为已经上市数年的免疫抑制剂和抗病毒药物，安全性和疗效已经得到了一定的认可，预计不会给受试者带来额外的风险。

可能的获益：

1. 参与本研究的受试者可能从治疗组获益，对照组采样常规支持对症治疗，与目前治疗指南一致，故均可获得现有阶段最佳的治疗疗效。

2. 在参加研究期间，受试者相关检查费用和研究药物的费用将由申办方承担。

保密：

受试者的个人信息记录将被保密，根据法律和法规，将不会被公开。如果研究结果发表，受试者的个人身份仍将保密。

参考文献：

1.新型冠状病毒感染的肺炎诊疗方案（试行第五版）中华结核和呼吸杂志

2. Rainsford KD,et al. Therapy and pharmacological properties of hydroxychloroquine and chloroquine in treatment of systemic lupus erythematosus, rheumatoid arthritis and related diseases. Inflammopharmacology. 2015 Oct;23(5):231-69

3. A Savarino, JR Boelaert, A Cassone, et al. Effects of chloroquine on viral infections: drug against today’s diseases? The Lancet Infectious Disease 2003.

4. Andrea Sarvarino, Lart Luca Shytaj. Chloroquine and beyond:exploring anti-rheumatic drugs to reduce immune hyperactivation in HIV/AIDS. Retrovirology 2015. 12-51.

5. MJ Vincent, E Bergeron, S Benjanne, et al. Chloroquine is a potent inhibitor of SARS coronavirus infection and spread. Virology Journal. 2005: 2:69.

6. J Dyall, CM Coleman, BJ Hart, et al. Repurposing of clinically developed drugs for treatment of middle east respiratory syndrome coronavirus infection. Antimicrobial agents and chemotherapy. 2014:58(8):4885-93.

7. Liang Shen, Junwei Niu, Chunghua Wang, et al. High throughput screening and identification of potent broad-spectrum inhibitors of coronaviruses. Journal of Virology June 2019 Volume 93 Issue 12

8. Mcchesney EW. Animal toxicity and pharmacokinetics of hydroxychloroquine sulfate. The American Journal of Medicine. 1983, 75(1): 11-18.

9. Mcchesney EW et al. Tissue distribution of chloroquine, hydroxychloroquine, and desethylchloroquine in the rat. Toxicol Appl Pharmacol. 1967, 10(3): 501-513

Final Clinical Study Protocol: KY2020-29

Version date. 20 February 2020

This is the translated version of the final protocol originally written in Chinese.

Evaluation of the Efficacy and Safety of Hydroxychloroquine sulfate in Adult Patients With 2019-nCoV Respiratory Infection: A Multicenter, Randomized, Controlled Study

(Version: 6.0)

1. Background

Since December 2019, an increasing number of cases diagnosed with "pneumonia of unknown etiology" have been identified in Wuhan, Hubei provinces, China. All these patients were subsequently confirmed with infection of a novel coronavirus (nCoV), of which, the full-length genome sequences share 79.5% identity to another CoV that caused Severe Acute Respiratory Syndrome (SARS) pandemic back in 2003 [1]. This nCov was then officially designated as "2019-nCoV" by the World Health Organization (WHO). The epidemiology of 2019-nCoV infection expanded to cities outside Wuhan and internationally [2].

The disease course and prognosis can be largely improved by the effective inhibition of virus replication, whereas no such drug has yet been approved to treat human CoV [3]. One of the best solutions to this problem is to use approved drugs with potential anti-viral effects and good safety profiles; among them, hydroxychloroquine (HCQ) is a promising candidate.

HCQ is a commonly used antimalarial drug, which together with chloroquine (CQ), belong to a class of drugs known as 4-aminoquinolines. After the first successful chemical synthesis of CQ in 1944, HCQ was generated by replacing ethyl with hydroxyethyl in the basic side chine of CQ. Comparing to CQ, HCQ is absorbed faster by the intestinal tract with a wider distribution in human body. Clinical applications of HCQ is first reported in antimalarial treatment and expands to systemic lupus erythematosus in 1955 and to rheumatoid arthritis in 1988. The immune modulatory effects,

safety and treatment efficacy of HCQ has been widely acknowledged [4]. Although HCQ and CQ have similar treatment efficacy, HCQ is preferred due its reduced toxicity [5,6] and its clinical applications have been expanded from rheumatic diseases to dermatology, malignancies and other fields because of the multifaceted effects on immune modulation, anti-inflammation, inhibiting of malignant cell growth and viability, enhancing clearance of lipid, anti-thrombosis and reducing diabetes risk [7,8]. In addition, CQ significantly reduces inflammatory response of SARS, possibly through the inhibition of TNFα and IL-6 [9]. Similarly, CQ is able to prevent an overexuberant inflammatory response in patients with human immunodeficiency virus (HIV) infection by inhibiting the HIV activated TLR-7 signaling pathway [10]. Treatment with HCQ (200–400 mg/day) for 8 weeks has been found to significantly reduce immune response, especially in patients with high viral load [11,12]. More importantly, high dose of HCQ treatment demonstrates anti-viral effects in studies investigating SARS-CoV and Middle East Respiratory Syndrome Coronavirus, (MERS-CoV) [13,14]. It has been proposed to use HCQ for the prophylaxis and treatment of SARS-CoV infection [13]. Animal studies demonstrate a survival rate of over 80% using CQ for the treatment of mice infected with MERS-CoV [14]. The main anti-viral mechanisms of HCQ include: 1) impairing the maturation of lysosomes and autophagosomes by increasing the pH of endosomal compartments; 2) interfering with cellular ferritin metabolism; 3) inhibiting posttranslational modification of virus protein by increasing the pH of Golgi apparatus. Moreover, CQ is able to inhibit p38 MAPK signaling pathway in HCoV-229e infected cell [14,16,17]. CQ is also reported to interfere with terminal glycosylation of the cellular receptor, angiotensin converting enzyme 2 in SARS-CoV, which negatively influence the virus-receptor binding and abrogate the infection [13]. Finally, animal studies have demonstrated good safety and tissue distribution of HCQ [18,19]。

The aim of current study was to evaluate the efficacy and safety of high dose HCQ in the treatment of patients with 2019-nCoV respiratory infection stratified into mild, moderate and severe group. A prospective, multicenter, randomized clinical trial was performed to evaluate the effects of

HCQ on negative conversion rate of virus RNA, improvement of clinical symptom and laboratory parameters and prevention of disease progression. We hypothesized that HCQ could be an effective drug in the treatment of 2019-nCoV respiratory infection.

2. Study content and methods

2.1 Primary objective

The primary objective of this study is to evaluate the efficacy and safety of high dose of HCQ sulfate in patients with 2019-nCoV respiratory infection by comparisons between the HCQ plus standard-of-care (SOC) group and the SOC group.

2.2 Study design

2.2.1 Aim

To evaluate the efficacy and safety of high dose of HCQ sulfate in patients with 2019-nCoV respiratory infection

2.2.2 Study content

2.2.2.1 Study design

This trial was a prospective, multicenter, open-label, randomized trial of patients with 2019-nCoV. The trial enrolled 360 patients with 1:1 randomization ratio stratified by disease severity (mild/moderate/severe) to receive either HCQ plus SOC or SOC alone.

2.2.2.2 Study population

Inclusion criteria：

1) Age ≥18 years
2) Upper or lower respiratory infection with 2019-nCoV as confirmed with real-time reverse-transcriptase–polymerase-chain-reaction (RT-PCR) with or without chest computed tomography examination；
3) Hospitalized with a SaO2/SPO2≤94% on room air or Pa02/Fi02 ratio <300mgHg (only for severe group)；

4) Willing to participate

5) Consent not to be enrolled by other clinical trials during study period

Exclusion Criteria:

1) Age <18 years
2) Patients with severe conditions including malignancies, heart/liver/kidney disease or poorly controlled metabolic diseases.
3) Not suitable to be administrated through GI tract
4) Pregnant or lactation
5) Allergy to HCQ
6) Unable to co-operate with investigators due to cognitive impairments or poor mental status
7) Severe liver disease (e.g. Child Pugh grade C, ALT>5 times upper limit)
8) Patients with severe renal impairment (estimated glomerular filtration rate ≤30 mL/min/1.73 m2) or receiving continuous renal replacement therapy, hemodialysis, peritoneal dialysis

2.2.2.3 Procedures

- SOC：SOC was aligned with the indications from the available clinical practice guidelines.
- SOC plus HCQ：HCQ was administrated in combination with SOC. Dose of HCQ: Day1~Day3：oral dose (100mg/tablet or 200mg/tablet) 400mg, three times a day; Day4~Day14/Day21：oral dose (100mg/tablet or 200mg/tablet) 400mg, twice a day.
- Duration：Mild or Moderate 14 days, Severe type 21-days.
- Dose adjustment: Treatment dose will be adjusted only when adverse events are related to the investigated drug as judged by investigators. In any case, investigator should perform evaluation carefully based on clinical experience. Treatment can be temporarily stopped or adjusted to a lower level of dose (switch from 400mg to 300mg), if the investigator considers a continued treatment with pre-defined dose will increase toxicity based on the individual safety and tolerance. Treatment can be restarted at original dose or at a lower level if the patients was recovered from

adverse events.

Table 2-1 Study Drug Administration

	Original dose level	Adjusted dose level
Day 1-3	1200mg/day<br>three times/day，400mg/tablet	900mg/day<br>three times/day，300mg/tablet
Day 4~Day 14/21	800mg a day<br>twice/day，400mg/tablet	600mg a day<br>twice/day，300mg/tablet

The investigators should also evaluate the clinical benefits to decide whether continues treatment until Day14/21 in cases with disease progression.

2.2.2.4 Concomitant drugs

All standard drugs that are recommended by the guideline can be used for SOC, except for other drugs belongs to 4-aminoquinolines, *e.g.*, CQ phosphate. Patients who have received CQ can be enrolled after 48h of withdrawal. Medications for diseases other than 2019-nCoV respiratory infection can be used during the study period.

2.2.2.5 Early Termination

The trail can be terminated early if any of the following circumstance occurs:

① Treatment needs to be changed due to disease progression during study period after comprehensive assessment by investigators.

② Serious adverse events lead to disease progression

③ Patient is no longer willing to be treated with the investigated drug

2.2.2.6 Endpoints

Primary endpoint: Negative conversion rate of virus nucleic acid within 28-day

For severe disease subgroup, considering both virology conversion and clinical improvement.

- Definition for clinical improvement：decline of two categories from enrollment status on a six-category ordinal scale of clinical status which ranging from 1 (discharged) to 6 (death)

Table 2-2 Six-category ordinal scale

Scale	Description
6	Death
5	ICU, requiring ECMO and/or IMV
4	ICU/hospitalization, requiring NIV/ HFNC therapy
3	Hospitalization, requiring supplemental oxygen (but not NIV/ HFNC)
2	Hospitalization, not requiring supplemental oxygen;
1	Discharge

Abbreviation: IMV, invasive mechanical ventilation; NIV, non-invasive mechanical ventilation; HFNC, High-flow nasal cannula.

Secondary endpoint：

1) Negative conversion rate of virus nucleic acid at day 4, 7, 10, 14 or 21.
2) Improvement rate of clinical symptoms within 28-day
    - Definition for clinical symptoms improvement: resolving from fever (axillary temperature ≤36.6°C， or oral temperature ≤37.2°C, or rectal/ear temperature ≤37.8°C), normalization of SpO_2 (SpO_2 >94% on room air), disappearance of respiratory symptom.
3) Improvement rate of laboratory parameters within 28-day
    - Definition for laboratory parameters improvement: 50% decline of C-reactive protein, erythrocyte sedimentation rate, IL-6 and TNF-α level from baseline and normalization of absolute blood lymphocyte count.
4) Improvement rate of chest radiology within 28-day
    - Definition for radiology improvement: Resolved more than 50% from baseline on chest CT images
5) All-cause mortality
6) Clinical status at Day 7, 14, 21, 28 in patients with severe 2019-nCoV respiratory infection
7) For severe disease subgroup: Duration (days) of mechanical ventilation, extracorporeal membrane oxygenation, supplemental oxygenation and hospital stay
8) For mild/moderate subgroup: Progression rate within 28-day

Definition for progression：progress to severe or critically severe.

Definition for severe and critically severe 2019-nCoV pneumonia [3]：

C. Severe—Patients with 2019-nCoV pneumonia and at least one of the following：

   4. Respiratory distress，RR≥30 breaths per minute
   5. SpO2 ≤93% on room air
   6. Partial arterial oxygen pressure (PaO_2) / Fraction of inspired oxygen (FiO_2) ≤300mmHg (1mmHg=0.133kPa).

D. Critically severe—Patients with severe 2019-nCoV pneumonia and at least one of the following：

   4. Presence of lung failure requiring mechanical ventilation
   5. Presence of shock；
   6. Complicated with other organ failures requiring ICU monitoring

Safety endpoint：

① AE rate

Given that HCQ sulphate has been widely used in antimalarial and rheumatoid arthritis treatment, the side-effects of HCQ have been fully established as listed in Table 2-3. Among all the side-effects, gastrointestinal upset, particularly diarrhea, and retinopathy are relative common at a regular dose but usually resolve after treatment is stopped.

② Dropout rate

Table 2-3 Side-effects of tested drug

	HCQ sulphate
Eye	Long-term or high dose administration could increase the risk of irreversible retinopathy and visual impairment
Hearing	tinnitus
Musculoskeletal system	Sensory dyskinesia、Skeletal myopathy or neuromyopathy leads to progressive proximal muscle atrophy, diminished tendon reflexes,
Gastrointestinal system	Nausea, vomiting, diarrhea, bloating and burning sensation in the retrosternal area
Skin and Subcutaneous tissue	Rash, itching, skin and mucous membranes pigmentation disorders, hair bleaching or loss
hematological system	Bone marrow suppression, anemia, aplastic anemia, neutrophilic granulocytosis, leukopenia, thrombocytopenia
Nervous system	Dizziness, nystagmus, neurological aphasia、headache, epilepsy, ataxia and extrapyramidal diseases
Psychic side effects	Nervous, irritability, nightmare, psychosis, suicidal behavior
Cardiovascular system	Cardiomyopathy with possibility of heart failure and QT interval prolongation
Liver	Abnormality of liver function parameters
Immune system	Urticaria, angioedema, bronchospasm
Metabolism	Loss of appetite、hypoglycemia、porphyria、weight loss

2.2.2.7 Statistical Methods

Sample Size Justification：

Approximately 360 subjects (180 per group) will be randomized in the study. The sample size for this study is based on the alternative hypothesis of a 30% reduction of time to virus nucleic acid negativity. Under the exponential distribution, this benefit translates to a reduction in median time to virology negativity from 10 days to 7 days. A total of 248 events would provide a power of 80% to detect a 30% reduction of hazard ratio (control vs. treatment) of 0.70 with a Log-Rank test, assuming a two-sided significance level of 5%. A 75-day accrual period and an additional 7-day follow-up are assumed. The sample size calculation has also taken into consideration an annual dropout rate of 20%.

A co-primary endpoint in the subgroup defined by severe disease is 28-day clinical improvement. Assume 50% patients have clinical improvement by day 28 in control group, 100 patients can detect a 25% improvement in treatment group with a power=70%.

Analysis Set：

- Intent-to-treat (ITT): is defined as subjects who have been randomly assigned to the treatment group or control group. Analyses of efficacy will be based on this population. The ITT analyses grouping will be according to treatment planned.
- Per Protocol Set (PPS): is a subset of ITT， defined as all subjects who have been randomly assigned to the study and received at least 11-day treatment (mild/moderate disease)/16-day treatment (severe disease)， and have no major PD which significantly impact primary endpoint. The analyses grouping will be according to treatment planned.
- Safety population: is defined as subjects who have received at least 1 administration of any study treatment (partial or complete). This population will be used for all safety analyses. The safety analyses grouping will be according to treatment actually received.

Efficacy Analysis：

- **Primary Efficacy Analysis：**

The Kaplan-Meier method will be used to estimate the distribution of time to negativity for each treatment group. The median time to negativity with 95% CI will be provided. In addition, the number and percentage of subjects who have an event or are censored will be reported. The Kaplan-Meier curve will also be plotted by treatment group and will be compared by the stratified log-rank test. The stratification factor is the same as factor considered in the randomization procedure.

In the severe disease subgroup, the rate of clinical improvement by 28-day will be

summarized and compared by Chi-square test (or Exact test, if frequency is low). As the supporting analysis, KM method may be applied to estimate the proportion of clinical status improvement by day 28.

- **Secondary Efficacy Analysis：**

Report the rates and relative risk and their 95% confidence interval by the day of 4,7,10,14,21 for secondary endpoints (Negative conversion rate of virus nucleic acid, Improvement rate of clinical symptoms, Improvement rate of laboratory parameters, Improvement rate of chest radiology, All-cause mortality, Clinical status change, progression, etc). Descriptive statistics (mean, standard deviation, median, range) will be used to summarize duration (days) of mechanical ventilation, extracorporeal membrane oxygenation, supplemental oxygenation and hospital stay. If applicable, KM method will be used to analyze time-to-events type endpoints. Median and 95% CI may be reported. KM curves will be compared by log-rank test.

Safety：

The incidence and prevalence of AEs, TEAEs, SAEs and ADRs will be summarized overall, by treatment group, by MedDRA (the latest version) system organ class and preferred term (PT).

Descriptive statistics (mean, standard deviation, median, range) will be used to summarize observed laboratory values, ECG, vital signs and physical examination findings and change from baseline in observed value at each scheduled visit for each treatment group. When applicable, shift table for baseline value (normal vs abnormal but not clinical meaningful vs. abnormal and clinical meaningful) and post-baseline value will be provided.

Interim Analysis：

An Independent Data Monitoring Committee (IDMC) will be established to review data at 1 interim analyses prior to the primary endpoint analysis.

The O’Brien-Fleming type α-spending function by Lan-DeMets algorithm（Lan-Demets，1983）

will be applied to control family-wise type-I error which is 0.05, two-sided, in this study.

The first interim analysis will occur after 40% of subjects (around 150 subjects) have been treated for 7 days or have discontinued. The goal is to evaluate efficacy and need of sample size re-estimation. p-value boundary for efficacy significance is 0.00079 (two-sided).

Final analysis will be conducted after 248 events are observed. p-value boundary is 0.04974 (two-sided).

The actual α spent and p-value boundaries at each analysis will be calculated based on the exact observed number of events.

2.2.2.8 Study Schematic

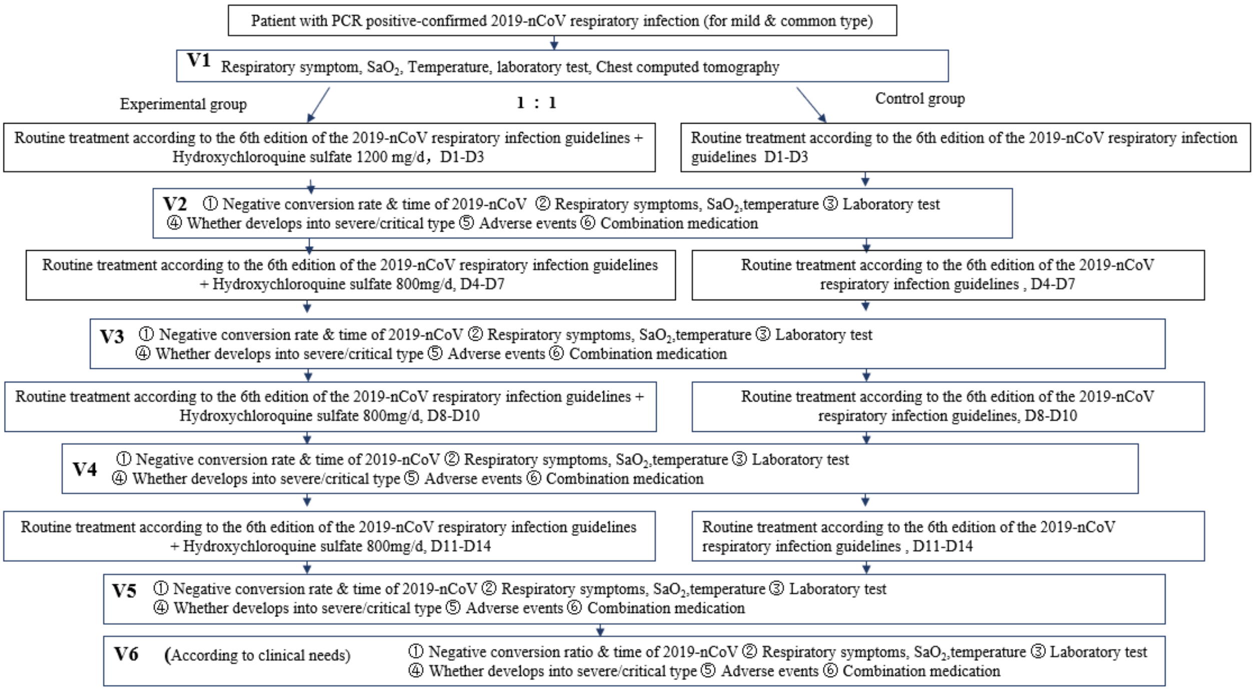

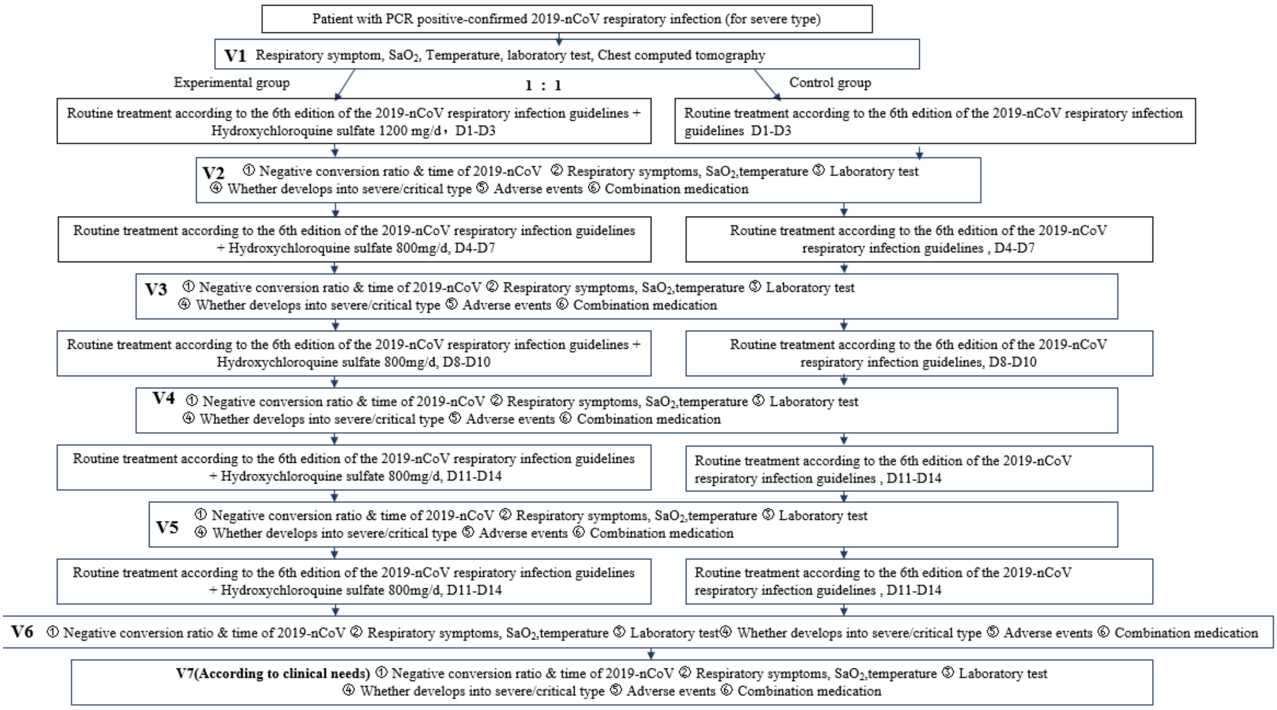

2.3 Time and Events Schedule

Table 2-3 Time and Events Schedule (mild/moderate subgroup)

**Evaluation / Procedure**	**Screen/ Treatment**	**Treatment and follow-up (Day 2-14)**				**Extended follow-up**[13]	
Visit	V1	V2	V3	V4	V5	V6	V7
Day +/- Window	D-3~1	D4±1d	D7±1d	D10±1d	D14±1d	D21±2d	D28±2d
Informed Consent	×						
I/E Criteria	×						
Randomization	×						
Demographics[1]	×						
Epidemiological and medical history[2]	×						
Physical examination[3]	×				×	×	×
Vital signs[4]	×	×	×	×	×	×	×
12-lead electrocardiogram	×[11]				×	×	×
CRP, ESR	×[11]	×	×	×	×	×	×
TNF-α，IL-6	×[11]	×	×	×	×	×	×
CBC with differential[5]	×[11]	×	×	×	×	×	×
Urinalysis[6]	×[11]				×	×	×
Blood chemistry[7] (hepatic and kidney panel, Myocardial enzyme)	×[11]	×	×	×	×	×	×
Coagulation panel[8]	×[11]	×	×	×	×	×	×
2019-nCoV RT-PCR[9]	×[12]	×	×	×	×	×	×
Pulse oximetry（on room air）	×	×	×	×	×	×	×
Pregnancy Test (Serum or Urine hCG)	×[11]				×		
Chest CT	×[11]				×	Optional (*as per* clinical needs)	
Respiratory symptom (Y/N)	×	×	×	×	×	×	×
Study drug administration [10]	×	×	×	×	×		
Progression to severe/critically severe type (Y/N)		×	×	×	×	×	×
Record of SMT	×	×	×	×	×	×	×
AE Assessment	×	×	×	×	×	×	×

1. Demographics include birth date, gender and ethnicity.
2. Epidemiological information includes recent travel history and exposure to epidemic area or infected patients; medical history includes present illness and past medical history including co-morbidity medications
3. Physical examination includes height, weight, general outlook, head, eye, ear, nose, throat, neck and thyroid, skin, cardiovascular system, Anterior chest and lung, abdomen, neuro system, skeletal muscle system and lymphatic system
4. Vital signs: body temperature, respiratory rate, pulse, blood pressure; record maximum temperature of the visiting day and use same measurement criteria throughout the study period.
5. CBC with differential includes: haptoglobin and absolute count of red blood cell, platelet, total white blood cell, neutrophil, lymphocyte, monocyte, eosinophil and basophil
6. Urinalysis: urinary pH, urinary red blood cell, urinary white blood cell, urinary protein, glucose and ketone
7. Blood chemistry includes alanine aminotransferase, aspartate aminotransferase, total bilirubin, albumin, γ-glutamyl transpeptidase, lactate dehydrogenase, creatine kinase, creatine kinase isoenzyme-MB, blood urea nitrogen and creatinine
8. Coagulation panel includes: Prothrombin time, activated partial thrombin time, thrombin time, fibrinogen and international normalized ratio.
9. RT-PCR results upon unplanned visit should also be recorded in CRF
10. Record daily administration of tested drug including under use and over use
11. These examinations can be exempted by the investigators if the participants can provide qualified examination results within 3-day prior to initiation of the study
12. RT-PCR results of 2019-nCoV within 5-day prior to initiation of the study can be used for screening
13. For patients discharge alive, only medications and AE information will be collected *via* telephone contact.

Table 2-3 Time and Events Schedule (severe subgroup)

**Evaluation / Procedure**	**Screen/ Treatment**	**Treatment and follow-up (Day 2-14)**					**Extended follow-up[13]**
Visit	V1	V2	V3	V4	V5	V6	V7
Day +/- Window	D-3~1	D4±1d	D7±1d	D10±1d	D14±1d	D21±2d	D28±2d
Informed Consent	×						
I/E Criteria	×						
Randomization	×						
Demographics[1]	×						
Epidemiological and medical history[2]	×						
Physical examination[3]	×					×	×
Vital signs[4]	×	×	×	×	×	×	×
12-lead electrocardiogram	×[11]					×	×
CRP, ESR	×[11]	×	×	×	×	×	×
TNF-α，IL-6	×[11]	×	×	×	×	×	×
CBC with differential[5]	×[11]	×	×	×	×	×	×
Urinalysis[6]	×[11]					×	×
Blood chemistry[7] (hepatic and kidney panel, Myocardial enzyme)	×[11]	×	×	×	×	×	×
Coagulation panel[8]	×[11]	×	×	×	×	×	×
2019-nCoV RT-PCR[9]	×[12]	×	×	×	×	×	×
Pulse oximetry（on room air）	×	×	×	×	×	×	×
Arterial blood gas analysis	×[11]	Optional (*as per* clinical needs)					
Pregnancy Test (Serum or Urine hCG)	×[11]					×	
Chest CT	×[11]					×	Optional (*as per* clinical needs)
Respiratory symptom (Y/N)	×	×	×	×	×	×	×
Study drug administration [10]	×	×	×	×	×	×	
Clinical status	×		×		×	×	×
IMV/NIV, ECMO or supplemental oxygen (Y/N)	×	×	×	×	×	×	×
Record of SMT	×	×	×	×	×	×	×

AE Assessment	×	×	×	×	×	×	×

1. Demographics include birth date, gender and ethnicity.
2. Epidemiological information includes recent travel history and exposure to epidemic area or infected patients; medical history includes present illness and past medical history including co-morbidity medications
3. Physical examination includes height, weight, general outlook, head, eye, ear, nose, throat, neck and thyroid, skin, cardiovascular system, Anterior chest and lung, abdomen, neuro system, skeletal muscle system and lymphatic system
4. Vital signs: body temperature, respiratory rate, pulse, blood pressure; record maximum temperature of the visiting day and use same measurement criteria throughout the study period.
5. CBC with differential includes: haptoglobin and absolute count of red blood cell, platelet, total white blood cell, neutrophil, lymphocyte, monocyte, eosinophil and basophil
6. Urinalysis: urinary pH, urinary red blood cell, urinary white blood cell, urinary protein, glucose and ketone
7. Blood chemistry includes alanine aminotransferase, aspartate aminotransferase, total bilirubin, albumin, γ-glutamyl transpeptidase, lactate dehydrogenase, creatine kinase, creatine kinase isoenzyme-MB, blood urea nitrogen and creatinine
8. Coagulation panel includes: Prothrombin time, activated partial thrombin time, thrombin time, fibrinogen and international normalized ratio.
9. RT-PCR results upon unplanned visit should also be recorded in CRF
10. Record daily administration of tested drug including under use and over use
11. These examinations can be exempted by the investigators if the participants can provide qualified examination results within 3-day prior to initiation of the study
12. RT-PCR results of 2019-nCoV within 5-day prior to initiation of the study can be used for screening

For patients discharge alive, only medications and AE information will be collected *via* telephone contact.

2.3 Risk and benefits

The current study is an open-label randomized trial for the evaluation of efficacy and safety of HCQ sulphate in the treatment of 2019-nCov respiratory infection. HCQ sulphate has been approved for clinical application as anti-inflammation and anti-microorganisms with good reported efficacy and safety profiles. We therefore anticipate that the administration of HCQ will not bring additional major risks.

Potential benefits：

Participants could benefit from the treatment group because the tested drug planned for

investigation in this study is thought to offer potential benefits to individual subjects, based upon previous pre-clinical and several retrospective case-series investigations. With regard to control group, all patients were treated according to the latest guideline and therefore receive the best treatment at current stage.

For each participant, either assigned into treatment or control group, all the expenses for examination and treatment are covered by the sponsors.

Confidential:

Record of personal information will be kept private without publication according to legislation and laws. Results from this study, but not your identity, may be shared with local medical providers, government health organizations or international health workers to help them better understand 2019-nCoV infection.

Reference：

1. Zhou P, Yang XL, Wang XG, et al. A pneumonia outbreak associated with a new coronavirus of probable bat origin [published online ahead of print, 2020 Feb 3]. Nature. 2020; doi:10.1038/s41586-020-2012-7

2. Wang C, Horby PW, Hayden FG, Gao GF. A novel coronavirus outbreak of global health concern [published online ahead of print, 2020 Jan 24]. Lancet. 2020; doi:10.1016/S0140-6736(20)30185-9.

3.国家卫生健康委员会. 新型冠状病毒感染的肺炎诊疗方案（试行第六版）, http://www.gov.cn/zhengce/zhengceku/2020-02/05/content_5474791.html, 2020.02.05 发行

4. Leden I. Antimalarial drugs-350 years. Scand J Rheumatol. 1981;10(4):307–312.

5. Rainsford KD, Parke AL, Clifford-Rashotte M, et al. Therapy and pharmacological properties of hydroxychloroquine and chloroquine in treatment of systemic lupus erythematosus, rheumatoid arthritis and related diseases. Inflammopharmacology. 2015;23(5):231–269

6. 赵义,李小霞, 用辩证的眼光看待硫酸羟氯喹的用药安全, 药物不良反应杂志 2015;17(3): 164-166.

7. Ben-Zvi I, Kivity S, Langevitz P, Shoenfeld Y. Hydroxychloroquine: from malaria to autoimmunity. Clin Rev

Allergy Immunol. 2012;42(2):145–153.

8. Olsen NJ, Schleich MA, Karp DR. Multifaceted effects of hydroxychloroquine in human disease. Semin Arthritis Rheum. 2013;43(2):264–272.

9. Savarino A, Boelaert JR, Cassone A, Majori G, Cauda R. Effects of chloroquine on viral infections: an old drug against today's diseases?. Lancet Infect Dis. 2003;3(11):722–727.

10. Savarino A, Gennero L, Sperber K, Boelaert JR. The anti-HIV-1 activity of chloroquine. J Clin Virol. 2001;20(3):131–135.

11. Savarino A, Shytaj IL. Chloroquine and beyond: exploring anti-rheumatic drugs to reduce immune hyperactivation in HIV/AIDS. Retrovirology. 2015;12:51, doi: 10.1186/s12977-015-0178-0.

12. Akpovwa H. Chloroquine could be used for the treatment of filoviral infections and other viral infections that emerge or emerged from viruses requiring an acidic pH for infectivity. Cell Biochem Funct. 2016;34(4):191–196.

13. Vincent MJ, Bergeron E, Benjannet S, et al. Chloroquine is a potent inhibitor of SARS coronavirus infection and spread. Virol J. 2005;2:69, doi: 10.1186/1743-422X-2-69.

14. Dyall J, Coleman CM, Hart BJ, et al. Repurposing of clinically developed drugs for treatment of Middle East respiratory syndrome coronavirus infection. Antimicrob Agents Chemother. 2014;58(8):4885–4893.

15. Keyaerts E, Vijgen L, Maes P, Neyts J, Van Ranst M. In vitro inhibition of severe acute respiratory syndrome coronavirus by chloroquine. Biochem Biophys Res Commun. 2004;323(1):264–268.

16. Shen L, Niu J, Wang C, et al. High-Throughput Screening and Identification of Potent Broad-Spectrum Inhibitors of Coronaviruses. J Virol. 2019;93(12):e00023-19.

17. Kono M, Tatsumi K, Imai AM, Saito K, Kuriyama T, Shirasawa H. Inhibition of human coronavirus 229E infection in human epithelial lung cells (L132) by chloroquine: involvement of p38 MAPK and ERK. Antiviral Res. 2008;77(2):150–152.

18. McChesney EW. Animal toxicity and pharmacokinetics of hydroxychloroquine sulfate. Am J Med. 1983;75(1A):11–18.

19. McChesney EW, Banks WF Jr, Fabian RJ. Tissue distribution of chloroquine, hydroxychloroquine, and

desethylchloroquine in the rat. Toxicol Appl Pharmacol. 1967;10(3):501–513.

Final Clinical Study Protocol: KY2020-29

Version date. 20 February 2020

This is the final version of the protocol in Chinese.

硫酸羟氯喹治疗新型冠状病毒肺炎的一项随机、对照、开放的多中心研究临床研究方案

（V6.0 版）

一、研究背景

2019 年 12 月以来，湖北省武汉市陆续发现了多例“不明原因肺炎”患者，后续研究在这些病例中分离到了一种冠状病毒（Coronavirus, CoV），其全基因组序列与 2003 年流行的严重急性呼吸综合征冠状病毒（Severe Acute Respiratory Syndrome Coronavirus, SARS-CoV）相似度达 79.5%，世界卫生组织将其命名为“2019-新型冠状病毒（2019-novol CoV, 2019-nCoV）”[1]。随着疫情的蔓延，我国其他地区及境外也相继发现了此类病例[2]。

有效控制病毒复制可以改善疾病进展和临床转归，但迄今为止，目前对 2019-nCoV 感染的肺炎的诊治方案中仍缺乏有效特异的抗冠状病毒药物[3]。尽早使用有潜在抗病毒效应且临床已经证明其在人体使用中有良好安全性的药物是解决这一问题的最快方案之一。

硫酸羟氯喹（hydroxychloroquine, HCQ）属 4-氨基喹啉类抗疟药，1944 年在氯喹（chloroquine, CQ）的基础上人工合成了 HCQ，两者的区别在于用羟乙基替代了 CQ 中的一个乙基，使 HCQ 在人体胃肠道吸收更快，体内分布更广。HCQ 最初用于抗疟治疗，1955 年开始用于治疗系统性红斑狼疮，1988 年开始用于治疗类风湿关节炎，其调节免疫的作用及安全性和有效性已经得到了临床的广泛认可[4]。HCQ 在保留原有 CQ 药效的同时，毒性显著降低[5,6]，具有免疫调节、抗炎、抑制细胞增殖和诱变、降低血脂以及抗血栓、降低糖尿病风险等多重作用机制 HCQ 的临床应用范围从风湿免疫性疾病延伸到皮肤、肿瘤等领域[7,8]。同时，在抗病毒方面，CQ 也有明显的治疗作用，可能通过抑制 TNFα 和 IL-6，治疗病毒感染引起的急性呼吸窘迫综合征（Acute respiratory distress syndrome, ARDS）[9]。在特殊病毒感染方面，CQ 可减轻艾滋病患者的免疫过度激活。人类免疫缺陷病毒（human immunodeficiency virus, HIV）进入表达 CD4 的浆细胞样树突状细胞（pDCs）；病毒被内吞、去壳，其 RNA 被 toll 样

受体 7（Toll-like receptor-7, TLR-7）识别；刺激 TLR-7 促进核内诱导 IFN-α 转录的信号级联；产生 IFN-α 有利于 T 细胞等多个细胞亚群的激活，B 细胞和自然杀伤淋巴细胞。氯喹通过在核内体中积累和减少 HIV 介导的 TLR-7 信号传导来降低这一机制的效率[10]。尤其对于高病毒载量的患者，800mg/d 连续服用 8 周 HCQ 可显著降低免疫激活[11,12]。在 SARS-CoV 和另一种冠状病毒，中东呼吸综合征冠状病毒病毒（Middle East Respiratory Syndrome Coronavirus, MERS-CoV）感染的相关研究中，HCQ 也显示了高剂量使用可能存在的抗病毒疗效[13,14]。有文献提到如果 SARS 再次出现，推荐 HCQ 立即用于 SARS-CoV 感染的预防和治疗[13]。小鼠研究 CQ 治疗冠状病毒感染，存活率达 80%以上[14]。而 HCQ 抗病毒的作用机制在于通过提高溶酶体等细胞器的 pH 值，干扰细胞内铁代谢，阻止病毒的内吞、脱壳。同时通过升高高尔基体的 pH 值，抑制病毒蛋白翻译后的修饰，杀死胞内的病原微生物。CQ 可以抑制 HCoV-229e 感染细胞中 p38 MAPK 的激活。从而抑制 CoV 复制[14,16,17]。在 SARS-CoV 的相关研究中，也可以通过干扰细胞受体的糖基化，阻止病毒传播[13]。其药物的安全性和组织中的分布浓度也有很好的表现[18,19]。

本研究拟在确诊的新型冠状病毒肺炎的轻型/普通型/重型患者中，探索高剂量 HCQ 对新型冠状病毒肺炎的疗效和安全性。本项目拟开展一项前瞻性、多中心、随机、对照的临床试验，评价该药物在新型冠状病毒肺炎的病毒转阴率、临床症状改善、实验室检查指标改善、预防疾病严重度增加的作用，预期可证实该药物可作为新型冠状病毒肺炎的轻型/普通型/重型患者抗病毒治疗用药。

二、研究内容和方法

（一）总体目标

本项目拟以高剂量硫酸羟氯喹片治疗轻型/普通型/重型新型冠状病毒肺炎进行临床研究；对 360 例轻型/普通型/重型新型冠状病毒肺炎患者进行治疗有效性和安全性评价的临床试验，全面评价该药物治疗在新型冠状病毒肺炎的临床作用。

（二）研究内容和方法

1.1 研究目的

评价高剂量硫酸羟氯喹片治疗轻型/普通型/重型新型冠状病毒肺炎的疗效和安全性。

1.2 试验内容

1.2.1 设计

按照 1:1 比例，共招募 360 例受试者，分为试验组 180 例，对照组 180 例，并以受试者临床分型（轻型/普通型或重型）作为随机分层因素。前瞻性、多中心、随机、开放对照研究。

1.2.2 研究人群

入组标准：

6) 18 周岁及以上；
7) 符合新型冠状病毒肺炎的诊断标准[3]，上或下呼吸道实验室检测 RT-PCR 证实 2019-nCov 核酸检测阳性，可以配合胸部 CT 影像学检查；
8) 室内空气下 $SaO_2/SpO_2 \leq 94\%$，或 $PaO_2/FiO_2 < 300mgHg$（适用于重型）；
9) 签署知情同意书；
10) 28 天内不计划参与其他研究药物的临床研究。

排除标准：

1) 年龄＜18 岁；
2) 合并其他严重内科疾病如恶性肿瘤、心肝肾疾病、不可控制的代谢性疾病等；
3) 不宜胃肠道给药者；
4) 妊娠期或哺乳期妇女；
5) 对本品成分过敏者；
6) 精神状态不能配合观察者或认知障碍者；
7) 严重肝病（如 Child Pugh C 级且 ALT>5 倍）；
8) 已知严重肾功能损害的患者（肌酐清除率≤30 mL/min）或连续肾脏替代疗法、血液透析、腹膜透析。

1.2.3 用药方法

- 试验组：按照指南推荐的标准治疗基础上，联合使用：第 1 天~第 3 天：口服硫酸羟氯喹

片（100mg/片、200mg/片），每次 400mg，每日 3 次；第 4 天~第 14/21 天：口服硫酸羟氯喹片（100mg/片、200mg/片），每次 400mg，每日 2 次。

- 对照组：按照指南推荐的标准治疗进行治疗。
- 治疗周期：轻型/普通型 14 天，重型 21 天。
- **剂量调整方案：**只有当研究者认为不良事件与研究药物有关时，才需进行剂量调整。若不良事件与研究药物无关，则不要求调整剂量。但是无论在任何情况下，研究者都要依据临床经验酌情判断。根据受试者个体的安全性和耐受性，如果研究者认为继续在给定剂量下使用研究药物可能会加重毒性，则可调整研究药物的剂量，允许暂停用药或剂量下调（仅可降低 1 个剂量水平）。受试者毒性反应恢复后，硫酸羟氯喹片的治疗可在原剂量，或低一级剂量重新开始。

	**原剂量水平**	**调整后剂量水平**
第 1~3 天	1200mg/日<br>3 次/日，400mg/次	900mg/日<br>3 次/日，300mg/次
第 4~14/21 天	800mg/日<br>2 次/日，400mg/次	600mg/日<br>2 次/日，300mg/次

若患者出现病情加重，由研究者评估患者是否从本研究药物中获益，若获益，可继续使用本研究药物至 14/21 天研究结束。

1.2.4 合并用药

治疗新型冠状病毒肺炎指南中推荐的标准治疗药物（但研究药物以外的氯喹和氯喹类药物禁用，如磷酸氯喹）均可使用。若已经使用了氯喹类药物，可停用 48 小时后参加本临床研究。受试者其他基础疾病用药可合并使用。

1.2.5 提前终止

符合以下任何一种情况的均可提前结束研究，并记录结束时间

①研究期间出现疾病恶化，经研究者综合评估后需要改变治疗；

②药物不良事件导致病情加重的出现；

③患者不愿意继续使用研究药物。

1.2.6 研究终点

主要终点：

28 天内的新型冠状病毒核酸总体检测转阴率。

对重型受试者亚组同时考虑 28 天内的临床改善率。

- 临床改善：从开始治疗到临床状态（六级分类量表）较入组时下降 2 个级别。

六级分类量表：

**级别**	**具体描述**
6	死亡
5	ICU，需要体外膜肺氧合（ECMO）和/或有创机械通气（IMV）
4	ICU/住院，需要无创机械通气（NIV）/高流量鼻导管氧疗（HFNC）
3	住院，需要氧疗（非 NIV/HFNC）
2	住院，不需要氧疗
1	出院

次要终点：

9) 4、7、10、14、21 天内的新型冠状病毒检测转阴率

10) 28 天内的临床症状改善率

- 临床症状改善：体温恢复正常（腋温≤36.6℃，或者口温≤37.2℃，或者肛温或耳温≤37.8℃），SpO_2 恢复正常（不吸氧状态下，SpO_2＞94%），呼吸道症状消失。

11) 28 天内的实验室指标改善率

- 实验室指标改善：C 反应蛋白（CRP）较基线下降≥50%，血沉较基线下降≥50%，外周血淋巴细胞绝对计数恢复正常，炎症标记物 IL-6、TNF-α 较基线下降≥50%。

12) 28 天内的影像学好转率

- 影像学好转：胸部 CT 病灶吸收较基线≥50%。

13) 全因死亡

14) 第 7、14、21、28 天的临床状态（重型）

15) 机械通气（IMV、NIV）的持续时间（天）、体外膜氧合的持续时间（天）、氧疗（鼻导管或面罩吸氧）的持续时间（天）、住院时间（天）（重型）

16) 28 天内的临床恶化率（轻型/普通型）

- 临床恶化：转为重型/危重型新型冠状病毒肺炎。

注：重型和危重型新型冠状病毒肺炎的定义[3]：

E. 重型——符合下列任何一条：

7. 呼吸窘迫，RR≥30 次/分；

8. 静息状态下，指氧饱和度≤93%；

9. 动脉血氧分压（PaO_2）/吸氧浓度（FiO_2）≤300mmHg（1mmHg=0.133kPa）。

F. 危重型——符合以下情况之一者：

7. 出现呼吸衰竭，且需要机械通气；

8. 出现休克；

9. 合并其他器官功能衰竭需 ICU 监护治疗。

安全性终点：

①不良事件发生率（鉴于硫酸羟氯喹片是临床常用的抗风湿及疟原虫药物，目前相关的不良反应已充分记载，具体如下表格）：其中，在临床常规使用剂量下，胃肠道反应（尤其是腹泻），视网膜病变相对较为常见，但停药后均可逆转。

	硫酸羟氯喹片
眼睛	长期或者高剂量服用可能会引发不可逆的视网膜损伤以及视觉障碍的风险
听力	耳鸣
肌肉骨胳系统	感觉运动障碍、骨骼肌肌病或神经肌病导致进行性弱和近端肌群萎缩、肌腱反射抑制和神经传导异常
胃肠系统	恶心、呕吐、腹泻、腹痛
皮肤和皮下组织等	皮疹、瘙痒、皮肤黏膜色素紊乱、毛色改变、脱发等
血液系统	骨髓衰竭、贫血、再生障碍性贫血、粒细胞增多、白细胞减少、血小板减少等
中枢神经系统	眩晕、眼球震颤、神经性失语、头痛、头晕、癫痫、共济失调和锥体外系疾病
精神类副作用	紧张、易怒、噩梦、精神病、自杀行为
心血管系统	心肌病并可能导致心力衰竭、QT 间期延长
肝脏	肝功能指标异常
免疫系统	荨麻疹、血管水肿、支气管痉挛
新陈代谢	食欲下降、低血糖、卟啉症、体重下降

②退出研究的比例

1.2.7 统计学处理

样本量估计：

本研究试验组与对照组随机分配比例为 1:1，假设病毒核酸转阴时间服从以转阴速度为参数的指数分布。假设对照组的中位转阴时间为 10 天，治疗组相对于对照组病毒核酸转阴的速度比为 1.43，即假设治疗组的中位转阴时间为 7 天，采用成组序贯设计，在大约 40%的人群入组时（至多 40%事件信息量）进行一次期中有效性分析，使用 Lan-Demets 算法的 O'Brien-Fleming 类型的 α-消耗函数以控制总体一类错误率 α=0.025（单侧），80%的把握度，用 Log-rank test 进行检验，至少需要 248 个转阴事件。假设 75 天完成入组，计划在最后一例受试者用药 7 天后进行最终分析，考虑 20%的脱落率，本试验需 360 例受试者。

本研究在重型受试者亚组中同时分析 28 天临床改善率。假设对照组临床改善率为 50%，100 例重型受试者可以至少有 70%的把握度检测出治疗组 25%的临床改善率的提高。

分析集：

- 意向性治疗集（ITT）：定义为所有随机进入试验的受试者。ITT 人群将作为本研究疗效分析的主要分析人群。受试者将基于其计划分配的治疗组来进行分析。
- 符合方案集（PPS）：符合方案集为 ITT 的子集，指所有随机入组接受过至少 11 天（轻型/普通型）/16 天（重型）的治疗，且无明显影响主要疗效的重大方案偏离的受试者，构成符合方案分析集。基于 PPS 的分析将作为支持性分析对基于 ITT 的分析进行补充。受试者将基于其计划分配的治疗组来进行分析。
- 安全性数据集（SS）：定义为所有接受了至少一次试验药物的受试者。安全性分析人群是安全性指标的主要分析人群。受试者将根据其实际分配的治疗组进行分析。

疗效分析：

- 主要疗效分析：

用 Kaplan-Meier 法分析整体转阴率。绘制随时间变化的转阴率的 KM 曲线，用分层 Log-Rank Test 比较两组曲线，以评估两组整体疗效。分层因素与随机时使用的分层因素相同。

在重型人群中进行亚组分析，考虑主要终点临床改善率的变化。记录各时间点的改善率。用卡方检验对两组中的 28 天临床改善率的差别进行统计检验。作为敏感性分析，也将用 KM 方法进行估算。

- 次要疗效分析：

计算和报告各组中其他各时间点的病毒核酸转阴率、临床症状改善率、实验室指标改善率、影像学好转率、全因死亡、临床恶化率等比例型终点的受试者比例，以及风险比（RR）及其 95%置信区间。采用 Kaplan-Meier 法分析机械通气/体外膜氧合/氧疗/住院时间和其他 time-to-event 类型的终点。报告中位时间及其 95%置信区间。KM 曲线的比较由 Log-Rank 检验进行。

安全性分析：

不良事件将按照编码后的系统器官分类（SOC）和首选术语（PT）分别归纳例数、发生率以及例次。汇总各组 AE、TEAE、SAE 以及 ADR 例数、发生率以及例次。

将汇总特定实验室检查结果、心电图检查、生命体征及体格检查等安全指标的检查值和其相对基线的变化。在适用的情况下，基线检查值与基线后各时间点检查值用交叉表形式呈现。

期中分析：

本研究将设立独立数据监查委员会（IDMC）进行期中分析。IDMC 的人员组成、任务责任及实施细节将在 IDMC 章程中规定。

本次研究一共安排两次分析：一次期中分析，一次最终分析。在 Group Sequential Design 中采用 Lan-Demets 算法，使用 O’Brien-Fleming 类型的 α-消耗函数（累积）以控制总体I类错误率 0.05。

第一次期中分析在 40%（大约 150 人）的受试者入组 7 天后进行，主要目的是进行样本量重估和优效性评估，显著性水平（p-value boundary）0.00079（双侧）。

最终分析在达到 248 个转阴事件后进行，显著性水平（p-value boundary）为 0.04974（双侧）。

每次分析的实际 α 消耗和显著性水平将根据分析时实际观察到的确切事件数进行调整。

1.2.8 研究流程图

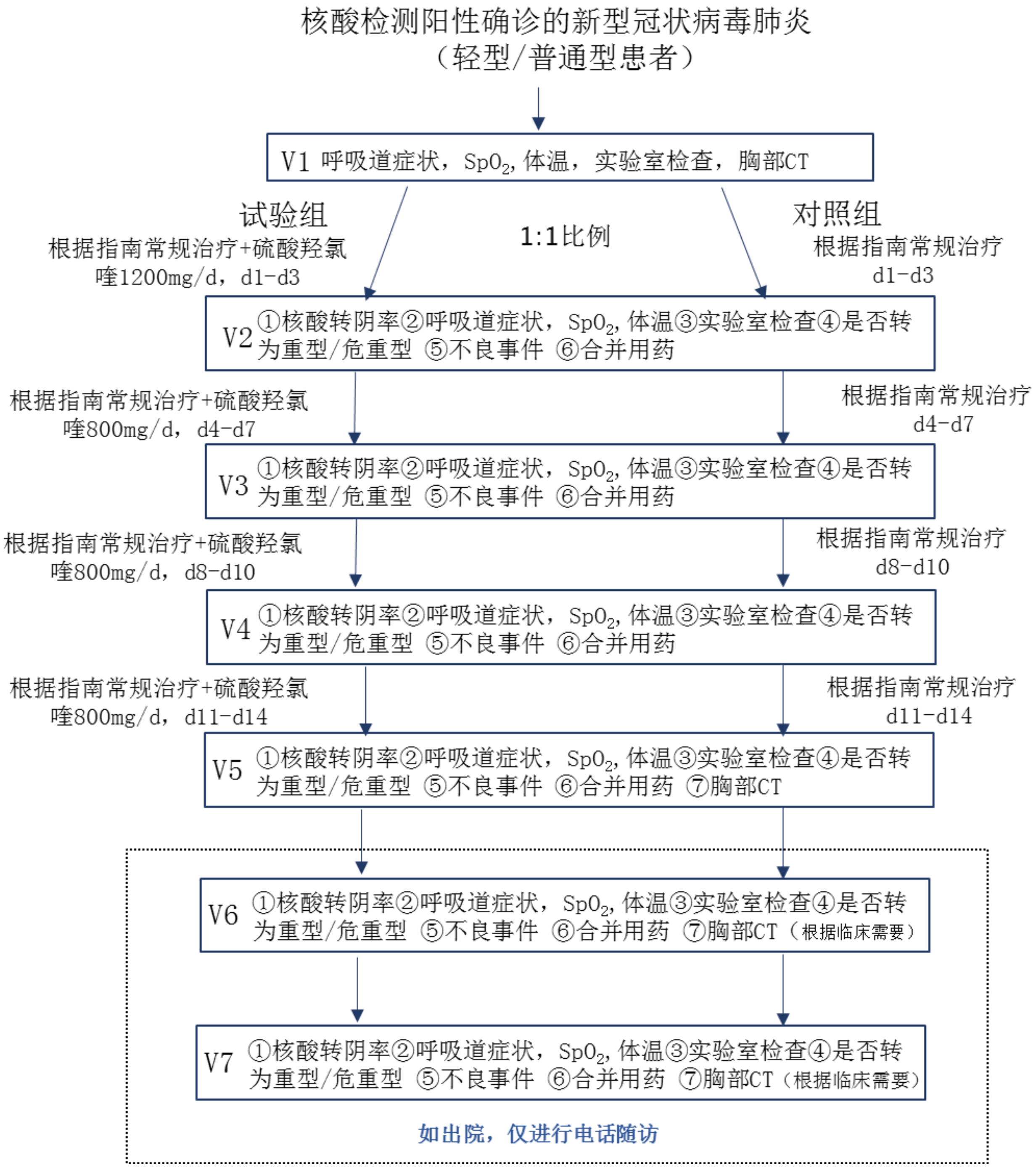

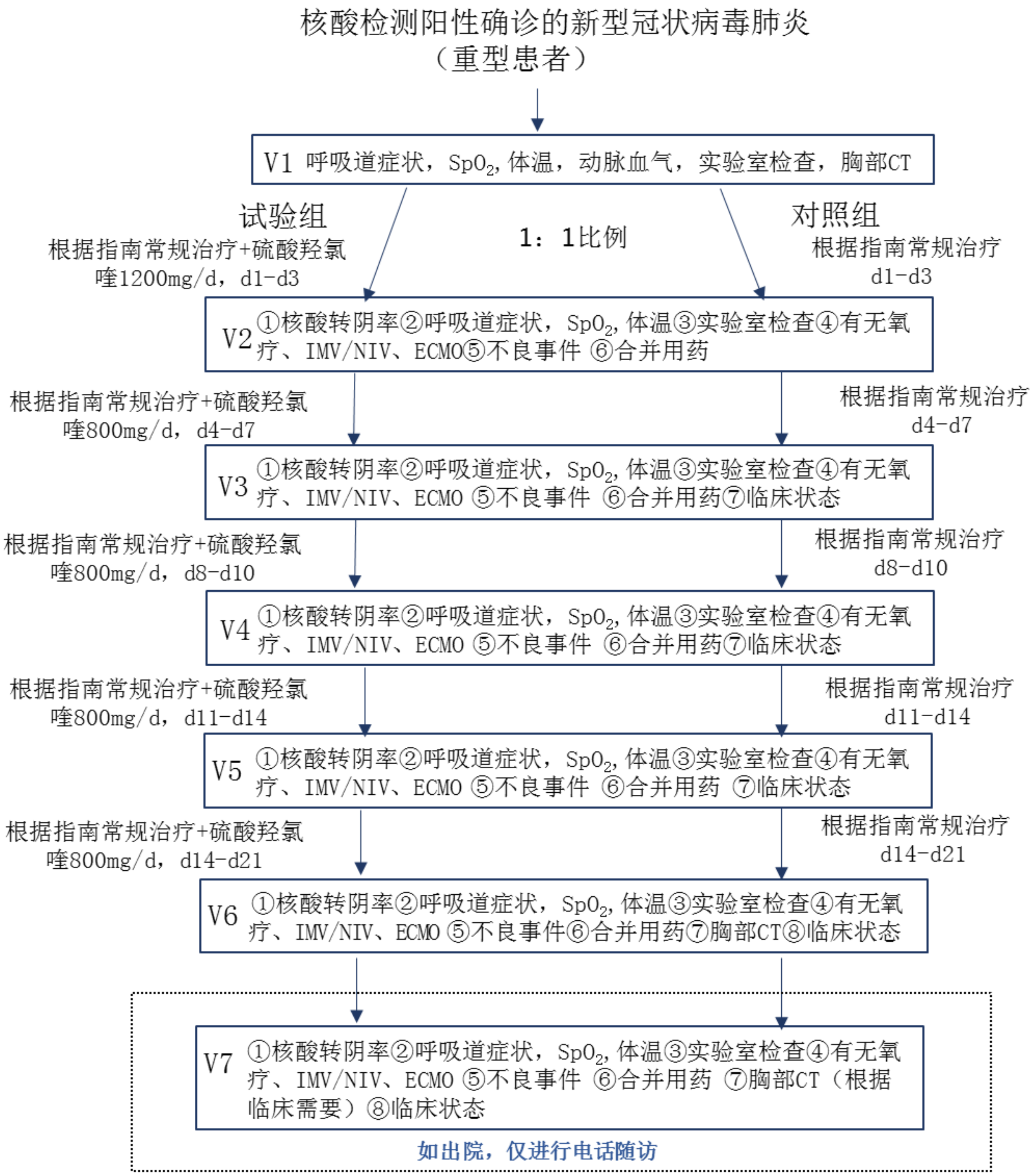
核酸检测阳性确诊的新型冠状病毒肺炎
（重型患者）
V1 呼吸道症状，SpO_2, 体温，动脉血气，实验室检查，胸部CT
试验组
根据指南常规治疗+硫酸羟氯喹1200mg/d，d1-d3
1：1比例
对照组
根据指南常规治疗 d1-d3
V2 ①核酸转阴率②呼吸道症状，SpO_2, 体温③实验室检查④有无氧疗、IMV/NIV、ECMO⑤不良事件 ⑥合并用药
根据指南常规治疗+硫酸羟氯喹800mg/d，d4-d7
根据指南常规治疗 d4-d7
V3 ①核酸转阴率②呼吸道症状，SpO_2, 体温③实验室检查④有无氧疗、IMV/NIV、ECMO ⑤不良事件 ⑥合并用药⑦临床状态
根据指南常规治疗+硫酸羟氯喹800mg/d，d8-d10
根据指南常规治疗 d8-d10
V4 ①核酸转阴率②呼吸道症状，SpO_2, 体温③实验室检查④有无氧疗、IMV/NIV、ECMO ⑤不良事件 ⑥合并用药⑦临床状态
根据指南常规治疗+硫酸羟氯喹800mg/d，d11-d14
根据指南常规治疗 d11-d14
V5 ①核酸转阴率②呼吸道症状，SpO_2, 体温③实验室检查④有无氧疗、IMV/NIV、ECMO ⑤不良事件 ⑥合并用药 ⑦临床状态
根据指南常规治疗+硫酸羟氯喹800mg/d，d14-d21
根据指南常规治疗 d14-d21
V6 ①核酸转阴率②呼吸道症状，SpO_2, 体温③实验室检查④有无氧疗、IMV/NIV、ECMO ⑤不良事件⑥合并用药⑦胸部CT⑧临床状态
V7 ①核酸转阴率②呼吸道症状，SpO_2, 体温③实验室检查④有无氧疗、IMV/NIV、ECMO ⑤不良事件 ⑥合并用药 ⑦胸部CT（根据临床需要）⑧临床状态
如出院，仅进行电话随访

1.3 临床研究流程图

试验流程图（轻型/普通型）

**研究流程**	**筛选期/治疗期**	**治疗期（2-14 天）**				**延伸随访期**[13]	
访视名称	V1	V2	V3	V4	V5	V6	V7
访视时间	D-3~1	D4±1d	D7±1d	D10±1d	D14±1d	D21±2d	D28±2d
知情同意	×						
确认入选/排除标准	×						
受试者随机化	×						
收集人口学资料[1]	×						
收集病史和伴随疾病治疗[2]	×						
体格检查[3]	×				×	×	×
生命体征[4]	×	×	×	×	×	×	×
12-导联心电图	×[11]				×	×	×
CRP，血沉	×[11]	×	×	×	×	×	×
TNF-α，IL-6	×[11]	×	×	×	×	×	×
血常规[5]	×[11]	×	×	×	×	×	×
尿常规[6]	×[11]				×	×	×
血生化[7]<br>（肝功能、肾功能、心肌酶）	×[11]	×	×	×	×	×	×
凝血功能[8]	×[11]	×	×	×	×	×	×
新型冠状病毒核酸检测[9]	×[12]	×	×	×	×	×	×
指脉氧饱和度(不吸氧情况下)	×	×	×	×	×	×	×
尿或血清 HCG<br>（仅适用于育龄女性）	×[11]				×		
胸部 CT	×[11]				×	根据临床需要进行	
有无呼吸道症状	×	×	×	×	×	×	×
试验用药情况[10]	×	×	×	×	×		
是否转为重型/危重型肺炎		×	×	×	×	×	×
合并用药	×	×	×	×	×	×	×
不良事件	×	×	×	×	×	×	×

14. 人口学资料记录包括出生日期、性别、民族；
15. 包括现病史和既往史，以及伴随疾病治疗；
16. 体格检查包括整体外观、头、眼、耳、鼻、喉、颈部及甲状腺、皮肤、心血管系统、胸部及肺、腹部、神经系统、骨骼肌系统及淋巴系统；

17. 生命体征：体温，呼吸频率，脉搏，血压；体温测量记录日最高体温，试验期间体温测量方法必须与基线保持一致；
18. 血常规：红细胞计数（RBC）、血红蛋白浓度（HGB）、白细胞计数（WBC）、血小板计数（PLT）、中性粒细胞计数（ANC）、淋巴细胞计数（LY）、单核细胞计数（MO）、嗜酸性粒细胞计数（EO）、嗜碱性粒细胞计数（BA）；
19. 尿常规：尿 pH 值、尿红细胞、尿白细胞、尿蛋白、尿葡萄糖、尿酮体；
20. 血生化：谷丙转氨酶（ALT）、谷草转氨酶（AST）、总胆红素（TBIL）、白蛋白（ALB）、γ-谷氨酰转肽酶（GGT）、乳酸脱氢酶（LDH）、肌酸激酶（CK）、肌酸激酶同工酶（CK-MB）、血尿素氮（BUN）、血肌酐（CR）；
21. 凝血功能：凝血酶原时间（PT）、活化部分凝血酶时间（APTT）、凝血酶时间（TT）血浆纤维蛋白原（Fbg）、国际标准化比率（INR）；
22. 研究者如果进行计划外访视的病毒核酸检测，数据结果需记录在 CRF 中；
23. 每天记录受试者用药情况，是否漏服、多服；
24. 如果受试者首次用药前 3 天内有符合要求的检查结果，获得研究者认可后，则该受试者筛选期不需要再进行检查。
25. 如果受试者有报告日期在首次用药前 5 天内的核酸检查结果，获得研究者认可后，则该受试者筛选期不需要再进行检查。
26. 对于已出院的受试者进行电话随访，仅收集合并用药、不良事件。

试验流程图（重型）

研究流程	筛选期/治疗期	治疗期（2-21 天）					延伸随访期[13]
访视名称	V1	V2	V3	V4	V5	V6	V7
访视时间	D-3~1	D4±1d	D7±1d	D10±1d	D14±1d	D21±2d	D28±2d
知情同意	×						
确认入选/排除标准	×						
受试者随机化	×						
收集人口学资料[1]	×						
收集病史和伴随疾病治疗[2]	×						
体格检查[3]	×					×	×
生命体征[4]	×	×	×	×	×	×	×
12-导联心电图	×[11]					×	×
CRP，血沉	×[11]	×	×	×	×	×	×
TNF-α，IL-6	×[11]	×	×	×	×	×	×
血常规[5]	×[11]	×	×	×	×	×	×
尿常规[6]	×[11]					×	×
血生化[7]	×[11]	×	×	×	×	×	×

（肝功能、肾功能、心肌酶）							
凝血功能[8]	×[11]	×	×	×	×	×	×
新型冠状病毒核酸检测[9]	×[12]	×	×	×	×	×	×
指脉氧饱和度（不吸氧情况下）	×	×	×	×	×	×	×
动脉血气分析	×[11]	根据临床需要进行					
尿或血清 HCG<br>（仅适用于育龄女性）	×[11]					×	
胸部 CT	×[11]					×	根据临床需要进行
有无呼吸道症状	×	×	×	×	×	×	×
试验用药情况[10]	×	×	×	×	×	×	
临床状态	×		×		×	×	×
有无 IMV/NIV、ECMO、氧疗	×	×	×	×	×	×	×
合并用药	×	×	×	×	×	×	×
不良事件	×	×	×	×	×	×	×

1. 人口学资料记录包括出生日期、性别、民族；
2. 包括现病史和既往史，以及伴随疾病治疗；
3. 体格检查包括整体外观、头、眼、耳、鼻、喉、颈部及甲状腺、皮肤、心血管系统、胸部及肺、腹部、神经系统、骨骼肌系统及淋巴系统；
4. 生命体征：体温，呼吸频率，脉搏，血压；体温测量记录日最高体温，试验期间体温测量方法必须与基线保持一致；
5. 血常规：红细胞计数（RBC）、血红蛋白浓度（HGB）、白细胞计数（WBC）、血小板计数（PLT）、中性粒细胞计数（ANC）、淋巴细胞计数（LY）、单核细胞计数（MO）、嗜酸性粒细胞计数（EO）、嗜碱性粒细胞计数（BA）；
6. 尿常规：尿 pH 值、尿红细胞、尿白细胞、尿蛋白、尿葡萄糖、尿酮体；
7. 血生化：谷丙转氨酶（ALT）、谷草转氨酶（AST）、总胆红素（TBIL）、白蛋白（ALB）、γ-谷氨酰转肽酶（GGT）、乳酸脱氢酶（LDH）、肌酸激酶（CK）、肌酸激酶同工酶（CK-MB）、血尿素氮（BUN）、血肌酐（CR）；
8. 凝血功能：凝血酶原时间（PT）、活化部分凝血酶时间（APTT）、凝血酶时间（TT）血浆纤维蛋白原（Fbg）、国际标准化比率（INR）；
9. 研究者如果进行计划外访视的病毒核酸检测，数据结果需记录在 CRF 中；
10. 每天记录受试者用药情况，是否漏服、多服；
11. 如果受试者首次用药前 3 天内有符合要求的检查结果，获得研究者认可后，则该受试者筛选期不需要再进行检查。
12. 如果受试者有报告日期在首次用药前 5 天内的核酸检查结果，获得研究者认可后，则该受试者筛选期不需要再进行检查。

13. 对于已出院的受试者进行电话随访，仅收集合并用药、不良事件。

三、受试者的风险与获益风险

本项目是一项随机对照开放性研究，硫酸羟氯喹片作为已经上市数年的免疫抑制剂和抗病原微生物药物，安全性和疗效已经得到了一定的认可，预计不会给受试者带来额外的风险。

可能的获益：

1. 参与本研究的受试者可能从治疗组获益，对照组采样根据指南的常规治疗，故均可获得现有阶段最佳的治疗疗效。
2. 在参加研究期间，受试者相关检查费用和研究药物的费用将由申办方承担。

保密：

受试者的个人信息记录将被保密，根据法律和法规，将不会被公开。如果研究结果发表，受试者的个人身份仍将保密。

参考文献：

1. Zhou P, Yang XL, Wang XG, et al. A pneumonia outbreak associated with a new coronavirus of probable bat origin [published online ahead of print, 2020 Feb 3]. Nature. 2020; doi:10.1038/s41586-020-2012-7

2. Wang C, Horby PW, Hayden FG, Gao GF. A novel coronavirus outbreak of global health concern [published online ahead of print, 2020 Jan 24]. Lancet. 2020; doi:10.1016/S0140-6736(20)30185-9.

3.国家卫生健康委员会. 新型冠状病毒感染的肺炎诊疗方案（试行第六版）, http://www.gov.cn/zhengce/zhengceku/2020-02/05/content_5474791.html, 2020.02.05 发行

4. Leden I. Antimalarial drugs-350 years. Scand J Rheumatol. 1981;10(4):307–312.

5. Rainsford KD, Parke AL, Clifford-Rashotte M, et al. Therapy and pharmacological properties of hydroxychloroquine and chloroquine in treatment of systemic lupus erythematosus, rheumatoid arthritis and related diseases. Inflammopharmacology. 2015;23(5):231–269

6. 赵义,李小霞，用辩证的眼光看待硫酸羟氯喹的用药安全，药物不良反应杂志 2015;17(3): 164-166.

7. Ben-Zvi I, Kivity S, Langevitz P, Shoenfeld Y. Hydroxychloroquine: from malaria to autoimmunity. Clin Rev

Allergy Immunol. 2012;42(2):145–153.

8. Olsen NJ, Schleich MA, Karp DR. Multifaceted effects of hydroxychloroquine in human disease. Semin Arthritis Rheum. 2013;43(2):264–272.

9. Savarino A, Boelaert JR, Cassone A, Majori G, Cauda R. Effects of chloroquine on viral infections: an old drug against today's diseases?. Lancet Infect Dis. 2003;3(11):722–727.

10. Savarino A, Gennero L, Sperber K, Boelaert JR. The anti-HIV-1 activity of chloroquine. J Clin Virol. 2001;20(3):131–135.

11. Savarino A, Shytaj IL. Chloroquine and beyond: exploring anti-rheumatic drugs to reduce immune hyperactivation in HIV/AIDS. Retrovirology. 2015;12:51, doi: 10.1186/s12977-015-0178-0.

12. Akpovwa H. Chloroquine could be used for the treatment of filoviral infections and other viral infections that emerge or emerged from viruses requiring an acidic pH for infectivity. Cell Biochem Funct. 2016;34(4):191–196.

13. Vincent MJ, Bergeron E, Benjannet S, et al. Chloroquine is a potent inhibitor of SARS coronavirus infection and spread. Virol J. 2005;2:69, doi: 10.1186/1743-422X-2-69.

14. Dyall J, Coleman CM, Hart BJ, et al. Repurposing of clinically developed drugs for treatment of Middle East respiratory syndrome coronavirus infection. Antimicrob Agents Chemother. 2014;58(8):4885–4893.

15. Keyaerts E, Vijgen L, Maes P, Neyts J, Van Ranst M. In vitro inhibition of severe acute respiratory syndrome coronavirus by chloroquine. Biochem Biophys Res Commun. 2004;323(1):264–268.

16. Shen L, Niu J, Wang C, et al. High-Throughput Screening and Identification of Potent Broad-Spectrum Inhibitors of Coronaviruses. J Virol. 2019;93(12):e00023-19.

17. Kono M, Tatsumi K, Imai AM, Saito K, Kuriyama T, Shirasawa H. Inhibition of human coronavirus 229E infection in human epithelial lung cells (L132) by chloroquine: involvement of p38 MAPK and ERK. Antiviral Res. 2008;77(2):150–152.

18. McChesney EW. Animal toxicity and pharmacokinetics of hydroxychloroquine sulfate. Am J Med. 1983;75(1A):11–18.

19. McChesney EW, Banks WF Jr, Fabian RJ. Tissue distribution of chloroquine, hydroxychloroquine, and

desethylchloroquine in the rat. Toxicol Appl Pharmacol. 1967;10(3):501–513.

Summary of Protocol changes from: KY2020-29

This is the translated version of the final protocol originally written in Chinese.

Evaluation of the Efficacy and Safety of Hydroxychloroquine Sulfate in Adult Patients With 2019-nCoV Respiratory Infection: A Multicenter Randomized Study

SUMMARY OF CHANGES HISTORY

Protocol Version: 1.0-6.0

Key Changes:

1. The name of the study drug hydroxychloroquine in the original version change to hydroxychloroquine sulfate in the newest version.

2. Introduction was modified based on updating knowledge on 2019-nCoV and Hydroxychloroquine

3. The sample size of 200 cases in the original version was replaced by 360 cases in the newest version based on the alternative hypothesis of a 30% reduction of time to virus nucleic acid negativity. Under the exponential distribution, this benefit translates to a reduction in median time to virology negativity from 10 days to 7 days. A total of 248 events would provide a power of 80% to detect a 30% reduction of hazard ratio (control vs. treatment) of 0.70 with a Log-Rank test, assuming a two-sided significance level of 5%. A 75-day accrual period and an additional 7-day follow-up are assumed. The sample size calculation has also taken into consideration an annual dropout rate of 20%.

4. In the original version of the protocol, patients with severe pneumonia were excluded. However, considering the anti-inflammatory property of HCQ favoring disease regression, we decided to change exclusion criteria in order to include patients with severe type of pneumonia. Stratified random sampling was then applied to stratify all eligible patients according to the disease severity (mild-to-moderate or severe) followed by random assignment (in a 1:1 ratio) in each stratum to ensure balanced distribution of disease severity between treatment (HCQ plus SOC) and control (SOC only) groups.

5. In the original version of the protocol, age of patients was restricted to 14 or higher. However, given no data available of COVID-19 in adolescents, we decided to focus on adult patients without risking younger patients and lifted the threshold of age limitation to 18 years.

6. Patients with severe liver disease and renal diseases were excluded due to safety concerns of using hydroxychloroquine in this population.

7. In the 4th version, “Onset of illness ≤12 days” was added to focus on as earlier stage of COVID-19 as possible. However, in hospitalized patients, this criterion would rule out too many patients that could previously participate in. We therefore remove this criterion in last version.

8. In the original version, the study drug was orally administrated 2 tablets/time of hydroxychloroquine (250mg/ tablet), twice a day, from Day1-Day3 followed by 1 tablet/time of hydroxychloroquine (250mg/ tablet) , twice a day, from Day4-Day10. In the newest version, the study drug changed to orally administrated hydroxychloroquine sulfate tablets (100mg/ tablet) 4 tablets /time, three times a day, from day 1 to day 3 followed by hydroxychloroquine sulfate (100mg/ tablet), 4 tablets/time, twice a day, from Day 4 ~ day 14(for mild-to-moderate patients), from Day 4 ~ day 21(for severe patients).

9. Concomitant anti-viral treatments were forbidden after randomization in the original version. However, considering the fact that these drugs are recommended as standard-of-care in the national guideline, we decided to remove this restriction in the later version. Moreover, usage of chloroquine was prohibited after randomization. If the subject had already used chloroquine medication prior to randomization, he or she can participate in this clinical study after 48 hours of chloroquine medication.

10. In the newest version, addition to the secondary efficacy endpoint: time to negative of the novel coronavirus nucleic acid detection (day) and rate of negative percentage of the novel coronavirus detection of the treatment day 4, day 7, day 14 & day 21.

11. In the original version, the clinical symptom improvement as one of the secondary study endpoints was described as respiratory symptom reduction ⩾50%. In the newest version, this was described as respiratory symptom disappearance.

12. Add six-level classification scale for evaluation the clinical severity of subjects with severe type of 2019-nCoV pneumonia: Level specific description are as follows:

Score 6: death; Score 5: ICU administration and/or requiring extracorporeal membrane pulmonary oxygenation (ECMO) and/or invasive mechanical ventilation (IMV); Score 4: ICU/ hospitalization requiring non-invasive mechanical ventilation (NIV)/high-flow nasal catheter oxygen therapy (HFNC); Score 3: Hospitalization, requiring oxygen therapy (non-niv /HFNC); Score 2: Hospitalization, no need of oxygen therapy; Score1:discharge from the hospital.

13. Add details to safety end point in the newest version: Gastrointestinal reactions (especially diarrhea) and retinopathy are relatively common at clinically prescribed doses, but can be reversed after withdrawal. Long-term or high doses may cause irreversible retinal damage and the risk of visual impairment.

14. Add secondary endpoint for subject with severe type of 2019-nCoV pneumonia: All-cause mortality.

15. Add secondary endpoint for subject with severe type of 2019-nCoV pneumonia: clinical status on days 7, 14, 21 and 28.

16. Add secondary endpoint for subject with severe type of 2019-nCoV pneumonia: duration of mechanical ventilation (IMV, NIV) (days), extracorporeal membrane oxygenation (days), oxygen therapy (nasal catheter or mask oxygen) (days), hospitalization (days).

Original Statistical Analysis Plan: KY2020-29

Version date. 20 February 2020

No amendment of this statistical analysis plan until the study completion.

Evaluation of the Efficacy and Safety of Hydroxychloroquine sulfate in Adult Patients With 2019-nCoV Respiratory Infection: A Multicenter, Randomized, Controlled Study

Protocol Number: KY2020-29

Statistical Analysis Plan

Version：1.0

Date：2020.02.20

Content

1 PRIMARY OBJECTIVE 4

2 STUDY DESIGN 4

2.1 DESIGN 4

2.2 RANDOMIZATION 4

2.3 VISIT SCHEDULES 5

3 ENDPOINTS 9

3.1 PRIMARY ENDPOINTS 9

3.2 SECONDARY ENDPOINT 9

3.3 SAFETY ENDPOINTS 10

4 SAMPLE SIZE JUSTIFICATION 11

5 ANALYSIS SET 11

6 STATISTICAL ANALYSIS PLAN 11

6.1 GENERAL ANALYSIS METHOD 11

6.2 IMPUTATION OF MISSING DATA 12

6.3 THE DEFINITION OF BASELINE 14

6.4 DISPOSITION 14

6.4.1 Disposition Information 14

6.5 PROTOCOL DEVIATIONS 14

6.6 SUBJECT INFORMATION 15

6.6.1 Demographics and Baseline Characteristics 15

6.6.2 Disease Characteristics 15

6.6.3 Medical History 15

6.6.4 Medical Allergy History 15

6.6.5 Historical/Concomitant Medications 15

6.6.6 Historical/Concomitant Treatment(non-Medications) 16

7 EFFICACY 16

7.1.1 Primary Efficacy Analysis 16

7.1.2 Secondary Efficacy Analysis 17

8 SAFETY 18

8.1.1 Drug Exposure 18

8.1.2 Adverse Event 18

8.1.3 Clinical Laboratory Tests 19

8.1.4 Electrocardiogram (ECG) 20

8.1.5 Vital Signs 20

8.1.6 Physical Examination Findings 20

9 INTERIM ANALYSIS 20

10 TFL SHELLS 21

11 REFERENCES 21

ABBREVIATIONS	
ADR	Adverse Drug Reaction
AE	adverse event
CI	Confidence Interval
CM	Concomitant Medication
CRF	Case Report Form
DBL	Database Lock
ITT	Intended-to-treat
MedDRA	Medical Dictionary for Regulatory Activities
PPS	Per Protocol
PT	Preferred Term
SAE	Serious adverse event
SAP	Statistical Analysis Plan
SOC	System Organ Class
SS	Safety Set
TEAE	Treatment-emergent Adverse Event
WHO	World Health Organization

1 Primary objective

The primary objective of this study is to evaluate the efficacy and safety of high dose of HCQ sulfate in patients with 2019-nCoV respiratory infection by comparisons between the HCQ group and the control group.

2 Study Design

2.1 Design

This trial was a prospective, multicenter, open-label, randomized trial of patients with 2019-nCoV. The trial enrolled 360 patients with 1:1 randomization ratio stratified by disease severity (mild/moderate/severe) to receive either standard-of-care (SOC) or SOC plus HCQ.

2.2 Randomization

The eligible patients will be randomly assigned to one of the treatment arms with ratio 1:1. The randomization number will be generated by computer and implemented by random envelops. The stratification factor includes severity of disease (mild/moderate and severe).

2.3 Visit Schedules

Table 2-1 Time and Events Schedule (mild/moderate subgroup)

**Evaluation / Procedure**	**Screen/ Treatment**	**Treatment and follow-up (Day 2-14)**				**Extended follow-up[13]**	
Visit	V1	V2	V3	V4	V5	V6	V7
Day +/- Window	D-3~1	D4±1d	D7±1d	D10±1d	D14±1d	D21±2d	D28±2d
Informed Consent	×						
I/E Criteria	×						
Randomization	×						
Demographics[1]	×						
Epidemiological and medical history[2]	×						
Physical examination[3]	×				×	×	×
Vital signs[4]	×	×	×	×	×	×	×
12-lead electrocardiogram	×[11]				×	×	×
CRP, ESR	×[11]	×	×	×	×	×	×
TNF-α，IL-6	×[11]	×	×	×	×	×	×
CBC with differential[5]	×[11]	×	×	×	×	×	×
Urinalysis[6]	×[11]				×	×	×
Blood chemistry[7] (hepatic and kidney panel, Myocardial enzyme)	×[11]	×	×	×	×	×	×
Coagulation panel[8]	×[11]	×	×	×	×	×	×
2019-nCoV RT-PCR[9]	×[12]	×	×	×	×	×	×
Pulse oximetry （on room air）	×	×	×	×	×	×	×
Pregnancy Test (Serum or Urine hCG)	×[11]				×		
Chest CT	×[11]				×	Optional (*as per* clinical needs)	
Respiratory symptom (Y/N)	×	×	×	×	×	×	×
Study drug administration [10]	×	×	×	×	×		
Progression to severe/critically severe type (Y/N)		×	×	×	×	×	×
Record of SMT	×	×	×	×	×	×	×
AE Assessment	×	×	×	×	×	×	×
27. Demographics include birth date, gender and ethnicity.							

28. Epidemiological information includes recent travel history and exposure to epidemic area or infected patients; medical history includes present illness and past medical history including co-morbidity medications
29. Physical examination includes height, weight, general outlook, head, eye, ear, nose, throat, neck and thyroid, skin, cardiovascular system, Anterior chest and lung, abdomen, neuro system, skeletal muscle system and lymphatic system
30. Vital signs: body temperature, respiratory rate, pulse, blood pressure; record maximum temperature of the visiting day and use same measurement criteria throughout the study period.
31. CBC with differential includes: haptoglobin and absolute count of red blood cell, platelet, total white blood cell, neutrophil, lymphocyte, monocyte, eosinophil and basophil
32. Urinalysis: urinary pH, urinary red blood cell, urinary white blood cell, urinary protein, glucose and ketone
33. Blood chemistry includes alanine aminotransferase, aspartate aminotransferase, total bilirubin, albumin, γ-glutamyl transpeptidase, lactate dehydrogenase, creatine kinase, creatine kinase isoenzyme-MB, blood urea nitrogen and creatinine
34. Coagulation panel includes: Prothrombin time, activated partial thrombin time, thrombin time, fibrinogen and international normalized ratio.
35. RT-PCR results upon unplanned visit should also be recorded in CRF
36. Record daily administration of tested drug including under use and over use
37. These examinations can be exempted by the investigators if the participants can provide qualified examination results within 3-day prior to initiation of the study
38. RT-PCR results of 2019-nCoV within 5-day prior to initiation of the study can be used for screening
39. For patients discharge alive, only medications and AE information will be collected *via* telephone contact.

Table 2-2 Time and Events Schedule (severe subgroup)

**Evaluation / Procedure**	**Screen/ Treatment**	**Treatment and follow-up (Day 2-14)**					**Extended follow-up**[13]
Visit	V1	V2	V3	V4	V5	V6	V7
Day +/- Window	D-3~1	D4±1d	D7±1d	D10±1d	D14±1d	D21±2d	D28±2d
Informed Consent	×						
I/E Criteria	×						
Randomization	×						
Demographics[1]	×						
Epidemiological and medical history[2]	×						
Physical examination[3]	×					×	×
Vital signs[4]	×	×	×	×	×	×	×
12-lead	×[11]					×	×

electrocardiogram							
CRP, ESR	×[11]	×	×	×	×	×	×
TNF-α，IL-6	×[11]	×	×	×	×	×	×
CBC with differential[5]	×[11]	×	×	×	×	×	×
Urinalysis[6]	×[11]					×	×
Blood chemistry[7] (hepatic and kidney panel, Myocardial enzyme)	×[11]	×	×	×	×	×	×
Coagulation panel[8]	×[11]	×	×	×	×	×	×
2019-nCoV RT-PCR[9]	×[12]	×	×	×	×	×	×
Pulse oximetry（on room air）	×	×	×	×	×	×	×
Arterial blood gas analysis	×[11]	Optional (*as per* clinical needs)					
Pregnancy Test (Serum or Urine hCG)	×[11]					×	
Chest CT	×[11]					×	Optional (*as per* clinical needs)
Respiratory symptom (Y/N)	×	×	×	×	×	×	×
Study drug administration [10]	×	×	×	×	×	×	
Clinical status	×		×		×	×	×
IMV/NIV, ECMO or supplemental oxygen (Y/N)	×	×	×	×	×	×	×
Record of SMT	×	×	×	×	×	×	×
AE Assessment	×	×	×	×	×	×	×

13. Demographics include birth date, gender and ethnicity.
14. Epidemiological information includes recent travel history and exposure to epidemic area or infected patients; medical history includes present illness and past medical history including co-morbidity medications
15. Physical examination includes height, weight, general outlook, head, eye, ear, nose, throat, neck and thyroid, skin, cardiovascular system, Anterior chest and lung, abdomen, neuro system, skeletal muscle system and lymphatic system
16. Vital signs: body temperature, respiratory rate, pulse, blood pressure; record maximum temperature of the visiting day and use same measurement criteria throughout the study period.
17. CBC with differential includes: haptoglobin and absolute count of red blood cell, platelet,

total white blood cell, neutrophil, lymphocyte, monocyte, eosinophil and basophil

18. Urinalysis: urinary pH, urinary red blood cell, urinary white blood cell, urinary protein, glucose and ketone
19. Blood chemistry includes alanine aminotransferase, aspartate aminotransferase, total bilirubin, albumin, γ-glutamyl transpeptidase, lactate dehydrogenase, creatine kinase, creatine kinase isoenzyme-MB, blood urea nitrogen and creatinine
20. Coagulation panel includes: Prothrombin time, activated partial thrombin time, thrombin time, fibrinogen and international normalized ratio.
21. RT-PCR results upon unplanned visit should also be recorded in CRF
22. Record daily administration of tested drug including under use and over use
23. These examinations can be exempted by the investigators if the participants can provide qualified examination results within 3-day prior to initiation of the study
24. RT-PCR results of 2019-nCoV within 5-day prior to initiation of the study can be used for screening

For patients discharge alive, only medications and AE information will be collected *via* telephone contact.

3 Endpoints

3.1 Primary Endpoints

(1) Negative conversion rate of virus nucleic acid within 28-day

(2) For severe disease subgroup, considering both virology conversion and clinical improvement.

- Definition for clinical improvement： decline of two categories from enrollment status on a six-category ordinal scale of clinical status which ranging from 1 (discharged) to 6 (death).

Table 3-1 Six-category ordinal scale

Scale	Description
6	Death
5	ICU, requiring ECMO and/or IMV
4	ICU/hospitalization, requiring NIV/ HFNC therapy
3	Hospitalization, requiring supplemental oxygen (but not NIV/ HFNC)
2	Hospitalization, not requiring supplemental oxygen;
1	Discharge

Abbreviation: IMV, invasive mechanical ventilation; NIV, non-invasive mechanical ventilation; HFNC, High-flow nasal cannula.

3.2 Secondary endpoint

17) Negative conversion rate of virus nucleic acid at day 4, 7, 10, 14 or 21
18) Improvement rate of clinical symptoms within 28-day
    - Definition for clinical symptoms improvement: resolving from fever (axillary temperature ≤36.6°C， or oral temperature ≤37.2°C, or rectal/ear temperature ≤37.8°C), normalization of SpO_2 (SpO_2 >94% on room air), disappearance of respiratory symptom
19) Improvement rate of laboratory parameters within 28-day
    - Definition for laboratory parameters improvement: 50% decline of C-reactive protein, erythrocyte sedimentation rate, IL-6 and TNF-α level from baseline and normalization of absolute blood lymphocyte count.
20) Improvement rate of chest radiology within 28-day
    - Definition for radiology improvement: Resolved more than 50% from baseline on chest CT

images

21) All-cause mortality

22) Clinical status at Day 7, 14, 21, 28 in patients with severe 2019-nCoV respiratory infection

23) For severe disease subgroup: Duration (days) of mechanical ventilation, extracorporeal membrane oxygenation, supplemental oxygenation and hospital stay

24) For mild/moderate subgroup: Progression rate within 28-day

- Definition for progression： progress to severe or critically severe.

- Definition for severe and critically severe 2019-nCoV pneumonia [3]：

  G. Severe—Patients with 2019-nCoV pneumonia and at least one of the following：

     10. Respiratory distress， RR≥30 breaths per minute
     11. SpO2 ≤93% on room air
     12. Partial arterial oxygen pressure (PaO_2) / Fraction of inspired oxygen (FiO_2) ≤300mmHg (1mmHg=0.133kPa).

  H. Critically severe—Patients with severe 2019-nCoV pneumonia and at least one of the following：

     10. Presence of lung failure requiring mechanical ventilation
     11. Presence of shock；
     12. Complicated with other organ failures requiring ICU monitoring

3.3 Safety endpoints

Adverse Events， Laboratory values, ECG, vital signs and physical examination findings will be monitored and recorded.

4 Sample Size Justification

Approximately 360 subjects (180 per group) will be randomized in the study. The sample size for this study is based on the alternative hypothesis of a 30% reduction in the risk of virus nucleic acid negativity. Under the exponential distribution, this benefit translates to a reduction in median virology negativity from 10 days to 7 days. A total of 248 events would provide a power of 80% to detect a reduction of 30% (hazard ratio (control vs. treatment) of 0.70) with a log-rank test, assuming

a two-sided significance level of 5%. A 75-day accrual period and an additional 7-day follow-up are assumed. The sample size calculation has also taken into consideration an annual dropout rate of 20%.

Meanwhile, co-primary endpoint in the subgroup defined by severe disease is 28-day clinical improvement. Assume 50% patients have clinical improvement by day 28 in control group, 100 patients can detect a 25% improvement in treatment group with a power=70%.

5 Analysis Set

Intent-to-treat (ITT): is defined as subjects who have been randomly assigned to the treatment group or placebo group. Analyses of efficacy will be based on this population. The ITT analyses grouping will be based on treatment planned.

Per Protocol Set (PPS)： Is a subset of ITT， defined as all subjects who have been randomly assigned to the study and received at least 11-day treatment(mild/moderate disease) /16-day treatment(severe disease)， and have no major PD which significantly impact primary endpoint. The analyses grouping will be according to treatment planned.

Safety Set（SS）: is defined as subjects who have received at least 1 administration of any study treatment (partial or complete). This population will be used for all safety analyses. The safety analyses grouping will be according to treatment actually received. All patients in control arm will be included in SS and randomization date is used as substitute for treatment date.

6 Statistical Analysis Plan

6.1 General Analysis Method

All analyses will be conducted in SAS 9.2(or higher version). In general, continuous variables will be summarized using descriptive statistics such as mean, standard deviation (SD), median and range. Categorical variables will be summarized using frequency and percentage. For time-to-event variables, the Kaplan-Meier method will be used for descriptive summaries.

In listing, list results that tests/evaluation actually happened. Unless otherwise specified, listing follows the order: treatment, patient ID, visit, parameters.

Study month=number of days/30.4375。

Unless otherwise specified, display of mean and median will have one more digit than original data, standard deviation have 1 more digit than mean. All numerical display do not show more than

3 digits. Percentage has 1 digit and no percentage display if frequency is 0. In general, denominator in percentage is number of patients in the corresponding header.

Primary efficacy analysis is on ITT and supportive efficacy analysis is on PPS. Safety analysis is SS.

6.2 Imputation of Missing Data

（1） If a lab value was recorded as < normal value，≤normal value， > normal value or ≥normal value, the value will be treated as =normal value in the summary tables, but listed as-is in the listings.

（2）Missing/Partial Date of Birth

If year and month and date are missing, then BOB is missing. If year is not missing, use June01 to impute month and date. If year and month are not missing, use date 15 to impute date.

（3） Partial Concomitant Medication Start/End Date

- Partial Concomitant Medication Start Date
  - If the year, month and date are completely missing, no imputation will be performed, the medication was treated as concomitant medication.
  - If the date only is missing, use the date 1 to impute.
  - If the date and month are missing, use January 01 to impute month and date.
- Partial Concomitant Medication End Date
  - If the year, month and date are completely missing, no imputation will be performed, the medication was treated as concomitant medication.
  - If the date only is missing, use the last date of the same month to impute.
  - If the date and month are missing, use Dec 31 to impute month and date.
  - If the end date after imputation is after the last visit date, then use the last visit date as the end date.

（4）Missing/Partial Adverse Event：

- Missing/Partial Adverse Event Onset Date

1) When month and year are present and the day is missing,

  - If the onset month and year are the same as the month and year of first study treatment, the day of first study treatment or the day-component of the AE end date (possibly

imputed) is imputed, whichever is earlier

- ➢ If the onset month and year are not the same as the month and year of first study treatment, then the first day of the month is imputed

2) When only a year is present or no components of the onset date are present,

- ➢ If the onset year is the same as the year of first study treatment. If AE end date is available and is prior to first study treatment, the day and month of AE end date are imputed. Otherwise, the day and month of first study treatment are imputed
- ➢ If the onset year is different from the year of first study treatment, the 1st of January is imputed

3) If the onset date is completely missing, the date of first study treatment is imputed as the onset date.

- Missing/Partial Adverse Event End Date

  If the end date of an adverse event is missing completely or partially, the following imputation rules will be used.

  - ➢ If month and year are present and the day of the month is missing, the last day of the month is imputed.
  - ➢ If only a year is present, the 31st of December is used.
  - ➢ If the imputed date is later than the date of death (if available), the date of death will be used as the imputed date instead.

(4) If the relevance of an AE with the study drug is missing or not evaluable, the AE will be counted in “relevant”.

6.3 The definition of Baseline

Unless otherwise specified, baseline values is defined as values at visit “V1”. The change from baseline is defined as the values-baseline value.

6.4 Disposition

6.4.1 Disposition Information

An overview of subject disposition by treatment group will be provided. The overview includes a summary of total number of subjects who are randomized to each treatment group, the number and percentage of subjects who are randomized but not treated and number and percentage of subjects

who are treated. The number and percentage of subjects who discontinued study treatment including reason for discontinuation as indicated by the investigators will be summarized. The similar summaries will be presented for all randomized subjects who discontinued from study participation.

A listing of subjects who discontinued study treatment including reasons for discontinuation will be provided. A similar listing will be provided for subjects who discontinued study participation.

6.5 Protocol Deviations

Major protocol deviations will be summarized for the ITT population by the following types of deviation for each treatment group:

Entered but did not satisfy inclusion/exclusion criteria

Developed withdrawal criteria but not withdrawn

Received wrong treatment or incorrect dose

Received an excluded concomitant treatment

Efficacy assessment deviation

Other – protocol non-compliance

A list of subjects with major protocol deviations including subject ID, type of deviation, and reasons for deviation will be provided.

6.6 SUBJECT INFORMATION

6.6.1 Demographics and Baseline Characteristics

Unless specified otherwise, all demographic and baseline characteristics variables will be summarized for the ITT population. No statistical comparisons between the 2 treatment groups are planned.

6.6.2 Disease Characteristics

Parameters relevant to the study disease will be summarized in ITT by treatment group, including time from the first positive test to the randomization, time from the first onset of symptoms to the randomization, exposure history, SpO2, onset of fever, onset of respiratory symptom, medication allergy history, etc.

6.6.3 Medical History

Pre-existing conditions and medical history will be coded by MedDra (version 22.0 or higher)

and summarized by SOC and PT.

A listing of subjects who had pre-existing conditions and medical history will be provided.

6.6.4 Historical/Concomitant Medications

Historical medications and concomitant medications will be coded based on WHO DD(the lastest version).

Historical medication is defined as those medication the ending date of which is before randomization date.

Concomitant medication is defined as those medication the ending date of which is after randomization date or missing.

Historical/Concomitant Medication collected in the CRF page during the study will be summarized by therapeutic class, pharmacologic class, and drug name for each treatment group.

6.6.5 Historical/Concomitant Treatment(non-Medications)

Similar analytic approach as for historical/concomitant treatment(medications).

7 Efficacy

Efficacy analysis will be done on both ITT and PPS. Results on ITT is primary.

7.1.1 Primary Efficacy Analysis

The frequency and proportion of virology negativity by day 4、7、10、14、21 will be reported for each group. Proportion is defined by cumulative # of patients who had negativity by the given day/number of ITT in each group. Conversion of virology negativity is defined as two consecutive testing results showing negativity (at least one day interval between two tests) and no result is positive before end of study(or early drop-out). If a planned test is done but result is pending, impute result as “positive”. Unscheduled testing can be used to confirm negativity.

The Kaplan-Meier method will be used to estimate the distribution of time to negativity for each treatment group. The median time to negativity with 95% CI will be provided. In addition, the number and percentage of subjects who had an event or were censored will be reported. The Kaplan-Meier curve will also be plotted by treatment group and will be compared by the stratified log-rank test. The stratification factor is the same as factor considered in the randomization.

The primary treatment comparison of the distribution of overall negativity will be based on a stratified log-rank test. The p-value from a stratified log-rank test will be reported. Hazard ratio and its 95% confidence interval will be estimated based on a stratified Cox's regression model with treatment as the sole explanatory variable. Stratification factors used in the analyses include severity of disease.

In addition, 4, 7, 10, 14, 21 and 28 day of negativity rate with 95% CI will be estimated by Kaplan-Meier method and reported for each treatment group. Determination of dates of event and dates for censoring is summarized in Table 1.

Sensitivity analysis: In order to evaluate the impact of missing testing results, if a planned testing is done but result is pending, impute result as "negativity".

In the listing, list each testing results, date, testing location, results, etc.

Subgroup Analysis: In the severe disease subgroup, the rate of clinical improvement by 28-day will be summarized and compared by chis-square test (or Exact test, if frequency is low). As supporting analysis, KM method may be applied to estimate the rate of clinical improvement by day 28.

Table 7-1 Rule for dates of event and dates for censoring

#	Situation	Date of negativity or censoring	Outcome
1	Two consecutive negative results and no subsequent positive results before the end of study	The first date test result is negative	Event
2	Two consecutive negative results followed by subsequent positive results	The date result is positive	Censored
3	One negative result only before the end of study	The last date test is done	Censored
4	No tests after baseline	Randomization date	Censored

7.1.2 Secondary Efficacy Analysis

Report the rates and relative risk and their 95% confidence interval by the day of 4,7,10,14,21 for secondary endpoints (Negative conversion rate of virus nucleic acid, Improvement rate of clinical symptoms, Improvement rate of laboratory parameters, Improvement rate of chest radiology, All-cause mortality, Clinical status change, progression, etc). Descriptive statistics (mean, standard deviation, median, range) will be used to summarize duration (days) of mechanical ventilation, extracorporeal membrane oxygenation, supplemental oxygenation and hospital stay. If applicable, KM method will be used to analyze time-to-events type endpoints. Median and 95% CI may be reported. KM curves will be compared by log-rank test.

Subgroup Analysis：

- In the severe disease subgroup， report the clinical state change at each time point. Summarize duration (days) of mechanical ventilation, extracorporeal membrane oxygenation, supplemental oxygenation and hospital stay.
- In the mild/moderate subgroup, report progression rate at each time point.。

8 Safety

Safety analysis will be conducted among SS set.

8.1.1 Drug Exposure

Compliance is defined as the actual dose/planned dose*100%.

Summarize compliance the treatment duration. The treatment duration for study drug is defined by the last day of drug use-the first day of drug use and the treatment duration for control group is defined by the last day in study-randomization date.

8.1.2 Adverse Event

The general rule when AE is analyzed:

- Adverse events will be coded using the latest version of Medical Dictionary for Regulatory Activities (MedDRA) coding dictionary and will be recorded in standard medical terminology and graded according to the National Cancer Institute Common Terminology Criteria for Adverse Events (NCI-CTCAE)

- AE will be summarized by actual treatment received.
- If a patient had the same AE for multiple times, in the table summarizing prevalence at patient-level, the highest grade of AE will be summarized. In the table summarizing prevalence at incidence-level, The same AE on the same patient on the same date, the highest grade will be summarized.
- Summary of AE events does not come alone with percentage.
- When summarizing a subgroup of AE which prevalence is greater than a given percentage, search the AE within SOC/PT.

The incidence and prevalence of AEs will be summarized overall, by treatment group, by MedDRA system organ class (SOC) and preferred term, by toxicity grade, and by relationship to study treatment administration. Specifically, the following AE summaries will be presented by treatment group:

- Overview of AEs
- TEAEs
- TEAE sCTCAE Grade >=3
- TEAEs Leading to discontinuation of study drug
- TEAEs leading to death
- TEAEs related to study drug
- TEAEs related to study drug and CTCAE grade>=3
- SAEs
- TEAEs related to study drug
- ADRs

TEAEs are defined as any AE that occurs after randomization through 30 days after the last study treatment; or the day prior to start of subsequent therapy, whichever is earlier; or any AE that is considered drug-related (very likely, probably, or possibly related) regardless of the start date of the event; or any AE that is present at baseline but worsens in toxicity grade or is subsequently considered drug-related by the investigator.

ADRs are defined as any TEAE that is considered drug-related (very likely, probably, or possibly related, or non-evaluable).

List all AEs, including SOC/PT, start/end date, CTCAE grade, relationship to drug, SAE, ADR,

outcome

8.1.3 Clinical Laboratory Tests

Descriptive statistics (mean, standard deviation, median, range) will be used to summarize observed laboratory values (Hematology panel, Blood chemistry panel, coagulation test, urinalysis) and change from baseline in observed value at each scheduled visit for each treatment group. Shift table for baseline value (normal vs abnormal but not clinical meaningful vs. abnormal and clinical meaningful) and post-baseline value will be provided.

8.1.4 Electrocardiogram (ECG)

ECG will be summarized by visit and by treatment group. Shift table for baseline value (normal vs abnormal but not clinical meaningful vs. abnormal and clinical meaningful) and post-baseline value may be provided.

8.1.5 Vital Signs

Vital signs (systolic and diastolic blood pressure, heart rate, and temperature) values and change from baseline will be summarized by visit and by treatment group. Shift table for baseline value (normal vs abnormal but not clinical meaningful vs. abnormal and clinical meaningful) and post-baseline value may be provided.

8.1.6 Physical Examination Findings

Physical examination findings will be summarized by visit and by treatment group. Shift table for baseline value (normal vs abnormal but not clinical meaningful vs. abnormal and clinical meaningful) and the worst post-baseline value may be provided.

9 Interim Analysis

An Independent Data Monitoring Committee (IDMC) will be established to review data at 1 interim analyses prior to the primary endpoint analysis.

The following cumulative α-spending function (O'Brien-Fleming type by Lan-DeMets algorimth)（Lan-Demets，1983）will be applied to control family-wise type-I error (0.05)

$$\alpha(t_k) = 2\left(1 - \Phi\left(\Phi^{-1}\left(1 - \frac{\alpha}{2}\right) \Big/ \sqrt{t_k}\right)\right)$$

where, t_k is the cumulative information at the time of kth analysis, Φ is standard normal distribution, Φ^{-1} quantile function. The cumulative alpha spending function $\alpha(t_k)$ is a function of cumulative information, therefore, the significance level (p-value boundary) is a function of cumulative information, i.e., the observed number of events.

The first interim analysis will occur after 40% of subjects (around 150 subjects) have been treated for at least 7 days or have discontinued. The goal is to evaluate efficacy and need of sample size re-estimation. P-value boundary for efficacy significance is 0.00079(two-sided).

Final analysis will be conducted after 248 events are observed. P-value boundary is 0.04974(two-sided).

The actual alpha spent and p-value boundaries at each analysis will be calculated based on the exact number of events.

10 TFL shells

TFL shells will be provided in a separate file.

11 References

1. K. K. GORDON LAN, DAVID L. DEMETS, **Discrete sequential boundaries for clinical trials**, Biometrika, Volume 70, Issue 3, December 1983, Pages 659–663

Final Case Report Form: KY2020-29

Version date. 25 February 2020

This is the translated version of the final Case Report Form originally written in Chinese.

Hydroxychloroquine sulfate in Adult Patients With 2019-nCoV Respiratory Infection: A Multicenter, Randomized, Open-Label，Controlled Study

Case Report Form

Center Number ：	|__|__|
Subject Number ：	|__|__|__|__|__|
Abbreviation of Subject's Name：	|__|__|__|__|
Name of Center （regular script）：	____________________
Name of Doctor （regular script）：	____________________
The Version and Date ：	Version 3.0 /February 25,2020
Applicable Clinical Type ：	Mild & Common type

CONFIDENTIAL

The information contained here relates to trade secrets and business information. These business information terms confidential content or proprietary information, except as required by regulations, shall not be disclosed by any person. Any individual who has access to the document shall be aware of its proprietary or confidential nature and shall not disclose the document without authorization. The restrictions on the confidentiality of the above documents will apply to any information provided to you in the future that is marked as proprietary or confidential.

Guide to Fill out the Form

1. The case report form shall be recorded by an authorized person, and each page in the CRF shall be carefully checked and signed.
2. The trial physicians should be familiar with the trial protocol and master the GCP principles.
3. Please write clearly and legibly with a sign pen in blue or black ink, preferably in capital letters for English, for example：CASE REPORT FORM
4. When filling in Numbers in open squares, fill in one number in each box；If the number of digits is less than the corresponding number of squares, please fill with zero. Spaces are not allowed. For example：56Kg，fill in the blanks as follows:| 0 | 5 | 6 |. | 0 | 0 |Kg.
5. The date is in the international ISO8601 date format：yyyymmdd. For example, December 21, 2007 was written as| 2 | 0 | 0 | 7 |YEAR| 1 | 2 |Month| 2 | 1 |DAY。
6. For the items with "□"in the form, please type "×"in the corresponding box, , or fill in the relevant Numbers. If not done or the data is not available, please fill in ND(Not Done) or NK(not known) in the corresponding space; NA is not applicable.
7. Each item must be filled in accurately, do not arbitrarily alter, do not use correction fluid or other ways to cover the original data. If you find any errors, please cross out the wrong record with a single line so that the original record is still legible, then fill in the correct record at the top right, indicating the name, abbreviation and date of modification. For example：~~58.6~~ 56.8 ZH 2007-12-21.
8. The four of the Chinese phoneticize abbreviation of the patient's name should be filled in. Fill in the first two letters of each word for the two-character name; fill in the first letter of three words and the second letter of the third word or the three-character name; and fill in the first letter of each word for the four-character name. The Chinese phoneticize abbreviation of each patient's name should be unified in their CRF. For example：Zhang Hong| Z | H | H | 0 |，Li Shuming| L | S |M | I |，Ou Yang Xiaohui| O | Y | X | H |.
9. Do not change the format of the case report form. If you find that there is no place in the form to fill in the information the recorder wishes to record, please contact the responsible CRA.
10. Upon completion of the case report form, the principal investigator shall examine each page of the case report form and sign at the investigator's statement.

Date of Current Visit： |__|__|__|__|Year|__|__|MONTH|__|__|DAY

Sign the Informed Consent	
Whether to sign informed consent	□0 NO □1 YES
Date of signing the Informed Consent	|__|__|__|__|YEAR|__|__|MONTH|__|__|DAY
Version number of Informed Consent	____________
The version number of the protocol shall apply when the informed consent is signed	|__|__|.|__|
**Confirm Inclusion/Exclusion Criteria**	
Do subjects meet all inclusion criteria and not all exclusion criteria?	□1 YES □0 NO
□1 Does not meet the selection criteria, the selection criteria number (multiple choices)	□1□2 □3□4□5□6 □7<br>□Other，Please describe：________
□2 Meet the exclusion criteria,exclusion criteria number (multiple choices)	□1□2 □3□4□5□6 □7 □8 □9<br>□ Other，Please describe：________

Demographic Data	
Gender	□1male □2female
Date of Birth	|__|__|__|__|YEAR|__|__|MONTH|__|__|DAY If not available, please state age：|__|__| years old
Nationality	□1Han □2Other，Please describe：______________

History of 2019-nCoV Respiratory Infection	
Date of first onset	|__|__|__|__|YEAR|__|__|MONTH|__|__|DAY
History of exposure to related causes within 14 days prior to onset	□0NO □1YES □2 Unknown
Contact type (multiple choice)	□1 Contact with Hubei region<br>□2 Contact with person infected with 2019-nCoV<br>□3 Unknown<br>□4 Other，please describe ：______________________

Previous anti-2019-nCoV treatment or not	□0NO □1YES
Date of first positive nucleic acid test	|__|__|__|__|YEAR|__|__|MONTH|__|__|DAY
Clinical type at enrollment	□1Mild □2 Common □3 severe □4 critical
Note: If the subject ever had a treatment for 2019-nCoV pneumonia, please fill in the form "past/combined drug therapy" and "past/combined non-drug therapy".	

**History of Drug Allergy**
Does the subject have a history of drug allergy?<br>□0NO □1YES, Please describe : ____<br>____

Past/Concomitant Disease History

Does the subject have any past/concomitant disease other than 2019-nCoV pneumonia?
□0 NO □1 YES, Please fill in the form below.

Number	Disease	Confirmed Date	Disease persists or not?	End Date	Receive treatment or not?
1		|__|__|__|__|YEAR |__|__|MONTH| __|__|DAY	□0 NO □1 YES	|__|__|__|__|YEAR |__|__|MONTH| __|__|DAY	□0 NO □1 YES
2		|__|__|__|__|YEAR |__|__|MONTH| __|__|DAY	□0 NO □1 YES	|__|__|__|__|YEAR |__|__|MONTH| __|__|DAY	□0 NO □1 YES
3		|__|__|__|__|YEAR |__|__|MONTH| __|__|DAY	□0 NO □1 YES	|__|__|__|__|YEAR |__|__|MONTH| __|__|DAY	□0 NO □1 YES
4		|__|__|__|__|YEAR |__|__|MONTH| __|__|DAY	□0 NO □1 YES	|__|__|__|__|YEAR |__|__|MONTH| __|__|DAY	□0 NO □1 YES
5		|__|__|__|__|YEAR |__|__|MONTH| __|__|DAY	□0 NO □1 YES	|__|__|__|__|YEAR |__|__|MONTH| __|__|DAY	□0 NO □1 YES
6		|__|__|__|__|YEAR |__|__|MONTH| __|__|DAY	□0 NO □1 YES	|__|__|__|__|YEAR |__|__|MONTH| __|__|DAY	□0 NO □1 YES
7		|__|__|__|__|YEAR |__|__|MONTH| __|__|DAY	□0 NO □1 YES	|__|__|__|__|YEAR |__|__|MONTH| __|__|DAY	□0 NO □1 YES

8		|__|__|__|__|YEAR |__|__|MONTH| __|__|DAY	□0 NO □1 YES	|__|__|__|__|YEAR |__|__|MONTH| __|__|DAY	□0 NO □1 YES
9		|__|__|__|__|YEAR |__|__|MONTH| __|__|DAY	□0 NO □1 YES	|__|__|__|__|YEAR |__|__|MONTH| __|__|DAY	□0 NO □1 YES
10		|__|__|__|__|YEAR |__|__|MONTH| __|__|DAY	□0 NO □1 YES	|__|__|__|__|YEAR |__|__|MONTH| __|__|DAY	□0 NO □1 YES

**Vital Signs** □0 Unexamined，reason: ________________			
Date	|__|__|__|__|YEAR|__|__|MONTH|__|__|DAY		
Daily maximum temperature	|__|__|.|__|℃	Measurement method	□1 Axilla □2 Oral □3 Anus □4 Ear □5 Unknown □6 Other，please describe________
Respiratory	|__|__|times/min	Pulse	|__|__|__|times/min
Blood pressure	|__|__|__|/|__|__|__| mmHg（Systolic/diastolic blood pressure）		

**physical examination** □0 Unexamined, **reason**:________________					
Date	|__|__|__|__|YEAR|__|__|MONTH|__|__|DAY				
Inspection Part	Result<br>1= Normal<br>2= Abnormal, no clinical significance<br>3= Abnormal, with clinical significance<br>0=Unexamined				If abnormal，please describe
General appearance	□1	□2	□3	□0	
Head	□1	□2	□3	□0	
Eyes	□1	□2	□3	□0	
Nose	□1	□2	□3	□0	
Larynx	□1	□2	□3	□0	

Neck and thyroid	□1	□2	□3	□0	
Skin	□1	□2	□3	□0	
Cardiovascular system	□1	□2	□3	□0	
Chest and lungs	□1	□2	□3	□0	
Abdomen	□1	□2	□3	□0	
Nervous system	□1	□2	□3	□0	
Musculoskeletal system	□1	□2	□3	□0	
Lymphatic system	□1	□2	□3	□0	

Respiratory symptoms □0Unexamined, reason: ________________________

Date: |__|__|__|__|YEAR|__|__|MONTH|__|__|DAY

Whether the subject has respiratory symptoms or not: □0 NO □1 YES, Please describe: _____

____

Blood Count □0 Unexamined, reason________________________

Date: |__|__|__|__|YEAR|__|__|MONTH|__|__|DAY Normal range Number. |__|__|__|__|

Item	Result		Result Interpretation			Unexamined
	measured values	Unit	Normal	Abnormal: No clinical significance	Abnormal: Significant clinically	
Red Blood Cell (RBC)		□1 10^{12}/L □2 Other: ___	□1	□2	□3	□0
White blood cell		□1 10^{9}/L □2 Other: ___	□1	□2	□3	□0
Hemoglobin (Hb)		□1 g/L □2 Other: ___	□1	□2	□3	□0
Platelet (PLT)		□1 10^{9}/L □2 Other: _____	□1	□2	□3	□0
Neutrophil (ANC)		□1 10^{9}/L □2 Other: _____	□1	□2	□3	□0
Lymphocyte (LY)		□1 10^{9}/L □2 Other: _____	□1	□2	□3	□0
Monocyte (MO)		□1 10^{9}/L □2 Other: _____	□1	□2	□3	□0
Eosinophil (EO)		□1 10^{9}/L □2 Other: _____	□1	□2	□3	□0
Basophil (BA)		□1 10^{9}/L □2 Other: _____	□1	□2	□3	□0

Blood Biochemistry □0 Unexamined, reason________________________

Date: \|__\|__\|__\|__\|YEAR\|__\|__\|MONTH\|__\|__\|DAY Normal range Number: \|__\|__\|__\|__\|

Item	Result		Result Interpretation			Unexamined
	measured value	Unit	Normal	Abnormal		
				No clinical significance	Significant clinically	
Glutamic-pyruvic transaminase（ALT）		□1 U/L □2 Other: _____	□1	□2	□3	□0
Glutamic-oxalacetic transaminease（AST）		□1 U/L □2 Other: _____	□1	□2	□3	□0
Total bilirubin（TBIL）		□1 μmol/L □2 Other: _____	□1	□2	□3	□0
Albumin（ALB）		□1 g/L □2 Other: _____	□1	□2	□3	□0
γ- glutamyl transpeptidas（GGT）		□1 U/L □2 Other: _____	□1	□2	□3	□0
Lactic dehydrogenase（LDH）		□1 U/L □2 Other: _____	□1	□2	□3	□0
Creatine kinase（CK）		□1 U/L □2 Other: _____	□1	□2	□3	□0
Creatine kinase isoenzyme（CK-MB）		□1 U/L □2 Other: _____	□1	□2	□3	□0
Blood urea nitrogen（BUN）		□1 mmol/L □2 Other: _____	□1	□2	□3	□0
Urea（Urea）		□1 mmol/L □2 Other: _____	□1	□2	□3	□0
Serum creatinine（CR）		□1 μmol/L □2 Other: _____	□1	□2	□3	□0

Urine Test □0 Unexamined, reason________________________________

Date: \|__\|__\|__\|__\|YEAR\|__\|__\|MONTH\|__\|__\|DAY Normal range Number: \|__\|__\|__\|__\|

Item	Result		Result Interpretation			Unexamined
	measured value	Unit	Normal	Abnormal		
				No clinical significance	Significant clinically	
Urine Protein		□1Not Applicable □2 Other : _____	□1	□2	□3	□0
Urine Glucose		□1 mmol/L □2 Other : _____	□1	□2	□3	□0
Urine PH value		□1 Not Applicable □2 Other : _____	□1	□2	□3	□0
Urine Erythrocyte		□1 Not Applicable □2 Other : _____	□1	□2	□3	□0
Urine Leukocyte		□1 Not Applicable □2 Other : _____	□1	□2	□3	□0
Urine Ketone		□1 Not Applicable □2 Other : _____	□1	□2	□3	□0

Coagulation Function □0 Unexamined, reason____________________

Date: \|__\|__\|__\|__\|YEAR\|__\|__\|MONTH\|__\|__\|DAY Normal range Number: \|__\|__\|__\|__\|

Item	Result			Result Interpretation			Unexamined Unit
	measured value	Unit		Normal	Abnormal		
					No clinical significance	Significant clinically	
ProthrombinTime（PT）		□1 S	□2 Other: ______	□1	□2	□3	□0
Activated Partial Thromboplastin Time（APTT）		□1 S	□2 Other: ______	□1	□2	□3	□0
Thrombin Time（TT）		□1 S	□2 Other: ______	□1	□2	□3	□0
Fibrinogen（Fbg）		□1 g/L	□2 Other: ______	□1	□2	□3	□0
International Normalized Ratio（INR）		□1 Not Applicable	□2 Other: ______	□1	□2	□3	□0

C-Reaction Protein（CRP） □0 Unexamined, reason____________________

Date: \|__\|__\|__\|__\|YEAR\|__\|__\|MONTH\|__\|__\|DAY Normal range Number: \|__\|__\|__\|__\|

Item	Result		Result Interpretation		
C-Reactive Protine（CRP）	Measured Value	Unit	Normal	Abnormal	
				No clinical significance	Significant clinically
		□1 mg/L □2 Other: __	□1	□2	□3

Erythrocyte Sedimentation Rate（ESR） □0 Unexamined, reason____________________

Date: \|__\|__\|__\|__\|YEAR\|__\|__\|MONTH\|__\|__\|DAY Normal range Number: \|__\|__\|__\|__\|

Item	Result		Result Interpretation		
Erythrocyte Sedimentation Rate（ESR）	Measured Value	Unit	Normal	Abnormal	
				No clinical significance	Significant clinically
		□1 mm/h □2 Other:	□1	□2	□3

TNF-α □0 Unexamined, reason____________________

Date: \|__\|__\|__\|__\|YEAR\|__\|__\|MONTH\|__\|__\|DAY Normal range Number: \|__\|__\|__\|__\|

Item	Result			Result Interpretation		
TNF-α	Measured Value	Unit		Normal	U	
					No clinical significance	Significant clinically
		□1 pg/mL	□2 Other:	□1	□2	□3

IL-6 □0Unexamined，reason______						
Date：|__|__|__|__|YEAR|__|__|MONTH|__|__|DAY Normal range Number：|__|__|__|__|						
Item	Result			Result Interpretation		
	Measured Value	Unit		Normal	Abnormal	
					No clinical significance	Significant clinically
IL-6		□1 pg/mL	□2Other：	□1	□2	□3

Urine or serum HCG（Only applicable to women of childbearing age）<br>□0Unexamined，reason______<br>□1Not Applicable（Man or woman who isn't able to reproduce）
Date：|__|__|__|__|YEAR|__|__|MONTH|__|__|DAY
Sample：□1 Blood □2 Urine
Result：□1 Negative □2 Positive

Routine 12 Leads Electrocardiogram □0Unexamined，reason______
Date：|__|__|__|__|YEAR|__|__|MONTH|__|__|DAY
Result：□1 Normal □2 Abnormal，No clinical significance □3 Abnormal，Significant clinically
If"Abnormal"，please describe：______<br>______

Chest CT □0Unexamined，reason______
Date：|__|__|__|__|YEAR|__|__|MONTH|__|__|DAY
Result：______<br>______

Nucleic Acid Test of 2019-nCov □0 Unexamined，reason______	
Sampling Part（mutiple choice）：□1Upper respiratory tract □2Lower respiratory tract	
Upper respiratory tract	Date：|__|__|__|__|YEAR|__|__|MONTH|__|__|DAY<br>Result：□1 Negative □2 Positive □3 Unidentified □4 Other，please describe ______
Lower respiratory tract	Date：|__|__|__|__|YEAR|__|__|MONTH|__|__|DAY<br>Result：□1 Negative □2 Positive □3 Unidentified □4 Other，please describe ______

Result	□1 Negative □2 Positive □3 Unidentified □4 Other, please describe ____________

**Pulse oxygen saturation (without oxygen inhalation)** □0Unexamined, reason_____
Date: |__|__|__|__|YEAR|__|__|MONTH|__|__|DAY
Measured Value: _______________% (Rest state)

**Screening Result** □0 Not Evaluation	
Is the subject screened successfully?<br>□0 NO, the main cause of failure screening: ______________________________<br>□1 YES, please randomize	
Randomize Date: |__|__|__|__|YEAR|__|__|MONTH|__|__|DAY	
Randomize Number:	
Randomized Group	□1 Experimental group □2 Control group

If any adverse events have occurred since the signing of the informed consent, please fill in the form of "adverse events"; If there is medication combination, please fill in the form "previous/combined medication"; If there is combined non-drug therapy, please fill in the "previous/combined non-drug therapy" form; If there is an out-of-plan inspection, please fill in the "out-of-plan inspection" form; If there is IMV/NIV, ECMO, HFNC, oxygen therapy, please fill in the "IMV/NIV, ECMO, HFNC, oxygen therapy" form.

Date of Current Visit：\|__\|__\|__\|__\|YEAR\|__\|__\|MONTH\|__\|__\|DAY

**Vital Signs**	□0Unexamined，reason________		
Date	|__|__|__|__|YEAR|__|__|MONTH|__|__|DAY		
Daily maximum temperature	|__|__|.|__|℃	Measurement method	□1 Axilla □2Oral □3 Anus □4 Ear □5 Unknown □6 Other，please describe________
Respiratory	|__|__|times/min	Pulse	|__|__|__|times/min
Blood pressure	|__|__|__|/|__|__|__| mmHg（Systolic/diastolic blood pressure）		

**Respiratory symptoms** □0Unexamined，reason________
Date：|__|__|__|__|YEAR|__|__|MONTH|__|__|DAY
Whether the subject has respiratory symptoms or not：□0 NO □1 NO，please describe：________
Result：□1 Normal □2 AbnormalNo clinical significance □3 AbnormalSignificant clinically

**Blood Count** □0Unexamined，reason________
Date：|__|__|__|__|YEAR|__|__|MONTH|__|__|DAY Normal range Number：|__|__|__|__|

Item	Result		Result Interpretation			Unexamined
	Measured Value	Unit	Normal	Abnormal		
				No clinical significance	Significant clinically	
Red Blood Cell（RBC）		□1 10^{12}/L □2Other：_____	□1	□2	□3	□0
White Blood Cell		□1 10^{9}/L □2Other：_____	□1	□2	□3	□0
Hemoglobin（Hb）		□1 g/L □2Other：_____	□1	□2	□3	□0
Platelet（PLT）		□1 10^{9}/L □2Other：_____	□1	□2	□3	□0
Neutrophil（ANC）		□1 10^{9}/L □2Other：_____	□1	□2	□3	□0
Lymphocyte（LY）		□1 10^{9}/L □2Other：_____	□1	□2	□3	□0
Monocyte（MO）		□1 10^{9}/L □2Other：_____	□1	□2	□3	□0
Eosinophil（EO）		□1 10^{9}/L □2Other：_____	□1	□2	□3	□0
Basophil（BA）		□1 10^{9}/L □2Other：_____	□1	□2	□3	□0

**Blood Biochemistry** □0Unexamined，reason________

Date|__|__|__|__|YEAR|__|__|MONTH|__|__|DAY Normal range Number: |__|__|__|__|

Item	Result		Result Interpretation			Unexamined
	Measured Value	Unit	Normal	Abnormal		
				No clinical significance	Significant clinically	
Glutamic-pyruvic transaminase（ALT）		□1 U/L □2Other: _____	□1	□2	□3	□0
Glutamic-oxalacetic transaminease（AST）		□1 U/L □2Other: _____	□1	□2	□3	□0
Total Bilirubin（TBIL）		□1 μmol/L □2Other: _____	□1	□2	□3	□0
Albumin（ALB）		□1 g/L □2Other: _____	□1	□2	□3	□0
γ- glutamyl transpeptidas（GGT）		□1 U/L □2Other: _____	□1	□2	□3	□0
Lactic dehydrogenase（LDH）		□1 U/L □2Other: _____	□1	□2	□3	□0
Creatine kinase（CK）		□1 U/L □2Other: _____	□1	□2	□3	□0
Creatine kinase isoenzyme（CK-MB）		□1 U/L □2Other: _____	□1	□2	□3	□0
Blood urea nitrogen（BUN）		□1 mmol/L □2Other: _____	□1	□2	□3	□0
Urea（Urea）		□1 mmol/L □2Other: _____	□1	□2	□3	□0
Serum creatinine（CR）		□1 μmol/L □2Other: _____	□1	□2	□3	□0

Urine Test □0Unexamined, reason________________________________
□1 Not Applicable

Date: |__|__|__|__|YEAR|__|__|MONTH|__|__|DAY Normal range Number: |__|__|__|__|

Item	Result		Result Interpretation			Unexamined
	Measured Value	Unit	Normal	Abnormal		
				No clinical significance	Significant clinically	
Urine Protein		□1Not Applicable □2Other: _____	□1	□2	□3	□0
Urine Glucose		□1 mmol/L □2Other: _____	□1	□2	□3	□0
Urine PH value		□1 Not Applicable □2Other: _____	□1	□2	□3	□0
Urine Erythrocyte		□1Not Applicable □2Other: _____	□1	□2	□3	□0
Urine Leukocyte		□1Not Applicable □2Other: _____	□1	□2	□3	□0
Urine Ketone		□1Not Applicable □2Other: _____	□1	□2	□3	□0

**Coagulation Function** □0Unexamined，reason________							
Date：|__|__|__|__|YEAR|__|__|MONTH|__|__|DAY Normal range Number：|__|__|__|__|							
**Item**	**Result**		**Result Interpretation**			**Unexamined**	
	**Measured Value**	**Unit**	**Normal**	**Abnormal**			
				**No clinical significance**	**Significant clinically**		
ProthrombinTime（PT）		□1 S □2Other：_____	□1	□2	□3	□0	
Activated Partial Thromboplastin Time（APTT）		□1 S □2Other：_____	□1	□2	□3	□0	
Thrombin Time（TT）		□1 S □2Other：_____	□1	□2	□3	□0	
Fibrinogen（Fbg）		□1 g/L □2Other：_____	□1	□2	□3	□0	
International Normalized Ratio（INR）		□1Not Applicable □2Other：_____	□1	□2	□3	□0	

**C-REACTION PROTEIN（CRP）** □0Unexamined，reason________					
Date：|__|__|__|__|YEAR|__|__|MONTH|__|__|DAY Normal range Number：|__|__|__|__|					
**Item**	**Result**		**Result Interpretation**		
C-REACTION PROTEIN（CRP）	**Measured Value**	**Unit**	**Normal**	**Abnormal**	
				**No clinical significance**	**Significant clinically**
		□1 mg/L □2Other：_____	□1	□2	□3

**Erythrocyte Sedimentation Rate（ESR）** □0Unexamined，reason________					
Date：|__|__|__|__|YEAR|__|__|MONTH|__|__|DAY Normal range Number：|__|__|__|__|					
**Item**	**Result**		**Result Interpretation**		
Erythrocyte Sedimentation Rate（ESR）	**Measured Value**	**Unit**	**Normal**	**Abnormal**	
				**No clinical significance**	**Significant clinically**
		□1 mm/h □2Other：	□1	□2	□3

**TNF-α** □0Unexamined，reason________					
Date：|__|__|__|__|YEAR|__|__|MONTH|__|__|DAY Normal range Number：|__|__|__|__|					
**Item**	**Result**		**Result Interpretation**		
TNF-α	**Measured Value**	**Unit**	**Normal**	**Abnormal**	
				**No clinical significance**	**Significant clinically**
		□1 pg/mL □2Other：	□1	□2	□3

**IL-6** □0Unexamined, reason________						
Date: |__|__|__|__|YEAR|__|__|MONTH|__|__|DAY Normal range Number: |__|__|__|__|						
**Item**	**Result**			**Result Interpretation**		
					**Abnormal**	
IL-6	**Measured Value**	**Unit**		**Normal**	**No clinical significance**	**Significant clinically**
		□1 pg/mL	□2Other:	□1	□2	□3

**Nucleic Acid Test of 2019-nCov** □0 Unexamined, reason________ □1 Not Applicable
Sampling Part: □1Upper respiratory tract □2Lower respiratory tract
Date: |__|__|__|__|YEAR|__|__|MONTH|__|__|DAY Date: |__|__|:|__|__|
Result: □1 Negative □2 Positive □3 Unidentified □4 Other, please describe________

**Pulse oxygen saturation (without oxygen inhalation)** □0Unexamined, reason_____
Date: |__|__|__|__|YEAR|__|__|MONTH|__|__|DAY
Measured Value: ____________% (Rest state)

**Whether develops into severe/critical type** □0 No evaluation Date: |__|__|__|__|YEAR|__|__|MONTH|__|__|DAY
Whether develops into severe/critical type: □0 NO □1 YES
Date of developing into severe/critical type: |__|__|__|__|YEAR|__|__|MONTH|__|__|DAY

If any adverse events have occurred since the last visit, please fill in the "Adverse Events Form". If there is medication combination, please fill in the "Previous/Combined medication Form"; If there is combined non-drug therapy, please fill in the " Comined Non-Drug Therapy Form"; If there is an out-of-plan inspection, please fill in the "Out-Of-Plan Inspection Form".

Date of Current Visit： |__|__|__|__|YEAR|__|__|MONTH|__|__|DAY

**Vital Signs** □0Unexamined，reason________			
Date	|__|__|__|__|YEAR|__|__|MONTH|__|__|DAY		
Daily maximum temperature	|__|__|.|__|℃	Measurement method	□1 Axilla □2 Oral □3 Anus □4 Ear □5 Unknown □6 Other，please describe________
Respiratory	|__|__|times/min	Pulse	|__|__|__|times/min
Blood pressure	|__|__|__|/|__|__|__| mmHg（Systolic/diastolic blood pressure）		

**Respiratory symptoms** □0Unexamined，reason________
Date：|__|__|__|__|YEAR|__|__|MONTH|__|__|DAY
Whether the subject has respiratory symptoms or not：□0 NO □1 NO，please describe：________
Result：□1 Normal □2 AbnormalNo clinical significance □3 AbnormalSignificant clinically

Blood Count □0Unexamined，reason________

Date：|__|__|__|__|YEAR|__|__|MONTH|__|__|DAY Normal range Number：|__|__|__|__|

Item	Result		Result Interpretation			Unexamined
	Measured Value	Unit	Normal	Abnormal		
				No clinical significance	Significant clinically	
Red Blood Cell（RBC）		□1 10^{12}/L □2Other：_____	□1	□2	□3	□0
White Blood Cell		□1 10^{9}/L □2Other：_____	□1	□2	□3	□0
Hemoglobin（Hb）		□1 g/L □2Other：_____	□1	□2	□3	□0
Platelet（PLT）		□1 10^{9}/L □2Other：_____	□1	□2	□3	□0
Neutrophil（ANC）		□1 10^{9}/L □2Other：_____	□1	□2	□3	□0
Lymphocyte（LY）		□1 10^{9}/L □2Other：_____	□1	□2	□3	□0
Monocyte（MO）		□1 10^{9}/L □2Other：_____	□1	□2	□3	□0
Eosinophil（EO）		□1 10^{9}/L □2Other：_____	□1	□2	□3	□0
Basophil（BA）		□1 10^{9}/L □2Other：_____	□1	□2	□3	□0

Blood Biochemistry □0Unexamined，reason________

Date\|__\|__\|__\|__\|YEAR\|__\|__\|MONTH\|__\|__\|DAY Normal range Number：\|__\|__\|__\|__\|

Item	Result		Result Interpretation			Unexamined
	Measured Value	Unit	Normal	Abnormal: No clinical significance	Abnormal: Significant clinically	
Glutamic-pyruvic transaminase（ALT）		□1 U/L □2Other：_____	□1	□2	□3	□0
Glutamic-oxalacetic transaminease（AST）		□1 U/L □2Other：_____	□1	□2	□3	□0
Total Bilirubin（TBIL）		□1 μmol/L □2Other：_____	□1	□2	□3	□0
Albumin（ALB）		□1 g/L □2Other：_____	□1	□2	□3	□0
γ- glutamyl transpeptidas（GGT）		□1 U/L □2Other：_____	□1	□2	□3	□0
Lactic dehydrogenase（LDH）		□1 U/L □2Other：_____	□1	□2	□3	□0
Creatine kinase（CK）		□1 U/L □2Other：_____	□1	□2	□3	□0
Creatine kinase isoenzyme（CK-MB）		□1 U/L □2Other：_____	□1	□2	□3	□0
Blood urea nitrogen（BUN）		□1 mmol/L □2Other：_____	□1	□2	□3	□0
Urea（Urea）		□1 mmol/L □2Other：_____	□1	□2	□3	□0
Serum creatinine（CR）		□1 μmol/L □2Other：_____	□1	□2	□3	□0

Urine Test □0Unexamined，reason________________________________
□1 Not Applicable

Date：\|__\|__\|__\|__\|YEAR\|__\|__\|MONTH\|__\|__\|DAY Normal range Number：\|__\|__\|__\|__\|

Item	Result		Result Interpretation			Unexamined
	Measured Value	Unit	Normal	Abnormal: No clinical significance	Abnormal: Significant clinically	
Urine Protein		□1Not Applicable □2Other：_____	□1	□2	□3	□0
Urine Glucose		□1 mmol/L □2Other：_____	□1	□2	□3	□0
Urine PH value		□1 Not Applicable □2Other：_____	□1	□2	□3	□0
Urine Erythrocyte		□1Not Applicable □2Other：_____	□1	□2	□3	□0
Urine Leukocyte		□1Not Applicable □2Other：_____	□1	□2	□3	□0
Urine Ketone		□1Not Applicable □2Other：_____	□1	□2	□3	□0

**Coagulation Function** □0Unexamined, reason________							
Date: |__|__|__|__|YEAR|__|__|MONTH|__|__|DAY Normal range Number: |__|__|__|__|__|							
**Item**	**Result**		**Result Interpretation**				**Unexamined**
	**Measured Value**	**Unit**	**Normal**	**Abnormal**			
				**No clinical significance**	**Significant clinically**		
ProthrombinTime（PT）		□1 S □2Other: _____	□1	□2	□3		□0
Activated Partial Thromboplastin Time（APTT）		□1 S □2Other: _____	□1	□2	□3		□0
Thrombin Time（TT）		□1 S □2Other: _____	□1	□2	□3		□0
Fibrinogen（Fbg）		□1 g/L □2Other: _____	□1	□2	□3		□0
International Normalized Ratio（INR）		□1Not Applicable □2Other: _____	□1	□2	□3		□0

**C-REACTION PROTEIN（CRP）** □0Unexamined, reason________					
Date: |__|__|__|__|YEAR|__|__|MONTH|__|__|DAY Normal range Number: |__|__|__|__|__|					
**Item**	**Result**		**Result Interpretation**		
C-REACTION PROTEIN（CRP）	**Measured Value**	**Unit**	**Normal**	**Abnormal**	
				**No clinical significance**	**Significant clinically**
		□1 mg/L □2Other: _____	□1	□2	□3

**Erythrocyte Sedimentation Rate（ESR）** □0Unexamined, reason________					
Date: |__|__|__|__|YEAR|__|__|MONTH|__|__|DAY Normal range Number: |__|__|__|__|__|					
**Item**	**Result**		**Result Interpretation**		
Erythrocyte Sedimentation Rate（ESR）	**Measured Value**	**Unit**	**Normal**	**Abnormal**	
				**No clinical significance**	**Significant clinically**
		□1 mm/h □2Other:	□1	□2	□3

**TNF-α** □0Unexamined, reason________						
Date: |__|__|__|__|YEAR|__|__|MONTH|__|__|DAY Normal range Number: |__|__|__|__|__|						
**Item**	**Result**			**Result Interpretation**		
TNF-α	**Measured Value**	**Unit**		**Normal**	**Abnormal**	
					**No clinical significance**	**Significant clinically**
		□1 pg/mL	□2Other:	□1	□2	□3

<table>
<tr><td colspan="7">IL-6 □0Unexamined，reason______________________________</td></tr>
<tr><td colspan="7">Date： |__|__|__|__|YEAR|__|__|MONTH|__|__|DAY Normal range Number： |__|__|__|__|</td></tr>
<tr><th>Item</th><th colspan="3">Result</th><th colspan="3">Result Interpretation</th></tr>
<tr><td rowspan="3">IL-6</td><td rowspan="2">Measured Value</td><td rowspan="2" colspan="2">Unit</td><td rowspan="2">Normal</td><td colspan="2">Abnormal</td></tr>
<tr><td>No clinical significance</td><td>Significant clinically</td></tr>
<tr><td></td><td>□1 pg/mL</td><td>□2Other：</td><td>□1</td><td>□2</td><td>□3</td></tr>
</table>

**Nucleic Acid Test of 2019-nCov** □0 Unexamined，reason______________ □1 Not Applicable
Sampling Part：□1Upper respiratory tract □2Lower respiratory tract
Date：|__|__|__|__|YEAR|__|__|MONTH|__|__|DAY Date：|__|__|:|__|__|
Result ： □1 Negative □2 Positive □3 Unidentified □4 Other ， please describe______________________

**Pulse oxygen saturation（without oxygen inhalation ）** □0Unexamined，reason_____
Date：|__|__|__|__|YEAR|__|__|MONTH|__|__|DAY
Measured Value： ______________%（Rest state）

**Whether develops into severe/critical type** □0 No evaluation Date|__|__|__|__|YEAR|__|__|MONTH|__|__|DAY
Whether develops into severe/critical type： □0 NO □1 YES
Date of developing into severe/critical type：|__|__|__|__|YEAR|__|__|MONTH|__|__|DAY
Date of developing into severe/critical type：|__|__|__|__|YEAR|__|__|MONTH|__|__|DAY

If any adverse events have occurred since the last visit, please fill in the “Adverse Events Form”. If there is medication combination, please fill in the “Previous/Combined medication Form”; If there is combined non-drug therapy, please fill in the “ Comined Non-Drug Therapy Form”; If there is an out-of-plan inspection, please fill in the “Out-Of-Plan Inspection Form”.

Date of Current Visit：|__|__|__|__|YEAR|__|__|MONTH|__|__|DAY

**Vital Signs**	□0Unexamined，reason________		
Date	|__|__|__|__|YEAR|__|__|MONTH|__|__|DAY		
Daily maximum temperature	|__|__|.|__|℃	Measurement method	□1 Axilla □2Oral □3 Anus □4 Ear □5 Unknown □6 Other，please describe________
Respiratory	|__|__|times/min	Pulse	|__|__|__|times/min
Blood pressure	|__|__|__|/|__|__|__| mmHg（Systolic/diastolic blood pressure）		

**Respiratory symptoms** □0Unexamined，reason________
Date：|__|__|__|__|YEAR|__|__|MONTH|__|__|DAY
Whether the subject has respiratory symptoms or not：□0 NO □1 NO，please describe：________
Result：□1 Normal □2 AbnormalNo clinical significance □3 AbnormalSignificant clinically

Blood Count □0Unexamined，reason________

Date：|__|__|__|__|YEAR|__|__|MONTH|__|__|DAY Normal range Number：|__|__|__|__|

Item	Result		Result Interpretation			Unexamined
	Measured Value	Unit	Normal	Abnormal: No clinical significance	Abnormal: Significant clinically	
Red Blood Cell（RBC）		□1 10^{12}/L □2Other：_____	□1	□2	□3	□0
White Blood Cell		□1 10^9/L □2Other：_____	□1	□2	□3	□0
Hemoglobin（Hb）		□1 g/L □2Other：_____	□1	□2	□3	□0
Platelet（PLT）		□1 10^9/L □2Other：_____	□1	□2	□3	□0
Neutrophil（ANC）		□1 10^9/L □2Other：_____	□1	□2	□3	□0
Lymphocyte（LY）		□1 10^9/L □2Other：_____	□1	□2	□3	□0
Monocyte（MO）		□1 10^9/L □2Other：_____	□1	□2	□3	□0
Eosinophil（EO）		□1 10^9/L □2Other：_____	□1	□2	□3	□0
Basophil（BA）		□1 10^9/L □2Other：_____	□1	□2	□3	□0

Blood Biochemistry □0Unexamined，reason________

Date|__|__|__|__|YEAR|__|__|MONTH|__|__|DAY Normal range Number：|__|__|__|__|

Item	Result		Result Interpretation			Unexamined
	Measured Value	Unit	Normal	Abnormal: No clinical significance	Abnormal: Significant clinically	
Glutamic-pyruvic transaminase（ALT）		□1 U/L □2Other：_____	□1	□2	□3	□0
Glutamic-oxalacetic transaminease（AST）		□1 U/L □2Other：_____	□1	□2	□3	□0
Total Bilirubin（TBIL）		□1 μmol/L □2Other：_____	□1	□2	□3	□0
Albumin（ALB）		□1 g/L □2Other：_____	□1	□2	□3	□0
γ- glutamyl transpeptidas（GGT）		□1 U/L □2Other：_____	□1	□2	□3	□0
Lactic dehydrogenase（LDH）		□1 U/L □2Other：_____	□1	□2	□3	□0
Creatine kinase（CK）		□1 U/L □2Other：_____	□1	□2	□3	□0
Creatine kinase isoenzyme（CK-MB）		□1 U/L □2Other：_____	□1	□2	□3	□0
Blood urea nitrogen（BUN）		□1 mmol/L □2Other：_____	□1	□2	□3	□0
Urea（Urea）		□1 mmol/L □2Other：_____	□1	□2	□3	□0
Serum creatinine（CR）		□1 μmol/L □2Other：_____	□1	□2	□3	□0

Urine Test □0Unexamined，reason________________________________
□1 Not Applicable

Date：|__|__|__|__|YEAR|__|__|MONTH|__|__|DAY Normal range Number：|__|__|__|__|

Item	Result		Result Interpretation			Unexamined
	Measured Value	Unit	Normal	Abnormal: No clinical significance	Abnormal: Significant clinically	
Urine Protein		□1Not Applicable □2Other：_____	□1	□2	□3	□0
Urine Glucose		□1 mmol/L □2Other：_____	□1	□2	□3	□0
Urine PH value		□1 Not Applicable □2Other：_____	□1	□2	□3	□0
Urine Erythrocyte		□1Not Applicable □2Other：_____	□1	□2	□3	□0
Urine Leukocyte		□1Not Applicable □2Other：_____	□1	□2	□3	□0
Urine Ketone		□1Not Applicable □2Other：_____	□1	□2	□3	□0

Coagulation Function □0Unexamined，reason________________________________

Date: |__|__|__|__|__|YEAR|__|__|MONTH|__|__|DAY Normal range Number: |__|__|__|__|

Item	Result		Result Interpretation			Unexamined
	Measured Value	Unit	Normal	Abnormal		
				No clinical significance	Significant clinically	
ProthrombinTime（PT）		□1 S □2Other: _____	□1	□2	□3	□0
Activated Partial Thromboplastin Time（APTT）		□1 S □2Other: _____	□1	□2	□3	□0
Thrombin Time（TT）		□1 S □2Other: _____	□1	□2	□3	□0
Fibrinogen（Fbg）		□1 g/L □2Other: _____	□1	□2	□3	□0
International Normalized Ratio（INR）		□1Not Applicable □2Other: _____	□1	□2	□3	□0

C-REACTION PROTEIN（CRP） □0Unexamined, reason________________________

Date: |__|__|__|__|__|YEAR|__|__|MONTH|__|__|DAY Normal range Number: |__|__|__|__|

Item	Result		Result Interpretation		
	Measured Value	Unit	Normal	Abnormal	
				No clinical significance	Significant clinically
C-REACTION PROTEIN（CRP）		□1 mg/L □2Other: _____	□1	□2	□3

Erythrocyte Sedimentation Rate（ESR） □0Unexamined, reason________________________

Date: |__|__|__|__|__|YEAR|__|__|MONTH|__|__|DAY Normal range Number: |__|__|__|__|

Item	Result		Result Interpretation		
	Measured Value	Unit	Normal	Abnormal	
				No clinical significance	Significant clinically
Erythrocyte Sedimentation Rate（ESR）		□1 mm/h □2Other:	□1	□2	□3

TNF-α □0Unexamined, reason________________________

Date: |__|__|__|__|__|YEAR|__|__|MONTH|__|__|DAY Normal range Number: |__|__|__|__|

Item	Result			Result Interpretation		
	Measured Value	Unit		Normal	Abnormal	
					No clinical significance	Significant clinically
TNF-α		□1 pg/mL	□2Other:	□1	□2	□3

IL-6 □0Unexamined, reason________________________

Date: |__|__|__|__|YEAR|__|__|MONTH|__|__|DAY				Normal range Number: |__|__|__|__|		
**Item**	**Result**			**Result Interpretation**		
					**Abnormal**	
IL-6	**Measured Value**	**Unit**		**Normal**	**No clinical significance**	**Significant clinically**
		□1 pg/mL	□2Other:	□1	□2	□3

**Nucleic Acid Test of 2019-nCov** □0 Unexamined, reason____________	
Sampling Part（mutiple choice）: □1Upper respiratory tract □2Lower respiratory tract	
Upper respiratory tract	Date: |__|__|__|__|YEAR|__|__|MONTH|__|__|DAY<br>Result: □1 Negative □2 Positive □3 Unidentified □4 Other, please describe ____________
Lower respiratory tract	Date: |__|__|__|__|YEAR|__|__|MONTH|__|__|DAY<br>Result: □1 Negative □2 Positive □3 Unidentified □4 Other, please describe ____________
Result	□1 Negative □2 Positive □3 Unidentified □4 Other, please describe ____________

**Pulse oxygen saturation（without oxygen inhalation）** □0Unexamined, reason_____
Date: |__|__|__|__|YEAR|__|__|MONTH|__|__|DAY
Measured Value: _______________%（Rest state）

**Whether develops into severe/critical type** □0 No Evaluation Date|__|__|__|__|YEAR|__|__|MONTH|__|__|DAY
Whether develops into severe/critical type: □0 NO □1 YES
Date of developing into severe/critical type: |__|__|__|__|YEAR|__|__|MONTH|__|__|DAY

If any adverse events have occurred since the last visit, please fill in the “Adverse Events Form”. If there is medication combination, please fill in the “Previous/Combined medication Form”; If there is combined non-drug therapy, please fill in the “ Comined Non-Drug Therapy Form”; If there is an out-of-plan inspection, please fill in the “Out-Of-Plan Inspection Form”.

Date of Current Visit: |__|__|__|__|YEAR|__|__|MONTH|__|__|DAY			
**Vital Signs**	□0Unexamined, reason________		
Date	|__|__|__|__|YEAR|__|__|MONTH|__|__|DAY		
Daily maximum temperature	|__|__|.|__|℃	Measurement method	□1 Axilla □2Oral □3 Anus □4 Ear □5 Unknown □6 Other, please describe________
Respiratory	|__|__|times/min	Pulse	|__|__|__|times/min
Blood pressure	|__|__|__|/|__|__|__| mmHg（Systolic/diastolic blood pressure）		

**physical examination**	□0 Unexamined, **reason**:________				
Date	|__|__|__|__|YEAR|__|__|MONTH|__|__|DAY				
Inspection Part	Result<br>1= Normal<br>2= Abnormal, no clinical significance<br>3= Abnormal, with clinical significance<br>0=Unexamined				If abnormal, please describe
General appearance	□1	□2	□3	□0	
Head	□1	□2	□3	□0	
Eyes	□1	□2	□3	□0	
Nose	□1	□2	□3	□0	
Larynx	□1	□2	□3	□0	
Neck and thyroid	□1	□2	□3	□0	
Skin	□1	□2	□3	□0	
Cardiovascular system	□1	□2	□3	□0	
Chest and lungs	□1	□2	□3	□0	
Abdomen	□1	□2	□3	□0	

Nervous system	□1	□2	□3	□0	
Musculoskeletal system	□1	□2	□3	□0	
Lymphatic system	□1	□2	□3	□0	

**Respiratory symptoms** □0Unexamined, reason________
Date: |__|__|__|__|YEAR|__|__|MONTH|__|__|DAY
Whether the subject has respiratory symptoms or not: □0 NO □1 NO, please describe: ________
Result: □1 Normal □2 AbnormalNo clinical significance □3 AbnormalSignificant clinically

Blood Count □0Unexamined, reason________

Date: \|__\|__\|__\|__\|YEAR\|__\|__\|MONTH\|__\|__\|DAY Normal range Number: \|__\|__\|__\|__\|

Item	Result		Result Interpretation			Unexamined
	Measured Value	Unit	Normal	Abnormal		
				No clinical significance	Significant clinically	
Red Blood Cell (RBC)		□1 10^{12}/L □2Other: _____	□1	□2	□3	□0
White Blood Cell		□1 10^{9}/L □2Other: _____	□1	□2	□3	□0
Hemoglobin (Hb)		□1 g/L □2Other: _____	□1	□2	□3	□0
Platelet (PLT)		□1 10^{9}/L □2Other: _____	□1	□2	□3	□0
Neutrophil (ANC)		□1 10^{9}/L □2Other: _____	□1	□2	□3	□0
Lymphocyte (LY)		□1 10^{9}/L □2Other: _____	□1	□2	□3	□0
Monocyte (MO)		□1 10^{9}/L □2Other: _____	□1	□2	□3	□0
Eosinophil (EO)		□1 10^{9}/L □2Other: _____	□1	□2	□3	□0
Basophil (BA)		□1 10^{9}/L □2Other: _____	□1	□2	□3	□0

Blood Biochemistry □0Unexamined, reason________

Date\|__\|__\|__\|__\|YEAR\|__\|__\|MONTH\|__\|__\|DAY Normal range Number: \|__\|__\|__\|__\|

Item	Result		Result Interpretation			Unexamined
	Measured Value	Unit	Normal	Abnormal		
				No clinical significance	Significant clinically	
Glutamic-pyruvic transaminase (ALT)		□1 U/L □2Other: _____	□1	□2	□3	□0
Glutamic-oxalacetic transaminease (AST)		□1 U/L □2Other: _____	□1	□2	□3	□0

Total Bilirubin（TBIL）		□₁ μmol/L □₂Other: _____	□1	□2	□3	□0
Albumin（ALB）		□1 g/L □2Other: _____	□1	□2	□3	□0
γ- glutamyl transpeptidas（GGT）		□1 U/L □2Other: _____	□1	□2	□3	□0
Lactic dehydrogenase（LDH）		□1 U/L □2Other: _____	□1	□2	□3	□0
Creatine kinase（CK）		□1 U/L □2Other: _____	□1	□2	□3	□0
Creatine kinase isoenzyme（CK-MB）		□1 U/L □2Other: _____	□1	□2	□3	□0
Blood urea nitrogen（BUN）		□1 mmol/L □2Other: _____	□1	□2	□3	□0
Urea（Urea）		□1 mmol/L □2Other: _____	□1	□2	□3	□0
Serum creatinine（CR）		□1 μmol/L □2Other: _____	□1	□2	□3	□0

Urine Test □0Unexamined, reason________________

Date: |__|__|__|__|YEAR|__|__|MONTH|__|__|DAY Normal range Number: |__|__|__|__|

Item	Result		Result Interpretation			Unexamined
	Measured Value	Unit	Normal	Abnormal		
				No clinical significance	Significant clinically	
Urine Protein		□1Not Applicable □2Other: _____	□1	□2	□3	□0
Urine Glucose		□1 mmol/L □2Other: _____	□1	□2	□3	□0
Urine PH value		□1 Not Applicable □2Other: _____	□1	□2	□3	□0
Urine Erythrocyte		□1Not Applicable □2Other: _____	□1	□2	□3	□0
Urine Leukocyte		□1Not Applicable □2Other: _____	□1	□2	□3	□0
Urine Ketone		□1Not Applicable □2Other: _____	□1	□2	□3	□0

Coagulation Function □0Unexamined, reason________________

Date: |__|__|__|__|YEAR|__|__|MONTH|__|__|DAY Normal range Number: |__|__|__|__|

Item	Result		Result Interpretation			Unexamined
	Measured Value	Unit	Normal	Abnormal		
				No clinical significance	Significant clinically	
ProthrombinTime（PT）		□1 S □2Other: _____	□1	□2	□3	□0
Activated Partial Thromboplastin Time		□1 S □2Other: _____	□1	□2	□3	□0

(APTT)						
Thrombin Time（TT）		□1 S □2Other: _____	□1	□2	□3	□0
Fibrinogen（Fbg）		□1 g/L □2Other: _____	□1	□2	□3	□0
International Normalized Ratio（INR）		□1Not Applicable □2Other: _____	□1	□2	□3	□0

C-REACTION PROTEIN（CRP） □0Unexamined, reason________________

Date: |__|__|__|__|YEAR|__|__|MONTH|__|__|DAY Normal range Number: |__|__|__|__|

Item	Result		Result Interpretation		
				Abnormal	
C-REACTION PROTEIN（CRP）	Measured Value	Unit	Normal	No clinical significance	Significant clinically
		□1 mg/L □2Other: _____	□1	□2	□3

Erythrocyte Sedimentation Rate（ESR） □0Unexamined, reason________________

Date: |__|__|__|__|YEAR|__|__|MONTH|__|__|DAY Normal range Number: |__|__|__|__|

Item	Result		Result Interpretation		
				Abnormal	
Erythrocyte Sedimentation Rate（ESR）	Measured Value	Unit	Normal	No clinical significance	Significant clinically
		□1 mm/h □2Other:	□1	□2	□3

TNF-α □0Unexamined, reason________________

Date: |__|__|__|__|YEAR|__|__|MONTH|__|__|DAY Normal range Number: |__|__|__|__|

Item	Result			Result Interpretation		
					Abnormal	
TNF-α	Measured Value	Unit		Normal	No clinical significance	Significant clinically
		□1 pg/mL	□2Other:	□1	□2	□3

IL-6 □0Unexamined, reason________________

Date: |__|__|__|__|YEAR|__|__|MONTH|__|__|DAY Normal range Number: |__|__|__|__|

Item	Result		Result Interpretation		
				Abnormal	
IL-6	Measured Value	Unit	Normal	No clinical significance	Significant clinically

		□1 pg/mL	□2Other:	□1	□2	□3

**Nucleic Acid Test of 2019-nCov** □0 Unexamined, reason________________<br>□1 Not Applicable
Sampling Part: □1Upper respiratory tract □2Lower respiratory tract
Date: |__|__|__|__|YEAR|__|__|MONTH|__|__|DAY Date: |__|__|:|__|__|
Result : □1 Negative □2 Positive □3 Unidentified □4 Other , please describe________________

**Pulse oxygen saturation（without oxygen inhalation ）** □0Unexamined, reason_____
Date: |__|__|__|__|YEAR|__|__|MONTH|__|__|DAY
Measured Value: ____________%（Rest state）

**Routine 12 Leads Electrocardiogram** □0Unexamined, reason________________
Date: |__|__|__|__|YEAR|__|__|MONTH|__|__|DAY
Result: □1 Normal □2 AbnormalNo clinical significance □3 AbnormalSignificant clinically
If“Abnormal”, please describe: ________________________________

**Urine or serum HCG (Only applicable to women of childbearing age)** □0Unexamined, reason________________<br>□1Not Applicable（Man or woman who isn’t able to reproduce）
Date: |__|__|__|__|YEAR|__|__|MONTH|__|__|DAY
Sample: □1 Blood □2 Urine
Result: □1 Negative □2 Positive

**Chest CT** □0Unexamined, reason________________
Date: |__|__|__|__|YEAR|__|__|MONTH|__|__|DAY
Result: □1 Normal □2 Abnormal,no clinical significance □3 Abnormal, significant clinically_
If abnormal, please describe ________________
Wheather chest CT lesion absorbed≥50% than baseline??<br>□0 NO □1 YES □2 Unidentified □3 Unknown<br>□4 Other, please describe: ________________

**Whether develops into severe/critical type** □0 No evaluation Date|__|__|__|__|YEAR|__|__|MONTH|__|__|DAY
Whether develops into severe/critical type： □0 NO □1 YES
Date of developing into severe/critical type： |__|__|__|__|YEAR|__|__|MONTH|__|__|DAY

If any adverse events have occurred since the last visit, please fill in the "Adverse Events Form". If there is medication combination, please fill in the "Previous/Combined medication Form"; If there is combined non-drug therapy, please fill in the " Comined Non-Drug Therapy Form"; If there is an out-of-plan inspection, please fill in the "Out-Of-Plan Inspection Form".

End of the Treatment
Whether the subject finished all the treatment according to the protocol? □0 NO □1 YES
End Date: |__|__|__|__|YEAR|__|__|MONTH|__|__|DAY
If the patient exit ahead of schedule, please choose the most important reason (One-Choice):<br>□1 Disease progression occurred during the study and changes in treatment were required after comprehensive assessment by the investigators.<br>□2 Adverse events lead to an exacerbation of the disease<br>□3 The subject is reluctant to continue using the study drug.<br>□4 Pregnancy<br>□5 Loss to follow-up<br>□6 Death<br>□7 Subject withdraws the Informed Consent<br>□8 Study suspension<br>□9 Other, please describe: ____________________<br>____________________<br><br>Notes (Please record other matter which needs additional clarification):<br>____________________<br>____________________<br>____________________

Type of visit	□1 Telephone follow-up □2 Hospital follow-up
**Date of Current Visit：**	|__|__|__|__|YEAR|__|__|MONTH|__|__|DAY

If the subject has been discharged from the hospital, the following contents may not be carried out, please leave blank; If the subject is not discharged, please conduct the following examinations.

**Vital Signs**	□0Unexamined，reason________		
Date	|__|__|__|__|YEAR|__|__|MONTH|__|__|DAY		
Daily maximum temperature	|__|__|.|__|℃	Measurement method	□1 Axilla □2Oral □3 Anus □4 Ear □5 Unknown □6 Other，please describe________
Respiratory	|__|__|times/min	Pulse	|__|__|__|times/min
Blood pressure	|__|__|__|/|__|__|__| mmHg（Systolic/diastolic blood pressure）		

**physical examination**	□0 Unexamined**, reason**:________				
Date	|__|__|__|__|YEAR|__|__|MONTH|__|__|DAY				
Inspection Part	Result<br>1= Normal<br>2= Abnormal, no clinical significance<br>3= Abnormal, with clinical significance<br>0=Unexamined				If abnormal，please describe
General appearance	□1	□2	□3	□0	
Head	□1	□2	□3	□0	
Eyes	□1	□2	□3	□0	
Nose	□1	□2	□3	□0	
Larynx	□1	□2	□3	□0	
Neck and thyroid	□1	□2	□3	□0	
Skin	□1	□2	□3	□0	
Cardiovascular system	□1	□2	□3	□0	

Chest and lungs	□1	□2	□3	□0	
Abdomen	□1	□2	□3	□0	
Nervous system	□1	□2	□3	□0	
Musculoskeletal system	□1	□2	□3	□0	
Lymphatic system	□1	□2	□3	□0	

**Respiratory symptoms** □0Unexamined, reason________________________________
Date: |__|__|__|__|YEAR|__|__|MONTH|__|__|DAY
Whether the subject has respiratory symptoms or not: □0 NO □1 NO, please describe: _____ ________________________________
Result: □1 Normal □2 AbnormalNo clinical significance □3 AbnormalSignificant clinically

Blood Count □0Unexamined, reason________________________________

Date: \|__\|__\|__\|__\|YEAR\|__\|__\|MONTH\|__\|__\|DAY Normal range Number: \|__\|__\|__\|__\|

Item	Result		Result Interpretation			Unexamined
				Abnormal		
	Measured Value	Unit	Normal	No clinical significance	Significant clinically	
Red Blood Cell（RBC）		□1 10^{12}/L □2Other: _____	□1	□2	□3	□0
White Blood Cell		□1 10^9/L □2Other: _____	□1	□2	□3	□0
Hemoglobin（Hb）		□1 g/L □2Other: _____	□1	□2	□3	□0
Platelet（PLT）		□1 10^9/L □2Other: _____	□1	□2	□3	□0
Neutrophil（ANC）		□1 10^9/L □2Other: _____	□1	□2	□3	□0
Lymphocyte（LY）		□1 10^9/L □2Other: _____	□1	□2	□3	□0
Monocyte（MO）		□1 10^9/L □2Other: _____	□1	□2	□3	□0
Eosinophil（EO）		□1 10^9/L □2Other: _____	□1	□2	□3	□0
Basophil（BA）		□1 10^9/L □2Other: _____	□1	□2	□3	□0

Blood Biochemistry □0Unexamined, reason________________________________

Date\|__\|__\|__\|__\|YEAR\|__\|__\|MONTH\|__\|__\|DAY Normal range Number: \|__\|__\|__\|__\|

Item	Result		Result Interpretation			Unexamined
				Abnormal		
	Measured Value	Unit	Normal	No clinical significanc	Significant clinically	

				e		
Glutamic-pyruvic transaminase（ALT）		□1 U/L □2Other: _____	□1	□2	□3	□0
Glutamic-oxalacetic transaminease（AST）		□1 U/L □2Other: _____	□1	□2	□3	□0
Total Bilirubin（TBIL）		□1 μmol/L □2Other: _____	□1	□2	□3	□0
Albumin（ALB）		□1 g/L □2Other: _____	□1	□2	□3	□0
γ- glutamyl transpeptidas（GGT）		□1 U/L □2Other: _____	□1	□2	□3	□0
Lactic dehydrogenase（LDH）		□1 U/L □2Other: _____	□1	□2	□3	□0
Creatine kinase（CK）		□1 U/L □2Other: _____	□1	□2	□3	□0
Creatine kinase isoenzyme（CK-MB）		□1 U/L □2Other: _____	□1	□2	□3	□0
Blood urea nitrogen（BUN）		□1 mmol/L □2Other: _____	□1	□2	□3	□0
Urea（Urea）		□1 mmol/L □2Other: _____	□1	□2	□3	□0
Serum creatinine（CR）		□1 μmol/L □2Other: _____	□1	□2	□3	□0

Urine Test □0Unexamined, reason________________________

Date：\|__\|__\|__\|__\|YEAR\|__\|__\|MONTH\|__\|__\|DAY Normal range Number：\|__\|__\|__\|__\|

Item	Result		Result Interpretation			Unexamined
				Abnormal		
	Measured Value	Unit	Normal	No clinical significance	Significant clinically	
Urine Protein		□1Not Applicable □2Other: _____	□1	□2	□3	□0
Urine Glucose		□1 mmol/L □2Other: _____	□1	□2	□3	□0
Urine PH value		□1 Not Applicable □2Other: _____	□1	□2	□3	□0
Urine Erythrocyte		□1Not Applicable □2Other: _____	□1	□2	□3	□0
Urine Leukocyte		□1Not Applicable □2Other: _____	□1	□2	□3	□0
Urine Ketone		□1Not Applicable □2Other: _____	□1	□2	□3	□0

Coagulation Function □0Unexamined, reason________________________

Date：\|__\|__\|__\|__\|YEAR\|__\|__\|MONTH\|__\|__\|DAY Normal range Number：\|__\|__\|__\|__\|

Item	Result		Result Interpretation		Unexamined
	Measured	Unit	Norm	Abnormal	

	Value		al	No clinical significance	Significant clinically	
ProthrombinTime（PT）		□1 S □2Other: _____	□1	□2	□3	□0
Activated Partial Thromboplastin Time（APTT）		□1 S □2Other: _____	□1	□2	□3	□0
Thrombin Time（TT）		□1 S □2Other: _____	□1	□2	□3	□0
Fibrinogen（Fbg）		□1 g/L □2Other: _____	□1	□2	□3	□0
International Normalized Ratio（INR）		□1Not Applicable □2Other: _____	□1	□2	□3	□0

C-REACTION PROTEIN（CRP） □0Unexamined，reason____________________

Date：\|__\|__\|__\|__\|YEAR\|__\|__\|MONTH\|__\|__\|DAY Normal range Number：\|__\|__\|__\|__\|

Item	Result		Result Interpretation		
				Abnormal	
	Measured Value	Unit	Normal	No clinical significance	Significant clinically
C-REACTION PROTEIN（CRP）		□1 mg/L □2Other: _____	□1	□2	□3

Erythrocyte Sedimentation Rate（ESR） □0Unexamined，reason____________________

Date：\|__\|__\|__\|__\|YEAR\|__\|__\|MONTH\|__\|__\|DAY Normal range Number：\|__\|__\|__\|__\|

Item	Result		Result Interpretation		
				Abnormal	
	Measured Value	Unit	Normal	No clinical significance	Significant clinically
Erythrocyte Sedimentation Rate（ESR）		□1 mm/h □2Other:	□1	□2	□3

TNF-α □0Unexamined，reason____________________

Date：\|__\|__\|__\|__\|YEAR\|__\|__\|MONTH\|__\|__\|DAY Normal range Number：\|__\|__\|__\|__\|

Item	Result		Result Interpretation		
				Abnormal	
	Measured Value	Unit	Normal	No clinical significance	Significant clinically
TNF-α		□1 pg/mL □2Other:	□1	□2	□3

IL-6 □0Unexamined，reason____________________

Date：\|__\|__\|__\|__\|YEAR\|__\|__\|MONTH\|__\|__\|DAY Normal range Number：\|__\|__\|__\|__\|

Item	Result		Result Interpretation	
	Measured	Unit	Norm	Abnormal

IL-6	Value			al	No clinical significance	Significant clinically
		□1 pg/mL	□2Other：	□1	□2	□3

**Urine or serum HCG（Only applicable to women of childbearing age）** □0Unexamined，reason________________ □1Not Applicable（Man or woman who isn't able to reproduce）
Date：|__|__|__|__|YEAR|__|__|MONTH|__|__|DAY
Sample：□1 Blood □2 Urine
Result：□1 Negative □2 Positive

**Routine 12 Leads Electrocardiogram** □0Unexamined，reason________________
Date：|__|__|__|__|YEAR|__|__|MONTH|__|__|DAY
Result：□1 Normal □2 AbnormalNo clinical significance □3 AbnormalSignificant clinically
If"Abnormal"，please describe：________________

**Chest CT** □0Unexamined，reason________________
Date：|__|__|__|__|YEAR|__|__|MONTH|__|__|DAY
Result：________________
Wheather chest CT lesion absorbed≥50% than baseline?？ □0 NO □1 YES □2 Unidentified □3 Unknown □4 Other，please describe：________________

**Nucleic Acid Test of 2019-nCov** □0 Unexamined，reason________________ □1 Not Applicable
Sampling Part：□1Upper respiratory tract □2Lower respiratory tract
Date：|__|__|__|__|YEAR|__|__|MONTH|__|__|DAY Date：|__|__|:|__|__|
Result ：□1 Negative □2 Positive □3 Unidentified □4 Other ，please describe________________

**Pulse oxygen saturation（without oxygen inhalation ）** □0Unexamined，reason_____

Date：|__|__|__|__|YEAR|__|__|MONTH|__|__|DAY
Measured Value：_______________%（Rest state）

**Whether develops into severe/critical type** □0 No evaluation Date|__|__|__|__|YEAR|__|__|MONTH|__|__|DAY
Whether develops into severe/critical type： □0 NO □1 YES
Date of developing into severe/critical type：|__|__|__|__|YEAR|__|__|MONTH|__|__|DAY

If any adverse events have occurred since the last visit, please fill in the "Adverse Events Form". If there is medication combination, please fill in the "Previous/Combined medication Form"; If there is combined non-drug therapy, please fill in the " Comined Non-Drug Therapy Form"; If there is an out-of-plan inspection, please fill in the "Out-Of-Plan Inspection Form".

Type of visit	□1 Telephone follow-up □2 Hospital follow-up
**Date of Current Visit:** |__|__|__|__|YEAR|__|__|MONTH|__|__|DAY	

If the subject has been discharged from the hospital, the following contents may not be carried out, please leave blank; If the subject is not discharged, please conduct the following examinations.

**Vital Signs**	□0Unexamined, reason________________		
Date	|__|__|__|__|YEAR|__|__|MONTH|__|__|DAY		
Daily maximum temperature	|__|__|.|__|℃	Measurement method	□1 Axilla □2Oral □3 Anus □4 Ear □5 Unknown □6 Other, please describe_______
Respiratory	|__|__|times/min	Pulse	|__|__|__|times/min
Blood pressure	|__|__|__|/|__|__|__| mmHg (Systolic/diastolic blood pressure)		

**physical examination**	□0 Unexamined, **reason**:________________				
Date	|__|__|__|__|YEAR|__|__|MONTH|__|__|DAY				
Inspection Part	Result<br>1= Normal<br>2= Abnormal, no clinical significance<br>3= Abnormal, with clinical significance<br>0=Unexamined				If abnormal, please describe
General appearance	□1	□2	□3	□0	
Head	□1	□2	□3	□0	
Eyes	□1	□2	□3	□0	
Nose	□1	□2	□3	□0	
Larynx	□1	□2	□3	□0	
Neck and thyroid	□1	□2	□3	□0	
Skin	□1	□2	□3	□0	

Cardiovascular system	□1	□2	□3	□0	
Chest and lungs	□1	□2	□3	□0	
Abdomen	□1	□2	□3	□0	
Nervous system	□1	□2	□3	□0	
Musculoskeletal system	□1	□2	□3	□0	
Lymphatic system	□1	□2	□3	□0	

Respiratory symptoms □0Unexamined, reason________________

Date: |__|__|__|__|YEAR|__|__|MONTH|__|__|DAY

Whether the subject has respiratory symptoms or not: □0 NO □1 NO, please describe: _____

____

Result: □1 Normal □2 AbnormalNo clinical significance □3 AbnormalSignificant clinically

Blood Count □0Unexamined, reason________________

Date: |__|__|__|__|YEAR|__|__|MONTH|__|__|DAY Normal range Number: |__|__|__|__|

Item	Result		Result Interpretation			Unexamined
				Abnormal		
	Measured Value	Unit	Normal	No clinical significance	Significant clinically	
Red Blood Cell (RBC)		□1 10^{12}/L □2Other: _____	□1	□2	□3	□0
White Blood Cell		□1 10^9/L □2Other: _____	□1	□2	□3	□0
Hemoglobin (Hb)		□1 g/L □2Other: _____	□1	□2	□3	□0
Platelet (PLT)		□1 10^9/L □2Other: _____	□1	□2	□3	□0
Neutrophil (ANC)		□1 10^9/L □2Other: _____	□1	□2	□3	□0
Lymphocyte (LY)		□1 10^9/L □2Other: _____	□1	□2	□3	□0
Monocyte (MO)		□1 10^9/L □2Other: _____	□1	□2	□3	□0
Eosinophil (EO)		□1 10^9/L □2Other: _____	□1	□2	□3	□0
Basophil (BA)		□1 10^9/L □2Other: _____	□1	□2	□3	□0

Blood Biochemistry □0Unexamined, reason________________

Date|__|__|__|__|YEAR|__|__|MONTH|__|__|DAY Normal range Number: |__|__|__|__|

Item	Result	Result Interpretation	Unexa

	Measured Value	Unit	Normal	Abnormal		mined
				No clinical significance	Significant clinically	
Glutamic-pyruvic transaminase（ALT）		□1 U/L □2Other: _____	□1	□2	□3	□0
Glutamic-oxalacetic transaminease（AST）		□1 U/L □2Other: _____	□1	□2	□3	□0
Total Bilirubin（TBIL）		□1 μmol/L □2Other: _____	□1	□2	□3	□0
Albumin（ALB）		□1 g/L □2Other: _____	□1	□2	□3	□0
γ- glutamyl transpeptidas（GGT）		□1 U/L □2Other: _____	□1	□2	□3	□0
Lactic dehydrogenase（LDH）		□1 U/L □2Other: _____	□1	□2	□3	□0
Creatine kinase（CK）		□1 U/L □2Other: _____	□1	□2	□3	□0
Creatine kinase isoenzyme（CK-MB）		□1 U/L □2Other: _____	□1	□2	□3	□0
Blood urea nitrogen（BUN）		□1 mmol/L □2Other: _____	□1	□2	□3	□0
Urea（Urea）		□1 mmol/L □2Other: _____	□1	□2	□3	□0
Serum creatinine（CR）		□1 μmol/L □2Other: _____	□1	□2	□3	□0

Urine Test □0Unexamined, reason________________

Date: |__|__|__|__|YEAR|__|__|MONTH|__|__|DAY Normal range Number: |__|__|__|__|

Item	Result		Result Interpretation			Unexamined
			Normal	Abnormal		
	Measured Value	Unit		No clinical significance	Significant clinically	
Urine Protein		□1Not Applicable □2Other: _____	□1	□2	□3	□0
Urine Glucose		□1 mmol/L □2Other: _____	□1	□2	□3	□0
Urine PH value		□1 Not Applicable □2Other: _____	□1	□2	□3	□0
Urine Erythrocyte		□1Not Applicable □2Other: _____	□1	□2	□3	□0
Urine Leukocyte		□1Not Applicable □2Other: _____	□1	□2	□3	□0
Urine Ketone		□1Not Applicable □2Other: _____	□1	□2	□3	□0

Coagulation Function □0Unexamined, reason________________

Date: |__|__|__|__|YEAR|__|__|MONTH|__|__|DAY Normal range Number: |__|__|__|__|

Item	Result		Result Interpretation			Unexamined
	Measured Value	Unit	Normal	Abnormal		
				No clinical significance	Significant clinically	
ProthrombinTime（PT）		□1 S　□2Other: _____	□1	□2	□3	□0
Activated Partial Thromboplastin Time（APTT）		□1 S　□2Other: _____	□1	□2	□3	□0
Thrombin Time（TT）		□1 S　□2Other: _____	□1	□2	□3	□0
Fibrinogen（Fbg）		□1 g/L　□2Other: _____	□1	□2	□3	□0
International Normalized Ratio（INR）		□1Not Applicable　□2Other: _____	□1	□2	□3	□0

C-REACTION PROTEIN（CRP）　□0Unexamined，reason________________________

Date：\|__\|__\|__\|__\|YEAR\|__\|__\|MONTH\|__\|__\|DAY　Normal range Number：\|__\|__\|__\|__\|

Item	Result		Result Interpretation		
C-REACTION PROTEIN（CRP）	Measured Value	Unit	Normal	Abnormal	
				No clinical significance	Significant clinically
		□1 mg/L　□2Other: _____	□1	□2	□3

Erythrocyte Sedimentation Rate（ESR）　□0Unexamined，reason________________________

Date：\|__\|__\|__\|__\|YEAR\|__\|__\|MONTH\|__\|__\|DAY　Normal range Number：\|__\|__\|__\|__\|

Item	Result		Result Interpretation		
Erythrocyte Sedimentation Rate（ESR）	Measured Value	Unit	Normal	Abnormal	
				No clinical significance	Significant clinically
		□1 mm/h　□2Other:	□1	□2	□3

TNF-α　□0Unexamined，reason________________________

Date：\|__\|__\|__\|__\|YEAR\|__\|__\|MONTH\|__\|__\|DAY　Normal range Number：\|__\|__\|__\|__\|

Item	Result			Result Interpretation		
TNF-α	Measured Value	Unit		Normal	Abnormal	
					No clinical significance	Significant clinically
		□1 pg/mL	□2Other:	□1	□2	□3

IL-6　□0Unexamined，reason________________________

Date：\|__\|__\|__\|__\|YEAR\|__\|__\|MONTH\|__\|__\|DAY　Normal range Number：\|__\|__\|__\|__\|

Item	Result	Result Interpretation

IL-6	**Measured Value**	**Unit**		**Normal**	**Abnormal**	
					**No clinical significance**	**Significant clinically**
		□1 pg/mL	□2Other:	□1	□2	□3

**Urine or serum HCG（Only applicable to women of childbearing age）** □0Unexamined, reason________ □1Not Applicable（Man or woman who isn't able to reproduce）
Date: |__|__|__|__|YEAR|__|__|MONTH|__|__|DAY
Sample: □1 Blood □2 Urine
Result: □1 Negative □2 Positive

**Routine 12 Leads Electrocardiogram** □0Unexamined, reason________
Date: |__|__|__|__|YEAR|__|__|MONTH|__|__|DAY
Result: □1 Normal □2 Abnormal No clinical significance □3 Abnormal Significant clinically
If "Abnormal", please describe: ________

**Nucleic Acid Test of 2019-nCov** □0 Unexamined, reason________ □1 Not Applicable
Sampling Part: □1Upper respiratory tract □2Lower respiratory tract
Date: |__|__|__|__|YEAR|__|__|MONTH|__|__|DAY Date: |__|__|:|__|__|
Result: □1 Negative □2 Positive □3 Unidentified □4 Other, please describe________

**Pulse oxygen saturation（without oxygen inhalation ）** □0Unexamined, reason________
Date: |__|__|__|__|YEAR|__|__|MONTH|__|__|DAY
Measured Value: ________%（Rest state）

**Chest CT** □0Unexamined, reason________
Date: |__|__|__|__|YEAR|__|__|MONTH|__|__|DAY
Result: ________

____
Wheather chest CT lesion absorbed≥50% than baseline?? □0 NO □1 YES □2 Unidentified □3 Unknown □4 Other, please describe: ____________________

**Whether develops into severe/critical type** □0 No evaluation Date|__|__|__|__|YEAR|__|__|MONTH|__|__|DAY
Whether develops into severe/critical type: □0 NO □1 YES
Date of developing into severe/critical type: |__|__|__|__|YEAR|__|__|MONTH|__|__|DAY

If any adverse events have occurred since the last visit, please fill in the "Adverse Events Form". If there is medication combination, please fill in the "Previous/Combined medication Form"; If there is combined non-drug therapy, please fill in the " Comined Non-Drug Therapy Form"; If there is an out-of-plan inspection, please fill in the "Out-Of-Plan Inspection Form".

Does the subject use the drug studied? ☐0 NO ☐1 YES ☐2 Not applicable（Control Group）								
**Situation of Drug Usage**								
**Days of taking the drug**	**Whether use the drug**	**Usage Date**	**Planned daily dosage (mg)**	**Actual daily dosage (mg)**	**Frequency (1)**	**Delivery (2)**	**State the reason in case of dose adjustment or suspension**	**Please explain in case of missed、overdose or other situation**
**Day__**	☐0 NO<br>☐1 YES	|__|__|__|__|YEAR<br>|__|__|MONTH<br>|__|__|DAY	|__|__|__|__|	|__|__|__|__|	|__| _______	|__| _________	☐1 AE，Number__<br>☐2 Other, _____	
**Day__**	☐0 NO<br>☐1 YES	|__|__|__|__|YEAR<br>|__|__|MONTH<br>|__|__|DAY	|__|__|__|__|	|__|__|__|__|	|__| ________	|__| _________	☐1 AE，Number__<br>☐2 Other, _____	
**Day__**	☐0 NO<br>☐1 YES	|__|__|__|__|YEAR<br>|__|__|MONTH<br>|__|__|DAY	|__|__|__|__|	|__|__|__|__|	|__| _________	|__| _________	☐1 AE，Number__<br>☐2 Other, _____	
**Day__**	☐0 NO<br>☐1 YES	|__|__|__|__|YEAR<br>|__|__|MONTH<br>|__|__|DAY	|__|__|__|__|	|__|__|__|__|	|__| _________	|__| _________	☐1 AE，Number__<br>☐2 Other, _____	
**Day__**	☐0 NO<br>☐1 YES	|__|__|__|__|YEAR<br>|__|__|MONTH<br>|__|__|DAY	|__|__|__|__|	|__|__|__|__|	|__| _________	|__| _________	☐1 AE，Number__<br>☐2 Other, _____	
**(1) Frequency: 1= three times a day 2= twice a day 3= once a day 4= unknown 5= others, please specify in the line**<br>**(2) Delivery: 1= not applicable 2= dose adjustment 3= missed 4= overdose 5= suspended administration 6= others, please specify in the line**								

Does the subject use the drug studied? □0 NO □1 YES □2 Not applicable（Control Group）								
**Situation of Drug Usage**								
**Days of taking the drug**	**Whether use the drug**	**Usage Date**	**Planned daily dosage (mg)**	**Actual daily dosage (mg)**	**Frequency (1)**	**Delivery (2)**	**State the reason in case of dose adjustment or suspension**	**Please explain in case of missed、overdose or other situation**
**Day__**	□0 NO<br>□1 YES	|__|__|__|__|YEAR<br>|__|__|MONTH<br>|__|__|DAY	|__|__|__|__|	|__|__|__|__|	|__| ________	|__| ________	□1 AE，Number__<br>□2 Other, _____	
**Day__**	□0 NO<br>□1 YES	|__|__|__|__|YEAR<br>|__|__|MONTH<br>|__|__|DAY	|__|__|__|__|	|__|__|__|__|	|__| ________	|__| ________	□1 AE，Number__<br>□2 Other, _____	
**Day__**	□0 NO<br>□1 YES	|__|__|__|__|YEAR<br>|__|__|MONTH<br>|__|__|DAY	|__|__|__|__|	|__|__|__|__|	|__| ________	|__| ________	□1 AE，Number__<br>□2 Other, _____	
**Day__**	□0 NO<br>□1 YES	|__|__|__|__|YEAR<br>|__|__|MONTH<br>|__|__|DAY	|__|__|__|__|	|__|__|__|__|	|__| ________	|__| ________	□1 AE，Number__<br>□2 Other, _____	
**Day__**	□0 NO<br>□1 YES	|__|__|__|__|YEAR<br>|__|__|MONTH<br>|__|__|DAY	|__|__|__|__|	|__|__|__|__|	|__| ________	|__| ________	□1 AE，Number__<br>□2 Other, _____	

(1) Frequency: 1= three times a day 2= twice a day 3= once a day 4= unknown 5= others, please specify in the line<br>(2) Delivery: 1= not applicable 2= dose adjustment 3= missed 4= overdose 5= suspended administration 6= others, please specify in the line

Did the subject experience any adverse event? □0 NO □1 YES, please fill in refer to the code at the bottom of the form

Adverse Events?

NO.	Adverse Event	Date Of begining	Sustained at the end of the study?		CTCAE Level (1)	Taken Measurements		Relationship with the studie	AE Outcome (5)	SAE or Not?	
			YES	NO→Date of End		Measurement for the studied drug (2)	Other measurement (3)			No	YES, the specific SAE type (6)
1		|__|__|__|__|YEAR|__|__|MONTH|__|__|DAY	□	|__|__|__|__|YEAR|__|__|MONTH|__|__|DAY	|__|	|__| ________	|__| ________________	|__|	|__|	□	|__|
2		|__|__|__|__|YEAR|__|__|MONTH|__|__|DAY	□	|__|__|__|__|YEAR|__|__|MONTH|__|__|DAY	|__|	|__| ________	|__| ________________	|__|	|__|	□	|__|
3		|__|__|__|__|YEAR|__|__|MONTH|__|__|DAY	□	|__|__|__|__|YEAR|__|__|MONTH|__|__|DAY	|__|	|__| ________	|__| ________________	|__|	|__|	□	|__|
4		|__|__|__|__|YEAR|__|__|MONTH|__|__|DAY	□	|__|__|__|__|YEAR|__|__|MONTH|__|__|DAY	|__|	|__| ________	|__| ________________	|__|	|__|	□	|__|

(1) CTCAE Level	(2) Measurement for the studied drug	(3) Other measurements	(4) Relationship with the studied drug	(5) AE outcome	(6) Specific SAE type
1=level 1<br>2=level 2<br>3=level 3<br>4=level 4<br>5=level 5	1=Maintenance of current treatment<br>2=Suspension<br>3=Termination<br>4=Reduce the dose<br>5=Not applicable<br>6= Others, please specify in thel line	1= No<br>2= Combined medication，fill in the Combined Medication Form<br>3= Combined with non-drug therapy，fill in the Combined non-drug therapy Form<br>4= Others, please specify in thel line	1=Certainly related<br>2=Probably related<br>3=Possibly related<br>4= Possibly non-related<br>5= Certainly non- related<br>6= Remain to be evaluated<br>7=Unable to evaluate	1 = unchanged<br>2=deterioration<br>3= recovery/healing<br>4=improvement/recovery<br>5= recovery with sequelae<br>6 = death<br>7 = unknown	1= life threatening<br>2= cause death<br>3= require hospitalization or extend the current stay<br>4= cause permanent or significant disability or dysfunction<br>5= causes congenital malformation/birth defects<br>6= other important medical events

Does the subject have previous/combined medication?

□0 **NO** □1 **YES，please fill in the form below refer to the code at the bottom of the table.**

Previous/Combined medication

NO.	Drug Name (generic name)	Indications (1)	Indications Notes	single dose	Dose Unit (2)	Dosing frequency (3)	administration route (4)	Date of beginning	Sustain at the end of the study?	
									Yes	NO→Date of end
1		|__|			|__|	|__|	|__|	|__|__|__|__|YEAR|__|__|MONTH|__|__|DAY	□	|__|__|__|__|YEAR|__|__|MONTH|__|__|DAY
2		|__|			|__|	|__|	|__|	|__|__|__|__|YEAR|__|__|MONTH|__|__|DAY	□	|__|__|__|__|YEAR|__|__|MONTH|__|__|DAY
3		|__|			|__|	|__|	|__|	|__|__|__|__|YEAR|__|__|MONTH|__|__|DAY	□	|__|__|__|__|YEAR|__|__|MONTH|__|__|DAY
4		|__|			|__|	|__|	|__|	|__|__|__|__|YEAR|__|__|MONTH|__|__|DAY	□	|__|__|__|__|YEAR|__|__|MONTH|__|__|DAY
5		|__|			|__|	|__|	|__|	|__|__|__|__|YEAR|__|__|MONTH|__|__|DAY	□	|__|__|__|__|YEAR|__|__|MONTH|__|__|DAY

(1) Indications	(2) dosage Unit	(3) Dosing frequency	(4) administration route
1=Disease studied in this study<br>2= Previous history (please fill in the history number in the Notes)<br>3= Adverse events (please fill in the number of adverse events in Notes)	1 = micrograms (μg)<br>2 = milligram (mg)<br>3 = gram (g)<br>4 = milliliter (mL)<br>5 = International Unit (IU)<br>6=Unit(U)<br>7=Tablet(Tab)<br>8=capsule (Cap)<br>9=Unknown<br>10=Other,please fill in the line	1= Once a day (qd)<br>2= Twice a day (bid)<br>3= Three times a day (tid)<br>4= Four<br>6=Once a week（qw)<br>7=Before sleep(hs)<br>8=As required (prn)<br>9=Unknown<br>10=Other，please fill in the line	1=oral (PO)<br>2= intravenous drop(IVgtt)<br>3=Intravenous injection(IV)<br>4=Intramuscular (IM)<br>5= Subcutaneous<br>7= Local (skin) (TOP)<br>8= ophthalmus(OPH)<br>9=Inhalation(INH)<br>10=Unknown<br>11= Other，please fill in the line

4=Other（please describe in the Notes）		times a day (qid)<br>5= once every other day (qod)	(SQ)<br>6= sublingual (SL)

Does the subject have previous/combined medication?

□0 **NO** □1 **YES, please fill in the form below refer to the code at the bottom of the table.**

Previous/Combined medication

NO.	Drug Name (generic name)	Indications (1)	Notes for indications	single dose	Dose Unit (2)	Dosing frequency (3)	administration route (4)	Date of beginning	Sustain at the end of the study? Yes	NO→Date of end
1		|__|			|__|	|__|	|__|	|__|__|__|__|YEAR|__|__ |MONTH|__|__|DAY	□	|__|__|__|__|YEAR|__|__|MONTH|__|__|DAY
2		|__|			|__|	|__|	|__|	|__|__|__|__|YEAR|__|__ |MONTH|__|__|DAY	□	|__|__|__|__|YEAR|__|__|MONTH|__|__|DAY
3		|__|			|__|	|__|	|__|	|__|__|__|__|YEAR|__|__ |MONTH|__|__|DAY	□	|__|__|__|__|YEAR|__|__|MONTH|__|__|DAY
4		|__|			|__|	|__|	|__|	|__|__|__|__|YEAR|__|__ |MONTH|__|__|DAY	□	|__|__|__|__|YEAR|__|__|MONTH|__|__|DAY
5		|__|			|__|	|__|	|__|	|__|__|__|__|YEAR|__|__ |MONTH|__|__|DAY	□	|__|__|__|__|YEAR|__|__|MONTH|__|__|DAY

(1) Indications	(2) dosage Unit	(3) Dosing frequency	(4) administration route
1=Disease studied in this study<br>2= Previous history (please fill in the history number in the Notes)<br>3= Adverse events (please fill in the number of adverse events in Notes)	1 = micrograms (μg)<br>2 = milligram (mg)<br>3 = gram (g)<br>4 = milliliter (mL)<br>5 = International Unit (IU)<br>6=Unit(U)<br>7=Tablet(Tab)<br>8=capsule (Cap)<br>9=Unknown<br>10=Other,please fill in the line	1= Once a day (qd)<br>2= Twice a day (bid)<br>3= Three times a day (tid)<br>4= Four<br>6=Once a week (qw)<br>7=Before sleep(hs)<br>8=As required (prn)<br>9=Unknown<br>10=Other, please fill in the line	1=oral (PO)<br>2= intravenous drop(IVgtt)<br>3=Intravenous injection(IV)<br>4=Intramuscular (IM)<br>5= Subcutaneous<br>7= Local (skin) (TOP)<br>8= ophthalmus(OPH)<br>9=Inhalation(INH)<br>10=Unknown<br>11= Other, please fill in the line

4=Other (please describe in the Notes)		times a day (qid)<br>5= once every other day (qod)	(SQ)<br>6= sublingual (SL)

Does the subject have previous/combined non-drug treatment?				☐0 NO		☐1 YES，Please continue to fill in the form below.	
**Previous/Combined non-drug treatment**							
**NO.**	**Name of Treatment** (please describe in professional medical terms)	**Indications (1)**	**Notes for indications**	**Date of beginning**	**Sustain at the end of the study?**		**Whether it is the standard treatment recommended in the guidelines? (2)**
					**YES**	**NO→Date of end**	
**1**		|__|		|__|__|__|__|YEAR|__|__|MONTH|__|__|DAY	☐	|__|__|__|__|YEAR|__|__|MONTH|__|__|DAY	
**2**		|__|		|__|__|__|__|YEAR|__|__|MONTH|__|__|DAY	☐	|__|__|__|__|YEAR|__|__|MONTH|__|__|DAY	
**3**		|__|		|__|__|__|__|YEAR|__|__|MONTH|__|__|DAY	☐	|__|__|__|__|YEAR|__|__|MONTH|__|__|DAY	
**4**		|__|		|__|__|__|__|YEAR|__|__|MONTH|__|__|DAY	☐	|__|__|__|__|YEAR|__|__|MONTH|__|__|DAY	

**5**		|__|		|__|__|__|__|YEAR|__|__|MONTH|__|__|DAY	□	|__|__|__|__|YEAR|__|__|MONTH|__|__|DAY	
**6**		|__|		|__|__|__|__|YEAR|__|__|MONTH|__|__|DAY	□	|__|__|__|__|YEAR|__|__|MONTH|__|__|DAY	

（1）Indications：1**= Disease studied in this study 2= Previous history (please fill in the history number in the Notes) 3= Adverse events (please fill in the number of adverse events in Notes) 4=Other（please describe in the Notes）**

（2）Whether it is the standard treatment recommended in the guidelines? 0=NO 1=YES 2=Not applicable

IMV/NIV、ECMO、HFNC、Oxygen Therapy					
No.	Name of Therapy	Date of the beginning	Time of the beginning	Date of the end	Time of the end
	□1 IMV □3 ECMO □2 NIV □4 HFNC □5 Oxygen Therapy	|__|__|__|__|YEAR|__|__|MONTH|__|__|DAY	|__|__|：|__|__|	|__|__|__|__|YEAR|__|__|MONTH|__|__|DAY	|__|__|：|__|__|
	□1 IMV □3 ECMO □2 NIV □4 HFNC □5 Oxygen Therapy	|__|__|__|__|YEAR|__|__|MONTH|__|__|DAY	|__|__|：|__|__|	|__|__|__|__|YEAR|__|__|MONTH|__|__|DAY	|__|__|：|__|__|
	□1 IMV □3 ECMO □2 NIV □4 HFNC □5 Oxygen Therapy	|__|__|__|__|YEAR|__|__|MONTH|__|__|DAY	|__|__|：|__|__|	|__|__|__|__|YEAR|__|__|MONTH|__|__|DAY	|__|__|：|__|__|
	□1 IMV □3 ECMO □2 NIV □4 HFNC □5 Oxygen Therapy	|__|__|__|__|YEAR|__|__|MONTH|__|__|DAY	|__|__|：|__|__|	|__|__|__|__|YEAR|__|__|MONTH|__|__|DAY	|__|__|：|__|__|
	□1 IMV □3 ECMO □2 NIV □4 HFNC □5 Oxygen Therapy	|__|__|__|__|YEAR|__|__|MONTH|__|__|DAY	|__|__|：|__|__|	|__|__|__|__|YEAR|__|__|MONTH|__|__|DAY	|__|__|：|__|__|

Is there any other examination to record?		☐0 NO		☐1 YES，please fill in the table below.			
**Other Examinations**							
**Item**	**Date of sample or examine**	**Result**	**Unit**	**Result Interpretation**<br>**1=Normal**<br>**2=Abnormal, no clinical significance**<br>**3=Abnormal, with clinical significance**			**Notes**
	|__|__|__|__|YEAR|__|__|MONTH|__|__|DAY			☐1	☐2	☐3	
	|__|__|__|__|YEAR|__|__|MONTH|__|__|DAY			☐1	☐2	☐3	
	|__|__|__|__|YEAR|__|__|MONTH|__|__|DAY			☐1	☐2	☐3	
	|__|__|__|__|YEAR|__|__|MONTH|__|__|DAY			☐1	☐2	☐3	
	|__|__|__|__|YEAR|__|__|MONTH|__|__|DAY			☐1	☐2	☐3	
	|__|__|__|__|YEAR|__|__|MONTH|__|__|DAY			☐1	☐2	☐3	

Is there any other examination to record? ☐0 NO ☐1 YES，please fill in the table below.							
**Other Examinations**							
**Item**	**Date of sample or examine**	**Result**	**Unit**	**Result Interpretation**<br>**1=Normal**<br>**2=Abnormal, no clinical significance**<br>**3=Abnormal, significant clinically**			**Notes**
	|__|__|__|__|YEAR|__|__|MONTH|__|__|DAY			☐1	☐2	☐3	
	|__|__|__|__|YEAR|__|__|MONTH|__|__|DAY			☐1	☐2	☐3	
	|__|__|__|__|YEAR|__|__|MONTH|__|__|DAY			☐1	☐2	☐3	
	|__|__|__|__|YEAR|__|__|MONTH|__|__|DAY			☐1	☐2	☐3	
	|__|__|__|__|YEAR|__|__|MONTH|__|__|DAY			☐1	☐2	☐3	
	|__|__|__|__|YEAR|__|__|MONTH|__|__|DAY			☐1	☐2	☐3	
	|__|__|__|__|YEAR|__|__|MONTH|__|__|DAY			☐1	☐2	☐3	

	|__|__|__|__|YEAR|__|__|MONTH|__|__|DAY			□1	□2	□3	
	|__|__|__|__|YEAR|__|__|MONTH|__|__|DAY			□1	□2	□3	

Study Summary
Whether the subject finished the study according to the protocol？<br>□1 YES，Finish Date：|__|__|__|__|YEAR|__|__|MONTH|__|__|DAY<br>□0 NO，End Date：|__|__|__|__|YEAR|__|__|MONTH|__|__|DAY
If the patient exit ahead of schedule，please choose the most important reason（One-Choice）:<br>□1 Disease progression occurred during the study and changes in treatment were required after comprehensive assessment by the investigators.<br>□2 Adverse events lead to an exacerbation of the disease<br>□3 The subject is reluctant to continue using the study drug.<br>□4 Pregnancy<br>□5 Loss to follow-up<br>□6 Death<br>□7 Subject withdraws the Informed Consent<br>□8 Study suspension<br>□9 Other，please describe：____________________<br>____________________<br><br>Notes（Please record other matter which needs additional clarification）:<br>____________________<br>____________________<br>____________________

Information on hospitalization
Was the subject hospitalized with 2019-nCov respiratory infection？□0 NO □1 YES
If Yes，date of admission：|__|__|__|__|YEAR|__|__|MONTH|__|__|DAY
Be discharged from hospital at the end of the study or not？ □0 NO □1 YES
If yes，discharge date ：|__|__|__|__|YEAR|__|__|MONTH|__|__|DAY

Death
Are the subjects dead? □0 NO □1 YES，please fill in □2 Unknown
Date of death：|__|__|__|__|YEAR|__|__|MONTH|__|__|DAY
The cause of death ：<br>□1 Adverse events<br>□2 Unknown<br>□3 Other，please describe____<br>____

Investigator Statement
I have reviewed and confirmed that all data recorded in this case report form are true, accurate, reliable, complete, and consistent with the original data. All errors or omissions have been corrected or noted.
**Signature of principal investigator：** ________________
**Date：|__|__|__|__|YEAR|__|__|MONTH|__|__|DAY**

CRA Statement
I have checked and confirmed that all the data recorded in this case report form are true, accurate, reliable, complete and correct and consistent with the original data.
**CRA Signature：** ____________________
**Date：|__|__|__|__|YEAR|__|__|MONTH|__|__|DAY**

Vital Signs	□0Unexamined，reason________________________________		
Date	|__|__|__|__|YEAR|__|__|MONTH|__|__|DAY		
Daily maximum temperature	|__|__|.|__|℃	Measurement method	□1 Axilla □2Oral □3 Anus □4 Ear □5 Unknown □6 Other，please describe________
Respiratory	|__|__|times/min	Pulse	|__|__|__|times/min
Blood pressure	|__|__|__|/|__|__|__| mmHg（Systolic/diastolic blood pressure）		

Vital Signs	□0Unexamined，reason________________________________		
Date	|__|__|__|__|YEAR|__|__|MONTH|__|__|DAY		
Daily maximum temperature	|__|__|.|__|℃	Measurement method	□1 Axilla □2Oral □3 Anus □4 Ear □5 Unknown □6 Other，please describe_______
Respiratory	|__|__|times/min	Pulse	|__|__|__|times/min
Blood pressure	|__|__|__|/|__|__|__| mmHg（Systolic/diastolic blood pressure）		

Vital Signs	□0Unexamined，reason________________________________		
Date	|__|__|__|__|YEAR|__|__|MONTH|__|__|DAY		
Daily maximum temperature	|__|__|.|__|℃	Measurement method	□1 Axilla □2Oral □3 Anus □4 Ear □5 Unknown □6 Other，please describe_______
Respiratory	|__|__|times/min	Pulse	|__|__|__|times/min
Blood pressure	|__|__|__|/|__|__|__| mmHg（Systolic/diastolic blood pressure）		

Vital Signs	□0Unexamined，reason________________________________		
Date	|__|__|__|__|YEAR|__|__|MONTH|__|__|DAY		
Daily maximum temperature	|__|__|.|__|℃	Measurement method	□1 Axilla □2Oral □3 Anus □4 Ear □5 Unknown □6 Other，please describe_______
Respiratory	|__|__|times/min	Pulse	|__|__|__|times/min
Blood pressure	|__|__|__|/|__|__|__| mmHg（Systolic/diastolic blood pressure）		

**physical examination**	☐0 Unexamined, **reason**:________________				
Date	|__|__|__|__|YEAR|__|__|MONTH|__|__|DAY				
Inspection Part	Result<br>1= Normal<br>2= Abnormal, no clinical significance<br>3= Abnormal, with clinical significance<br>0=Unexamined				If abnormal，please describe
General appearance	☐1	☐2	☐3	☐0	
Head	☐1	☐2	☐3	☐0	
Eyes	☐1	☐2	☐3	☐0	
Nose	☐1	☐2	☐3	☐0	
Larynx	☐1	☐2	☐3	☐0	
Neck and thyroid	☐1	☐2	☐3	☐0	
Skin	☐1	☐2	☐3	☐0	
Cardiovascular system	☐1	☐2	☐3	☐0	
Chest and lungs	☐1	☐2	☐3	☐0	
Abdomen	☐1	☐2	☐3	☐0	
Nervous system	☐1	☐2	☐3	☐0	
Musculoskeletal system	☐1	☐2	☐3	☐0	
Lymphatic system	☐1	☐2	☐3	☐0	

Protocol Number：KY2020-29		Attachment Out of Plan
Center Number |__|__|	Subject Screening Number |__|__|__|__|__|__|	

Respiratory symptoms	□0Unexamined，reason_______________
Date：|__|__|__|__|YEAR|__|__|MONTH|__|__|DAY	
Whether the subject has respiratory symptoms or not：□0 NO □1 NO，please describe：_____	
Result：□1 Normal □2 Abnormal No clinical significance □3 Abnormal Significant clinically	

Respiratory symptoms	□0Unexamined，reason_______________
Date：|__|__|__|__|YEAR|__|__|MONTH|__|__|DAY	
Whether the subject has respiratory symptoms or not：□0 NO □1 NO，please describe：_____	
Result：□1 Normal □2 Abnormal No clinical significance □3 Abnormal Significant clinically	

Respiratory symptoms	□0Unexamined，reason_______________
Date：|__|__|__|__|YEAR|__|__|MONTH|__|__|DAY	
Whether the subject has respiratory symptoms or not：□0 NO □1 NO，please describe：_____	
Result：□1 Normal □2 Abnormal No clinical significance □3 Abnormal Significant clinically	

Respiratory symptoms	□0Unexamined，reason_______________
Date：|__|__|__|__|YEAR|__|__|MONTH|__|__|DAY	
Whether the subject has respiratory symptoms or not：□0 NO □1 NO，please describe：_____	
Result：□1 Normal □2 Abnormal No clinical significance □3 Abnormal Significant clinically	

Respiratory symptoms	□0Unexamined，reason_______________
Date：|__|__|__|__|YEAR|__|__|MONTH|__|__|DAY	
Whether the subject has respiratory symptoms or not：□0 NO □1 NO，please describe：_____	
Result：□1 Normal □2 Abnormal No clinical significance □3 Abnormal Significant clinically	

Respiratory symptoms	□0Unexamined，reason_______________
Date：|__|__|__|__|YEAR|__|__|MONTH|__|__|DAY	
Whether the subject has respiratory symptoms or not：□0 NO □1 NO，please describe：_____	
Result：□1 Normal □2 Abnormal No clinical significance □3 Abnormal Significant clinically	

Blood Count □0Unexamined， reason______

Date：\|__\|__\|__\|__\|YEAR\|__\|__\|MONTH\|__\|__\|DAY Normal range Number：\|__\|__\|__\|__\|

Item	Result		Result Interpretation			Unexamined
	Measured Value	Unit	Normal	Abnormal: No clinical significance	Abnormal: Significant clinically	
Red Blood Cell（RBC）		□1 10^{12}/L □2Other：_____	□1	□2	□3	□0
White Blood Cell		□1 10^{9}/L □2Other：_____	□1	□2	□3	□0
Hemoglobin（Hb）		□1 g/L □2Other：_____	□1	□2	□3	□0
Platelet（PLT）		□1 10^{9}/L □2Other：_____	□1	□2	□3	□0
Neutrophil（ANC）		□1 10^{9}/L □2Other：_____	□1	□2	□3	□0
Lymphocyte（LY）		□1 10^{9}/L □2Other：_____	□1	□2	□3	□0
Monocyte（MO）		□1 10^{9}/L □2Other：_____	□1	□2	□3	□0
Eosinophil（EO）		□1 10^{9}/L □2Other：_____	□1	□2	□3	□0
Basophil（BA）		□1 10^{9}/L □2Other：_____	□1	□2	□3	□0

Blood Count □0Unexamined， reason______

Date：\|__\|__\|__\|__\|YEAR\|__\|__\|MONTH\|__\|__\|DAY Normal range Number：\|__\|__\|__\|__\|

Item	Result		Result Interpretation			Unexamined
	Measured Value	Unit	Normal	Abnormal: No clinical significance	Abnormal: Significant clinically	
Red Blood Cell（RBC）		□1 10^{12}/L □2Other：_____	□1	□2	□3	□0
White Blood Cell		□1 10^{9}/L □2Other：_____	□1	□2	□3	□0
Hemoglobin（Hb）		□1 g/L □2Other：_____	□1	□2	□3	□0
Platelet（PLT）		□1 10^{9}/L □2Other：_____	□1	□2	□3	□0
Neutrophil（ANC）		□1 10^{9}/L □2Other：_____	□1	□2	□3	□0
Lymphocyte（LY）		□1 10^{9}/L □2Other：_____	□1	□2	□3	□0
Monocyte（MO）		□1 10^{9}/L □2Other：_____	□1	□2	□3	□0
Eosinophil（EO）		□1 10^{9}/L □2Other：_____	□1	□2	□3	□0
Basophil（BA）		□1 10^{9}/L □2Other：_____	□1	□2	□3	□0

Blood Biochemistry	□0Unexamined，reason________________
Date|__|__|__|__|YEAR|__|__|MONTH|__|__|DAY	Normal range Number：|__|__|__|__|

Item	Result		Result Interpretation			Unexamined
	Measured Value	Unit	Normal	Abnormal		
				No clinical significance	Significant clinically	
Glutamic-pyruvic transaminase（ALT）		□1 U/L □2Other：_____	□1	□2	□3	□0
Glutamic-oxalacetic transaminease（AST）		□1 U/L □2Other：_____	□1	□2	□3	□0
Total Bilirubin（TBIL）		□1 μmol/L □2Other：_____	□1	□2	□3	□0
Albumin（ALB）		□1 g/L □2Other：_____	□1	□2	□3	□0
γ- glutamyl transpeptidas（GGT）		□1 U/L □2Other：_____	□1	□2	□3	□0
Lactic dehydrogenase（LDH）		□1 U/L □2Other：_____	□1	□2	□3	□0
Creatine kinase（CK）		□1 U/L □2Other：_____	□1	□2	□3	□0
Creatine kinase isoenzyme（CK-MB）		□1 U/L □2Other：_____	□1	□2	□3	□0
Blood urea nitrogen（BUN）		□1 mmol/L □2Other：_____	□1	□2	□3	□0
Urea（Urea）		□1 mmol/L □2Other：_____	□1	□2	□3	□0
Serum creatinine（CR）		□1 μmol/L □2Other：_____	□1	□2	□3	□0

**Urine Test** □0Unexamined，reason________							
Date：|__|__|__|__|YEAR|__|__|MONTH|__|__|DAY Normal range Number：|__|__|__|__|							
**Item**	**Result**		**Result Interpretation**			**Unexamined**
	**Measured Value**	**Unit**	**Normal**	**Abnormal**		
				**No clinical significance**	**Significant clinically**	
Urine Protein		□1Not Applicable □2Other：_____	□1	□2	□3	□0
Urine Glucose		□1 mmol/L □2Other：_____	□1	□2	□3	□0
Urine PH value		□1 Not Applicable □2Other：_____	□1	□2	□3	□0
Urine Erythrocyte		□1Not Applicable □2Other：_____	□1	□2	□3	□0
Urine Leukocyte		□1Not Applicable □2Other：_____	□1	□2	□3	□0
Urine Ketone		□1Not Applicable □2Other：_____	□1	□2	□3	□0

**Urine Test** □0Unexamined，reason________						
Date：|__|__|__|__|YEAR|__|__|MONTH|__|__|DAY Normal range Number：|__|__|__|__|						
**Item**	**Result**		**Result Interpretation**			**Unexamined**
	**Measured Value**	**Unit**	**Normal**	**Abnormal**		
				**No clinical significance**	**Significant clinically**	
Urine Protein		□1Not Applicable □2Other：_____	□1	□2	□3	□0
Urine Glucose		□1 mmol/L □2Other：_____	□1	□2	□3	□0
Urine PH value		□1 Not Applicable □2Other：_____	□1	□2	□3	□0
Urine Erythrocyte		□1Not Applicable □2Other：_____	□1	□2	□3	□0
Urine Leukocyte		□1Not Applicable □2Other：_____	□1	□2	□3	□0
Urine Ketone		□1Not Applicable □2Other：_____	□1	□2	□3	□0

Protocol Number：KY2020-29		Attachment Out of Plan
Center Number |__|__|	Subject Screening Number |__|__|__|__|__|__|	

Coagulation Function	□0Unexamined，reason________________						
Date：|__|__|__|__|YEAR|__|__|MONTH|__|__|DAY	Normal range Number：|__|__|__|__|__|						
Item	Result		Result Interpretation			Unexamined	
	Measured Value	Unit		Normal	Abnormal: No clinical significance	Abnormal: Significant clinically	
ProthrombinTime（PT）		□1 S	□2Other：_____	□1	□2	□3	□0
Activated Partial Thromboplastin Time（APTT）		□1 S	□2Other：_____	□1	□2	□3	□0
Thrombin Time（TT）		□1 S	□2Other：_____	□1	□2	□3	□0
Fibrinogen（Fbg）		□1 g/L	□2Other：_____	□1	□2	□3	□0
International Normalized Ratio（INR）		□1Not Applicable	□2Other：_____	□1	□2	□3	□0

Coagulation Function	□0Unexamined，reason________________						
Date：|__|__|__|__|YEAR|__|__|MONTH|__|__|DAY	Normal range Number：|__|__|__|__|__|						
Item	Result		Result Interpretation			Unexamined	
	Measured Value	Unit		Normal	Abnormal: No clinical significance	Abnormal: Significant clinically	
ProthrombinTime（PT）		□1 S	□2Other：_____	□1	□2	□3	□0
Activated Partial Thromboplastin Time（APTT）		□1 S	□2Other：_____	□1	□2	□3	□0
Thrombin Time（TT）		□1 S	□2Other：_____	□1	□2	□3	□0
Fibrinogen（Fbg）		□1 g/L	□2Other：_____	□1	□2	□3	□0
International Normalized Ratio（INR）		□1Not Applicable	□2Other：_____	□1	□2	□3	□0

**C-REACTION PROTEIN（CRP）** □0Unexamined，reason________________						
Date：|__|__|__|__|YEAR|__|__|MONTH|__|__|DAY Normal range Number：|__|__|__|__|						
**Item**	**Result**		**Result Interpretation**			
				**Abnormal**		
C-REACTION PROTEIN（CRP）	**Measured Value**	**Unit**	**Normal**	**No clinical significance**	**Significant clinically**	
		□1 mg/L □2Other：_____	□1	□2	□3	

**C-REACTION PROTEIN（CRP）** □0Unexamined，reason________________						
Date：|__|__|__|__|YEAR|__|__|MONTH|__|__|DAY Normal range Number：|__|__|__|__|						
**Item**	**Result**		**Result Interpretation**			
				**Abnormal**		
C-REACTION PROTEIN（CRP）	**Measured Value**	**Unit**	**Normal**	**No clinical significance**	**Significant clinically**	
		□1 mg/L □2Other：_____	□1	□2	□3	

**C-REACTION PROTEIN（CRP）** □0Unexamined，reason________________						
Date：|__|__|__|__|YEAR|__|__|MONTH|__|__|DAY Normal range Number：|__|__|__|__|						
**Item**	**Result**		**Result Interpretation**			
				**Abnormal**		
C-REACTION PROTEIN（CRP）	**Measured Value**	**Unit**	**Normal**	**No clinical significance**	**Significant clinically**	
		□1 mg/L □2Other：_____	□1	□2	□3	

**C-REACTION PROTEIN（CRP）** □0Unexamined，reason________________						
Date：|__|__|__|__|YEAR|__|__|MONTH|__|__|DAY Normal range Number：|__|__|__|__|						
**Item**	**Result**		**Result Interpretation**			
				**Abnormal**		
C-REACTION PROTEIN（CRP）	**Measured Value**	**Unit**	**Normal**	**No clinical significance**	**Significant clinically**	
		□1 mg/L □2Other：_____	□1	□2	□3	

Erythrocyte Sedimentation Rate（ESR） □0Unexamined, reason________________

Date : |__|__|__|__|YEAR|__|__|MONTH|__|__|DAY Normal range

Number: |__|__|__|__|

Item	Result		Result Interpretation		
				Abnormal	
Erythrocyte Sedimentation Rate (ESR)	Measured Value	Unit	Normal	No clinical significance	Significant clinically
		□1 mm/h □2Other:	□1	□2	□3

Erythrocyte Sedimentation Rate（ESR） □0Unexamined, reason________________

Date : |__|__|__|__|YEAR|__|__|MONTH|__|__|DAY Normal range

Number: |__|__|__|__|

Item	Result		Result Interpretation		
				Abnormal	
Erythrocyte Sedimentation Rate (ESR)	Measured Value	Unit	Normal	No clinical significance	Significant clinically
		□1 mm/h □2Other:	□1	□2	□3

Erythrocyte Sedimentation Rate（ESR） □0Unexamined, reason________________

Date : |__|__|__|__|YEAR|__|__|MONTH|__|__|DAY Normal range

Number: |__|__|__|__|

Item	Result		Result Interpretation		
				Abnormal	
Erythrocyte Sedimentation Rate (ESR)	Measured Value	Unit	Normal	No clinical significance	Significant clinically
		□1 mm/h □2Other:	□1	□2	□3

Erythrocyte Sedimentation Rate（ESR） □0Unexamined, reason________________

Date : |__|__|__|__|YEAR|__|__|MONTH|__|__|DAY Normal range

Number: |__|__|__|__|

Item	Result		Result Interpretation		
				Abnormal	
Erythrocyte Sedimentation Rate (ESR)	Measured Value	Unit	Normal	No clinical significance	Significant clinically
		□1 mm/h □2Other:	□1	□2	□3

**TNF-α** □0Unexamined, reason________________						
Date: |__|__|__|__|YEAR|__|__|MONTH|__|__|DAY Normal range Number: |__|__|__|__|						
**Item**	**Result**			**Result Interpretation**		
	**Measured Value**	**Unit**		**Normal**	**Abnormal**	
					**No clinical significance**	**Significant clinically**
TNF-α		□1 pg/mL	□2Other:	□1	□2	□3

**TNF-α** □0Unexamined, reason________________						
Date: |__|__|__|__|YEAR|__|__|MONTH|__|__|DAY Normal range Number: |__|__|__|__|						
**Item**	**Result**			**Result Interpretation**		
	**Measured Value**	**Unit**		**Normal**	**Abnormal**	
					**No clinical significance**	**Significant clinically**
TNF-α		□1 pg/mL	□2Other:	□1	□2	□3

**TNF-α** □0Unexamined, reason________________						
Date: |__|__|__|__|YEAR|__|__|MONTH|__|__|DAY Normal range Number: |__|__|__|__|						
**Item**	**Result**			**Result Interpretation**		
	**Measured Value**	**Unit**		**Normal**	**Abnormal**	
					**No clinical significance**	**Significant clinically**
TNF-α		□1 pg/mL	□2Other:	□1	□2	□3

**TNF-α** □0Unexamined, reason________________						
Date: |__|__|__|__|YEAR|__|__|MONTH|__|__|DAY Normal range Number: |__|__|__|__|						
**Item**	**Result**			**Result Interpretation**		
	**Measured Value**	**Unit**		**Normal**	**Abnormal**	
					**No clinical significance**	**Significant clinically**
TNF-α		□1 pg/mL	□2Other:	□1	□2	□3

**TNF-α** □0Unexamined, reason________________						
Date: |__|__|__|__|YEAR|__|__|MONTH|__|__|DAY Normal range Number: |__|__|__|__|						
**Item**	**Result**			**Result Interpretation**		
	**Measured Value**	**Unit**		**Normal**	**Abnormal**	
					**No clinical significance**	**Significant clinically**
TNF-α		□1 pg/mL	□2Other:	□1	□2	□3

IL-6 □0Unexamined， reason________________

Date：\|__\|__\|__\|__\|YEAR\|__\|__\|MONTH\|__\|__\|DAY Normal range Number：\|__\|__\|__\|__\|

Item	Result			Result Interpretation		
					Abnormal	
IL-6	Measured Value	Unit		Normal	No clinical significance	Significant clinically
		□1 pg/mL	□2Other:	□1	□2	□3

IL-6 □0Unexamined， reason________________

Date：\|__\|__\|__\|__\|YEAR\|__\|__\|MONTH\|__\|__\|DAY Normal range Number：\|__\|__\|__\|__\|

Item	Result			Result Interpretation		
					Abnormal	
IL-6	Measured Value	Unit		Normal	No clinical significance	Significant clinically
		□1 pg/mL	□2Other:	□1	□2	□3

IL-6 □0Unexamined， reason________________

Date：\|__\|__\|__\|__\|YEAR\|__\|__\|MONTH\|__\|__\|DAY Normal range Number：\|__\|__\|__\|__\|

Item	Result			Result Interpretation		
					Abnormal	
IL-6	Measured Value	Unit		Normal	No clinical significance	Significant clinically
		□1 pg/mL	□2Other:	□1	□2	□3

IL-6 □0Unexamined， reason________________

Date：\|__\|__\|__\|__\|YEAR\|__\|__\|MONTH\|__\|__\|DAY Normal range Number：\|__\|__\|__\|__\|

Item	Result			Result Interpretation		
					Abnormal	
IL-6	Measured Value	Unit		Normal	No clinical significance	Significant clinically
		□1 pg/mL	□2Other:	□1	□2	□3

IL-6 □0Unexamined， reason________________

Date：\|__\|__\|__\|__\|YEAR\|__\|__\|MONTH\|__\|__\|DAY Normal range Number：\|__\|__\|__\|__\|

Item	Result			Result Interpretation		
					Abnormal	
IL-6	Measured Value	Unit		Normal	No clinical significance	Significant clinically
		□1 pg/mL	□2Other:	□1	□2	□3

Urine or serum HCG (Only applicable to women of childbearing age) □0Unexamined, reason________ □1Not Applicable（Man or woman who isn't able to reproduce）
Date：|__|__|__|__|YEAR|__|__|MONTH|__|__|DAY
Sample：□1 Blood □2 Urine
Result：□1 Negative □2 Positive

Urine or serum HCG (Only applicable to women of childbearing age) □0Unexamined, reason________ □1Not Applicable（Man or woman who isn't able to reproduce）
Date：|__|__|__|__|YEAR|__|__|MONTH|__|__|DAY
Sample：□1 Blood □2 Urine
Result：□1 Negative □2 Positive

Urine or serum HCG (Only applicable to women of childbearing age) □0Unexamined, reason________ □1Not Applicable（Man or woman who isn't able to reproduce）
Date：|__|__|__|__|YEAR|__|__|MONTH|__|__|DAY
Sample：□1 Blood □2 Urine
Result：□1 Negative □2 Positive

Urine or serum HCG (Only applicable to women of childbearing age) □0Unexamined, reason________ □1Not Applicable（Man or woman who isn't able to reproduce）
Date：|__|__|__|__|YEAR|__|__|MONTH|__|__|DAY
Sample：□1 Blood □2 Urine
Result：□1 Negative □2 Positive

Urine or serum HCG (Only applicable to women of childbearing age) □0Unexamined, reason________ □1Not Applicable（Man or woman who isn't able to reproduce）
Date：|__|__|__|__|YEAR|__|__|MONTH|__|__|DAY

Sample：□1 Blood □2 Urine
Result：□1 Negative □2 Positive

**Routine 12 Leads Electrocardiogram** □0Unexamined，reason________________________
Date：|__|__|__|__|YEAR|__|__|MONTH|__|__|DAY
Result：□1 Normal □2 Abnormal No clinical significance □3 Abnormal Significant clinically
If “Abnormal”，please describe：____

**Routine 12 Leads Electrocardiogram** □0Unexamined，reason________________________
Date：|__|__|__|__|YEAR|__|__|MONTH|__|__|DAY
Result：□1 Normal □2 Abnormal No clinical significance □3 Abnormal Significant clinically
If “Abnormal”，please describe：____

**Routine 12 Leads Electrocardiogram** □0Unexamined，reason________________________
Date：|__|__|__|__|YEAR|__|__|MONTH|__|__|DAY
Result：□1 Normal □2 Abnormal No clinical significance □3 Abnormal Significant clinically
If “Abnormal”，please describe：____

**Routine 12 Leads Electrocardiogram** □0Unexamined，reason________________________
Date：|__|__|__|__|YEAR|__|__|MONTH|__|__|DAY
Result：□1 Normal □2 Abnormal No clinical significance □3 Abnormal Significant clinically
If “Abnormal”，please describe：____

**Routine 12 Leads Electrocardiogram** □0Unexamined，reason________________________
Date：|__|__|__|__|YEAR|__|__|MONTH|__|__|DAY
Result：□1 Normal □2 Abnormal No clinical significance □3 Abnormal Significant clinically
If “Abnormal”，please describe：____

**Routine 12 Leads Electrocardiogram** □0Unexamined，reason________________________
Date：|__|__|__|__|YEAR|__|__|MONTH|__|__|DAY
Result：□1 Normal □2 Abnormal No clinical significance □3 Abnormal Significant clinically
If “Abnormal”，please describe：____

**Nucleic Acid Test of 2019-nCov**	□0 Unexamined，reason________________________ □1 Not Applicable
Sampling Part：□1Upper respiratory tract □2Lower respiratory tract	
Date：|__|__|__|__|YEAR|__|__|MONTH|__|__|DAY	Date：|__|__|:|__|__|
Result：□1 Negative □2 Positive □3 Unidentified □4 Other，please describe______________	

**Nucleic Acid Test of 2019-nCov**	□0 Unexamined，reason________________________ □1 Not Applicable
Sampling Part：□1Upper respiratory tract □2Lower respiratory tract	
Date：|__|__|__|__|YEAR|__|__|MONTH|__|__|DAY	Date：|__|__|:|__|__|
Result：□1 Negative □2 Positive □3 Unidentified □4 Other，please describe______________	

**Nucleic Acid Test of 2019-nCov**	□0 Unexamined，reason________________________ □1 Not Applicable
Sampling Part：□1Upper respiratory tract □2Lower respiratory tract	
Date：|__|__|__|__|YEAR|__|__|MONTH|__|__|DAY	Date：|__|__|:|__|__|
Result：□1 Negative □2 Positive □3 Unidentified □4 Other，please describe______________	

**Nucleic Acid Test of 2019-nCov**	□0 Unexamined，reason________________________ □1 Not Applicable
Sampling Part：□1Upper respiratory tract □2Lower respiratory tract	
Date：|__|__|__|__|YEAR|__|__|MONTH|__|__|DAY	Date：|__|__|:|__|__|
Result：□1 Negative □2 Positive □3 Unidentified □4 Other，please describe______________	

**Nucleic Acid Test of 2019-nCov**	□0 Unexamined，reason________________________ □1 Not Applicable
Sampling Part：□1Upper respiratory tract □2Lower respiratory tract	
Date：|__|__|__|__|YEAR|__|__|MONTH|__|__|DAY	Date：|__|__|:|__|__|
Result：□1 Negative □2 Positive □3 Unidentified □4 Other，please describe______________	

Pulse oxygen saturation（without oxygen inhalation ）	□0Unexamined, reason_____
Date: |__|__|__|__|YEAR|__|__|MONTH|__|__|DAY	
Measured Value: _______________%（Rest state）	

Pulse oxygen saturation（without oxygen inhalation ）	□0Unexamined, reason_____
Date: |__|__|__|__|YEAR|__|__|MONTH|__|__|DAY	
Measured Value: _______________%（Rest state）	

Pulse oxygen saturation（without oxygen inhalation ）	□0Unexamined, reason_____
Date: |__|__|__|__|YEAR|__|__|MONTH|__|__|DAY	
Measured Value: _______________%（Rest state）	

Pulse oxygen saturation（without oxygen inhalation ）	□0Unexamined, reason_____
Date: |__|__|__|__|YEAR|__|__|MONTH|__|__|DAY	
Measured Value: _______________%（Rest state）	

Pulse oxygen saturation（without oxygen inhalation ）	□0Unexamined, reason_____
Date: |__|__|__|__|YEAR|__|__|MONTH|__|__|DAY	
Measured Value: _______________%（Rest state）	

Pulse oxygen saturation（without oxygen inhalation ）	□0Unexamined, reason_____
Date: |__|__|__|__|YEAR|__|__|MONTH|__|__|DAY	
Measured Value: _______________%（Rest state）	

Pulse oxygen saturation（without oxygen inhalation ）	□0Unexamined, reason_____
Date: |__|__|__|__|YEAR|__|__|MONTH|__|__|DAY	
Measured Value: _______________%（Rest state）	

**Whether develops into severe/critical type** □0 No evaluation Date |__|__|__|__|YEAR|__|__|MONTH|__|__|DAY
Whether develops into severe/critical type： □0 NO □1 YES
Date of developing into severe/critical type： |__|__|__|__|YEAR|__|__|MONTH|__|__|DAY

**Whether develops into severe/critical type** □0 No evaluation Date |__|__|__|__|YEAR|__|__|MONTH|__|__|DAY
Whether develops into severe/critical type： □0 NO □1 YES
Date of developing into severe/critical type： |__|__|__|__|YEAR|__|__|MONTH|__|__|DAY

**Whether develops into severe/critical type** □0 No evaluation Date |__|__|__|__|YEAR|__|__|MONTH|__|__|DAY
Whether develops into severe/critical type： □0 NO □1 YES
Date of developing into severe/critical type： |__|__|__|__|YEAR|__|__|MONTH|__|__|DAY

**Whether develops into severe/critical type** □0 No evaluation Date |__|__|__|__|YEAR|__|__|MONTH|__|__|DAY
Whether develops into severe/critical type： □0 NO □1 YES
Date of developing into severe/critical type： |__|__|__|__|YEAR|__|__|MONTH|__|__|DAY

**Whether develops into severe/critical type** □0 No evaluation Date |__|__|__|__|YEAR|__|__|MONTH|__|__|DAY
Whether develops into severe/critical type： □0 NO □1 YES
Date of developing into severe/critical type： |__|__|__|__|YEAR|__|__|MONTH|__|__|DAY

**Whether develops into severe/critical type** □0 No evaluation Date |__|__|__|__|YEAR|__|__|MONTH|__|__|DAY
Whether develops into severe/critical type： □0 NO □1 YES
Date of developing into severe/critical type： |__|__|__|__|YEAR|__|__|MONTH|__|__|DAY

**Chest CT** □0Unexamined，reason____
Date：|__|__|__|__|YEAR|__|__|MONTH|__|__|DAY
Result：□1 Normal □2 Abnormal, no clinical significance □3 Abnormal，significant clinically __
If abnormal, please describe ______________________________
Whether chest CT lesion absorbed≥50% than baseline?? □0 NO □1 YES □2 Unidentified □3 Unknown □4 Other，please describe：________________________________

**Chest CT** □0Unexamined，reason____
Date：|__|__|__|__|YEAR|__|__|MONTH|__|__|DAY
Result：□1 Normal □2 Abnormal, no clinical significance □3 Abnormal，significant clinically __
If abnormal, please describe ______________________________
Whether chest CT lesion absorbed≥50% than baseline?? □0 NO □1 YES □2 Unidentified □3 Unknown □4 Other，please describe：________________________________

**Chest CT** □0Unexamined，reason____
Date：|__|__|__|__|YEAR|__|__|MONTH|__|__|DAY
Result：□1 Normal □2 Abnormal, no clinical significance □3 Abnormal，significant clinically __
If abnormal, please describe ______________________________
Whether chest CT lesion absorbed≥50% than baseline?? □0 NO □1 YES □2 Unidentified □3 Unknown □4 Other，please describe：________________________________

**Arterial Blood Gas Analysis** □0Unexamined，reason________________________						
Inhalation oxygen or not? □0 No □1Yes, (FiO2):_______________%						
Date: |__|__|__|__|YEAR|__|__|MONTH|__|__|DAY						
**Item**	**Result**		**Result Interpretation**			**Unexamined**
	**Measured Value**	**Unit**	**Normal**	**Abnormal**		
				**No clinical significance**	**Significant clinically**	
Arterial partial pressure of oxygen (PaO2)		□1 mmHg □2 Other:_____	□1	□2	□3	□0
Oxygen saturation (SaO2)		□1 % □2 Other:_____	□1	□2	□3	□0

**Arterial Blood Gas Analysis** □0Unexamined，reason________________________						
Inhalate oxygen or not? □0 No □1Yes, (FiO2):_______________%						
Date: |__|__|__|__|YEAR|__|__|MONTH|__|__|DAY						
**Item**	**Result**		**Result Interpretation**			**Unexamined**
	**Measured Value**	**Unit**	**Normal**	**Abnormal**		
				**No clinical significance**	**Significant clinically**	
Arterial partial pressure of oxygen (PaO2)		□1 mmHg □2 Other:_____	□1	□2	□3	□0
Oxygen saturation (SaO2)		□1 % □2 Other:_____	□1	□2	□3	□0

**Clinical status**	□0 No evaluation，reason________________________
Date：	|__|__|__|__|YEAR|__|__|MONTH|__|__|DAY
Result	□1 Level 1: discharge<br>□2 Level 2: hospitalization，oxygen therapy is not required<br>□3 Level 3: hospitalization，require oxygen therapy<br>□4 Level 4:ICU/ hospitalization，require noninvasive ventilation (NIV)/ High flow nasal catheter oxygen therapy (HFNC)<br>□5 Level 5:ICU，require extracorporeal membrane oxygenation (ECMO)and/or invasive mechanical ventilation(IMV)<br>□6 Level 6:Death

**Clinical status**	□0 No evaluation，reason________________________
Date：	|__|__|__|__|YEAR|__|__|MONTH|__|__|DAY
Result	□1 Level 1: discharge<br>□2 Level 2: hospitalization，oxygen therapy is not required<br>□3 Level 3: hospitalization，require oxygen therapy<br>□4 Level 4:ICU/ hospitalization，require noninvasive ventilation (NIV)/ High flow nasal catheter oxygen therapy (HFNC)<br>□5 Level 5:ICU，require extracorporeal membrane oxygenation (ECMO)and/or invasive mechanical ventilation(IMV)<br>□6 Level 6:Death

**Clinical status**	□0 No evaluation，reason________________________
Date：	|__|__|__|__|YEAR|__|__|MONTH|__|__|DAY
Result	□1 Level 1: discharge<br>□2 Level 2: hospitalization，oxygen therapy is not required<br>□3 Level 3: hospitalization，require oxygen therapy<br>□4 Level 4:ICU/ hospitalization，require noninvasive ventilation (NIV)/ High flow nasal catheter oxygen therapy (HFNC)<br>□5 Level 5:ICU，require extracorporeal membrane oxygenation (ECMO)and/or invasive mechanical ventilation(IMV)<br>□6 Level 6:Death

Signment of the Informed Consent Form (ICF)	
Date of signing the Informed Consent	|__|__|__|__|YEAR|__|__|MONTH|__|__|DAY
Version number of the Informed Consent	____________
The version number of the protocol shall apply when the Informed Consent is signed	__|__|.|__|

Signment of the Informed Consent Form (ICF)	
Date of signing the Informed Consent	|__|__|__|__|YEAR|__|__|MONTH|__|__|DAY
Version number of the Informed Consent	____________
The version number of the protocol shall apply when the Informed Consent is signed	__|__|.|__|

Signment of the Informed Consent Form (ICF)	
Date of signing the Informed Consent	|__|__|__|__|YEAR|__|__|MONTH|__|__|DAY
Version number of the Informed Consent	____________
The version number of the protocol shall apply when the Informed Consent is signed	__|__|.|__|

Signment of the Informed Consent Form (ICF)	
Date of signing the Informed Consent	|__|__|__|__|YEAR|__|__|MONTH|__|__|DAY
Version number of the Informed Consent	____________
The version number of the protocol shall apply when the Informed Consent is signed	__|__|.|__|

Signment of the Informed Consent Form (ICF)	
Date of signing the Informed Consent	|__|__|__|__|YEAR|__|__|MONTH|__|__|DAY
Version number of the Informed Consent	____________
The version number of the protocol shall apply when the Informed Consent is signed	__|__|.|__|

Hydroxychloroquine sulfate in Adult Patients With 2019-nCoV Respiratory Infection: A Multicenter, Randomized, Open-Label，Controlled Study

Case Report Form

Center Number ：	|__|__|
Subject Number ：	|__|__|__|__|__|
Abbreviation of Subject's Name：	|__|__|__|__|
Name of Center （regular script）：	____________________
Name of Doctor （regular script）：	____________________
The Version and Date ：	Version 3.0 /February 25,2020
Applicable Clinical Type ：	Severe type

CONFIDENTIAL

The information contained here relates to trade secrets and business information. These business information terms confidential content or proprietary information, except as required by regulations, shall not be disclosed by any person. Any individual who has access to the document shall be aware of its proprietary or confidential nature and shall not disclose the document without authorization. The restrictions on the confidentiality of the above documents will apply to any information provided to you in the future that is marked as proprietary or confidential.

Guide to Fill out the Form

1. The case report form shall be recorded by an authorized person, and each page in the CRF shall be carefully checked and signed.

2. The trial physicians should be famililar with the trial protocol and master the GCP principles.

3. Please write clearly and legibly with a sign pen in blue or black ink, preferably in capital letters for English,for example：CASE REPORT FORM

4. When filling in Numbers in open squares, fill in one number in each box；If the number of digits is less than the corresponding number of squares, please fill with zero.Spaces are not allowed.For example：56Kg，fill in the blanks as follows:| 0 | 5 | 6 |. | 0 | 0 |Kg.

5. The date is in the international ISO8601 date format：yyyymmdd. For example, December 21, 2007 was written as| 2 | 0 | 0 | 7 |YEAR| 1 | 2 |Month| 2 | 1 |DAY。

6. For the items with “□”in the form, please type “×”in the corresponding box, , or fill in the relevant Numbers. If not done or the data is not available, please fill in ND(Not Done) or NK(not known) in the corresponding space; NA is not applicable.

7. Each item must be filled in accurately, do not arbitrarily alter, do not use correction fluid or other ways to cover the original data. If you find any errors, please cross out the wrong record with a single line so that the original record is still legible,then fill in the correct record at the top right, indicating the name, abbreviation and date of modification.For example：58.6 56.8 ZH 2007-12-21.

8. The four of the Chinese phoneticize abbreviation of the patient's name should be filled in. Fill in the first two letters of each word for the two-character name; fill in the first letter of three words and the second letter of the third word or the three-character name; and fill in the first letter of each word for the four-character name. The chinese phoneticize abbreviation of each patient's name should be unified in their CRF. For example：Zhang Hong| Z | H | H | 0 |，Li Shuming| L | S |M | I |，Ou Yang Xiaohui| O | Y | X | H |.

9. Do not change the format of the case report form. If you find that there is no place in the form to fill in the information the recorder wishes to record, please contact the responsible CRA.

10. Upon completion of the case report form, the principal investigator shall examine each page of the case report form and sign at the investigator's statement.

Date of Current Visit： |__|__|__|__|Year|__|__|MONTH|__|__|DAY

**Sign the Informed Consent**	
Whether to sign informed consent	□0NO □1 YES
Date of signing the Informed Consent	|__|__|__|__|YEAR|__|__|MONTH|__|__|DAY
Version number of Informed Consent	________________
The version number of the protocol shall apply when the informed consent is signed	|__|__|.|__|
**Confirm Inclusion/Exclusion Criteria**	
Do subjects meet all inclusion criteria and not all exclusion criteria?	□1 YES □0 NO
□1 Does not meet the selection criteria, the selection criteria number (multiple choices)	□1□2 □3□4□5□6 □7<br>□Other，Please describe：__________
□2 Meet the exclusion criteria,the exclusion criteria number (multiple choices)	□1□2 □3□4□5□6 □7 □8 □9<br>□ Other，Please describe：_________

**Demographic Data**	
Gender	□1male □2female
Date of Birth	|__|__|__|__|YEAR|__|__|MONTH|__|__|DAY If not available, please state age：|__|__| years old
Nationality	□1Han □2Other，Please describe：__________________

**History of 2019-nCoV Respiratory Infection**	
Date of first onset	|__|__|__|__|YEAR|__|__|MONTH|__|__|DAY
History of exposure to related causes within 14 days prior to onset	□0NO □1YES □2 Unknown

Contact type (multiple choice)	□1 Contact with Hubei region<br>□2 Contact with person infected with 2019-nCoV<br>□3 Unknown<br>□4 Other，please describe ：______________________
Previous anti-2019-nCoV treatment or not	□0NO □1YES
Date of first positive nucleic acid test	|__|__|__|__|YEAR|__|__|MONTH|__|__|DAY
Clinical type at enrollment	□1Mild □2 Common □3 severe □4 critical
Note: If the subject ever had a treatment for 2019-nCoV pneumonia,please fill in the form "past/combined drug therapy" and "past/combined non-drug therapy".	

History of Drug Allergy

Does the subject have a history of drug allergy?
□0NO □1YES，Please describe ：______________________

____

Past/Concomitant Disease History

Does the subject have any past/concomitant disease other than 2019-nCoV pneumonia?
□0 NO □1 YES，Please fill in the form below.

Number	Disease	Confirmed Date	Disease persists or not?	End Date	Receive treatment or not?
1		|__|__|__|__|YEAR |__|__|MONTH| __|__|DAY	□0 NO □1 YES	|__|__|__|__|YEAR |__|__|MONTH| __|__|DAY	□0 NO □1 YES
2		|__|__|__|__|YEAR |__|__|MONTH| __|__|DAY	□0 NO □1 YES	|__|__|__|__|YEAR |__|__|MONTH| __|__|DAY	□0 NO □1 YES
3		|__|__|__|__|YEAR |__|__|MONTH| __|__|DAY	□0 NO □1 YES	|__|__|__|__|YEAR |__|__|MONTH| __|__|DAY	□0 NO □1 YES
4		|__|__|__|__|YEAR |__|__|MONTH| __|__|DAY	□0 NO □1 YES	|__|__|__|__|YEAR |__|__|MONTH| __|__|DAY	□0 NO □1 YES
5		|__|__|__|__|YEAR |__|__|MONTH| __|__|DAY	□0 NO □1 YES	|__|__|__|__|YEAR |__|__|MONTH| __|__|DAY	□0 NO □1 YES
6		|__|__|__|__|YEAR |__|__|MONTH| __|__|DAY	□0 NO □1 YES	|__|__|__|__|YEAR |__|__|MONTH| __|__|DAY	□0 NO □1 YES

7		|__|__|__|__|YEAR |__|__|MONTH| __|__|DAY	□0 NO □1 YES	|__|__|__|__|YEAR |__|__|MONTH| __|__|DAY	□0 NO □1 YES
8		|__|__|__|__|YEAR |__|__|MONTH| __|__|DAY	□0 NO □1 YES	|__|__|__|__|YEAR |__|__|MONTH| __|__|DAY	□0 NO □1 YES
9		|__|__|__|__|YEAR |__|__|MONTH| __|__|DAY	□0 NO □1 YES	|__|__|__|__|YEAR |__|__|MONTH| __|__|DAY	□0 NO □1 YES
10		|__|__|__|__|YEAR |__|__|MONTH| __|__|DAY	□0 NO □1 YES	|__|__|__|__|YEAR |__|__|MONTH| __|__|DAY	□0 NO □1 YES

**Vital Signs** □0 Unexamined，reason：________________________			
Date	|__|__|__|__|YEAR|__|__|MONTH|__|__|DAY		
Daily maximum temperature	|__|__|.|__|℃	Measurement method	□1 Axilla □2 Oral □3 Anus □4 Ear □5 Unknown □6 Other，please describe_______
Respiratory	|__|__|times/min	Pulse	|__|__|__|times/min
Blood pressure	|__|__|__|/|__|__|__| mmHg（Systolic/diastolic blood pressure）		

**Physical Examination** □0 Unexamined, reason:________________________					
Date	|__|__|__|__|YEAR|__|__|MONTH|__|__|DAY				
Inspection Part	Result<br>1= Normal<br>2= Abnormal, No Clinical significance<br>3= Abnormal, with Clinically significant<br>0=Unexamined				If abnormal，please describe
General appearance	□1	□2	□3	□0	
Head	□1	□2	□3	□0	
Eyes	□1	□2	□3	□0	

Nose	□1	□2	□3	□0	
Larynx	□1	□2	□3	□0	
Neck and thyroid	□1	□2	□3	□0	
Skin	□1	□2	□3	□0	
Cardiovascular system	□1	□2	□3	□0	
Chest and lungs	□1	□2	□3	□0	
Abdomen	□1	□2	□3	□0	
Nervous system	□1	□2	□3	□0	
Musculoskeletal system	□1	□2	□3	□0	
Lymphatic system	□1	□2	□3	□0	

Respiratory symptoms □0Unexamined, reason: ____________________

Date: |__|__|__|__|YEAR|__|__|MONTH|__|__|DAY

Whether the subject has respiratory symptoms or not: □0 NO □1 YES, Please describe: _____
____

Blood Count □0 Unexamined, reason____________________

Date: |__|__|__|__|YEAR|__|__|MONTH|__|__|DAY Normal range Number. |__|__|__|__|

Item	Result		Result Interpretation			Unexamined
	measured values	Unit	Normal	Abnormal: No Clinical significance	Abnormal: Clinically significant	
Red Blood Cell（RBC）		□1 10^{12}/L □2 Other: ___	□1	□2	□3	□0
White blood cell		□1 10^9/L □2 Other: ___	□1	□2	□3	□0
Hemoglobin（Hb）		□1 g/L □2 Other: ___	□1	□2	□3	□0
Platelet（PLT）		□1 10^9/L □2 Other: _____	□1	□2	□3	□0
Neutrophil（ANC）		□1 10^9/L □2 Other: _____	□1	□2	□3	□0
Lymphocyte（LY）		□1 10^9/L □2 Other: _____	□1	□2	□3	□0
Monocyte（MO）		□1 10^9/L □2 Other:	□1	□2	□3	□0

Eosinophil（EO）		□1 10^9/L □2 Other：_____	□1	□2	□3	□0
Basophil（BA）		□1 10^9/L □2 Other：_____	□1	□2	□3	□0

Blood Biochemistry □0 Unexamined, reason______________________________

Date：\|__\|__\|__\|__\|YEAR\|__\|__\|MONTH\|__\|__\|DAY Normal range Number：\|__\|__\|__\|__\|

Item	Result		Result Interpretation			Unexamined
	Measured value	Unit	Normal	Abnormal		
				No Clinical significance	Clinical significance	
Glutamic-pyruvic transaminase（ALT）		□1 U/L □2 Other：_____	□1	□2	□3	□0
Glutamic-oxalacetic transaminease（AST）		□1 U/L □2 Other：_____	□1	□2	□3	□0
Total bilirubin（TBIL）		□1 μmol/L □2 Other：_____	□1	□2	□3	□0
Albumin（ALB）		□1 g/L □2 Other：_____	□1	□2	□3	□0
γ- glutamyl transpeptidas（GGT）		□1 U/L □2 Other：_____	□1	□2	□3	□0
Lactic dehydrogenase（LDH）		□1 U/L □2 Other：_____	□1	□2	□3	□0
Creatine kinase（CK）		□1 U/L □2 Other：_____	□1	□2	□3	□0
Creatine kinase isoenzyme（CK-MB）		□1 U/L □2 Other：_____	□1	□2	□3	□0
Blood urea nitrogen（BUN）		□1 mmol/L □2 Other：_____	□1	□2	□3	□0
Urea（Urea）		□1 mmol/L □2 Other：_____	□1	□2	□3	□0
Serum creatinine（CR）		□1 μmol/L □2 Other：_____	□1	□2	□3	□0

Urine Test □0 Unexamined, reason______________________________

Date：\|__\|__\|__\|__\|YEAR\|__\|__\|MONTH\|__\|__\|DAY Normal range Number：\|__\|__\|__\|__\|

Item	Result		Result Interpretation			Unexamined
	measured value	Unit	Normal	Abnormal		
				No Clinical significance	Clinical significance	
Urine Protein		□1Not Applicable □2 Other：_____	□1	□2	□3	□0
Urine Glucose		□1 mmol/L □2 Other：_____	□1	□2	□3	□0
Urine PH value		□1 Not Applicable □2 Other：_____	□1	□2	□3	□0

Urine Erythrocyte		□1 Not Applicable □2 Other：_____	□1	□2	□3	□0
Urine Leukocyte		□1 Not Applicable □2 Other：_____	□1	□2	□3	□0
Urine Ketone		□1 Not Applicable □2 Other：_____	□1	□2	□3	□0

Coagulation Function □0 Unexamined, reason______________________________

Date：\|__\|__\|__\|__\|YEAR\|__\|__\|MONTH\|__\|__\|DAY Normal range Number：\|__\|__\|__\|__\|

Item	Result		Result Interpretation			Unexamined Unit
	measured value	Unit	Normal	Abnormal		
				No Clinical significance	Clinical significance	
ProthrombinTime（PT）		□1 S □2 Other：_____	□1	□2	□3	□0
Activated Partial Thromboplastin Time（APTT）		□1 S □2 Other：_____	□1	□2	□3	□0
Thrombin Time（TT）		□1 S □2 Other：_____	□1	□2	□3	□0
Fibrinogen（Fbg）		□1 g/L □2 Other：_____	□1	□2	□3	□0
International Normalized Ratio（INR）		□1 Not Applicable □2 Other：_____	□1	□2	□3	□0

C-Reactive Protein（CRP） □0 Unexamined, reason______________________________

Date：\|__\|__\|__\|__\|YEAR\|__\|__\|MONTH\|__\|__\|DAY Normal range Number：\|__\|__\|__\|__\|

Item	Result		Result Interpretation		
	Measured Value	Unit	Normal	Abnormal	
				No Clinical significance	Clinical significance
C-Reactive Protine（CRP）		□1 mg/L □2 Other：__	□1	□2	□3

Erythrocyte Sedimentation Rate（ESR） □0 Unexamined, reason______________________________

Date：\|__\|__\|__\|__\|YEAR\|__\|__\|MONTH\|__\|__\|DAY Normal range Number：\|__\|__\|__\|__\|

Item	Result		Result Interpretation		
	Measured Value	Unit	Normal	Abnormal	
				No Clinical significance	Clinical significance
Erythrocyte Sedimentation Rate（ESR）		□1 mm/h □2 Other：_____	□1	□2	□3

TNF-α □0 Unexamined, reason______________________________

Date：\|__\|__\|__\|__\|YEAR\|__\|__\|MONTH\|__\|__\|DAY Normal range Number：\|__\|__\|__\|__\|

Item	Result			Result Interpretation		
TNF-α	Measured Value	Unit		Normal	U	
					No Clinical significance	Clinical significance
		□1 pg/mL	□2 Other: _____	□1	□2	□3

**IL-6** □0Unexamined，reason______
Date：|__|__|__|__|YEAR|__|__|MONTH|__|__|DAY Normal range Number：|__|__|__|__|

Item	Result			Result Interpretation		
IL-6	Measured Value	Unit		Normal	Abnormal	
					No Clinical significance	Clinical significance
		□1 pg/mL	□2Other: _____	□1	□2	□3

**Urine or serum HCG（Only applicable to women of childbearing age）**<br>□0Unexamined，reason______<br>□1Not Applicable（Man or woman without fertility）
Date：|__|__|__|__|YEAR|__|__|MONTH|__|__|DAY
Sample：□1 Blood □2 Urine
Result：□1 Negative □2 Positive

**Routine 12 Leads Electrocardiogram** □0Unexamined，reason______
Date：|__|__|__|__|YEAR|__|__|MONTH|__|__|DAY
Result：□1 Normal □2 Abnormal，No Clinical significance □3 Abnormal，Clinically significant
If"Abnormal"，please describe：______

**Chest CT** □0Unexamined，reason______
Date：|__|__|__|__|YEAR|__|__|MONTH|__|__|DAY
Result：<br>□1 Normal □2 Abnormal，No Clinical significance □3 Abnormal，Clinical significanct
If abnormal，please describe ______

**Nucleic Acid Test of 2019-nCov** □0 Unexamined，reason______
Sampling Part（mutiple choice）：□1Upper respiratory tract □2Lower respiratory tract

Upper respiratory tract	Date: |__|__|__|__|YEAR|__|__|MONTH|__|__|DAY<br>Result: □1 Negative □2 Positive □3 Unidentified □4 Other, please describe ___________
Lower respiratory tract	Date: |__|__|__|__|YEAR|__|__|MONTH|__|__|DAY<br>Result: □1 Negative □2 Positive □3 Unidentified □4 Other, please describe ___________
Result	□1 Negative □2 Positive □3 Unidentified □4 Other, please describe ___________

**Pulse oxygen saturation (without oxygen inhalation)** □0Unexamined, reason_____
Date: |__|__|__|__|YEAR|__|__|MONTH|__|__|DAY
Measured Value: _____________% (Rest state)

**Arterial Blood Gas Analysis** □0Unexamined, reason________________________
Inhalate oxygen or not? □0 No □1Yes, (FiO2): _____________%
Date: |__|__|__|__|YEAR|__|__|MONTH|__|__|DAY

**Item**	**Result**		**Result Interpretation**			**Unexamined**
				**Abnormal**		
	**Measured Value**	**Unit**	**Normal**	**No Clinical significance**	**Significant clinically**	
Arterial partial pressure of oxygen (PaO2)		□1 mmHg □2 Other:_____	□1	□2	□3	□0
Oxygen saturation (SaO2)		□1 % □2 Other:_____	□1	□2	□3	□0

**Clinical status** □0 No evaluation, reason________________________	
Date:	|__|__|__|__|YEAR|__|__|MONTH|__|__|DAY
Result	□1 Level 1: discharge<br>□2 Level 2: hospitalization, oxygen therapy is not required<br>□3 Level 3: hospitalization, require oxygen therapy<br>□4 Level 4:ICU/ hospitalization, require noninvasive ventilation (NIV)/ High flow nasal catheter oxygen therapy (HFNC)<br>□5 Level 5:ICU, require extracorporeal membrane oxygenation

	(ECMO)and/or invasive mechanical ventilation(IMV)<br>☐6 Level 6:Death

**Screening Result** □0 Not Evaluated, reason________________________________	
Is the subject screened successfully?<br>□0 NO, the main cause of failure screening：______________________________<br>□1 YES, please randomize	
Randomize Date：|__|__|__|__|YEAR|__|__|MONTH|__|__|DAY	
Randomize Number：	
Randomized Group	□1 Experimental group □2 Control group

If any adverse events have occurred since the signing of the informed consent, please fill in the form of "adverse events"; If there is medication combination, please fill in the form "previous/combined medication"; If there is combined non-drug therapy, please fill in the "previous/combined non-drug therapy" form; If there is an out-of-plan inspection, please fill in the "out-of-plan inspection" form; If there is IMV/NIV, ECMO, HFNC, oxygen therapy, please fill in the "IMV/NIV, ECMO, HFNC, oxygen therapy" form.

Date of Current Visit：|__|__|__|__|YEAR|__|__|MONTH|__|__|DAY

**Vital Signs** □0Unexamined，reason________			
Date	|__|__|__|__|YEAR|__|__|MONTH|__|__|DAY		
Daily maximum temperature	|__|__|.|__|℃	Measurement method	□1 Axilla □2Oral □3 Anus □4 Ear □5 Unknown □6 Other，please describe________
Respiratory	|__|__|times/min	Pulse	|__|__|__|times/min
Blood pressure	|__|__|__|/|__|__|__| mmHg（Systolic/diastolic blood pressure）		

**Respiratory symptoms** □0Unexamined，reason________
Date：|__|__|__|__|YEAR|__|__|MONTH|__|__|DAY
Whether the subject has respiratory symptoms or not：□0 NO □1 YES，please describe：________
Result：□1 Normal □2 Abnormal,No Clinical significance □3 Abnormal,Clinically significant

Blood Count □0Unexamined，reason________

Date：|__|__|__|__|YEAR|__|__|MONTH|__|__|DAY Normal range Number：|__|__|__|__|

Item	Result		Result Interpretation			Unexamined
	Measured Value	Unit	Normal	Abnormal: No Clinical significance	Abnormal: Clinical significance	
Red Blood Cell（RBC）		□1 10^{12}/L □2Other：_____	□1	□2	□3	□0
White Blood Cell		□1 10^9/L □2Other：_____	□1	□2	□3	□0
Hemoglobin（Hb）		□1 g/L □2Other：_____	□1	□2	□3	□0
Platelet（PLT）		□1 10^9/L □2Other：_____	□1	□2	□3	□0
Neutrophil（ANC）		□1 10^9/L □2Other：_____	□1	□2	□3	□0
Lymphocyte（LY）		□1 10^9/L □2Other：_____	□1	□2	□3	□0
Monocyte（MO）		□1 10^9/L □2Other：_____	□1	□2	□3	□0
Eosinophil（EO）		□1 10^9/L □2Other：_____	□1	□2	□3	□0
Basophil（BA）		□1 10^9/L □2Other：_____	□1	□2	□3	□0

Blood Biochemistry □0Unexamined，reason________

Date\|__\|__\|__\|__\|YEAR\|__\|__\|MONTH\|__\|__\|DAY Normal range Number：\|__\|__\|__\|__\|

Item	Result		Result Interpretation			Unexamined
	Measured Value	Unit	Normal	Abnormal: No Clinical significance	Abnormal: Clinical significance	
Glutamic-pyruvic transaminase（ALT）		□1 U/L □2Other：_____	□1	□2	□3	□0
Glutamic-oxalacetic transaminease（AST）		□1 U/L □2Other：_____	□1	□2	□3	□0
Total Bilirubin（TBIL）		□1 μmol/L □2Other：_____	□1	□2	□3	□0
Albumin（ALB）		□1 g/L □2Other：_____	□1	□2	□3	□0
γ- glutamyl transpeptidas（GGT）		□1 U/L □2Other：_____	□1	□2	□3	□0
Lactic dehydrogenase（LDH）		□1 U/L □2Other：_____	□1	□2	□3	□0
Creatine kinase（CK）		□1 U/L □2Other：_____	□1	□2	□3	□0
Creatine kinase isoenzyme（CK-MB）		□1 U/L □2Other：_____	□1	□2	□3	□0
Blood urea nitrogen（BUN）		□1 mmol/L □2Other：_____	□1	□2	□3	□0
Urea（Urea）		□1 mmol/L □2Other：_____	□1	□2	□3	□0
Serum creatinine（CR）		□1 μmol/L □2Other：_____	□1	□2	□3	□0

Urine Test □0Unexamined，reason________________________________
□1 Not Applicable

Date：\|__\|__\|__\|__\|YEAR\|__\|__\|MONTH\|__\|__\|DAY Normal range Number：\|__\|__\|__\|__\|

Item	Result		Result Interpretation			Unexamined
	Measured Value	Unit	Normal	Abnormal: No Clinical significance	Abnormal: Clinical significance	
Urine Protein		□1Not Applicable □2Other：_____	□1	□2	□3	□0
Urine Glucose		□1 mmol/L □2Other：_____	□1	□2	□3	□0
Urine PH value		□1 Not Applicable □2Other：_____	□1	□2	□3	□0
Urine Erythrocyte		□1Not Applicable □2Other：_____	□1	□2	□3	□0
Urine Leukocyte		□1Not Applicable □2Other：_____	□1	□2	□3	□0
Urine Ketone		□1Not Applicable □2Other：_____	□1	□2	□3	□0

Coagulation Function □0Unexamined，reason________

Date：\|__\|__\|__\|__\|YEAR\|__\|__\|MONTH\|__\|__\|DAY Normal range Number：\|__\|__\|__\|__\|

Item	Result		Result Interpretation			Unexamined
				Abnormal		
	Measured Value	Unit	Normal	No Clinical significance	Clinical significance	
ProthrombinTime（PT）		□1 S □2Other：_____	□1	□2	□3	□0
Activated Partial Thromboplastin Time（APTT）		□1 S □2Other：_____	□1	□2	□3	□0
Thrombin Time（TT）		□1 S □2Other：_____	□1	□2	□3	□0
Fibrinogen（Fbg）		□1 g/L □2Other：_____	□1	□2	□3	□0
International Normalized Ratio（INR）		□1Not Applicable □2Other：_____	□1	□2	□3	□0

C-REACTIVE PROTEIN（CRP） □0Unexamined，reason________

Date：\|__\|__\|__\|__\|YEAR\|__\|__\|MONTH\|__\|__\|DAY Normal range Number：\|__\|__\|__\|__\|

Item	Result		Result Interpretation		
				Abnormal	
	Measured Value	Unit	Normal	No Clinical significance	Clinical significance
C-REACTIVE PROTEIN（CRP）		□1 mg/L □2Other：_____	□1	□2	□3

Erythrocyte Sedimentation Rate（ESR） □0Unexamined，reason________

Date：\|__\|__\|__\|__\|YEAR\|__\|__\|MONTH\|__\|__\|DAY Normal range Number：\|__\|__\|__\|__\|

Item	Result		Result Interpretation		
				Abnormal	
	Measured Value	Unit	Normal	No Clinical significance	Clinical significance
Erythrocyte Sedimentation Rate（ESR）		□1 mm/h □2Other：_____	□1	□2	□3

TNF-α □0Unexamined，reason________

Date：\|__\|__\|__\|__\|YEAR\|__\|__\|MONTH\|__\|__\|DAY Normal range Number：\|__\|__\|__\|__\|

Item	Result			Result Interpretation		
					Abnormal	
	Measured Value	Unit		Normal	No Clinical significance	Clinical significance
TNF-α		□1 pg/mL	□2Other：_____	□1	□2	□3

IL-6	□0Unexamined，reason______					
Date：|__|__|__|__|YEAR|__|__|MONTH|__|__|DAY			Normal range Number：|__|__|__|__|__|			
**Item**	**Result**			**Result Interpretation**		
					**Abnormal**	
	**Measured Value**	**Unit**		**Normal**	**No Clinical significance**	**Clinical significance**
IL-6		□1 pg/mL	□2Other：_____	□1	□2	□3

Nucleic Acid Test of 2019-nCov　　□0 Unexamined，reason______ □1 Not Applicable
Sampling Part：□1Upper respiratory tract　□2Lower respiratory tract
Date：|__|__|__|__|YEAR|__|__|MONTH|__|__|DAY　　Date：|__|__|:|__|__|
Result：□1 Negative □2 Positive □3 Unidentified □4 Other，please describe______

Pulse oxygen saturation（without oxygen inhalation）　□0Unexamined，reason_____
Date：|__|__|__|__|YEAR|__|__|MONTH|__|__|DAY
Measured Value：_____________%（Rest state）

Arterial blood gas analysis　□0Unexamined，reason______ □1 Not applicable							
Oxygen inhalation：□1No　□2Yes, FiO2:_____________%							
Date：|__|__|__|__|YEAR|__|__|MONTH|__|__|DAY<br>Normal range Number：|__|__|__|__|__|							
**Item**	**Result**			**Result Interpretation**			**Unexamined**
					**Abnormal**		
	**Measured Value**	**Unit**		**Normal**	**No Clinical significance**	**Clinical significance**	
PaO2		□1 mmHg	□2 Other：_____	□1	□2	□3	□0
SaO2		□1 %	□2 Other：	□1	□2	□3	□0

If any adverse events have occurred since the last visit, please fill in the “adverse events” form. If there is medication combination, please fill in the “previous/combined medication” form; If there is combined non-drug therapy, please fill in the “combined non-drug therapy” form; If there is an out-of-plan inspection, please fill in the “out-of-plan inspection” form.

Date of Current Visit： \|__\|__\|__\|__\|YEAR\|__\|__\|MONTH\|__\|__\|DAY

**Vital Signs**	□0Unexamined，reason________		
Date	|__|__|__|__|YEAR|__|__|MONTH|__|__|DAY		
Daily maximum temperature	|__|__|.|__|℃	Measurement method	□1 Axilla □2 Oral □3 Anus □4 Ear □5 Unknown □6 Other，please describe________
Respiratory	|__|__|times/min	Pulse	|__|__|__|times/min
Blood pressure	|__|__|__|/|__|__|__| mmHg（Systolic/diastolic blood pressure）		

**Respiratory symptoms** □0Unexamined，reason________
Date：|__|__|__|__|YEAR|__|__|MONTH|__|__|DAY
Whether the subject has respiratory symptoms or not：□0 NO □1 YES，please describe：________
Result：□1 Normal □2 Abnormal,No Clinical significance □3 Abnormal,Clinically significant

Blood Count □0Unexamined，reason________

Date：\|__\|__\|__\|__\|YEAR\|__\|__\|MONTH\|__\|__\|DAY Normal range Number：\|__\|__\|__\|__\|__\|

Item	Result		Result Interpretation			Unexamined
				Abnormal		
	Measured Value	Unit	Normal	No Clinical significance	Clinically significant	
Red Blood Cell（RBC）		□1 10^{12}/L □2Other：_____	□1	□2	□3	□0
White Blood Cell		□1 10^{9}/L □2Other：_____	□1	□2	□3	□0
Hemoglobin（Hb）		□1 g/L □2Other：_____	□1	□2	□3	□0
Platelet（PLT）		□1 10^{9}/L □2Other：_____	□1	□2	□3	□0
Neutrophil（ANC）		□1 10^{9}/L □2Other：_____	□1	□2	□3	□0
Lymphocyte（LY）		□1 10^{9}/L □2Other：_____	□1	□2	□3	□0
Monocyte（MO）		□1 10^{9}/L □2Other：_____	□1	□2	□3	□0
Eosinophil（EO）		□1 10^{9}/L □2Other：_____	□1	□2	□3	□0
Basophil（BA）		□1 10^{9}/L □2Other：_____	□1	□2	□3	□0

Blood Biochemistry □0Unexamined，reason________

Date\|__\|__\|__\|__\|YEAR\|__\|__\|MONTH\|__\|__\|DAY Normal range Number：\|__\|__\|__\|__\|__\|

Item	Result		Result Interpretation			Unexamined
	Measured Value	Unit	Normal	Abnormal: No Clinical significance	Abnormal: Clinically significant	
Glutamic-pyruvic transaminase（ALT）		□1 U/L □2Other：_____	□1	□2	□3	□0
Glutamic-oxalacetic transaminease（AST）		□1 U/L □2Other：_____	□1	□2	□3	□0
Total Bilirubin（TBIL）		□1 μmol/L □2Other：_____	□1	□2	□3	□0
Albumin（ALB）		□1 g/L □2Other：_____	□1	□2	□3	□0
γ- glutamyl transpeptidas（GGT）		□1 U/L □2Other：_____	□1	□2	□3	□0
Lactic dehydrogenase（LDH）		□1 U/L □2Other：_____	□1	□2	□3	□0
Creatine kinase（CK）		□1 U/L □2Other：_____	□1	□2	□3	□0
Creatine kinase isoenzyme（CK-MB）		□1 U/L □2Other：_____	□1	□2	□3	□0
Blood urea nitrogen（BUN）		□1 mmol/L □2Other：_____	□1	□2	□3	□0
Urea（Urea）		□1 mmol/L □2Other：_____	□1	□2	□3	□0
Serum creatinine（CR）		□1 μmol/L □2Other：_____	□1	□2	□3	□0

Coagulation Function □0Unexamined，reason________________________

Date：\|__\|__\|__\|__\|YEAR\|__\|__\|MONTH\|__\|__\|DAY Normal range Number：\|__\|__\|__\|__\|__\|

Item	Result		Result Interpretation			Unexamined
	Measured Value	Unit	Normal	Abnormal: No Clinical significance	Abnormal: Clinically significant	
ProthrombinTime（PT）		□1 S □2Other：_____	□1	□2	□3	□0
Activated Partial Thromboplastin Time（APTT）		□1 S □2Other：_____	□1	□2	□3	□0
Thrombin Time（TT）		□1 S □2Other：_____	□1	□2	□3	□0
Fibrinogen（Fbg）		□1 g/L □2Other：_____	□1	□2	□3	□0
International Normalized Ratio（INR）		□1Not Applicable □2Other：_____	□1	□2	□3	□0

C-REACTIVE PROTEIN（CRP） □0Unexamined，reason________________________

Date：\|__\|__\|__\|__\|YEAR\|__\|__\|MONTH\|__\|__\|DAY Normal range Number：\|__\|__\|__\|__\|__\|

Item	Result		Result Interpretation		
				Abnormal	
C-REACTIVE PROTEIN（CRP）	Measured Value	Unit	Normal	No Clinical significance	Clinically significant
		□1 mg/L □2Other：_____	□1	□2	□3

Erythrocyte Sedimentation Rate（ESR） □0Unexamined，reason________________

Date：\|__\|__\|__\|__\|YEAR\|__\|__\|MONTH\|__\|__\|DAY Normal range Number：\|__\|__\|__\|__\|

Item	Result		Result Interpretation		
				Abnormal	
Erythrocyte Sedimentation Rate（ESR）	Measured Value	Unit	Normal	No Clinical significance	Clinically significant
		□1 mm/h □2Other：_____	□1	□2	□3

TNF-α □0Unexamined，reason________________

Date：\|__\|__\|__\|__\|YEAR\|__\|__\|MONTH\|__\|__\|DAY Normal range Number：\|__\|__\|__\|__\|

Item	Result			Result Interpretation		
					Abnormal	
TNF-α	Measured Value	Unit		Normal	No Clinical significance	Clinically significant
		□1 pg/mL	□2Other：_____	□1	□2	□3

IL-6 □0Unexamined，reason________________

Date：\|__\|__\|__\|__\|YEAR\|__\|__\|MONTH\|__\|__\|DAY Normal range Number：\|__\|__\|__\|__\|

Item	Result			Result Interpretation		
					Abnormal	
IL-6	Measured Value	Unit		Normal	No Clinical significance	Clinically significant
		□1 pg/mL	□2Other：_____	□1	□2	□3

Nucleic Acid Test of 2019-nCov □0 Unexamined，reason________________

Sampling Part（mutiple choice）：□1Upper respiratory tract □2Lower respiratory tract

Upper respiratory tract	Date：|__|__|__|__|YEAR|__|__|MONTH|__|__|DAY<br>Result：□1 Negative □2 Positive □3 Unidentified □4 Other，please describe ___________

Lower respiratory tract	Date：|__|__|__|__|YEAR|__|__|MONTH|__|__|DAY<br>Result：□1 Negative □2 Positive □3 Unidentified □4 Other，please describe ___________
Result	□1 Negative □2 Positive □3 Unidentified □4 Other， please describe ___________

**Pulse oxygen saturation（without oxygen inhalation ）** □0Unexamined，reason_____
Date：|__|__|__|__|YEAR|__|__|MONTH|__|__|DAY
Measured Value： _____________%（Rest state）

**Arterial blood gas analysis** □0Unexamined，reason______________________________ □1 Not applicable
Oxygen inhalation：□1No □2Yes, FiO2:_____________%
Date：|__|__|__|__|YEAR|__|__|MONTH|__|__|DAY<br>Normal range Number：|__|__|__|__|__|

Item	Result		Result Interpretation			Unexamined
				Abnormal		
	Measured Value	Unit	Normal	No Clinical significance	Clinical significance	
PaO2		□1 mmHg □2 Other：_____	□1	□2	□3	□0
SaO2		□1 % □2 Other：	□1	□2	□3	□0

**Clinical status** □0 No evaluation，reason____	
Date：	|__|__|__|__|YEAR|__|__|MONTH|__|__|DAY
Result	□1 Level 1: discharge<br>□2 Level 2: hospitalization，oxygen therapy is not required<br>□3 Level 3: hospitalization，require oxygen therapy<br>□4 Level 4:ICU/ hospitalization，require noninvasive ventilation (NIV)/ High flow nasal catheter oxygen therapy (HFNC)<br>□5 Level 5:ICU，require extracorporeal membrane oxygenation (ECMO)and/or invasive mechanical ventilation(IMV)<br>□6 Level 6:Death

If any adverse events have occurred since the last visit, please fill in the “adverse events” form. If there is medication combination, please fill in the “previous/combined medication” form; If there is combined non-drug therapy, please fill in the “combined non-drug therapy” form; If there is an out-of-plan inspection, please fill in the “out-of-plan inspection” form.

Date of Current Visit： |__|__|__|__|YEAR|__|__|MONTH|__|__|DAY

**Vital Signs**	□0Unexamined, reason__________		
Date	|__|__|__|__|YEAR|__|__|MONTH|__|__|DAY		
Daily maximum temperature	|__|__|.|__|℃	Measurement method	□1 Axilla □2Oral □3 Anus<br>□4 Ear □5 Unknown □6 Other, please describe________
Respiratory	|__|__|times/min	Pulse	|__|__|__|times/min
Blood pressure	|__|__|__|/|__|__|__| mmHg （Systolic/diastolic blood pressure）		

**Respiratory symptoms** □0Unexamined, reason__________
Date: |__|__|__|__|YEAR|__|__|MONTH|__|__|DAY
Whether the subject has respiratory symptoms or not: □0 NO □1 YES, please describe: ____ __________
Result: □1 Normal □2 Abnormal,No Clinical significance □3 Abnormal,Clinically significant

Blood Count □0Unexamined, reason__________

Date: |__|__|__|__|YEAR|__|__|MONTH|__|__|DAY Normal range Number: |__|__|__|__|__|

Item	Result		Result Interpretation			Unexamined
	Measured Value	Unit	Normal	Abnormal: No Clinical significance	Abnormal: Clinically significant	
Red Blood Cell （RBC）		□1 10^{12}/L □2Other: _____	□1	□2	□3	□0
White Blood Cell		□1 10^{9}/L □2Other: _____	□1	□2	□3	□0
Hemoglobin （Hb）		□1 g/L □2Other: _____	□1	□2	□3	□0
Platelet （PLT）		□1 10^{9}/L □2Other: _____	□1	□2	□3	□0
Neutrophil （ANC）		□1 10^{9}/L □2Other: _____	□1	□2	□3	□0
Lymphocyte （LY）		□1 10^{9}/L □2Other: _____	□1	□2	□3	□0
Monocyte （MO）		□1 10^{9}/L □2Other: _____	□1	□2	□3	□0
Eosinophil （EO）		□1 10^{9}/L □2Other: _____	□1	□2	□3	□0
Basophil （BA）		□1 10^{9}/L □2Other: _____	□1	□2	□3	□0

Blood Biochemistry □0Unexamined, reason__________

Date|__|__|__|__|YEAR|__|__|MONTH|__|__|DAY Normal range Number：|__|__|__|__|

Item	Result		Result Interpretation			Unexamined
	Measured Value	Unit	Normal	Abnormal: No Clinical significance	Abnormal: Clinically significant	
Glutamic-pyruvic transaminase（ALT）		□1 U/L □2Other：_____	□1	□2	□3	□0
Glutamic-oxalacetic transaminease（AST）		□1 U/L □2Other：_____	□1	□2	□3	□0
Total Bilirubin（TBIL）		□1 μmol/L □2Other：_____	□1	□2	□3	□0
Albumin（ALB）		□1 g/L □2Other：_____	□1	□2	□3	□0
γ- glutamyl transpeptidas（GGT）		□1 U/L □2Other：_____	□1	□2	□3	□0
Lactic dehydrogenase（LDH）		□1 U/L □2Other：_____	□1	□2	□3	□0
Creatine kinase（CK）		□1 U/L □2Other：_____	□1	□2	□3	□0
Creatine kinase isoenzyme（CK-MB）		□1 U/L □2Other：_____	□1	□2	□3	□0
Blood urea nitrogen（BUN）		□1 mmol/L □2Other：_____	□1	□2	□3	□0
Urea（Urea）		□1 mmol/L □2Other：_____	□1	□2	□3	□0
Serum creatinine（CR）		□1 μmol/L □2Other：_____	□1	□2	□3	□0

Coagulation Function □0Unexamined，reason________________________________

Date：|__|__|__|__|YEAR|__|__|MONTH|__|__|DAY Normal range Number：|__|__|__|__|

Item	Result		Result Interpretation			Unexamined
	Measured Value	Unit	Normal	Abnormal: No Clinical significance	Abnormal: Clinically significant	
ProthrombinTime（PT）		□1 S □2Other：_____	□1	□2	□3	□0
Activated Partial Thromboplastin Time（APTT）		□1 S □2Other：_____	□1	□2	□3	□0
Thrombin Time（TT）		□1 S □2Other：_____	□1	□2	□3	□0
Fibrinogen（Fbg）		□1 g/L □2Other：_____	□1	□2	□3	□0
International Normalized Ratio（INR）		□1Not Applicable □2Other：_____	□1	□2	□3	□0

C-REACTIVE PROTEIN（CRP） □0Unexamined，reason________________________________

Date：|__|__|__|__|YEAR|__|__|MONTH|__|__|DAY Normal range Number：|__|__|__|__|

Item	Result		Result Interpretation		
C-REACTIVE PROTEIN（CRP）	Measured Value	Unit	Normal	Abnormal: No Clinical significance	Abnormal: Clinically significant
		□1 mg/L □2Other: _____	□1	□2	□3

Erythrocyte Sedimentation Rate（ESR） □0Unexamined，reason______________________

Date：\|__\|__\|__\|__\|YEAR\|__\|__\|MONTH\|__\|__\|DAY Normal range Number：\|__\|__\|__\|__\|

Item	Result		Result Interpretation		
Erythrocyte Sedimentation Rate（ESR）	Measured Value	Unit	Normal	Abnormal: No Clinical significance	Abnormal: Clinically significant
		□1 mm/h □2Other: _____	□1	□2	□3

TNF-α □0Unexamined，reason______________________

Date：\|__\|__\|__\|__\|YEAR\|__\|__\|MONTH\|__\|__\|DAY Normal range Number：\|__\|__\|__\|__\|

Item	Result			Result Interpretation		
TNF-α	Measured Value	Unit		Normal	Abnormal: No Clinical significance	Abnormal: Clinically significant
		□1 pg/mL	□2Other: _____	□1	□2	□3

IL-6 □0Unexamined，reason______________________

Date：\|__\|__\|__\|__\|YEAR\|__\|__\|MONTH\|__\|__\|DAY Normal range Number：\|__\|__\|__\|__\|

Item	Result			Result Interpretation		
IL-6	Measured Value	Unit		Normal	Abnormal: No Clinical significance	Abnormal: Clinically significant
		□1 pg/mL	□2Other: _____	□1	□2	□3

Nucleic Acid Test of 2019-nCov □0 Unexamined，reason______________________

Sampling Part（mutiple choice）: □1Upper respiratory tract □2Lower respiratory tract

Upper respiratory tract	Date：|__|__|__|__|YEAR|__|__|MONTH|__|__|DAY<br>Result：□1 Negative □2 Positive □3 Unidentified □4 Other，please describe ___________
Lower respiratory tract	Date：|__|__|__|__|YEAR|__|__|MONTH|__|__|DAY<br>Result：□1 Negative □2 Positive □3 Unidentified □4 Other，please describe ___________
Result	□1 Negative □2 Positive □3 Unidentified □4 Other，please describe ___________

**Pulse oxygen saturation (without oxygen inhalation)** □0Unexamined, reason______
Date: |__|__|__|__|YEAR|__|__|MONTH|__|__|DAY
Measured Value: ______________% (Rest state)

**Arterial blood gas analysis** □0Unexamined, reason________________________________ □1 Not applicable
Oxygen inhalation: □1No □2Yes, FiO2:_____________%
Date: |__|__|__|__|YEAR|__|__|MONTH|__|__|DAY<br>Normal range Number: |__|__|__|__|

Item	Result		Result Interpretation			Unexamined
	Measured Value	Unit	Normal	Abnormal		
				No Clinical significance	Clinical significance	
PaO2		□1 mmHg □2 Other: _____	□1	□2	□3	□0
SaO2		□1 % □2 Other:	□1	□2	□3	□0

If any adverse events have occurred since the last visit, please fill in the "adverse events" form. If there is medication combination, please fill in the "previous/combined medication" form; If there is combined non-drug therapy, please fill in the "combined non-drug therapy" form; If there is an out-of-plan inspection, please fill in the "out-of-plan inspection" form;If there is IMV/NIV、ECMO、HFNC、oxygen therapy, please fill in the "IMV/NIV、ECMO、HFNC、oxygen therapy" form.

"

**Date of Current Visit：** |__|__|__|__|YEAR|__|__|MONTH|__|__|DAY			
**Vital Signs** □0Unexamined，reason________			
Date	|__|__|__|__|YEAR|__|__|MONTH|__|__|DAY		
Daily maximum temperature	|__|__|.|__|℃	Measurement method	□1 Axilla □2Oral □3 Anus □4 Ear □5 Unknown □6 Other，please describe________
Respiratory Rate	|__|__|times/min	Pulse	|__|__|__|times/min
Blood pressure	|__|__|__|/|__|__|__| mmHg（Systolic/diastolic blood pressure）		

**Respiratory symptoms** □0Unexamined，reason________
Date：|__|__|__|__|YEAR|__|__|MONTH|__|__|DAY
Whether the subject has respiratory symptoms or not：□0 NO □1 YES，please describe：________
Result：□1 Normal □2 Abnormal,No Clinical significance □3 Abnormal,Clinically significant

**Blood Count** □0Unexamined，reason________						
Date：|__|__|__|__|YEAR|__|__|MONTH|__|__|DAY Normal range Number：|__|__|__|__|__|						
**Item**	**Result**		**Result Interpretation**			**Unexamined**
				**Abnormal**		
	**Measured Value**	**Unit**	**Normal**	**No Clinical significance**	**Clinically significant**	
Red Blood Cell（RBC）		□1 10^{12}/L □2Other：_____	□1	□2	□3	□0
White Blood Cell		□1 10^{9}/L □2Other：_____	□1	□2	□3	□0
Hemoglobin（Hb）		□1 g/L □2Other：_____	□1	□2	□3	□0
Platelet（PLT）		□1 10^{9}/L □2Other：_____	□1	□2	□3	□0
Neutrophil（ANC）		□1 10^{9}/L □2Other：_____	□1	□2	□3	□0
Lymphocyte（LY）		□1 10^{9}/L □2Other：_____	□1	□2	□3	□0
Monocyte（MO）		□1 10^{9}/L □2Other：_____	□1	□2	□3	□0
Eosinophil（EO）		□1 10^{9}/L □2Other：_____	□1	□2	□3	□0
Basophil（BA）		□1 10^{9}/L □2Other：_____	□1	□2	□3	□0

**Blood Biochemistry** □0Unexamined，reason________
Date|__|__|__|__|YEAR|__|__|MONTH|__|__|DAY Normal range Number：|__|__|__|__|__|

Item	Result		Result Interpretation			Unexamined
	Measured Value	Unit	Normal	Abnormal		
				No Clinical significance	Clinically significant	
Glutamic-pyruvic transaminase（ALT）		□1 U/L □2Other：_____	□1	□2	□3	□0
Glutamic-oxalacetic transaminease（AST）		□1 U/L □2Other：_____	□1	□2	□3	□0
Total Bilirubin（TBIL）		□1 μmol/L □2Other：_____	□1	□2	□3	□0
Albumin（ALB）		□1 g/L □2Other：_____	□1	□2	□3	□0
γ- glutamyl transpeptidas（GGT）		□1 U/L □2Other：_____	□1	□2	□3	□0
Lactic dehydrogenase（LDH）		□1 U/L □2Other：_____	□1	□2	□3	□0
Creatine kinase（CK）		□1 U/L □2Other：_____	□1	□2	□3	□0
Creatine kinase isoenzyme（CK-MB）		□1 U/L □2Other：_____	□1	□2	□3	□0
Blood urea nitrogen（BUN）		□1 mmol/L □2Other：_____	□1	□2	□3	□0
Urea（Urea）		□1 mmol/L □2Other：_____	□1	□2	□3	□0
Serum creatinine（CR）		□1 μmol/L □2Other：_____	□1	□2	□3	□0

Coagulation Function □0Unexamined，reason________________

Date：|__|__|__|__|YEAR|__|__|MONTH|__|__|DAY Normal range Number：|__|__|__|__|

Item	Result		Result Interpretation			Unexamined
	Measured Value	Unit	Normal	Abnormal		
				No Clinical significance	Clinically significant	
ProthrombinTime（PT）		□1 S □2Other：_____	□1	□2	□3	□0
Activated Partial Thromboplastin Time（APTT）		□1 S □2Other：_____	□1	□2	□3	□0
Thrombin Time（TT）		□1 S □2Other：_____	□1	□2	□3	□0
Fibrinogen（Fbg）		□1 g/L □2Other：_____	□1	□2	□3	□0
International Normalized Ratio（INR）		□1Not Applicable □2Other：_____	□1	□2	□3	□0

C-REACTIVE PROTEIN（CRP） □0Unexamined，reason________________

Date：|__|__|__|__|YEAR|__|__|MONTH|__|__|DAY Normal range Number：|__|__|__|__|

Item	Result		Result Interpretation	
	Measured	Unit	Normal	Abnormal

C-REACTIVE PROTEIN (CRP)	Value		l	No Clinical significance	Clinically significant
		□1 mg/L □2Other: _____	□1	□2	□3

**Erythrocyte Sedimentation Rate (ESR)** □0Unexamined, reason________________					
Date: |__|__|__|__|YEAR|__|__|MONTH|__|__|DAY Normal range Number: |__|__|__|__|					
**Item**	**Result**		**Result Interpretation**		
Erythrocyte Sedimentation Rate (ESR)	**Measured Value**	**Unit**	**Normal**	**Abnormal**	
				**No Clinical significance**	**Clinically significant**
		□1 mm/h □2Other: _____	□1	□2	□3

**TNF-α** □0Unexamined, reason________________						
Date: |__|__|__|__|YEAR|__|__|MONTH|__|__|DAY Normal range Number: |__|__|__|__|						
**Item**	**Result**			**Result Interpretation**		
TNF-α	**Measured Value**	**Unit**		**Normal**	**Abnormal**	
					**No Clinical significance**	**Clinically significant**
		□1 pg/mL	□2Other: _____	□1	□2	□3

**IL-6** □0Unexamined, reason________________						
Date: |__|__|__|__|YEAR|__|__|MONTH|__|__|DAY Normal range Number: |__|__|__|__|						
**Item**	**Result**			**Result Interpretation**		
IL-6	**Measured Value**	**Unit**		**Normal**	**Abnormal**	
					**No Clinical significance**	**Clinically significant**
		□1 pg/mL	□2Other: _____	□1	□2	□3

**Nucleic Acid Test of 2019-nCov** □0 Unexamined, reason________________	
Sampling Part (mutiple choice): □1Upper respiratory tract □2Lower respiratory tract	
Upper respiratory tract	Date: |__|__|__|__|YEAR|__|__|MONTH|__|__|DAY<br>Result: □1 Negative □2 Positive □3 Unidentified □4 Other, please describe ___________
Lower respiratory tract	Date: |__|__|__|__|YEAR|__|__|MONTH|__|__|DAY<br>Result: □1 Negative □2 Positive □3 Unidentified □4 Other, please describe ___________
Result	□1 Negative □2 Positive □3 Unidentified □4 Other, please describe ___________

**Pulse oxygen saturation（without oxygen inhalation ）** □0Unexamined，reason_____
Date：|__|__|__|__|YEAR|__|__|MONTH|__|__|DAY
Measured Value： ____________%（Rest state）

**Arterial blood gas analysis** □0Unexamined，reason____________________________ □1 Not applicable
Oxygen inhalation：□1No □2Yes, FiO2:____________%
Date：|__|__|__|__|YEAR|__|__|MONTH|__|__|DAY<br>Normal range Number：|__|__|__|__|

Item	Result		Result Interpretation			Unexamined
	Measured Value	Unit	Normal	Abnormal		
				No Clinical significance	Clinical significance	
PaO2		□1 mmHg □2 Other：_____	□1	□2	□3	□0
SaO2		□1 % □2 Other：	□1	□2	□3	□0

**Clinical state** □0Unexamined，reason______________________________________	
Date	|__|__|__|__|YEAR|__|__|MONTH|__|__|DAY
Result	□1 Level 1: Discharge<br>□2 Level 2: Hospitalization, no requirment for oxygen therapy<br>□3 Level 3: Hospitalization, requiring oxygen therapy (non-NIV / HFNC)<br>□4 Level 4: ICU / Hospitalization, requiring non-invasive mechanical ventilation (NIV) / High Flow Nasal Catheter Oxygen Therapy (HFNC)<br>□5 Level 5: ICU, requiring extracorporeal membrane oxygenation (ECMO) and / or invasive mechanical ventilation (IMV)<br>□6 Level 6: Death

If any adverse events have occurred since the last visit, please fill in the “adverse events” form. If there is medication combination, please fill in the “previous/combined medication” form; If there is combined non-drug therapy, please fill in the “combined non-drug therapy” form; If there is an out-of-plan inspection, please fill in the “out-of-plan inspection” form; If there is IMV/NIV、ECMO、HFNC、oxygen therapy, please fill in the “IMV/NIV、ECMO、HFNC、oxygen therapy” form.

Date of Current Visit： \|__\|__\|__\|__\|YEAR\|__\|__\|MONTH\|__\|__\|DAY

**Vital Signs**	□0Unexamined，reason______		
Date	|__|__|__|__|YEAR|__|__|MONTH|__|__|DAY		
Daily maximum temperature	|__|__|.|__|℃	Measurement method	□1 Axilla □2Oral □3 Anus □4 Ear □5 Unknown □6 Other，please describe________
Respiratory	|__|__|times/min	Pulse	|__|__|__|__|times/min
Blood pressure	|__|__|__|__|/|__|__|__|__| mmHg（Systolic/diastolic blood pressure）		

**Physical examination**	□0 Unexamined, **reason**:______				
Date	|__|__|__|__|YEAR|__|__|MONTH|__|__|DAY				
Inspection Part	Result<br>1= Normal<br>2= Abnormal, No Clinical significance<br>3= Abnormal, with Clinically significant<br>0=Unexamined				If abnormal，please describe
General appearance	□1	□2	□3	□0	
Head	□1	□2	□3	□0	
Eyes	□1	□2	□3	□0	
Nose	□1	□2	□3	□0	
Larynx	□1	□2	□3	□0	
Neck and thyroid	□1	□2	□3	□0	
Skin	□1	□2	□3	□0	
Cardiovascular system	□1	□2	□3	□0	

Chest and lungs	□1	□2	□3	□0	
Abdomen	□1	□2	□3	□0	
Nervous system	□1	□2	□3	□0	
Musculoskeletal system	□1	□2	□3	□0	
Lymphatic system	□1	□2	□3	□0	

Respiratory symptoms □0Unexamined, reason____________________

Date: |__|__|__|__|YEAR|__|__|MONTH|__|__|DAY

Whether the subject has respiratory symptoms or not: □0 NO □1 YES, please describe: ____

____

Result: □1 Normal □2 Abnormal,No Clinical significance □3 Abnormal,Clinically significant

Blood Count □0Unexamined, reason____________________

Date: |__|__|__|__|YEAR|__|__|MONTH|__|__|DAY Normal range Number: |__|__|__|__|__|

Item	Result		Result Interpretation			Unexamined
				Abnormal		
	Measured Value	Unit	Normal	No Clinical significance	Clinically significant	
Red Blood Cell（RBC）		□1 10^{12}/L □2Other: _____	□1	□2	□3	□0
White Blood Cell		□1 10^{9}/L □2Other: _____	□1	□2	□3	□0
Hemoglobin（Hb）		□1 g/L □2Other: _____	□1	□2	□3	□0
Platelet（PLT）		□1 10^{9}/L □2Other: _____	□1	□2	□3	□0
Neutrophil（ANC）		□1 10^{9}/L □2Other: _____	□1	□2	□3	□0
Lymphocyte（LY）		□1 10^{9}/L □2Other: _____	□1	□2	□3	□0
Monocyte（MO）		□1 10^{9}/L □2Other: _____	□1	□2	□3	□0
Eosinophil（EO）		□1 10^{9}/L □2Other: _____	□1	□2	□3	□0
Basophil（BA）		□1 10^{9}/L □2Other: _____	□1	□2	□3	□0

Blood Biochemistry □0Unexamined, reason____________________

Date|__|__|__|__|YEAR|__|__|MONTH|__|__|DAY Normal range Number: |__|__|__|__|__|

Item	Result	Result Interpretation	Unexa

	Measured Value	Unit	Normal	Abnormal		mined
				No Clinical significance	Clinically significant	
Glutamic-pyruvic transaminase (ALT)		□1 U/L □2Other: _____	□1	□2	□3	□0
Glutamic-oxalacetic transaminease (AST)		□1 U/L □2Other: _____	□1	□2	□3	□0
Total Bilirubin (TBIL)		□1 μmol/L □2Other: _____	□1	□2	□3	□0
Albumin (ALB)		□1 g/L □2Other: _____	□1	□2	□3	□0
γ- glutamyl transpeptidas (GGT)		□1 U/L □2Other: _____	□1	□2	□3	□0
Lactic dehydrogenase (LDH)		□1 U/L □2Other: _____	□1	□2	□3	□0
Creatine kinase (CK)		□1 U/L □2Other: _____	□1	□2	□3	□0
Creatine kinase isoenzyme (CK-MB)		□1 U/L □2Other: _____	□1	□2	□3	□0
Blood urea nitrogen (BUN)		□1 mmol/L □2Other: _____	□1	□2	□3	□0
Urea (Urea)		□1 mmol/L □2Other: _____	□1	□2	□3	□0
Serum creatinine (CR)		□1 μmol/L □2Other: _____	□1	□2	□3	□0

Urine Test □0Unexamined, reason________________

Date: |__|__|__|__|YEAR|__|__|MONTH|__|__|DAY Normal range Number: |__|__|__|__|

Item	Result		Result Interpretation			Unexamined
	Measured Value	Unit	Normal	Abnormal		
				No Clinical significance	Clinically significant	
Urine Protein		□1Not Applicable □2Other: _____	□1	□2	□3	□0
Urine Glucose		□1 mmol/L □2Other: _____	□1	□2	□3	□0
Urine PH value		□1 Not Applicable □2Other: _____	□1	□2	□3	□0
Urine Erythrocyte		□1Not Applicable □2Other: _____	□1	□2	□3	□0
Urine Leukocyte		□1Not Applicable □2Other: _____	□1	□2	□3	□0
Urine Ketone		□1Not Applicable □2Other: _____	□1	□2	□3	□0

Coagulation Function □0Unexamined, reason________________

Date: \|__\|__\|__\|__\|YEAR\|__\|__\|MONTH\|__\|__\|DAY Normal range Number: \|__\|__\|__\|__\|

Item	Result		Result Interpretation			Unexamined
				Abnormal		
	Measured Value	Unit	Normal	No Clinical significance	Clinically significant	
ProthrombinTime（PT）		□1 S □2Other: _____	□1	□2	□3	□0
Activated Partial Thromboplastin Time（APTT）		□1 S □2Other: _____	□1	□2	□3	□0
Thrombin Time（TT）		□1 S □2Other: _____	□1	□2	□3	□0
Fibrinogen（Fbg）		□1 g/L □2Other: _____	□1	□2	□3	□0
International Normalized Ratio（INR）		□1Not Applicable □2Other: _____	□1	□2	□3	□0

C-REACTIVE PROTEIN（CRP） □0Unexamined, reason________________

Date: \|__\|__\|__\|__\|YEAR\|__\|__\|MONTH\|__\|__\|DAY Normal range Number: \|__\|__\|__\|__\|

Item	Result		Result Interpretation		
				Abnormal	
C-REACTIVE PROTEIN（CRP）	Measured Value	Unit	Normal	No Clinical significance	Clinically significant
		□1 mg/L □2Other: _____	□1	□2	□3

Erythrocyte Sedimentation Rate（ESR） □0Unexamined, reason________________

Date: \|__\|__\|__\|__\|YEAR\|__\|__\|MONTH\|__\|__\|DAY Normal range Number: \|__\|__\|__\|__\|

Item	Result		Result Interpretation		
				Abnormal	
Erythrocyte Sedimentation Rate（ESR）	Measured Value	Unit	Normal	No Clinical significance	Clinically significant
		□1 mm/h □2Other: _____	□1	□2	□3

TNF-α □0Unexamined, reason________________

Date: \|__\|__\|__\|__\|YEAR\|__\|__\|MONTH\|__\|__\|DAY Normal range Number: \|__\|__\|__\|__\|

Item	Result		Result Interpretation		
				Abnormal	
TNF-α	Measured Value	Unit	Normal	No Clinical significance	Clinically significant
		□1 pg/mL □2Other: _____	□1	□2	□3

IL-6 □0Unexamined, reason________________

Date：\|__\|__\|__\|__\|YEAR\|__\|__\|MONTH\|__\|__\|DAY　　Normal range Number：\|__\|__\|__\|__\|__\|

Item	Result			Result Interpretation		
	Measured Value	Unit		Normal	Abnormal	
					No Clinical significance	Clinically significant
IL-6		□1 pg/mL	□2Other：_____	□1	□2	□3

Urine or serum HCG (Only applicable to women of childbearing age) □0Unexamined，reason____

□1Not Applicable（Man or woman who isn't able to reproduce）

Date：\|__\|__\|__\|__\|YEAR\|__\|__\|MONTH\|__\|__\|DAY

Sample：□1 Blood　　□2 Urine

Result：□1 Negative　　□2 Positive

Routine 12 Leads Electrocardiogram　□0Unexamined，reason______________________

Date：\|__\|__\|__\|__\|YEAR\|__\|__\|MONTH\|__\|__\|DAY

Result：□1 Normal　□2 Abnormal,No Clinical significance　□3 AbnormalClinically significant

If"Abnormal"，please describe：____

____

Chest CT　□0Unexamined，reason____

Date：\|__\|__\|__\|__\|YEAR\|__\|__\|MONTH\|__\|__\|DAY

Result：□1　Normal　□2　Abnormal,No Clinical significance　□3　Abnormal，Clinically significant

If abnormal, please describe ____________________________

Wheather chest CT lesion absorbed≥50% than baseline??

□0　NO　□1　YES　□2 Unidentified　□3 Unknown

□4 Other，please describe：______________________________

Nucleic Acid Test of 2019-nCov　□0 Unexamined，reason_______________________

Sampling Part（mutiple choice）：□1Upper respiratory tract　□2Lower respiratory tract

Upper respiratory tract	Date：|__|__|__|__|YEAR|__|__|MONTH|__|__|DAY<br>Result：□1 Negative □2 Positive □3 Unidentified □4 Other，please describe ___________

Lower respiratory tract	Date: |__|__|__|__|YEAR|__|__|MONTH|__|__|DAY<br>Result: □1 Negative □2 Positive □3 Unidentified □4 Other, please describe ___________
Result	□1 Negative □2 Positive □3 Unidentified □4 Other, please describe ___________

**Pulse oxygen saturation（without oxygen inhalation ）** □0Unexamined, reason______
Date: |__|__|__|__|YEAR|__|__|MONTH|__|__|DAY
Measured Value: _____________% （Rest state）

**Arterial blood gas analysis** □0Unexamined, reason______________________________ □1 Not applicable
Oxygen inhalation: □1No □2Yes, FiO2:_____________%
Date: |__|__|__|__|YEAR|__|__|MONTH|__|__|DAY<br>Normal range Number: |__|__|__|__|__|

Item	Result		Result Interpretation			Unexamined
	Measured Value	Unit	Normal	Abnormal		
				No Clinical significance	Clinical significance	
PaO2		□1 mmHg □2 Other: _____	□1	□2	□3	□0
SaO2		□1 % □2 Other:	□1	□2	□3	□0

**Clinical state** □0Unexamined, reason____	
Date	|__|__|__|__|YEAR|__|__|MONTH|__|__|DAY
Result	□1 Level 1: Discharge<br>□2 Level 2: Hospitalization, no requirment for oxygen therapy<br>□3 Level 3: Hospitalization, requiring oxygen therapy (non-NIV / HFNC)<br>□4 Level 4: ICU / Hospitalization, requiring non-invasive mechanical ventilation (NIV) / High Flow Nasal Catheter Oxygen Therapy (HFNC)<br>□5 Level 5: ICU, requiring extracorporeal membrane oxygenation (ECMO) and / or invasive mechanical ventilation (IMV)<br>□6 Level 6: Death

If any adverse events have occurred since the last visit, please fill in the “adverse events” form. If there is medication combination, please fill in the “previous/combined medication” form; If there is combined non-drug therapy, please fill in the “combined non-drug therapy” form; If there is an out-of-plan inspection, please fill in the “out-of-plan inspection” form; If there is IMV/NIV、ECMO、HFNC、oxygen therapy, please fill in the “IMV/NIV、ECMO、HFNC、oxygen therapy” form.

End of the Treatment
Whether the subject finished all the treatment according to the protocol？ □0 NO □1 YES
End Date： |__|__|__|__|YEAR|__|__|MONTH|__|__|DAY
If the patient exit ahead of schedule，please choose the most important reason（One-Choice）:<br>□1 Disease progression occurred during the study and changes in treatment were required after comprehensive assessment by the investigators.<br>□2 Adverse events lead to an exacerbation of the disease<br>□3 The subject is reluctant to continue using the study drug.<br>□4 Pregnancy<br>□5 Loss to follow-up<br>□6 Death<br>□7 Subject withdraws the Informed Consent<br>□8 Study suspension<br>□9 Other，please describe：______________________________<br>______________________________<br><br>Notes（Please record other matter which needs additional clarification）:<br>______________________________<br>______________________________<br>______________________________

**Type of visit**	□1 Telephone follow-up □2 Hospital follow-up
**Date of Current Visit:** |__|__|__|__|YEAR|__|__|MONTH|__|__|DAY	

If the subject has been discharged from the hospital, the following contents may not be carried out, please leave blank; If the subject is not discharged, please conduct the following examinations.

**Vital Signs** □0Unexamined, reason________			
Date	|__|__|__|__|YEAR|__|__|MONTH|__|__|DAY		
Daily maximum temperature	|__|__|.|__|℃	Measurement method	□1 Axilla □2Oral □3 Anus □4 Ear □5 Unknown □6 Other, please describe________
Respiratory	|__|__|times/min	Pulse	|__|__|__|times/min
Blood pressure	|__|__|__|/|__|__|__| mmHg (Systolic/diastolic blood pressure)		

**Physical examination** □0 Unexamined, reason:________					
Date	|__|__|__|__|YEAR|__|__|MONTH|__|__|DAY				
Inspection Part	Result<br>1= Normal<br>2= Abnormal, No Clinical significance<br>3= Abnormal, with Clinically significant<br>0=Unexamined				If abnormal, please describe
General appearance	□1	□2	□3	□0	
Head	□1	□2	□3	□0	
Eyes	□1	□2	□3	□0	
Nose	□1	□2	□3	□0	
Larynx	□1	□2	□3	□0	
Neck and thyroid	□1	□2	□3	□0	
Skin	□1	□2	□3	□0	

Cardiovascular system	□1	□2	□3	□0	
Chest and lungs	□1	□2	□3	□0	
Abdomen	□1	□2	□3	□0	
Nervous system	□1	□2	□3	□0	
Musculoskeletal system	□1	□2	□3	□0	
Lymphatic system	□1	□2	□3	□0	

**Respiratory symptoms** □0Unexamined, reason________________________
Date: |__|__|__|__|YEAR|__|__|MONTH|__|__|DAY
Whether the subject has respiratory symptoms or not: □0 NO □1 YES, please describe: ____ ________________
Result: □1 Normal □2 Abnormal,No Clinical significance □3 Abnormal,Clinically significant

**Blood Count** □0Unexamined, reason________________________
Date: |__|__|__|__|YEAR|__|__|MONTH|__|__|DAY Normal range Number: |__|__|__|__|

Item	Result		Result Interpretation			Unexamined
	Measured Value	Unit	Normal	Abnormal: No Clinical significance	Abnormal: Clinically significant	
Red Blood Cell（RBC）		□1 10^{12}/L □2Other: _____	□1	□2	□3	□0
White Blood Cell		□1 10^9/L □2Other: _____	□1	□2	□3	□0
Hemoglobin（Hb）		□1 g/L □2Other: _____	□1	□2	□3	□0
Platelet（PLT）		□1 10^9/L □2Other: _____	□1	□2	□3	□0
Neutrophil（ANC）		□1 10^9/L □2Other: _____	□1	□2	□3	□0
Lymphocyte（LY）		□1 10^9/L □2Other: _____	□1	□2	□3	□0
Monocyte（MO）		□1 10^9/L □2Other: _____	□1	□2	□3	□0
Eosinophil（EO）		□1 10^9/L □2Other: _____	□1	□2	□3	□0
Basophil（BA）		□1 10^9/L □2Other: _____	□1	□2	□3	□0

**Blood Biochemistry** □0Unexamined, reason________________________
Date|__|__|__|__|YEAR|__|__|MONTH|__|__|DAY Normal range Number: |__|__|__|__|

Item	Result		Result Interpretation			Unexamined
	Measured Value	Unit	Normal	Abnormal: No Clinical significance	Abnormal: Clinically significant	
Glutamic-pyruvic transaminase（ALT）		□1 U/L □2Other：_____	□1	□2	□3	□0
Glutamic-oxalacetic transaminease（AST）		□1 U/L □2Other：_____	□1	□2	□3	□0
Total Bilirubin（TBIL）		□1 μmol/L □2Other：_____	□1	□2	□3	□0
Albumin（ALB）		□1 g/L □2Other：_____	□1	□2	□3	□0
γ- glutamyl transpeptidas（GGT）		□1 U/L □2Other：_____	□1	□2	□3	□0
Lactic dehydrogenase（LDH）		□1 U/L □2Other：_____	□1	□2	□3	□0
Creatine kinase（CK）		□1 U/L □2Other：_____	□1	□2	□3	□0
Creatine kinase isoenzyme（CK-MB）		□1 U/L □2Other：_____	□1	□2	□3	□0
Blood urea nitrogen（BUN）		□1 mmol/L □2Other：_____	□1	□2	□3	□0
Urea（Urea）		□1 mmol/L □2Other：_____	□1	□2	□3	□0
Serum creatinine（CR）		□1 μmol/L □2Other：_____	□1	□2	□3	□0

Urine Test □0Unexamined，reason________________________________

Date：|__|__|__|__|YEAR|__|__|MONTH|__|__|DAY Normal range Number：|__|__|__|__|

Item	Result		Result Interpretation			Unexamined
	Measured Value	Unit	Normal	Abnormal: No Clinical significance	Abnormal: Clinically significance	
Urine Protein		□1Not Applicable □2Other：_____	□1	□2	□3	□0
Urine Glucose		□1 mmol/L □2Other：_____	□1	□2	□3	□0
Urine PH value		□1 Not Applicable □2Other：_____	□1	□2	□3	□0
Urine Erythrocyte		□1Not Applicable □2Other：_____	□1	□2	□3	□0
Urine Leukocyte		□1Not Applicable □2Other：_____	□1	□2	□3	□0
Urine Ketone		□1Not Applicable □2Other：_____	□1	□2	□3	□0

Coagulation Function □0Unexamined，reason________________________________

Date: |__|__|__|__|__|YEAR|__|__|MONTH|__|__|DAY Normal range Number: |__|__|__|__|__|

Item	Result		Result Interpretation			Unexamined
	Measured Value	Unit	Normal	Abnormal: No Clinical significance	Abnormal: Clinically significant	
ProthrombinTime (PT)		□1 S □2Other: _____	□1	□2	□3	□0
Activated Partial Thromboplastin Time (APTT)		□1 S □2Other: _____	□1	□2	□3	□0
Thrombin Time (TT)		□1 S □2Other: _____	□1	□2	□3	□0
Fibrinogen (Fbg)		□1 g/L □2Other: _____	□1	□2	□3	□0
International Normalized Ratio (INR)		□1Not Applicable □2Other: _____	□1	□2	□3	□0

C-REACTIVE PROTEIN (CRP) □0Unexamined, reason______________________

Date: |__|__|__|__|__|YEAR|__|__|MONTH|__|__|DAY Normal range Number: |__|__|__|__|__|

Item	Result		Result Interpretation		
	Measured Value	Unit	Normal	Abnormal: No Clinical significance	Abnormal: Clinically significant
C-REACTIVE PROTEIN (CRP)		□1 mg/L □2Other: _____	□1	□2	□3

Erythrocyte Sedimentation Rate (ESR) □0Unexamined, reason______________________
____

Date: |__|__|__|__|__|YEAR|__|__|MONTH|__|__|DAY Normal range Number: |__|__|__|__|__|

Item	Result		Result Interpretation		
	Measured Value	Unit	Normal	Abnormal: No Clinical significance	Abnormal: Clinically significant
Erythrocyte Sedimentation Rate (ESR)		□1 mm/h □2Other: _____	□1	□2	□3

TNF-α □0Unexamined, reason______________________

Date: |__|__|__|__|__|YEAR|__|__|MONTH|__|__|DAY Normal range Number: |__|__|__|__|__|

Item	Result			Result Interpretation		
	Measured Value	Unit		Normal	Abnormal: No Clinical significance	Abnormal: Clinically significant
TNF-α		□1 pg/mL	□2Other: _____	□1	□2	□3

IL-6 □0Unexamined, reason______________________

Date： \|__\|__\|__\|__\|YEAR\|__\|__\|MONTH\|__\|__\|DAY Normal range Number： \|__\|__\|__\|__\|

Item	Result			Result Interpretation		
	Measured Value	Unit		Normal	Abnormal	
					No Clinical significance	Clinically significant
IL-6		□1 pg/mL	□2Other: _____	□1	□2	□3

Routine 12 Leads Electrocardiogram □0Unexamined, reason________________________

Date： \|__\|__\|__\|__\|YEAR\|__\|__\|MONTH\|__\|__\|DAY

Result： □1 Normal □2 Abnormal,No Clinical significance □3 AbnormalClinically significant

If"Abnormal", please describe： ____

____

Chest CT □0Unexamined, reason________________________________

Date： \|__\|__\|__\|__\|YEAR\|__\|__\|MONTH\|__\|__\|DAY

Result： ____

____

Wheather chest CT lesion absorbed⩾50% than baseline??

□0 NO □1 YES □2 Unidentified □3 Unknown □4 Other, please describe： ____

____

Nucleic Acid Test of 2019-nCov □0 Unexamined, reason________________________

Sampling Part（mutiple choice）： □1Upper respiratory tract □2Lower respiratory tract

Upper respiratory tract	Date： |__|__|__|__|YEAR|__|__|MONTH|__|__|DAY<br>Result： □1 Negative □2 Positive □3 Unidentified □4 Other, please describe ___________
Lower respiratory tract	Date： |__|__|__|__|YEAR|__|__|MONTH|__|__|DAY<br>Result： □1 Negative □2 Positive □3 Unidentified □4 Other, please describe ___________
Result	□1 Negative □2 Positive □3 Unidentified □4 Other, please describe ___________

Pulse oxygen saturation（without oxygen inhalation ） □0Unexamined, reason_____

Date： \|__\|__\|__\|__\|YEAR\|__\|__\|MONTH\|__\|__\|DAY

Measured Value：______________%（Rest state）

Arterial blood gas analysis □0Unexamined，reason________________________________ □1 Not applicable							
Oxygen inhalation：□1No □2Yes, FiO2:_____________%							
Date：|__|__|__|__|YEAR|__|__|MONTH|__|__|DAY<br>Normal range Number：|__|__|__|__|							
**Item**	**Result**		**Result Interpretation**			**Unexamined**	
	**Measured Value**	**Unit**	**Normal**	**Abnormal**			
				**No Clinical significance**	**Clinical significance**		
PaO2		□1 mmHg □2 Other：_____	□1	□2	□3	□0	
SaO2		□1 % □2 Other：	□1	□2	□3	□0	

**Clinical state** □0Unexamined，reason____	
Date	|__|__|__|__|YEAR|__|__|MONTH|__|__|DAY
Result	□1 Level 1: Discharge<br>□2 Level 2: Hospitalization, no requirment for oxygen therapy<br>□3 Level 3: Hospitalization, requiring oxygen therapy (non-NIV / HFNC)<br>□4 Level 4: ICU / Hospitalization, requiring non-invasive mechanical ventilation (NIV) / High Flow Nasal Catheter Oxygen Therapy (HFNC)<br>□5 Level 5: ICU, requiring extracorporeal membrane oxygenation (ECMO) and / or invasive mechanical ventilation (IMV)<br>□6 Level 6: Death

If any adverse events have occurred since the last visit, please fill in the “adverse events” form. If there is medication combination, please fill in the “previous/combined medication” form; If there is combined non-drug therapy, please fill in the “combined non-drug therapy” form; If there is an out-of-plan inspection, please fill in the “out-of-plan inspection” form; If there is IMV/NIV、ECMO、HFNC、oxygen therapy, please fill in the “IMV/NIV、ECMO、HFNC、oxygen therapy” form.

Does the subject use the drug studied? ☐0 NO ☐1 YES ☐2 Not applicable（Control Group）								
**Situation of Drug Usage**								
**Days of taking the drug**	**Whether use the drug**	**Usage Date**	**Planned daily dosage (mg)**	**Actual daily dosage (mg)**	**Frequency (1)**	**Delivery (2)**	**State the reason in case of dose adjustment or suspension**	**Please explain in case of missed、overdose or other situation**
**Day__**	☐0 NO<br>☐1 YES	|__|__|__|__|YEAR<br>|__|__|MONTH<br>|__|__|DAY	|__|__|__|__|	|__|__|__|__|	|__| ________	|__| ________	☐1 AE，Number__<br>☐2 Other, ____	
**Day__**	☐0 NO<br>☐1 YES	|__|__|__|__|YEAR<br>|__|__|MONTH<br>|__|__|DAY	|__|__|__|__|	|__|__|__|__|	|__| ________	|__| ________	☐1 AE，Number__<br>☐2 Other, ____	
**Day__**	☐0 NO<br>☐1 YES	|__|__|__|__|YEAR<br>|__|__|MONTH<br>|__|__|DAY	|__|__|__|__|	|__|__|__|__|	|__| ________	|__| ________	☐1 AE，Number__<br>☐2 Other, ____	
**Day__**	☐0 NO<br>☐1 YES	|__|__|__|__|YEAR<br>|__|__|MONTH<br>|__|__|DAY	|__|__|__|__|	|__|__|__|__|	|__| ________	|__| ________	☐1 AE，Number__<br>☐2 Other, ____	

**Day__**	☐ 0 NO<br>☐1 YES	|__|__|__|__|YEAR<br>|__|__|MONTH<br>|__|__|DAY	|__|__|__|__|	|__|__|__|__|	|__| ________	|__| ________	☐1 AE，Number__<br>☐2 Other, ____	

(1) Frequency: 1= three times a day 2= twice a day 3= once a day 4= unknown 5= others, please specify in the line
(2) Delivery: 1= not applicable 2= dose adjustment 3= missed 4= overdose 5= suspended administration 6= others, please specify in the line

Does the subject use the drug studied? ☐0 **NO** ☐1 **YES** ☐2 **Not applicable（Control Group）**

Situation of Drug Usage

Days of taking the drug	Whether use the drug	Usage Date	Planned daily dosage (mg)	Actual daily dosage (mg)	Frequency (1)	Delivery (2)	State the reason in case of dose adjustment or suspension	Please explain in case of missed、overdose or other situation
**Day__**	☐ 0 NO<br>☐1 YES	|__|__|__|__|YEAR<br>|__|__|MONTH<br>|__|__|DAY	|__|__|__|__|	|__|__|__|__|	|__| ________	|__| ________	☐1 AE，Number__<br>☐2 Other, ____	
**Day__**	☐ 0 NO<br>☐1 YES	|__|__|__|__|YEAR<br>|__|__|MONTH<br>|__|__|DAY	|__|__|__|__|	|__|__|__|__|	|__| ________	|__| ________	☐1 AE，Number__<br>☐2 Other, ____	
**Day__**	☐ 0 NO<br>☐1 YES	|__|__|__|__|YEAR<br>|__|__|MONTH<br>|__|__|DAY	|__|__|__|__|	|__|__|__|__|	|__| ________	|__| ________	☐1 AE，Number__<br>☐2 Other, ____	

**Day__**	☐ 0 NO<br>☐1 YES	|__|__|__|__|YEAR<br>|__|__|MONTH<br>|__|__|DAY	|__|__|__|__|	|__|__|__|__|	|__| ________	|__| ________	☐1 AE，Number__<br>☐2 Other, ____	
**Day__**	☐ 0 NO<br>☐1 YES	|__|__|__|__|YEAR<br>|__|__|MONTH<br>|__|__|DAY	|__|__|__|__|	|__|__|__|__|	|__| ________	|__| ________	☐1 AE，Number__<br>☐2 Other, ____	

（1）Frequency: 1= three times a day 2= twice a day 3= once a day 4= unknown 5= others, please specify in the line
（2）Delivery: 1= not applicable 2= dose adjustment 3= missed 4= overdose 5= suspended administration 6= others, please specify in the line

Did the subject experience any adverse event? ☐0 **NO** ☐1 **YES**， **please fill in refer to the code at the bottom of the form**

Adverse Events?

NO.	Adverse Event	Date Of begining	Sustained at the end of the study?		CTCAE Level (1)	Taken Measurements		Relationship with the studied drug (4)	AE Outcome (5)	SAE or Not?	
			YES	NO→Date of End		Measurement for the studied drug（2)	Other measurement (3)			No	YES，the specific SAE type（6)
1		|__|__|__|__|YEAR|__|__|MONTH|__|__|DAY	☐	|__|__|__|__|YEAR|__|__|MONTH|__|__|DAY	|__|	|__| ________	|__| ________	|__|	|__|	☐	|__|
2		|__|__|__|__|YEAR|__|__|MONTH|__|__|DAY	☐	|__|__|__|__|YEAR|__|__|MONTH|__|__|DAY	|__|	|__| ________	|__| ________	|__|	|__|	☐	|__|
3		|__|__|__|__|YEAR|__|__|MONTH|__|__|DAY	☐	|__|__|__|__|YEAR|__|__|MONTH|__|__|DAY	|__|	|__| ________	|__| ________	|__|	|__|	☐	|__|
4		|__|__|__|__|YEAR|__|__|MONTH|__|__|DAY	☐	|__|__|__|__|YEAR|__|__|MONTH|__|__|DAY	|__|	|__| ________	|__| ________	|__|	|__|	☐	|__|

(1) CTCAE Level	(2) Measurement for the studied drug	(3) Other measurements	(4) Relationship with the studied drug	(5) AE outcome	(6) Specific SAE type

Protocol Number: KY2020-29	Adverse Events
Center Number |__|__| Subject Screening Number |__|__|__|__|__|__|	

1=level 1<br>2=level 2<br>3=level 3<br>4=level 4<br>5=level 5	1=Maintenance of current treatment<br>2=Suspension<br>3=Termination<br>4=Reduce the dose<br>5=Not applicable<br>6= Others, please specify in thel line	1= No<br>2= Combined medication, fill in the Combined Medication Form<br>3= Combined with non-drug therapy, fill in the Combined non-drug therapy Form<br>4= Others, please specify in thel line	1=Certainly related<br>2=Probably related<br>3=Possibly related<br>4= Possibly non-related<br>5= Certainly non- related<br>6= Remain to be evaluated<br>7=Unable to evaluate	1 = unchanged<br>2=deterioration<br>3= recovery/healing<br>4=improvement/recovery<br>5= recovery with sequelae<br>6 = death<br>7 = unknown	1= life threatening<br>2= cause death<br>3= require hospitalization or extend the current stay<br>4= cause permanent or significant disability or dysfunction<br>5= causes congenital malformation/birth defects<br>6= other important medical events

Did the subject experience any adverse event? ☐0 **NO** ☐1 **YES**, **please fill in refer to the code at the bottom of the form**

Adverse Events?

**NO.**	**Adverse Event**	**Date Of begining**	**Sustained at the end of the study?**		**CTCAE Level (1)**	**Taken Measurements**		**Relationship with the studie**	**AE Outcome (5)**	**SAE or Not?**	
			**YES**	NO→Date of End		**Measurement for the studied drug (2)**	**Other measurement (3)**			**No**	**YES, the specific SAE type (6)**
**1**		|__|__|__|__|YEAR|__|__|MONTH|__|__|DAY	☐	|__|__|__|__|YEAR|__|__|MONTH|__|__|DAY	|__|	|__| ________	|__| ________________	|__|	|__|	☐	|__|
**2**		|__|__|__|__|YEAR|__|__|MONTH|__|__|DAY	☐	|__|__|__|__|YEAR|__|__|MONTH|__|__|DAY	|__|	|__| ________	|__| ________________	|__|	|__|	☐	|__|

**3**		|__|__|__|__|Y EAR|__|__|MO NTH|__|__|DA Y	☐	|__|__|__|__|YEA R|__|__|MONTH| __|__|DAY	|__|	|__| ________	|__| ________________	|_ _|	|__|	☐	|__|
**4**		|__|__|__|__|Y EAR|__|__|MO NTH|__|__|DA Y	☐	|__|__|__|__|YEA R|__|__|MONTH| __|__|DAY	|__|	|__| ________	|__| ________________	|_ _|	|__|	☐	|__|

**(1) CTCAE Level**	**(2) Measurement for the studied drug**	**(3) Other measurements**	**(4) Relationship with the studied drug**	**(5) AE outcome**	**(6) Specific SAE type**
1=level 1<br>2=level 2<br>3=level 3<br>4=level 4<br>5=level 5	1=Maintenance of current treatment<br>2=Suspension<br>3=Termination<br>4=Reduce the dose<br>5=Not applicable<br>6= Others, please specify in thel line	1= No<br>2= Combined medication，fill in the Combined Medication Form<br>3= Combined with non-drug therapy，fill in the Combined non-drug therapy Form<br>4= Others, please specify in thel line	1=Certainly related<br>2=Probably related<br>3=Possibly related<br>4= Possibly non-related<br>5= Certainly non- related<br>6= Remain to be evaluated<br>7=Unable to evaluate	1 = unchanged<br>2=deterioration<br>3= recovery/healing<br>4=improvement/recovery<br>5= recovery with sequelae<br>6 = death<br>7 = unknown	1= life threatening<br>2= cause death<br>3= require hospitalization or extend the current stay<br>4= cause permanent or significant disability or dysfunction<br>5= causes congenital malformation/birth defects<br>6= other important medical events

Does the subject have previous/combined medication?

☐0 NO ☐1 YES，please fill in the form below refer to the code at the bottom of the table.

Previous/Combined medication

NO.	Drug Name (generic name)	Indications (1)	Indications Notes	single dose	Dose Unit (2)	Dosing frequency (3)	administration route (4)	Date of beginning	Sustain at the end of the study?	
									Yes	NO→Date of end
1		|__|			|__|	|__|	|__|	|__|__|__|__|YEAR|__|__|MONTH|__|__|DAY	☐	|__|__|__|__|YEAR|__|__|MONTH|__|__|DAY
2		|__|			|__|	|__|	|__|	|__|__|__|__|YEAR|__|__|MONTH|__|__|DAY	☐	|__|__|__|__|YEAR|__|__|MONTH|__|__|DAY
3		|__|			|__|	|__|	|__|	|__|__|__|__|YEAR|__|__|MONTH|__|__|DAY	☐	|__|__|__|__|YEAR|__|__|MONTH|__|__|DAY
4		|__|			|__|	|__|	|__|	|__|__|__|__|YEAR|__|__|MONTH|__|__|DAY	☐	|__|__|__|__|YEAR|__|__|MONTH|__|__|DAY
5		|__|			|__|	|__|	|__|	|__|__|__|__|YEAR|__|__|MONTH|__|__|DAY	☐	|__|__|__|__|YEAR|__|__|MONTH|__|__|DAY

(1) Indications	(2) dosage Unit	(3) Dosing frequency	(4) administration route
1=Disease studied in this study<br>2= Previous history (please fill in the history number in the Notes)<br>3= Adverse events (please fill in the number of adverse events in Notes)	1 = micrograms (μg)<br>2 = milligram (mg)<br>3 = gram (g)<br>4 = milliliter (mL)<br>5 = International Unit (IU)<br>6=Unit(U)<br>7=Tablet(Tab)<br>8=capsule (Cap)<br>9=Unknown<br>10=Other,please fill in the line	1= Once a day (qd)<br>2= Twice a day (bid)<br>3= Three times a day (tid)<br>4= Four<br>6=Once a week（qw)<br>7=Before sleep(hs)<br>8=As required (prn)<br>9=Unknown<br>10=Other，please fill in the line	1=oral (PO)<br>2= intravenous drop(IVgtt)<br>3=Intravenous injection(IV)<br>4=Intramuscular (IM)<br>5= Subcutaneous<br>7= Local (skin) (TOP)<br>8= ophthalmus(OPH)<br>9=Inhalation(INH)<br>10=Unknown<br>11= Other，please fill in the line

4=Other（please describe in the Notes）		times a day (qid)<br>5= once every other day (qod)	(SQ)<br>6= sublingual (SL)

Does the subject have previous/combined medication?

☐0 **NO** ☐1 **YES，please fill in the form below refer to the code at the bottom of the table.**

Previous/Combined medication

NO.	Drug Name (generic name)	Indications (1)	Indications Notes	single dose	Dose Unit (2)	Dosing frequency (3)	administration route (4)	Date of beginning	Sustain at the end of the study?	
									Yes	NO→Date of end
1		|__|			|__|	|__|	|__|	|__|__|__|__|YEAR|__|__|MONTH|__|__|DAY	☐	|__|__|__|__|YEAR|__|__|MONTH|__|__|DAY
2		|__|			|__|	|__|	|__|	|__|__|__|__|YEAR|__|__|MONTH|__|__|DAY	☐	|__|__|__|__|YEAR|__|__|MONTH|__|__|DAY
3		|__|			|__|	|__|	|__|	|__|__|__|__|YEAR|__|__|MONTH|__|__|DAY	☐	|__|__|__|__|YEAR|__|__|MONTH|__|__|DAY
4		|__|			|__|	|__|	|__|	|__|__|__|__|YEAR|__|__|MONTH|__|__|DAY	☐	|__|__|__|__|YEAR|__|__|MONTH|__|__|DAY
5		|__|			|__|	|__|	|__|	|__|__|__|__|YEAR|__|__|MONTH|__|__|DAY	☐	|__|__|__|__|YEAR|__|__|MONTH|__|__|DAY

(1) Indications	(2) dosage Unit	(3) Dosing frequency	(4) administration route

1=Disease studied in this study<br>2= Previous history (please fill in the history number in the Notes)<br>3= Adverse events (please fill in the number of adverse events in Notes)<br>4=Other（please describe in the Notes）	1 = micrograms (μg)<br>2 = milligram (mg)<br>3 = gram (g)<br>4 = milliliter (mL)<br>5 = International Unit (IU)	6=Unit(U)<br>7=Tablet(Tab)<br>8=capsule (Cap)<br>9=Unknown<br>10=Other,please fill in the line	1= Once a day (qd)<br>2= Twice a day (bid)<br>3= Three times a day (tid)<br>4= Four times a day (qid)<br>5= once every other day （qod)	6=Once a week（qw)<br>7=Before sleep(hs)<br>8=As required (prn)<br>9=Unknown<br>10=Other，please fill in the line	1=oral (PO)<br>2= intravenous drop(IVgtt)<br>3=Intravenous injection(IV)<br>4=Intramuscular (IM)<br>5= Subcutaneous (SQ)<br>6= sublingual (SL)	7= Local (skin) (TOP)<br>8= ophthalmus(OPH)<br>9=Inhalation(INH)<br>10=Unknown<br>11= Other，please fill in the line

Does the subject have previous/combined non-drug treatment? ☐0 **NO** ☐1 **YES，Please continue to fill in the form below.**

Previous/Combined non-drug treatment							
**NO.**	**Name of Treatment** (please describe in professional medical terms)	**Indications (1)**	**Notes for indications**	**Date of beginning**	**Sustain at the end of the study?**		**Whether it is the standard treatment recommended in the guidelines? (2)**
					**YES**	**NO→Date of end**	
**1**		|__|		|__|__|__|__|YEAR|__|__|MONTH|__|__|DAY	☐	|__|__|__|__|YEAR|__|__|MONTH|__|__|DAY	
**2**		|__|		|__|__|__|__|YEAR|__|__|MONTH|__|__|DAY	☐	|__|__|__|__|YEAR|__|__|MONTH|__|__|DAY	
**3**		|__|		|__|__|__|__|YEAR|__|__|MONTH|__|__|DAY	☐	|__|__|__|__|YEAR|__|__|MONTH|__|__|DAY	
**4**		|__|		|__|__|__|__|YEAR|__|__|MONTH|__|__|DAY	☐	|__|__|__|__|YEAR|__|__|MONTH|	

						__|__|DAY	
**5**		|__|		|__|__|__|__|YEAR|__|__|MONTH|__|__|DAY	□	|__|__|__|__|YEAR|__|__|MONTH|__|__|DAY	
**6**		|__|		|__|__|__|__|YEAR|__|__|MONTH|__|__|DAY	□	|__|__|__|__|YEAR|__|__|MONTH|__|__|DAY	

(1) Indications：1= Disease studied in this study 2= Previous history (please fill in the history number in the Notes) 3= Adverse events (please fill in the number of adverse events in Notes) 4=Other（please describe in the Notes）

(2) Whether it is the standard treatment recommended in the guidelines? 0=NO 1=YES 2=Not applicable

Does the subject have previous/combined non-drug treatment? □0 **NO** □1 **YES，Please continue to fill in the form below.**

**Previous/Combined non-drug treatment**							
**NO.**	**Name of Treatment** (please describe in professional medical terms)	**Indications (1)**	**Notes for indications**	**Date of beginning**	**Sustain at the end of the study?**		**Whether it is the standard treatment recommended in the guidelines? (2)**
					**YES**	**NO→Date of end**	

**1**		|__|		|__|__|__|__|YEAR|__|__|MONTH|__|__|DAY	□	|__|__|__|__|YEAR|__|__|MONTH|__|__|DAY	
**2**		|__|		|__|__|__|__|YEAR|__|__|MONTH|__|__|DAY	□	|__|__|__|__|YEAR|__|__|MONTH|__|__|DAY	
**3**		|__|		|__|__|__|__|YEAR|__|__|MONTH|__|__|DAY	□	|__|__|__|__|YEAR|__|__|MONTH|__|__|DAY	
**4**		|__|		|__|__|__|__|YEAR|__|__|MONTH|__|__|DAY	□	|__|__|__|__|YEAR|__|__|MONTH|__|__|DAY	
**5**		|__|		|__|__|__|__|YEAR|__|__|MONTH|__|__|DAY	□	|__|__|__|__|YEAR|__|__|MONTH|__|__|DAY	
**6**		|__|		|__|__|__|__|YEAR|__|__|MONTH|__|__|DAY	□	|__|__|__|__|YEAR|__|__|MONTH|__|__|DAY	

（1）**Indications**：1**= Disease studied in this study 2= Previous history (please fill in the history number in the Notes) 3= Adverse events (please fill in the number of adverse events in Notes) 4=Other（please describe in the Notes）**
（2）**Whether it is the standard treatment recommended in the guidelines? 0=NO 1=YES 2=Not applicable**

IMV/NIV、ECMO、HFNC、0xygen Therapy					
No.	Name of Therapy	Date of the beginning	Time of the beginning	Date of the end	Time of the end
	□1 IMV □3 ECMO □2 NIV □4 HFNC □5 Oxygen Therapy	|__|__|__|__|YEAR|__|__|MONTH|__|__|DAY	|__|__|：|__|__|	|__|__|__|__|YEAR|__|__ |MONTH|__|__|DAY	|__|__|：|__|__|
	□1 IMV □3 ECMO □2 NIV □4 HFNC □5 Oxygen Therapy	|__|__|__|__|YEAR|__|__|MONTH|__|__|DAY	|__|__|：|__|__|	|__|__|__|__|YEAR|__|__ |MONTH|__|__|DAY	|__|__|：|__|__|
	□1 IMV □3 ECMO □2 NIV □4 HFNC □5 Oxygen Therapy	|__|__|__|__|YEAR|__|__|MONTH|__|__|DAY	|__|__|：|__|__|	|__|__|__|__|YEAR|__|__ |MONTH|__|__|DAY	|__|__|：|__|__|
	□1 IMV □3 ECMO □2 NIV □4 HFNC □5 Oxygen Therapy	|__|__|__|__|YEAR|__|__|MONTH|__|__|DAY	|__|__|：|__|__|	|__|__|__|__|YEAR|__|__ |MONTH|__|__|DAY	|__|__|：|__|__|
	□1 IMV □3 ECMO □2 NIV □4 HFNC □5 Oxygen Therapy	|__|__|__|__|YEAR|__|__|MONTH|__|__|DAY	|__|__|：|__|__|	|__|__|__|__|YEAR|__|__ |MONTH|__|__|DAY	|__|__|：|__|__|

IMV/NIV、ECMO、HFNC、0xygen Therapy					
No.	Name of Therapy	Date of the beginning	Time of the beginning	Date of the end	Time of the end
	□1 IMV □3 ECMO □2 NIV □4 HFNC □5 Oxygen Therapy	|__|__|__|__|YEAR|__|__|MONTH|__|__|DAY	|__|__|：|__|__|	|__|__|__|__|YEAR|__|__ |MONTH|__|__|DAY	|__|__|：|__|__|
	□1 IMV □3 ECMO □2 NIV □4 HFNC □5 Oxygen Therapy	|__|__|__|__|YEAR|__|__|MONTH|__|__|DAY	|__|__|：|__|__|	|__|__|__|__|YEAR|__|__ |MONTH|__|__|DAY	|__|__|：|__|__|
	□1 IMV □3 ECMO □2 NIV □4 HFNC □5 Oxygen Therapy	|__|__|__|__|YEAR|__|__|MONTH|__|__|DAY	|__|__|：|__|__|	|__|__|__|__|YEAR|__|__ |MONTH|__|__|DAY	|__|__|：|__|__|
	□1 IMV □3 ECMO □2 NIV □4 HFNC □5 Oxygen Therapy	|__|__|__|__|YEAR|__|__|MONTH|__|__|DAY	|__|__|：|__|__|	|__|__|__|__|YEAR|__|__ |MONTH|__|__|DAY	|__|__|：|__|__|
	□1 IMV □3 ECMO □2 NIV □4 HFNC □5 Oxygen Therapy	|__|__|__|__|YEAR|__|__|MONTH|__|__|DAY	|__|__|：|__|__|	|__|__|__|__|YEAR|__|__ |MONTH|__|__|DAY	|__|__|：|__|__|

Is there any other examination to record? ☐0 **NO** ☐1 **YES, please fill in the table below.**

**Other Examinations**							
**Item**	**Date of sample or examine**	**Result**	**Unit**	**Result Interpretation**<br>**1=Normal**<br>**2=Abnormal, No Clinical significance**<br>**3=Abnormal, with Clinically significant**			**Notes**
	|__|__|__|__|YEAR|__|__|MONTH|__|__|DAY			☐1	☐2	☐3	
	|__|__|__|__|YEAR|__|__|MONTH|__|__|DAY			☐1	☐2	☐3	
	|__|__|__|__|YEAR|__|__|MONTH|__|__|DAY			☐1	☐2	☐3	
	|__|__|__|__|YEAR|__|__|MONTH|__|__|DAY			☐1	☐2	☐3	
	|__|__|__|__|YEAR|__|__|MONTH|__|__|DAY			☐1	☐2	☐3	
	|__|__|__|__|YEAR|__|__|MONTH|__|__|DAY			☐1	☐2	☐3	

Study Summary
Whether the subject finished the study according to the protocol？<br>□1 YES，Finish Date：|__|__|__|__|YEAR|__|__|MONTH|__|__|DAY<br>□0 NO，End Date：|__|__|__|__|YEAR|__|__|MONTH|__|__|DAY
If the patient exit ahead of schedule，please choose the most important reason（One-Choice）:<br>□1 Disease progression occurred during the study and changes in treatment were required after comprehensive assessment by the investigators.<br>□2 Adverse events lead to an exacerbation of the disease<br>□3 The subject is reluctant to continue using the study drug.<br>□4 Pregnancy<br>□5 Loss to follow-up<br>□6 Death<br>□7 Subject withdraws the Informed Consent<br>□8 Study suspension<br>□9 Other，please describe：________________________________<br>________________________________<br><br>Notes（Please record other matter which needs additional clarification）:<br>________________________________<br>________________________________<br>________________________________

Information on hospitalization
Was the subject hospitalized with 2019-nCov respiratory infection？□0 NO □1 YES
If Yes，date of admission：|__|__|__|__|YEAR|__|__|MONTH|__|__|DAY
Be discharged from hospital at the end of the study or not？ □0 NO □1 YES
If yes，discharge date ：|__|__|__|__|YEAR|__|__|MONTH|__|__|DAY

Death			
Are the subjects dead?	□0 NO	□1 YES，please fill in	□2 Unkown
Date of dath：|__|__|__|__|YEAR|__|__|MONTH|__|__|DAY			
The cause of death ：<br>□1 Adverse events<br>□2 Unkown<br>□3 Other，please describe____<br>____			

Investigator Statement

I have reviewed and confirmed that all data recorded in this case report form are true, accurate, reliable, complete, and consistent with the original data. All errors or omissions have been corrected or noted.

Signature of principal investigator： ___________________

Date：|__|__|__|__|YEAR|__|__|MONTH|__|__|DAY

CRA Statement

I have checked and confirmed that all the data recorded in this case report form are true, accurate, reliable, complete and correct and consistent with the original data.

CRA Signature： _______________________

Date：|__|__|__|__|YEAR|__|__|MONTH|__|__|DAY

**Vital Signs**	□0Unexamined，reason________________________		
Date	|__|__|__|__|YEAR|__|__|MONTH|__|__|DAY		
Daily maximum temperature	|__|__|.|__|℃	Measurement method	□1 Axilla □2Oral □3 Anus □4 Ear □5 Unknown □6 Other，please describe________
Respiratory	|__|__|times/min	Pulse	|__|__|__|times/min
Blood pressure	|__|__|__|/|__|__|__| mmHg（Systolic/diastolic blood pressure）		

**Vital Signs**	□0Unexamined，reason________________________		
Date	|__|__|__|__|YEAR|__|__|MONTH|__|__|DAY		
Daily maximum temperature	|__|__|.|__|℃	Measurement method	□1 Axilla □2Oral □3 Anus □4 Ear □5 Unknown □6 Other，please describe_______
Respiratory	|__|__|times/min	Pulse	|__|__|__|times/min
Blood pressure	|__|__|__|/|__|__|__| mmHg（Systolic/diastolic blood pressure）		

**Vital Signs**	□0Unexamined，reason________________________		
Date	|__|__|__|__|YEAR|__|__|MONTH|__|__|DAY		
Daily maximum temperature	|__|__|.|__|℃	Measurement method	□1 Axilla □2Oral □3 Anus □4 Ear □5 Unknown □6 Other，please describe_______
Respiratory	|__|__|times/min	Pulse	|__|__|__|times/min
Blood pressure	|__|__|__|/|__|__|__| mmHg（Systolic/diastolic blood pressure）		

**Vital Signs**	□0Unexamined，reason________________________		
Date	|__|__|__|__|YEAR|__|__|MONTH|__|__|DAY		
Daily maximum temperature	|__|__|.|__|℃	Measurement method	□1 Axilla □2Oral □3 Anus □4 Ear □5 Unknown □6 Other，please describe_______
Respiratory	|__|__|times/min	Pulse	|__|__|__|times/min
Blood pressure	|__|__|__|/|__|__|__| mmHg（Systolic/diastolic blood pressure）		

**physical examination** □0 Unexamined, **reason**:________________					
Date	|__|__|__|__|YEAR|__|__|MONTH|__|__|DAY				
Inspection Part	Result<br>1= Normal<br>2= Abnormal, No Clinical significance<br>3= Abnormal, with Clinically significant<br>0=Unexamined				If abnormal，please describe
General appearance	□1	□2	□3	□0	
Head	□1	□2	□3	□0	
Eyes	□1	□2	□3	□0	
Nose	□1	□2	□3	□0	
Larynx	□1	□2	□3	□0	
Neck and thyroid	□1	□2	□3	□0	
Skin	□1	□2	□3	□0	
Cardiovascular system	□1	□2	□3	□0	
Chest and lungs	□1	□2	□3	□0	
Abdomen	□1	□2	□3	□0	
Nervous system	□1	□2	□3	□0	
Musculoskeletal system	□1	□2	□3	□0	
Lymphatic system	□1	□2	□3	□0	

Respiratory symptoms　□0Unexamined, reason________________

Date: |__|__|__|__|YEAR|__|__|MONTH|__|__|DAY

Whether the subject has respiratory symptoms or not: □0 NO　□1 NO, please describe: ______

Result: □1 Normal　□2 Abnormal,No Clinical significance　□3 AbnormalSignificant clinically

Respiratory symptoms　□0Unexamined, reason________________

Date: |__|__|__|__|YEAR|__|__|MONTH|__|__|DAY

Whether the subject has respiratory symptoms or not: □0 NO　□1 NO, please describe: ______

Result: □1 Normal　□2 Abnormal,No Clinical significance□3 AbnormalSignificant clinically

Respiratory symptoms　□0Unexamined, reason________________

Date: |__|__|__|__|YEAR|__|__|MONTH|__|__|DAY

Whether the subject has respiratory symptoms or not: □0 NO　□1 NO, please describe: ______

Result: □1 Normal　□2 Abnormal,No Clinical significance　□3 AbnormalSignificant clinically

Respiratory symptoms　□0Unexamined, reason________________

Date: |__|__|__|__|YEAR|__|__|MONTH|__|__|DAY

Whether the subject has respiratory symptoms or not: □0 NO　□1 NO, please describe: ______

Result: □1 Normal　□2 Abnormal,No Clinical significance　□3 AbnormalSignificant clinically

Respiratory symptoms　□0Unexamined, reason________________

Date: |__|__|__|__|YEAR|__|__|MONTH|__|__|DAY

Whether the subject has respiratory symptoms or not: □0 NO　□1 NO, please describe: ______

Result: □1 Normal　□2 Abnormal,No Clinical significance　□3 AbnormalSignificant clinically

Respiratory symptoms　□0Unexamined, reason________________

Date: |__|__|__|__|YEAR|__|__|MONTH|__|__|DAY

Whether the subject has respiratory symptoms or not: □0 NO　□1 NO, please describe: ______

Result: □1 Normal　□2 Abnormal,No Clinical significance　□3 AbnormalSignificant clinically

Blood Count □0Unexamined，reason________________

Date：\|__\|__\|__\|__\|YEAR\|__\|__\|MONTH\|__\|__\|DAY Normal range Number：\|__\|__\|__\|__\|

Item	Result		Result Interpretation			Unexamined
	Measured Value	Unit	Normal	Abnormal: No Clinical significance	Abnormal: Significant clinically	
Red Blood Cell（RBC）		□1 10^{12}/L □2Other：_____	□1	□2	□3	□0
White Blood Cell		□1 10^9/L □2Other：_____	□1	□2	□3	□0
Hemoglobin（Hb）		□1 g/L □2Other：_____	□1	□2	□3	□0
Platelet（PLT）		□1 10^9/L □2Other：_____	□1	□2	□3	□0
Neutrophil（ANC）		□1 10^9/L □2Other：_____	□1	□2	□3	□0
Lymphocyte（LY）		□1 10^9/L □2Other：_____	□1	□2	□3	□0
Monocyte（MO）		□1 10^9/L □2Other：_____	□1	□2	□3	□0
Eosinophil（EO）		□1 10^9/L □2Other：_____	□1	□2	□3	□0
Basophil（BA）		□1 10^9/L □2Other：_____	□1	□2	□3	□0

Blood Count □0Unexamined，reason________________

Date：\|__\|__\|__\|__\|YEAR\|__\|__\|MONTH\|__\|__\|DAY Normal range Number：\|__\|__\|__\|__\|

Item	Result		Result Interpretation			Unexamined
	Measured Value	Unit	Normal	Abnormal: No Clinical significance	Abnormal: Significant clinically	
Red Blood Cell（RBC）		□1 10^{12}/L □2Other：_____	□1	□2	□3	□0
White Blood Cell		□1 10^9/L □2Other：_____	□1	□2	□3	□0
Hemoglobin（Hb）		□1 g/L □2Other：_____	□1	□2	□3	□0
Platelet（PLT）		□1 10^9/L □2Other：_____	□1	□2	□3	□0
Neutrophil（ANC）		□1 10^9/L □2Other：_____	□1	□2	□3	□0
Lymphocyte（LY）		□1 10^9/L □2Other：_____	□1	□2	□3	□0
Monocyte（MO）		□1 10^9/L □2Other：_____	□1	□2	□3	□0
Eosinophil（EO）		□1 10^9/L □2Other：_____	□1	□2	□3	□0
Basophil（BA）		□1 10^9/L □2Other：_____	□1	□2	□3	□0

Protocol Number：KY2020-29	Attachement Out of Plan
Center Number |__|__|　　　Subject Screening Number |__|__|__|__|__|	

**Blood Biochemistry**	□0Unexamined，reason_______						
Date|__|__|__|__|YEAR|__|__|MONTH|__|__|DAY			Normal range Number：|__|__|__|__|				
**Item**	**Result**		**Result Interpretation**				**Unexamined**
	**Measured Value**	**Unit**	**Normal**	**Abnormal**			
				**No Clinical significance**	**Significant clinically**		
Glutamic-pyruvic transaminase（ALT）		□1 U/L　□2Other：_____	□1	□2	□3		□0
Glutamic-oxalacetic transaminease（AST）		□1 U/L　□2Other：_____	□1	□2	□3		□0
Total Bilirubin（TBIL）		□1 μmol/L　□2Other：_____	□1	□2	□3		□0
Albumin（ALB）		□1 g/L　□2Other：_____	□1	□2	□3		□0
γ- glutamyl transpeptidas（GGT）		□1 U/L　□2Other：_____	□1	□2	□3		□0
Lactic dehydrogenase（LDH）		□1 U/L　□2Other：_____	□1	□2	□3		□0
Creatine kinase（CK）		□1 U/L　□2Other：_____	□1	□2	□3		□0
Creatine kinase isoenzyme（CK-MB）		□1 U/L　□2Other：_____	□1	□2	□3		□0
Blood urea nitrogen（BUN）		□1 mmol/L　□2Other：_____	□1	□2	□3		□0
Urea（Urea）		□1 mmol/L　□2Other：_____	□1	□2	□3		□0
Serum creatinine（CR）		□1 μmol/L　□2Other：_____	□1	□2	□3		□0

Protocol Number: KY2020-29	Attachement Out of Plan
Center Number |__|__| Subject Screening Number |__|__|__|__|__|__|	

Urine Test □0Unexamined, reason____________________

Date: |__|__|__|__|YEAR|__|__|MONTH|__|__|DAY Normal range Number: |__|__|__|__|__|

Item	Result		Result Interpretation			Unexamined
	Measured Value	Unit	Normal	Abnormal		
				No Clinical significance	Significant clinically	
Urine Protein		□1Not Applicable □2Other: _____	□1	□2	□3	□0
Urine Glucose		□1 mmol/L □2Other: _____	□1	□2	□3	□0
Urine PH value		□1 Not Applicable □2Other: _____	□1	□2	□3	□0
Urine Erythrocyte		□1Not Applicable □2Other: _____	□1	□2	□3	□0
Urine Leukocyte		□1Not Applicable □2Other: _____	□1	□2	□3	□0
Urine Ketone		□1Not Applicable □2Other: _____	□1	□2	□3	□0

Urine Test □0Unexamined, reason____________________

Date: |__|__|__|__|YEAR|__|__|MONTH|__|__|DAY Normal range Number: |__|__|__|__|__|

Item	Result		Result Interpretation			Unexamined
	Measured Value	Unit	Normal	Abnormal		
				No Clinical significance	Significant clinically	
Urine Protein		□1Not Applicable □2Other: _____	□1	□2	□3	□0
Urine Glucose		□1 mmol/L □2Other: _____	□1	□2	□3	□0
Urine PH value		□1 Not Applicable □2Other: _____	□1	□2	□3	□0
Urine Erythrocyte		□1Not Applicable □2Other: _____	□1	□2	□3	□0
Urine Leukocyte		□1Not Applicable □2Other: _____	□1	□2	□3	□0
Urine Ketone		□1Not Applicable □2Other: _____	□1	□2	□3	□0

Coagulation Function □0Unexamined, reason______________________

Date: |__|__|__|__|YEAR|__|__|MONTH|__|__|DAY Normal range Number: |__|__|__|__|

Item	Result		Result Interpretation			Unexamined
	Measured Value	Unit	Normal	Abnormal: No Clinical significance	Abnormal: Significant clinically	
ProthrombinTime (PT)		□1 S □2Other: _____	□1	□2	□3	□0
Activated Partial Thromboplastin Time (APTT)		□1 S □2Other: _____	□1	□2	□3	□0
Thrombin Time (TT)		□1 S □2Other: _____	□1	□2	□3	□0
Fibrinogen (Fbg)		□1 g/L □2Other: _____	□1	□2	□3	□0
International Normalized Ratio (INR)		□1Not Applicable □2Other: _____	□1	□2	□3	□0

Coagulation Function □0Unexamined, reason______________________

Date: |__|__|__|__|YEAR|__|__|MONTH|__|__|DAY Normal range Number: |__|__|__|__|

Item	Result		Result Interpretation			Unexamined
	Measured Value	Unit	Normal	Abnormal: No Clinical significance	Abnormal: Significant clinically	
ProthrombinTime (PT)		□1 S □2Other: _____	□1	□2	□3	□0
Activated Partial Thromboplastin Time (APTT)		□1 S □2Other: _____	□1	□2	□3	□0
Thrombin Time (TT)		□1 S □2Other: _____	□1	□2	□3	□0
Fibrinogen (Fbg)		□1 g/L □2Other: _____	□1	□2	□3	□0
International Normalized Ratio (INR)		□1Not Applicable □2Other: _____	□1	□2	□3	□0

C-REACTION PROTEIN（CRP） □0Unexamined, reason________________

Date: |__|__|__|__|YEAR|__|__|MONTH|__|__|DAY Normal range Number: |__|__|__|__|

Item	Result		Result Interpretation		
				Abnormal	
C-REACTION PROTEIN（CRP）	Measured Value	Unit	Normal	No Clinical significance	Significant clinically
		□1 mg/L □2Other: _____	□1	□2	□3

C-REACTION PROTEIN（CRP） □0Unexamined, reason________________

Date: |__|__|__|__|YEAR|__|__|MONTH|__|__|DAY Normal range Number: |__|__|__|__|

Item	Result		Result Interpretation		
				Abnormal	
C-REACTION PROTEIN（CRP）	Measured Value	Unit	Normal	No Clinical significance	Significant clinically
		□1 mg/L □2Other: _____	□1	□2	□3

C-REACTION PROTEIN（CRP） □0Unexamined, reason________________

Date: |__|__|__|__|YEAR|__|__|MONTH|__|__|DAY Normal range Number: |__|__|__|__|

Item	Result		Result Interpretation		
				Abnormal	
C-REACTION PROTEIN（CRP）	Measured Value	Unit	Normal	No Clinical significance	Significant clinically
		□1 mg/L □2Other: _____	□1	□2	□3

C-REACTION PROTEIN（CRP） □0Unexamined, reason________________

Date: |__|__|__|__|YEAR|__|__|MONTH|__|__|DAY Normal range Number: |__|__|__|__|

Item	Result		Result Interpretation		
				Abnormal	
C-REACTION PROTEIN（CRP）	Measured Value	Unit	Normal	No Clinical significance	Significant clinically
		□1 mg/L □2Other: _____	□1	□2	□3

**Erythrocyte Sedimentation Rate（ESR）** □0Unexamined, reason________					
Date : |__|__|__|__|YEAR|__|__|MONTH|__|__|DAY Normal range Number: |__|__|__|__|__|					
**Item**	**Result**		**Result Interpretation**		
				**Abnormal**	
Erythrocyte Sedimentation Rate (ESR)	**Measured Value**	**Unit**	**Normal**	**No Clinical significance**	**Significant clinically**
		□1 mm/h □2Other: _____	□1	□2	□3

**Erythrocyte Sedimentation Rate（ESR）** □0Unexamined, reason________					
Date : |__|__|__|__|YEAR|__|__|MONTH|__|__|DAY Normal range Number: |__|__|__|__|__|					
**Item**	**Result**		**Result Interpretation**		
				**Abnormal**	
Erythrocyte Sedimentation Rate (ESR)	**Measured Value**	**Unit**	**Normal**	**No Clinical significance**	**Significant clinically**
		□1 mm/h □2Other: _____	□1	□2	□3

**Erythrocyte Sedimentation Rate（ESR）** □0Unexamined, reason________					
Date : |__|__|__|__|YEAR|__|__|MONTH|__|__|DAY Normal range Number: |__|__|__|__|__|					
**Item**	**Result**		**Result Interpretation**		
				**Abnormal**	
Erythrocyte Sedimentation Rate (ESR)	**Measured Value**	**Unit**	**Normal**	**No Clinical significance**	**Significant clinically**
		□1 mm/h □2Other: _____	□1	□2	□3

**Erythrocyte Sedimentation Rate（ESR）** □0Unexamined, reason________					
Date : |__|__|__|__|YEAR|__|__|MONTH|__|__|DAY Normal range Number: |__|__|__|__|__|					
**Item**	**Result**		**Result Interpretation**		
				**Abnormal**	
Erythrocyte Sedimentation Rate (ESR)	**Measured Value**	**Unit**	**Normal**	**No Clinical significance**	**Significant clinically**
		□1 mm/h □2Other: _____	□1	□2	□3

TNF-α □0Unexamined，reason____________						
Date：|__|__|__|__|YEAR|__|__|MONTH|__|__|DAY Normal range Number：|__|__|__|__|						
Item	Result			Result Interpretation		
	Measured Value	Unit		Normal	Abnormal	
					No Clinical significance	Significant clinically
TNF-α		□1 pg/mL	□2Other：_____	□1	□2	□3

TNF-α □0Unexamined，reason____________						
Date：|__|__|__|__|YEAR|__|__|MONTH|__|__|DAY Normal range Number：|__|__|__|__|						
Item	Result			Result Interpretation		
	Measured Value	Unit		Normal	Abnormal	
					No Clinical significance	Significant clinically
TNF-α		□1 pg/mL	□2Other：_____	□1	□2	□3

TNF-α □0Unexamined，reason____________						
Date：|__|__|__|__|YEAR|__|__|MONTH|__|__|DAY Normal range Number：|__|__|__|__|						
Item	Result			Result Interpretation		
	Measured Value	Unit		Normal	Abnormal	
					No Clinical significance	Significant clinically
TNF-α		□1 pg/mL	□2Other：_____	□1	□2	□3

TNF-α □0Unexamined，reason____________						
Date：|__|__|__|__|YEAR|__|__|MONTH|__|__|DAY Normal range Number：|__|__|__|__|						
Item	Result			Result Interpretation		
	Measured Value	Unit		Normal	Abnormal	
					No Clinical significance	Significant clinically
TNF-α		□1 pg/mL	□2Other：_____	□1	□2	□3

TNF-α □0Unexamined，reason____________						
Date：|__|__|__|__|YEAR|__|__|MONTH|__|__|DAY Normal range Number：|__|__|__|__|						
Item	Result			Result Interpretation		
	Measured Value	Unit		Normal	Abnormal	
					No Clinical significance	Significant clinically
TNF-α		□1 pg/mL	□2Other：_____	□1	□2	□3

IL-6 □0Unexamined，reason________________________________

Date：\|__\|__\|__\|__\|YEAR\|__\|__\|MONTH\|__\|__\|DAY Normal range Number：\|__\|__\|__\|__\|

Item	Result			Result Interpretation		
					Abnormal	
IL-6	Measured Value	Unit		Normal	No Clinical significance	Significant clinically
		□1 pg/mL	□2Other：_____	□1	□2	□3

IL-6 □0Unexamined，reason________________________________

Date：\|__\|__\|__\|__\|YEAR\|__\|__\|MONTH\|__\|__\|DAY Normal range Number：\|__\|__\|__\|__\|

Item	Result			Result Interpretation		
					Abnormal	
IL-6	Measured Value	Unit		Normal	No Clinical significance	Significant clinically
		□1 pg/mL	□2Other：_____	□1	□2	□3

IL-6 □0Unexamined，reason________________________________

Date：\|__\|__\|__\|__\|YEAR\|__\|__\|MONTH\|__\|__\|DAY Normal range Number：\|__\|__\|__\|__\|

Item	Result			Result Interpretation		
					Abnormal	
IL-6	Measured Value	Unit		Normal	No Clinical significance	Significant clinically
		□1 pg/mL	□2Other：_____	□1	□2	□3

IL-6 □0Unexamined，reason________________________________

Date：\|__\|__\|__\|__\|YEAR\|__\|__\|MONTH\|__\|__\|DAY Normal range Number：\|__\|__\|__\|__\|

Item	Result			Result Interpretation		
					Abnormal	
IL-6	Measured Value	Unit		Normal	No Clinical significance	Significant clinically
		□1 pg/mL	□2Other：_____	□1	□2	□3

IL-6 □0Unexamined，reason________________________________

Date：\|__\|__\|__\|__\|YEAR\|__\|__\|MONTH\|__\|__\|DAY Normal range Number：\|__\|__\|__\|__\|

Item	Result			Result Interpretation		
					Abnormal	
IL-6	Measured Value	Unit		Normal	No Clinical significance	Significant clinically
		□1 pg/mL	□2Other：_____	□1	□2	□3

Urine or serum HCG (Only applicable to women of childbearing age) □0Unexamined, reason____ □1Not Applicable（Man or woman who isn't able to reproduce）
Date：|__|__|__|__|YEAR|__|__|MONTH|__|__|DAY
Sample：□1 Blood □2 Urine
Result：□1 Negative □2 Positive

Urine or serum HCG (Only applicable to women of childbearing age) □0Unexamined, reason____ □1Not Applicable（Man or woman who isn't able to reproduce）
Date：|__|__|__|__|YEAR|__|__|MONTH|__|__|DAY
Sample：□1 Blood □2 Urine
Result：□1 Negative □2 Positive

Urine or serum HCG (Only applicable to women of childbearing age) □0Unexamined, reason____ □1Not Applicable（Man or woman who isn't able to reproduce）
Date：|__|__|__|__|YEAR|__|__|MONTH|__|__|DAY
Sample：□1 Blood □2 Urine
Result：□1 Negative □2 Positive

Urine or serum HCG (Only applicable to women of childbearing age) □0Unexamined, reason____ □1Not Applicable（Man or woman who isn't able to reproduce）
Date：|__|__|__|__|YEAR|__|__|MONTH|__|__|DAY
Sample：□1 Blood □2 Urine
Result：□1 Negative □2 Positive

Urine or serum HCG (Only applicable to women of childbearing age) □0Unexamined, reason____ □1Not Applicable（Man or woman who isn't able to reproduce）
Date：|__|__|__|__|YEAR|__|__|MONTH|__|__|DAY

Sample：□1 Blood □2 Urine	
Result：□1 Negative □2 Positive	

Routine 12 Leads Electrocardiogram □0Unexamined，reason________________________
Date：|__|__|__|__|YEAR|__|__|MONTH|__|__|DAY
Result：□1 Normal □2 Abnormal,No Clinical significance □3 AbnormalSignificant clinically
If“Abnormal”，please describe：____

Routine 12 Leads Electrocardiogram □0Unexamined，reason________________________
Date：|__|__|__|__|YEAR|__|__|MONTH|__|__|DAY
Result：□1 Normal □2 Abnormal,No Clinical significance □3 AbnormalSignificant clinically
If“Abnormal”，please describe：____

Routine 12 Leads Electrocardiogram □0Unexamined，reason________________________
Date：|__|__|__|__|YEAR|__|__|MONTH|__|__|DAY
Result：□1 Normal □2 Abnormal,No Clinical significance □3 AbnormalSignificant clinically
If“Abnormal”，please describe：____

Routine 12 Leads Electrocardiogram □0Unexamined，reason________________________
Date：|__|__|__|__|YEAR|__|__|MONTH|__|__|DAY
Result：□1 Normal □2 Abnormal,No Clinical significance □3 AbnormalSignificant clinically
If“Abnormal”，please describe：____

Routine 12 Leads Electrocardiogram □0Unexamined，reason________________________
Date：|__|__|__|__|YEAR|__|__|MONTH|__|__|DAY
Result：□1 Normal □2 Abnormal,No Clinical significance □3 AbnormalSignificant clinically
If“Abnormal”，please describe：____

Routine 12 Leads Electrocardiogram □0Unexamined，reason________________________
Date：|__|__|__|__|YEAR|__|__|MONTH|__|__|DAY
Result：□1 Normal □2 Abnormal,No Clinical significance □3 AbnormalSignificant clinically
If“Abnormal”，please describe：____

**Nucleic Acid Test of 2019-nCov**	□0 Unexamined，reason______________________ □1 Not Applicable
Sampling Part： □1Upper respiratory tract □2Lower respiratory tract	
Date： |__|__|__|__|YEAR|__|__|MONTH|__|__|DAY	Date： |__|__|:|__|__|
Result： □1 Negative □2 Positive □3 Unidentified □4 Other，please describe____________	

**Nucleic Acid Test of 2019-nCov**	□0 Unexamined，reason______________________ □1 Not Applicable
Sampling Part： □1Upper respiratory tract □2Lower respiratory tract	
Date： |__|__|__|__|YEAR|__|__|MONTH|__|__|DAY	Date： |__|__|:|__|__|
Result： □1 Negative □2 Positive □3 Unidentified □4 Other，please describe____________	

**Nucleic Acid Test of 2019-nCov**	□0 Unexamined，reason______________________ □1 Not Applicable
Sampling Part： □1Upper respiratory tract □2Lower respiratory tract	
Date： |__|__|__|__|YEAR|__|__|MONTH|__|__|DAY	Date： |__|__|:|__|__|
Result： □1 Negative □2 Positive □3 Unidentified □4 Other，please describe____________	

**Nucleic Acid Test of 2019-nCov**	□0 Unexamined，reason______________________ □1 Not Applicable
Sampling Part： □1Upper respiratory tract □2Lower respiratory tract	
Date： |__|__|__|__|YEAR|__|__|MONTH|__|__|DAY	Date： |__|__|:|__|__|
Result： □1 Negative □2 Positive □3 Unidentified □4 Other，please describe____________	

**Nucleic Acid Test of 2019-nCov**	□0 Unexamined，reason______________________ □1 Not Applicable
Sampling Part： □1Upper respiratory tract □2Lower respiratory tract	
Date： |__|__|__|__|YEAR|__|__|MONTH|__|__|DAY	Date： |__|__|:|__|__|
Result： □1 Negative □2 Positive □3 Unidentified □4 Other，please describe____________	

Pulse oxygen saturation（without oxygen inhalation ） □0Unexamined，reason______
Date：|__|__|__|__|YEAR|__|__|MONTH|__|__|DAY
Measured Value：______________%（Rest state）

Pulse oxygen saturation（without oxygen inhalation ） □0Unexamined，reason______
Date：|__|__|__|__|YEAR|__|__|MONTH|__|__|DAY
Measured Value：______________%（Rest state）

Pulse oxygen saturation（without oxygen inhalation ） □0Unexamined，reason______
Date：|__|__|__|__|YEAR|__|__|MONTH|__|__|DAY
Measured Value：______________%（Rest state）

Pulse oxygen saturation（without oxygen inhalation ） □0Unexamined，reason______
Date：|__|__|__|__|YEAR|__|__|MONTH|__|__|DAY
Measured Value：______________%（Rest state）

Pulse oxygen saturation（without oxygen inhalation ） □0Unexamined，reason______
Date：|__|__|__|__|YEAR|__|__|MONTH|__|__|DAY
Measured Value：______________%（Rest state）

Pulse oxygen saturation（without oxygen inhalation ） □0Unexamined，reason______
Date：|__|__|__|__|YEAR|__|__|MONTH|__|__|DAY
Measured Value：______________%（Rest state）

Pulse oxygen saturation（without oxygen inhalation ） □0Unexamined，reason______
Date：|__|__|__|__|YEAR|__|__|MONTH|__|__|DAY
Measured Value：______________%（Rest state）

**Chest CT** □0Unexamined，reason____
Date：|__|__|__|__|__|YEAR|__|__|MONTH|__|__|DAY
Result：□1 Normal □2 Abnormal,No Clinical significance □3 Abnormal，significant clinically _
If abnormal, please describe ________________________________
Wheather chest CT lesion absorbed⩾50% than baseline??   □0 NO □1 YES □2 Unidentified □3 Unknown   □4 Other，please describe：__________________________________

**Chest CT** □0Unexamined，reason____
Date：|__|__|__|__|__|YEAR|__|__|MONTH|__|__|DAY
Result：□1 Normal □2 Abnormal,No Clinical significance □3 Abnormal，significant clinically _
If abnormal, please describe ________________________________
Wheather chest CT lesion absorbed⩾50% than baseline??   □0 NO □1 YES □2 Unidentified □3 Unknown   □4 Other，please describe：__________________________________

**Chest CT** □0Unexamined，reason____
Date：|__|__|__|__|__|YEAR|__|__|MONTH|__|__|DAY
Result：□1 Normal □2 Abnormal,No Clinical significance □3 Abnormal，significant clinically _
If abnormal, please describe ________________________________
Wheather chest CT lesion absorbed⩾50% than baseline??   □0 NO □1 YES □2 Unidentified □3 Unknown   □4 Other，please describe：__________________________________

**Arterial Blood Gas Analysis** ☐0Unexamined, reason________________						
Inhalate oxygen or not? ☐0 No ☐1Yes,（FiO2）:______________%						
Date: |__|__|__|__|YEAR|__|__|MONTH|__|__|DAY						
**Item**	**Result**		**Result Interpretation**			**Unexamined**
				**Abnormal**		
	**Measured Value**	**Unit**	**Normal**	**No Clinical significance**	**Significant clinically**	
Arterial partial pressure of oxygen（PaO2）		☐1 mmHg ☐2 Other:_____	☐1	☐2	☐3	☐0
Oxygen saturation (SaO2)		☐1 % ☐2 Other:_____	☐1	☐2	☐3	☐0

**Arterial Blood Gas Analysis** ☐0Unexamined, reason________________						
Inhalate oxygen or not? ☐0 No ☐1Yes,（FiO2）:______________%						
Date: |__|__|__|__|YEAR|__|__|MONTH|__|__|DAY						
**Item**	**Result**		**Result Interpretation**			**Unexamined**
				**Abnormal**		
	**Measured Value**	**Unit**	**Normal**	**No Clinical significance**	**Significant clinically**	
Arterial partial pressure of oxygen（PaO2）		☐1 mmHg ☐2 Other:_____	☐1	☐2	☐3	☐0
Oxygen saturation (SaO2)		☐1 % ☐2 Other:_____	☐1	☐2	☐3	☐0

**Clinical status**	□0 No evaluation，reason____
Date：	|__|__|__|__|YEAR|__|__|MONTH|__|__|DAY
Result	□1 Level 1: discharge<br>□2 Level 2: hospitalization，oxygen therapy is not required<br>□3 Level 3: hospitalization，require oxygen therapy<br>□4 Level 4:ICU/ hospitalization，require noninvasive ventilation (NIV)/ High flow nasal catheter oxygen therapy (HFNC)<br>□5 Level 5:ICU，require extracorporeal membrane oxygenation (ECMO)and/or invasive mechanical ventilation(IMV)<br>□6 Level 6:Death

**Clinical status**	□0 No evaluation，reason____
Date：	|__|__|__|__|YEAR|__|__|MONTH|__|__|DAY
Result	□1 Level 1: discharge<br>□2 Level 2: hospitalization，oxygen therapy is not required<br>□3 Level 3: hospitalization，require oxygen therapy<br>□4 Level 4:ICU/ hospitalization，require noninvasive ventilation (NIV)/ High flow nasal catheter oxygen therapy (HFNC)<br>□5 Level 5:ICU，require extracorporeal membrane oxygenation (ECMO)and/or invasive mechanical ventilation(IMV)<br>□6 Level 6:Death

**Clinical status**	□0 No evaluation，reason____
Date：	|__|__|__|__|YEAR|__|__|MONTH|__|__|DAY
Result	□1 Level 1: discharge<br>□2 Level 2: hospitalization，oxygen therapy is not required<br>□3 Level 3: hospitalization，require oxygen therapy<br>□4 Level 4:ICU/ hospitalization，require noninvasive ventilation (NIV)/ High flow nasal catheter oxygen therapy (HFNC)<br>□5 Level 5:ICU，require extracorporeal membrane oxygenation (ECMO)and/or invasive mechanical ventilation(IMV)<br>□6 Level 6:Death

Signment of the Informed Consent Form (ICF)	
Date of signing the Informed Consent	|__|__|__|__|YEAR|__|__|MONTH|__|__|DAY
Version number of the Informed Consent	____________
The version number of the protocol shall apply when the Informed Consent is signed	__|__|.|__|

Signment of the Informed Consent Form (ICF)	
Date of signing the Informed Consent	|__|__|__|__|YEAR|__|__|MONTH|__|__|DAY
Version number of the Informed Consent	____________
The version number of the protocol shall apply when the Informed Consent is signed	__|__|.|__|

Signment of the Informed Consent Form (ICF)	
Date of signing the Informed Consent	|__|__|__|__|YEAR|__|__|MONTH|__|__|DAY
Version number of the Informed Consent	____________
The version number of the protocol shall apply when the Informed Consent is signed	__|__|.|__|

Signment of the Informed Consent Form (ICF)	
Date of signing the Informed Consent	|__|__|__|__|YEAR|__|__|MONTH|__|__|DAY
Version number of the Informed Consent	____________
The version number of the protocol shall apply when the Informed Consent is signed	__|__|.|__|

Signment of the Informed Consent Form (ICF)	
Date of signing the Informed Consent	|__|__|__|__|YEAR|__|__|MONTH|__|__|DAY
Version number of the Informed Consent	____________
The version number of the protocol shall apply when the Informed Consent is signed	__|__|.|__|

Final Case Report Form: KY2020-29

This is the original version of the final Case Report Form in Chinese.

硫酸羟氯喹治疗新型冠状病毒肺炎的一项随机、对照、开放的多中心研究
病例报告表
（Case Report Form）

中 心 编 号： |__|__|

受 试 者 筛 选 号 ： |__|__|__|__|__|

受试者姓名缩写： |__|__|__|__|

研究中心名称（正楷）： ______________________

研究医生姓名（正楷）： ______________________

版 本 号 及 日 期： 3.0 版/2020 年 2 月 25 日

适 用 临 床 分 型 ： 轻型/普通型

保密声明

本文件包含的相关信息涉及商业机密和商业信息，这些商业信息术语保密内容或专有信息，若非法规要求，任何人不得擅自公开这些保密内容。任何接触到该文件的个人应当了解文件的专有性或机密性，并不得擅自公开文件。以上文件保密方面的限制条款将适用于未来提供给你的任何注明具有专有性或机密性的资料。

填表指南

1. 病例报告表须由授权人员负责观察记录，并对CRF中的每一页认真检查并签字。

2. 试验医师应熟悉试验方案，并掌握GCP原则。

3. 请用蓝色或黑色签字笔填写，字迹应清晰，易于辨认，英文最好用大写字母，例如：CASE REPORT FORM。

4. 在开放的方格中填写数字时，每一格填写一个数字；如果数字位数少于相应的格子数时请以零填充，不可出现空格。例如：56Kg，填入空格中为：| 0 | 5 | 6 |. | 0 | 0 |Kg。

5. 日期采用国际ISO8601日期格式：yyyymmdd。例如2007年12月21日写成| 2 |0 | 0 | 7 |年| 1 | 2 |月| 2 | 1 |日。

6. 表中凡有“□”的项目，请在符合的方框内打“×”，或填写相关数字，如果未做或数据无法获得，请在相应空格上填写ND（未完成）或NK（无法获知）；不适用填写NA。

7. 每项填写内容务必准确，不得随便涂改，禁止使用涂改液或以其他方式掩盖原始数据。如发现填写内容有误，请用单横线划去错误的记录，使原有记录仍可辨认，在右上方填写正确记录，并注明修改者的姓名缩写及修改日期。如：~~58.6~~ 56.8 ZH 2007-12-21。

8. 患者姓名拼音缩写四格应填满，两字姓名按每个字前两个字母填写，三字姓名按三字的首字母和第三个字的第二个字母填写；四字姓名填写每一个字的首字母。每位患者姓名的拼音缩写应在其CRF中统一。举例：张红| Z | H | H | 0 |，李淑明| L | S |M | I |，欧阳小惠| O | Y | X | H |。

9. 不要改变病例报告表的格式，如发现表中没有填写位置填写记录者希望记录的资料时，请联系负责的CRA。

10. 病例报告表填写完成后，主要研究者应检查病例报告表的每一页记录，并在病例报告表的研究者声明处签字。

本次访视日期：|__|__|__|__|年|__|__|月|__|__|日

**签署知情同意书**	
是否签署知情同意书	□0 否　　□1 是
签署知情同意书日期	|__|__|__|__|年|__|__|月|__|__|日
知情同意书版本号	____________________
签署知情同意书时适用方案版本号	|__|__|.|__|

**确认入选/排除标准**	
受试者是否满足所有入选标准且不满足所有排除标准？　□1 是　□0 否，请填写。	
□1 不满足入选标准，入选标准编号（可多选）	□ 1　□ 2　□ 3　□ 4　□ 5　□ 6　□ 7<br>□ 其他，请描述：________________
□2 满足排除标准，排除标准编号（可多选）	□ 1　□ 2　□ 3　□ 4　□ 5　□ 6　□ 7<br>□ 8　□ 9　□ 其他，请描述：__________

**人口学资料**	
性　　别	□1 男　□2 女
出 生 日 期	|__|__|__|__|年|__|__|月|__|__|日　如不能获得，请填写年龄：|__|__| 岁
民　　族	□1 汉　□2 其他，请描述：____________________

**新型冠状病毒肺炎病史**	
首次发病日期	|__|__|__|__|年|__|__|月|__|__|日
发病前 14 天内有无相关病源接触史	□0 无　□1 有　□2 未知
接触类型（可多选）	□1 接触湖北地区<br>□2 接触新型冠状病毒感染者<br>□3 未知<br>□4 其他，请描述 ：____
既往有无进行抗新型冠状病毒肺炎治疗	□0 无　□1 有
首次核酸检测阳性报告日期	|__|__|__|__|年|__|__|月|__|__|日
入组时临床分型	□1 轻型　□2 普通型　□3 重型　□4 危重型

注：如进行抗新型冠状病毒肺炎治疗，请在“既往/合并药物治疗”和“既往/合并非药物治疗”表中填写。

药物过敏史

受试者有无药物过敏史？

☐0 无　　☐1 有，请描述 ：________________________________

____

既往/伴随疾病

受试者是否有除新型冠状病毒肺炎之外的既往/伴随疾病？　☐0 否　☐1 是，请填写下表。

编号	疾病名称	确诊日期	目前是否持续？	结束日期	是否接受治疗
1		|__|__|__|__|年<br>|__|__|月|__|__|日	☐0 否<br>☐1 是	|__|__|__|__|年<br>|__|__|月|__|__|日	☐0 否<br>☐1 是
2		|__|__|__|__|年<br>|__|__|月|__|__|日	☐0 否<br>☐1 是	|__|__|__|__|年<br>|__|__|月|__|__|日	☐0 否<br>☐1 是
3		|__|__|__|__|年<br>|__|__|月|__|__|日	☐0 否<br>☐1 是	|__|__|__|__|年<br>|__|__|月|__|__|日	☐0 否<br>☐1 是
4		|__|__|__|__|年<br>|__|__|月|__|__|日	☐0 否<br>☐1 是	|__|__|__|__|年<br>|__|__|月|__|__|日	☐0 否<br>☐1 是
5		|__|__|__|__|年<br>|__|__|月|__|__|日	☐0 否<br>☐1 是	|__|__|__|__|年<br>|__|__|月|__|__|日	☐0 否<br>☐1 是
6		|__|__|__|__|年<br>|__|__|月|__|__|日	☐0 否<br>☐1 是	|__|__|__|__|年<br>|__|__|月|__|__|日	☐0 否<br>☐1 是
7		|__|__|__|__|年<br>|__|__|月|__|__|日	☐0 否<br>☐1 是	|__|__|__|__|年<br>|__|__|月|__|__|日	☐0 否<br>☐1 是
8		|__|__|__|__|年<br>|__|__|月|__|__|日	☐0 否<br>☐1 是	|__|__|__|__|年<br>|__|__|月|__|__|日	☐0 否<br>☐1 是
9		|__|__|__|__|年<br>|__|__|月|__|__|日	☐0 否<br>☐1 是	|__|__|__|__|年<br>|__|__|月|__|__|日	☐0 否<br>☐1 是
10		|__|__|__|__|年<br>|__|__|月|__|__|日	☐0 否<br>☐1 是	|__|__|__|__|年<br>|__|__|月|__|__|日	☐0 否<br>☐1 是

生命体征　☐0 未查，原因________________________________

检查日期	|__|__|__|__|年|__|__|月|__|__|日		
日最高体温	|__|__|.|__|℃	测量方式	☐1 腋下　☐2 口腔　☐3 肛门<br>☐4 耳朵　☐5 未知　☐6 其他，请描述_______
呼吸频率	|__|__|次/分	脉搏	|__|__|__|次/分

血压	|__|__|__|/|__|__|__| mmHg（收缩压/舒张压）

**体格检查** ☐0 未查，原因________________					
检查日期：|__|__|__|__|年|__|__|月|__|__|日					
**检查部位**	**检查结果**<br>**1=正常**<br>**2=异常无临床意义**<br>**3=异常有临床意义**<br>**0=未查**				**异常请描述**
整体外观	☐1	☐2	☐3	☐0	
头	☐1	☐2	☐3	☐0	
眼	☐1	☐2	☐3	☐0	
耳	☐1	☐2	☐3	☐0	
鼻	☐1	☐2	☐3	☐0	
喉	☐1	☐2	☐3	☐0	
颈部及甲状腺	☐1	☐2	☐3	☐0	
皮肤	☐1	☐2	☐3	☐0	
心血管系统	☐1	☐2	☐3	☐0	
胸部及肺	☐1	☐2	☐3	☐0	
腹部	☐1	☐2	☐3	☐0	
神经系统	☐1	☐2	☐3	☐0	
骨骼肌系统	☐1	☐2	☐3	☐0	
淋巴系统	☐1	☐2	☐3	☐0	

**有无呼吸道症状** ☐0 未查，原因________________
检查日期：|__|__|__|__|年|__|__|月|__|__|日
受试者有无呼吸道症状：☐0 无 ☐1 有，请描述：________________<br>________________

血常规 □0 未查，原因________________

采样日期：|__|__|__|__|年|__|__|月|__|__|日 正常值范围编号：|__|__|__|__|

检查项目	测量结果		结果判定			未查
	实测值	单位	正常	异常		
				无临床意义	有临床意义	
红细胞计数（RBC）		□1 10^{12}/L □2 其他：_____	□1	□2	□3	□0
白细胞计数（WBC）		□1 10^9/L □2 其他：_____	□1	□2	□3	□0
血红蛋白（Hb）		□1 g/L □2 其他：_____	□1	□2	□3	□0
血小板计数（PLT）		□1 10^9/L □2 其他：_____	□1	□2	□3	□0
中性粒细胞计数（ANC）		□1 10^9/L □2 其他：_____	□1	□2	□3	□0
淋巴细胞计数（LY）		□1 10^9/L □2 其他：_____	□1	□2	□3	□0
单核细胞计数（MO）		□1 10^9/L □2 其他：_____	□1	□2	□3	□0
嗜酸性粒细胞计数（EO）		□1 10^9/L □2 其他：_____	□1	□2	□3	□0
嗜碱性粒细胞计数（BA）		□1 10^9/L □2 其他：_____	□1	□2	□3	□0

血生化 □0 未查，原因________________

采样日期|__|__|__|__|年|__|__|月|__|__|日 正常值范围编号：|__|__|__|__|

检查项目	测量结果		结果判定			未查
	实测值	单位	正常	异常		
				无临床意义	有临床意义	
谷丙转氨酶（ALT）		□1 U/L □2 其他：_____	□1	□2	□3	□0
谷草转氨酶（AST）		□1 U/L □2 其他：_____	□1	□2	□3	□0
总胆红素（TBIL）		□1 μmol/L □2 其他：_____	□1	□2	□3	□0
白蛋白（ALB）		□1 g/L □2 其他：_____	□1	□2	□3	□0
γ-谷氨酰转肽酶（GGT）		□1 U/L □2 其他：_____	□1	□2	□3	□0
乳酸脱氢酶（LDH）		□1 U/L □2 其他：_____	□1	□2	□3	□0
肌酸激酶（CK）		□1 U/L □2 其他：_____	□1	□2	□3	□0
肌酸激酶同工酶（CK-MB）		□1 U/L □2 其他：_____	□1	□2	□3	□0
血尿素氮（BUN）		□1 mmol/L □2 其他：_____	□1	□2	□3	□0
尿素（Urea）		□1 mmol/L □2 其他：_____	□1	□2	□3	□0
血肌酐（CR）		□1 μmol/L □2 其他：_____	□1	□2	□3	□0

尿常规 □0 未查，原因____						
采样日期：|__|__|__|__|年|__|__|月|__|__|日			正常值范围编号：|__|__|__|__|			
检查项目	测量结果		结果判定			未查
	实测值	单位	正常	异常		
				无临床意义	有临床意义	
尿蛋白		□1 不适用 □2 其他：_____	□1	□2	□3	□0
尿葡萄糖		□1 mmol/L □2 其他：_____	□1	□2	□3	□0
尿 PH 值		□1 不适用 □2 其他：_____	□1	□2	□3	□0
尿红细胞		□1 不适用 □2 其他：_____	□1	□2	□3	□0
尿白细胞		□1 不适用 □2 其他：_____	□1	□2	□3	□0
尿酮体		□1 不适用 □2 其他：_____	□1	□2	□3	□0

凝血功能 □0 未查，原因____						
采样日期：|__|__|__|__|年|__|__|月|__|__|日			正常值范围编号：|__|__|__|__|			
检查项目	测量结果		结果判定			未查
	实测值	单位	正常	异常		
				无临床意义	有临床意义	
凝血酶原时间（PT）		□1 S □2 其他：_____	□1	□2	□3	□0
活化部分凝血活酶时间（APTT）		□1 S □2 其他：_____	□1	□2	□3	□0
凝血酶时间（TT）		□1 S □2 其他：_____	□1	□2	□3	□0
血浆纤维蛋白原（Fbg）		□1 g/L □2 其他：_____	□1	□2	□3	□0
国际标准化比率（INR）		□1 不适用 □2 其他：_____	□1	□2	□3	□0

C 反应蛋白（CRP） □0 未查，原因____					
采样日期：|__|__|__|__|年|__|__|月|__|__|日			正常值范围编号：|__|__|__|__|		
检查项目	测量结果		结果判定		
C 反应蛋白（CRP）	实测值	单位	正常	异常	
				无临床意义	有临床意义
		□1 mg/L □2 其他：_____	□1	□2	□3

血沉（ESR） □0 未查，原因____				
采样日期：|__|__|__|__|年|__|__|月|__|__|日			正常值范围编号：|__|__|__|__|	
检查项目	测量结果		结果判定	
	实测值	单位	正常	异常

				无临床意义	有临床意义
血沉（ESR）		□1 mm/h □2其他：_____	□1	□2	□3

TNF-α □0 未查，原因____

采样日期：|__|__|__|__|年|__|__|月|__|__|日 正常值范围编号：|__|__|__|__|__|

检查项目	测量结果		结果判定		
				异常	
	实测值	单位	正常	无临床意义	有临床意义
TNF-α		□1 pg/mL □2其他：_____	□1	□2	□3

IL-6 □0 未查，原因____

采样日期：|__|__|__|__|年|__|__|月|__|__|日 正常值范围编号：|__|__|__|__|__|

检查项目	测量结果		结果判定		
				异常	
	实测值	单位	正常	无临床意义	有临床意义
IL-6		□1 pg/mL □2其他：_____	□1	□2	□3

尿或血清 HCG（仅适用于育龄女性） □0 未查，原因____________________________
□1 不适用（男性或不具有生育能力的女性）

采样日期：|__|__|__|__|年|__|__|月|__|__|日 正常值范围编号：|__|__|__|__|__|

采样类别：□1 血液 □2 尿液

检查结果：□1 阴性 □2 阳性 □3 其他：______________________

单位： □1 不适用 □2 mIU/mL □3 其他：______________________

12 导联心电图 □0 未查，原因____

检查日期：|__|__|__|__|年|__|__|月|__|__|日

检查结果：□1 正常 □2 异常无临床意义 □3 异常有临床意义

若“异常”，请描述：____

____

胸部 CT □0 未查，原因____

检查日期：|__|__|__|__|年|__|__|月|__|__|日

检查结果：□1 正常 □2 异常无临床意义 □3 异常有临床意义

若异常，请描述：____

**新型冠状病毒核酸检测**	□0 未查，原因______________________
采样部位（可多选）	□0 上呼吸道 □1 下呼吸道
上呼吸道	采样日期：|__|__|__|__|年|__|__|月|__|__|日<br>检查结果：□1 阴性 □2 阳性 □3 无法判断 □4 其他，请描述________
下呼吸道	采样日期：|__|__|__|__|年|__|__|月|__|__|日<br>检查结果：□1 阴性 □2 阳性 □3 无法判断 □4 其他，请描述________
结果判定	□1 阴性 □2 阳性 □3 无法判断 □4 其他，请描述______________

**指脉氧饱和度**（不吸氧情况下） □0 未查，原因______________________
检查日期：|__|__|__|__|年|__|__|月|__|__|日
实测值：____________%（静息状态）

**筛选结果**
受试者是否筛选成功？<br>□0 否，筛选失败的主要原因：______________________<br>□1 是，请进行随机化。
随机日期：|__|__|__|__|年|__|__|月|__|__|日
随机编号：________________
随机分组：□1 试验组 □2 对照组

自签署知情同意书以来，如有不良事件发生，请填写“不良事件”表；如有合并药物治疗，请填写“既往/合并药物治疗”表；如有合并非药物治疗，请填写“既往/合并非药物治疗”表；如有计划外检查，请填写“计划外检查”表；如有 IMV/NIV、ECMO、HFNC、氧疗，请填写在“IMV/NIV、ECMO、 HFNC、氧疗”表中。

本次访视日期：|__|__|__|__|年|__|__|月|__|__|日

**生命体征** □0 未查，原因________			
检查日期	|__|__|__|__|年|__|__|月|__|__|日		
日最高体温	|__|__|.|__|℃	测量方式	□1 腋下 □2 口腔 □3 肛门<br>□4 耳朵 □5 未知 □6 其他，请描述______
呼吸频率	|__|__|次/分	脉搏	|__|__|__|次/分
血压	|__|__|__|/|__|__|__| mmHg（收缩压/舒张压）		

**有无呼吸道症状** □0 未查，原因________
检查日期：|__|__|__|__|年|__|__|月|__|__|日
受试者有无呼吸道症状：□0 无 □1 有，请描述：________

血常规 □0 未查，原因________

采样日期：|__|__|__|__|年|__|__|月|__|__|日 正常值范围编号：|__|__|__|__|

检查项目	测量结果		结果判定			未查
	实测值	单位	正常	异常		
				无临床意义	有临床意义	
红细胞计数（RBC）		□1 10^{12}/L □2 其他：____	□1	□2	□3	□0
白细胞计数（WBC）		□1 10^9/L □2 其他：____	□1	□2	□3	□0
血红蛋白（Hb）		□1 g/L □2 其他：____	□1	□2	□3	□0
血小板计数（PLT）		□1 10^9/L □2 其他：____	□1	□2	□3	□0
中性粒细胞计数（ANC）		□1 10^9/L □2 其他：____	□1	□2	□3	□0
淋巴细胞计数（LY）		□1 10^9/L □2 其他：____	□1	□2	□3	□0
单核细胞计数（MO）		□1 10^9/L □2 其他：____	□1	□2	□3	□0
嗜酸性粒细胞计数（EO）		□1 10^9/L □2 其他：____	□1	□2	□3	□0
嗜碱性粒细胞计数（BA）		□1 10^9/L □2 其他：____	□1	□2	□3	□0

血生化 □0 未查，原因________

采样日期|__|__|__|__|年|__|__|月|__|__|日 正常值范围编号：|__|__|__|__|

检查项目	测量结果		结果判定			未查
	实测值	单位	正常	异常		
				无临床意义	有临床意义	

谷丙转氨酶（ALT）		□$_1$ U/L □$_2$其他：_____	□$_1$	□$_2$	□$_3$	□$_0$
谷草转氨酶（AST）		□$_1$ U/L □$_2$其他：_____	□$_1$	□$_2$	□$_3$	□$_0$
总胆红素（TBIL）		□$_1$ μmol/L □$_2$其他：_____	□$_1$	□$_2$	□$_3$	□$_0$
白蛋白（ALB）		□$_1$ g/L □$_2$其他：_____	□$_1$	□$_2$	□$_3$	□$_0$
γ-谷氨酰转肽酶（GGT）		□$_1$ U/L □$_2$其他：_____	□$_1$	□$_2$	□$_3$	□$_0$
乳酸脱氢酶（LDH）		□$_1$ U/L □$_2$其他：_____	□$_1$	□$_2$	□$_3$	□$_0$
肌酸激酶（CK）		□$_1$ U/L □$_2$其他：_____	□$_1$	□$_2$	□$_3$	□$_0$
肌酸激酶同工酶（CK-MB）		□$_1$ U/L □$_2$其他：_____	□$_1$	□$_2$	□$_3$	□$_0$
血尿素氮（BUN）		□$_1$ mmol/L □$_2$其他：_____	□$_1$	□$_2$	□$_3$	□$_0$
尿素（Urea）		□$_1$ mmol/L □$_2$其他：_____	□$_1$	□$_2$	□$_3$	□$_0$
血肌酐（CR）		□$_1$ μmol/L □$_2$其他：_____	□$_1$	□$_2$	□$_3$	□$_0$

尿常规 □$_0$ 未查，原因________________________________

□$_1$ 不适用

采样日期：|__|__|__|__|年|__|__|月|__|__|日 正常值范围编号：|__|__|__|__|

检查项目	测量结果		结果判定			未查
	实测值	单位	正常	异常		
				无临床意义	有临床意义	
尿蛋白		□$_1$不适用 □$_2$其他：_____	□$_1$	□$_2$	□$_3$	□$_0$
尿葡萄糖		□$_1$ mmol/L □$_2$其他：_____	□$_1$	□$_2$	□$_3$	□$_0$
尿 PH 值		□$_1$ 不适用 □$_2$其他：_____	□$_1$	□$_2$	□$_3$	□$_0$
尿红细胞		□$_1$不适用 □$_2$其他：_____	□$_1$	□$_2$	□$_3$	□$_0$
尿白细胞		□$_1$不适用 □$_2$其他：_____	□$_1$	□$_2$	□$_3$	□$_0$
尿酮体		□$_1$不适用 □$_2$其他：_____	□$_1$	□$_2$	□$_3$	□$_0$

凝血功能 □$_0$ 未查，原因________________________________

采样日期：|__|__|__|__|年|__|__|月|__|__|日 正常值范围编号：|__|__|__|__|

检查项目	测量结果		结果判定			未查
	实测值	单位	正常	异常		
				无临床意义	有临床意义	
凝血酶原时间（PT）		□$_1$ S □$_2$其他：_____	□$_1$	□$_2$	□$_3$	□$_0$
活化部分凝血活酶时间（APTT）		□$_1$ S □$_2$其他：_____	□$_1$	□$_2$	□$_3$	□$_0$

凝血酶时间（TT）		□1 S　□2 其他：_____	□1	□2	□3	□0
血浆纤维蛋白原（Fbg）		□1 g/L　□2 其他：_____	□1	□2	□3	□0
国际标准化比率（INR）		□1 不适用　□2 其他：_____	□1	□2	□3	□0

C 反应蛋白（CRP）　□0 未查，原因____

采样日期：\|__\|__\|__\|__\|年\|__\|__\|月\|__\|__\|日　　正常值范围编号：\|__\|__\|__\|__\|

检查项目	测量结果		结果判定		
	实测值	单位	正常	异常	
				无临床意义	有临床意义
C 反应蛋白（CRP）		□1 mg/L　□2 其他：_____	□1	□2	□3

血沉（ESR）　□0 未查，原因____

采样日期：\|__\|__\|__\|__\|年\|__\|__\|月\|__\|__\|日　　正常值范围编号：\|__\|__\|__\|__\|

检查项目	测量结果		结果判定		
	实测值	单位	正常	异常	
				无临床意义	有临床意义
血沉（ESR）		□1 mm/h　□2 其他：_____	□1	□2	□3

TNF-α　□0 未查，原因____

采样日期：\|__\|__\|__\|__\|年\|__\|__\|月\|__\|__\|日　　正常值范围编号：\|__\|__\|__\|__\|

检查项目	测量结果		结果判定		
	实测值	单位	正常	异常	
				无临床意义	有临床意义
TNF-α		□1 pg/mL　□2 其他：_____	□1	□2	□3

IL-6　□0 未查，原因____

采样日期：\|__\|__\|__\|__\|年\|__\|__\|月\|__\|__\|日　　正常值范围编号：\|__\|__\|__\|__\|

检查项目	测量结果		结果判定		
	实测值	单位	正常	异常	
				无临床意义	有临床意义
IL-6		□1 pg/mL　□2 其他：_____	□1	□2	□3

**新型冠状病毒核酸检测**	□$_0$ 未查，原因________________________________
采样部位（可多选）	□$_0$ 上呼吸道　　□$_1$ 下呼吸道
上呼吸道	采样日期：|__|__|__|__|年|__|__|月|__|__|日<br>检查结果：□$_1$ 阴性 □$_2$ 阳性 □$_3$ 无法判断 □$_4$ 其他，请描述_________
下呼吸道	采样日期：|__|__|__|__|年|__|__|月|__|__|日<br>检查结果：□$_1$ 阴性 □$_2$ 阳性 □$_3$ 无法判断 □$_4$ 其他，请描述_________
结果判定	□$_1$ 阴性 □$_2$ 阳性 □$_3$ 无法判断 □$_4$ 其他，请描述__________________

**指脉氧饱和度**（不吸氧情况下）　□$_0$ 未查，原因________________________________
检查日期：|__|__|__|__|年|__|__|月|__|__|日
实测值：_______________%（静息状态）

**是否转成重型/危重型肺炎**　□$_0$ 未评价　　评价日期|__|__|__|__|年|__|__|月|__|__|日
是否转成重型/危重型肺炎：　□$_0$ 否　　□$_1$ 是，临床分型：□$_1$ 重型　□$_2$ 危重型
转成重型/危重型肺炎日期：|__|__|__|__|年|__|__|月|__|__|日

自上次访视以来，受试者如有不良事件发生，请填写“不良事件”表；如有合并药物治疗，请填写“既往/合并药物治疗”表；如有合并非药物治疗，请填写“既往/合并非药物治疗”表；如有计划外检查，请填写“计划外检查”表；如有 IMV/NIV、ECMO、HFNC、氧疗，请填写在“IMV/NIV、ECMO、HFNC、氧疗”表中。

本次访视日期：|__|__|__|__|年|__|__|月|__|__|日

**生命体征** ☐$_0$ 未查，原因________________			
检查日期	|__|__|__|__|年|__|__|月|__|__|日		
日最高体温	|__|__|.|__|℃	测量方式	☐$_1$ 腋下 ☐$_2$ 口腔 ☐$_3$ 肛门<br>☐$_4$ 耳朵 ☐$_5$ 未知 ☐$_6$ 其他，请描述______
呼吸频率	|__|__|次/分	脉搏	|__|__|__|次/分
血压	|__|__|__|/|__|__|__| mmHg（收缩压/舒张压）		

**有无呼吸道症状** ☐$_0$ 未查，原因________________
检查日期：|__|__|__|__|年|__|__|月|__|__|日
受试者有无呼吸道症状：☐$_0$ 无 ☐$_1$ 有，请描述：________________<br>________________

血常规 ☐$_0$ 未查，原因________________

采样日期：|__|__|__|__|年|__|__|月|__|__|日 正常值范围编号：|__|__|__|__|

检查项目	测量结果		结果判定			未查
	实测值	单位	正常	异常		
				无临床意义	有临床意义	
红细胞计数（RBC）		☐$_1$ 10^{12}/L ☐$_2$ 其他：____	☐$_1$	☐$_2$	☐$_3$	☐$_0$
白细胞计数（WBC）		☐$_1$ 10^9/L ☐$_2$ 其他：____	☐$_1$	☐$_2$	☐$_3$	☐$_0$
血红蛋白（Hb）		☐$_1$ g/L ☐$_2$ 其他：____	☐$_1$	☐$_2$	☐$_3$	☐$_0$
血小板计数（PLT）		☐$_1$ 10^9/L ☐$_2$ 其他：____	☐$_1$	☐$_2$	☐$_3$	☐$_0$
中性粒细胞计数（ANC）		☐$_1$ 10^9/L ☐$_2$ 其他：____	☐$_1$	☐$_2$	☐$_3$	☐$_0$
淋巴细胞计数（LY）		☐$_1$ 10^9/L ☐$_2$ 其他：____	☐$_1$	☐$_2$	☐$_3$	☐$_0$
单核细胞计数（MO）		☐$_1$ 10^9/L ☐$_2$ 其他：____	☐$_1$	☐$_2$	☐$_3$	☐$_0$
嗜酸性粒细胞计数（EO）		☐$_1$ 10^9/L ☐$_2$ 其他：____	☐$_1$	☐$_2$	☐$_3$	☐$_0$
嗜碱性粒细胞计数（BA）		☐$_1$ 10^9/L ☐$_2$ 其他：____	☐$_1$	☐$_2$	☐$_3$	☐$_0$

血生化 ☐$_0$ 未查，原因________________

采样日期|__|__|__|__|年|__|__|月|__|__|日 正常值范围编号：|__|__|__|__|

检查项目	测量结果		结果判定			未查
	实测值	单位	正常	异常		
				无临床意义	有临床意义	

谷丙转氨酶（ALT）		□1 U/L □2 其他：_____	□1	□2	□3	□0
谷草转氨酶（AST）		□1 U/L □2 其他：_____	□1	□2	□3	□0
总胆红素（TBIL）		□1 μmol/L □2 其他：_____	□1	□2	□3	□0
白蛋白（ALB）		□1 g/L □2 其他：_____	□1	□2	□3	□0
γ-谷氨酰转肽酶（GGT）		□1 U/L □2 其他：_____	□1	□2	□3	□0
乳酸脱氢酶（LDH）		□1 U/L □2 其他：_____	□1	□2	□3	□0
肌酸激酶（CK）		□1 U/L □2 其他：_____	□1	□2	□3	□0
肌酸激酶同工酶（CK-MB）		□1 U/L □2 其他：_____	□1	□2	□3	□0
血尿素氮（BUN）		□1 mmol/L □2 其他：_____	□1	□2	□3	□0
尿素（Urea）		□1 mmol/L □2 其他：_____	□1	□2	□3	□0
血肌酐（CR）		□1 μmol/L □2 其他：_____	□1	□2	□3	□0

尿常规 □0 未查，原因______________________________
□1 不适用

采样日期：|__|__|__|__|年|__|__|月|__|__|日 正常值范围编号：|__|__|__|__|

检查项目	测量结果		结果判定			未查
	实测值	单位	正常	异常		
				无临床意义	有临床意义	
尿蛋白		□1 不适用 □2 其他：_____	□1	□2	□3	□0
尿葡萄糖		□1 mmol/L □2 其他：_____	□1	□2	□3	□0
尿 PH 值		□1 不适用 □2 其他：_____	□1	□2	□3	□0
尿红细胞		□1 不适用 □2 其他：_____	□1	□2	□3	□0
尿白细胞		□1 不适用 □2 其他：_____	□1	□2	□3	□0
尿酮体		□1 不适用 □2 其他：_____	□1	□2	□3	□0

凝血功能 □0 未查，原因______________________________

采样日期：|__|__|__|__|年|__|__|月|__|__|日 正常值范围编号：|__|__|__|__|

检查项目	测量结果		结果判定			未查
	实测值	单位	正常	异常		
				无临床意义	有临床意义	
凝血酶原时间（PT）		□1 S □2 其他：_____	□1	□2	□3	□0
活化部分凝血活酶时间（APTT）		□1 S □2 其他：_____	□1	□2	□3	□0

凝血酶时间（TT）		□1 S □2 其他：_____	□1	□2	□3	□0
血浆纤维蛋白原（Fbg）		□1 g/L □2 其他：_____	□1	□2	□3	□0
国际标准化比率（INR）		□1 不适用 □2 其他：_____	□1	□2	□3	□0

C 反应蛋白（CRP） □0 未查，原因________________________________

采样日期：|__|__|__|__|年|__|__|月|__|__|日 正常值范围编号：|__|__|__|__|

检查项目	测量结果		结果判定		
	实测值	单位	正常	异常	
				无临床意义	有临床意义
C 反应蛋白（CRP）		□1 mg/L □2 其他：_____	□1	□2	□3

血沉（ESR） □0 未查，原因________________________________

采样日期：|__|__|__|__|年|__|__|月|__|__|日 正常值范围编号：|__|__|__|__|

检查项目	测量结果		结果判定		
	实测值	单位	正常	异常	
				无临床意义	有临床意义
血沉（ESR）		□1 mm/h □2 其他：_____	□1	□2	□3

TNF-α □0 未查，原因________________________________

采样日期：|__|__|__|__|年|__|__|月|__|__|日 正常值范围编号：|__|__|__|__|

检查项目	测量结果		结果判定		
	实测值	单位	正常	异常	
				无临床意义	有临床意义
TNF-α		□1 pg/mL □2 其他：_____	□1	□2	□3

IL-6 □0 未查，原因________________________________

采样日期：|__|__|__|__|年|__|__|月|__|__|日 正常值范围编号：|__|__|__|__|

检查项目	测量结果		结果判定		
	实测值	单位	正常	异常	
				无临床意义	有临床意义
IL-6		□1 pg/mL □2 其他：_____	□1	□2	□3

**新型冠状病毒核酸检测**	□0 未查，原因________________________________
采样部位（可多选）	□0 上呼吸道　　□1 下呼吸道
上呼吸道	采样日期：|__|__|__|__|年|__|__|月|__|__|日<br>检查结果：□1 阴性 □2 阳性 □3 无法判断 □4 其他，请描述________
下呼吸道	采样日期：|__|__|__|__|年|__|__|月|__|__|日<br>检查结果：□1 阴性 □2 阳性 □3 无法判断 □4 其他，请描述________
结果判定	□1 阴性 □2 阳性 □3 无法判断 □4 其他，请描述_______________

**指脉氧饱和度**（不吸氧情况下）　□0 未查，原因________________________________
检查日期：|__|__|__|__|年|__|__|月|__|__|日
实测值：_____________%（静息状态）

**是否转成重型/危重型肺炎**　　□0 未评价　　　评价日期|__|__|__|__|年|__|__|月|__|__|日
是否转成重型/危重型肺炎：　□0 否　　□1 是，临床分型：□1 重型　□2 危重型
转成重型/危重型肺炎日期：|__|__|__|__|年|__|__|月|__|__|日

自上次访视以来，受试者如有不良事件发生，请填写“不良事件”表；如有合并药物治疗，请填写“既往/合并药物治疗”表；如有合并非药物治疗，请填写“既往/合并非药物治疗”表；如有计划外检查，请填写“计划外检查”表；如有 IMV/NIV、ECMO、HFNC、氧疗，请填写在“IMV/NIV、ECMO、HFNC、氧疗”表中。

本次访视日期：|__|__|__|__|年|__|__|月|__|__|日

**生命体征** □$_0$未查，原因________			
检查日期	|__|__|__|__|年|__|__|月|__|__|日		
日最高体温	|__|__|.|__|℃	测量方式	□$_1$ 腋下 □$_2$ 口腔 □$_3$ 肛门<br>□$_4$ 耳朵 □$_5$ 未知 □$_6$ 其他，请描述______
呼吸频率	|__|__|次/分	脉搏	|__|__|__|次/分
血压	|__|__|__|/|__|__|__| mmHg（收缩压/舒张压）		

**有无呼吸道症状** □$_0$未查，原因________
检查日期：|__|__|__|__|年|__|__|月|__|__|日
受试者有无呼吸道症状：□$_0$ 无 □$_1$ 有，请描述：________

血常规 □$_0$未查，原因________

采样日期：|__|__|__|__|年|__|__|月|__|__|日　　正常值范围编号：|__|__|__|__|

检查项目	测量结果		结果判定			未查
	实测值	单位	正常	异常		
				无临床意义	有临床意义	
红细胞计数（RBC）		□$_1$ 10^{12}/L □$_2$其他：____	□$_1$	□$_2$	□$_3$	□$_0$
白细胞计数（WBC）		□$_1$ 10^9/L □$_2$其他：____	□$_1$	□$_2$	□$_3$	□$_0$
血红蛋白（Hb）		□$_1$ g/L □$_2$其他：____	□$_1$	□$_2$	□$_3$	□$_0$
血小板计数（PLT）		□$_1$ 10^9/L □$_2$其他：____	□$_1$	□$_2$	□$_3$	□$_0$
中性粒细胞计数（ANC）		□$_1$ 10^9/L □$_2$其他：____	□$_1$	□$_2$	□$_3$	□$_0$
淋巴细胞计数（LY）		□$_1$ 10^9/L □$_2$其他：____	□$_1$	□$_2$	□$_3$	□$_0$
单核细胞计数（MO）		□$_1$ 10^9/L □$_2$其他：____	□$_1$	□$_2$	□$_3$	□$_0$
嗜酸性粒细胞计数（EO）		□$_1$ 10^9/L □$_2$其他：____	□$_1$	□$_2$	□$_3$	□$_0$
嗜碱性粒细胞计数（BA）		□$_1$ 10^9/L □$_2$其他：____	□$_1$	□$_2$	□$_3$	□$_0$

血生化 □$_0$未查，原因________

采样日期|__|__|__|__|年|__|__|月|__|__|日　　正常值范围编号：|__|__|__|__|

检查项目	测量结果		结果判定			未查
	实测值	单位	正常	异常		
				无临床意义	有临床意义	

谷丙转氨酶（ALT）		□1 U/L　□2 其他：_____	□1	□2	□3	□0
谷草转氨酶（AST）		□1 U/L　□2 其他：_____	□1	□2	□3	□0
总胆红素（TBIL）		□1 μmol/L　□2 其他：_____	□1	□2	□3	□0
白蛋白（ALB）		□1 g/L　□2 其他：_____	□1	□2	□3	□0
γ-谷氨酰转肽酶（GGT）		□1 U/L　□2 其他：_____	□1	□2	□3	□0
乳酸脱氢酶（LDH）		□1 U/L　□2 其他：_____	□1	□2	□3	□0
肌酸激酶（CK）		□1 U/L　□2 其他：_____	□1	□2	□3	□0
肌酸激酶同工酶（CK-MB）		□1 U/L　□2 其他：_____	□1	□2	□3	□0
血尿素氮（BUN）		□1 mmol/L　□2 其他：_____	□1	□2	□3	□0
尿素（Urea）		□1 mmol/L　□2 其他：_____	□1	□2	□3	□0
血肌酐（CR）		□1 μmol/L　□2 其他：_____	□1	□2	□3	□0

尿常规　□0 未查，原因______________________________
□1 不适用

采样日期：|__|__|__|__|年|__|__|月|__|__|日　正常值范围编号：|__|__|__|__|

检查项目	测量结果		结果判定			未查
	实测值	单位	正常	异常		
				无临床意义	有临床意义	
尿蛋白		□1 不适用　□2 其他：_____	□1	□2	□3	□0
尿葡萄糖		□1 mmol/L　□2 其他：_____	□1	□2	□3	□0
尿 PH 值		□1 不适用　□2 其他：_____	□1	□2	□3	□0
尿红细胞		□1 不适用　□2 其他：_____	□1	□2	□3	□0
尿白细胞		□1 不适用　□2 其他：_____	□1	□2	□3	□0
尿酮体		□1 不适用　□2 其他：_____	□1	□2	□3	□0

凝血功能　□0 未查，原因______________________________

采样日期：|__|__|__|__|年|__|__|月|__|__|日　正常值范围编号：|__|__|__|__|

检查项目	测量结果		结果判定			未查
	实测值	单位	正常	异常		
				无临床意义	有临床意义	
凝血酶原时间（PT）		□1 S　□2 其他：_____	□1	□2	□3	□0
活化部分凝血活酶时间（APTT）		□1 S　□2 其他：_____	□1	□2	□3	□0

凝血酶时间（TT）		□1 S　□2 其他：____	□1	□2	□3	□0
血浆纤维蛋白原（Fbg）		□1 g/L　□2 其他：____	□1	□2	□3	□0
国际标准化比率（INR）		□1 不适用　□2 其他：____	□1	□2	□3	□0

C 反应蛋白（CRP）　□0 未查，原因____________

采样日期：|__|__|__|__|年|__|__|月|__|__|日　　正常值范围编号：|__|__|__|__|

检查项目	测量结果		结果判定		
	实测值	单位	正常	异常	
				无临床意义	有临床意义
C 反应蛋白（CRP）		□1 mg/L　□2 其他：____	□1	□2	□3

血沉（ESR）　□0 未查，原因____________

采样日期：|__|__|__|__|年|__|__|月|__|__|日　　正常值范围编号：|__|__|__|__|

检查项目	测量结果		结果判定		
	实测值	单位	正常	异常	
				无临床意义	有临床意义
血沉（ESR）		□1 mm/h　□2 其他：____	□1	□2	□3

TNF-α　□0 未查，原因____________

采样日期：|__|__|__|__|年|__|__|月|__|__|日　　正常值范围编号：|__|__|__|__|

检查项目	测量结果		结果判定		
	实测值	单位	正常	异常	
				无临床意义	有临床意义
TNF-α		□1 pg/mL　□2 其他：____	□1	□2	□3

IL-6　□0 未查，原因____________

采样日期：|__|__|__|__|年|__|__|月|__|__|日　　正常值范围编号：|__|__|__|__|

检查项目	测量结果		结果判定		
	实测值	单位	正常	异常	
				无临床意义	有临床意义
IL-6		□1 pg/mL　□2 其他：____	□1	□2	□3

**新型冠状病毒核酸检测**	□$_0$ 未查，原因________________________________
采样部位（可多选）	□$_0$ 上呼吸道　　□$_1$ 下呼吸道
上呼吸道	采样日期：|__|__|__|__|年|__|__|月|__|__|日<br>检查结果：□$_1$ 阴性 □$_2$ 阳性 □$_3$ 无法判断 □$_4$ 其他，请描述_________
下呼吸道	采样日期：|__|__|__|__|年|__|__|月|__|__|日<br>检查结果：□$_1$ 阴性 □$_2$ 阳性 □$_3$ 无法判断 □$_4$ 其他，请描述_________
结果判定	□$_1$ 阴性 □$_2$ 阳性 □$_3$ 无法判断 □$_4$ 其他，请描述__________________

**指脉氧饱和度**（不吸氧情况下）　□$_0$ 未查，原因________________________________
检查日期：|__|__|__|__|年|__|__|月|__|__|日
实测值：_______________%（静息状态）

**是否转成重型/危重型肺炎**　□$_0$ 未评价　评价日期|__|__|__|__|年|__|__|月|__|__|日
是否转成重型/危重型肺炎：　□$_0$ 否　　□$_1$ 是，临床分型：□$_1$ 重型　□$_2$ 危重型
转成重型/危重型肺炎日期：|__|__|__|__|年|__|__|月|__|__|日

自上次访视以来，受试者如有不良事件发生，请填写“不良事件”表；如有合并药物治疗，请填写“既往/合并药物治疗”表；如有合并非药物治疗，请填写“既往/合并非药物治疗”表；如有计划外检查，请填写“计划外检查”表；如有 IMV/NIV、ECMO、HFNC、氧疗，请填写在“IMV/NIV、ECMO、HFNC、氧疗”表中。

本次访视日期：|__|__|__|__|年|__|__|月|__|__|日

**生命体征**	□0 未查，原因________________________________		
检查日期	|__|__|__|__|年|__|__|月|__|__|日		
日最高体温	|__|__|.|__|℃	测量方式	□1 腋下 □2 口腔 □3 肛门<br>□4 耳朵 □5 未知 □6 其他，请描述______
呼吸频率	|__|__|次/分	脉搏	|__|__|__|次/分
血压	|__|__|__|/|__|__|__| mmHg（收缩压/舒张压）		

**体格检查**	□0 未查，原因________________________________				
检查日期：|__|__|__|__|年|__|__|月|__|__|日					
**检查部位**	**检查结果**<br>**1=正常**<br>**2=异常无临床意义**<br>**3=异常有临床意义**<br>**0=未查**				**异常请描述**
整体外观	□1	□2	□3	□0	
头	□1	□2	□3	□0	
眼	□1	□2	□3	□0	
耳	□1	□2	□3	□0	
鼻	□1	□2	□3	□0	
喉	□1	□2	□3	□0	
颈部及甲状腺	□1	□2	□3	□0	
皮肤	□1	□2	□3	□0	
心血管系统	□1	□2	□3	□0	
胸部及肺	□1	□2	□3	□0	
腹部	□1	□2	□3	□0	
神经系统	□1	□2	□3	□0	
骨骼肌系统	□1	□2	□3	□0	
淋巴系统	□1	□2	□3	□0	

有无呼吸道症状 □0 未查，原因________________
检查日期：|__|__|__|__|年|__|__|月|__|__|日
受试者有无呼吸道症状：□0 无 □1 有，请描述：________________

血常规 □0 未查，原因________________						
采样日期：|__|__|__|__|年|__|__|月|__|__|日			正常值范围编号：|__|__|__|__|			
检查项目	测量结果		结果判定			未查
	实测值	单位	正常	异常		
				无临床意义	有临床意义	
红细胞计数（RBC）		□1 10^{12}/L □2 其他：____	□1	□2	□3	□0
白细胞计数（WBC）		□1 10^{9}/L □2 其他：____	□1	□2	□3	□0
血红蛋白（Hb）		□1 g/L □2 其他：____	□1	□2	□3	□0
血小板计数（PLT）		□1 10^{9}/L □2 其他：____	□1	□2	□3	□0
中性粒细胞计数（ANC）		□1 10^{9}/L □2 其他：____	□1	□2	□3	□0
淋巴细胞计数（LY）		□1 10^{9}/L □2 其他：____	□1	□2	□3	□0
单核细胞计数（MO）		□1 10^{9}/L □2 其他：____	□1	□2	□3	□0
嗜酸性粒细胞计数（EO）		□1 10^{9}/L □2 其他：____	□1	□2	□3	□0
嗜碱性粒细胞计数（BA）		□1 10^{9}/L □2 其他：____	□1	□2	□3	□0

血生化 □0 未查，原因________________						
采样日期|__|__|__|__|年|__|__|月|__|__|日			正常值范围编号：|__|__|__|__|			
检查项目	测量结果		结果判定			未查
	实测值	单位	正常	异常		
				无临床意义	有临床意义	
谷丙转氨酶（ALT）		□1 U/L □2 其他：____	□1	□2	□3	□0
谷草转氨酶（AST）		□1 U/L □2 其他：____	□1	□2	□3	□0
总胆红素（TBIL）		□1 μmol/L □2 其他：____	□1	□2	□3	□0
白蛋白（ALB）		□1 g/L □2 其他：____	□1	□2	□3	□0
γ-谷氨酰转肽酶（GGT）		□1 U/L □2 其他：____	□1	□2	□3	□0
乳酸脱氢酶（LDH）		□1 U/L □2 其他：____	□1	□2	□3	□0
肌酸激酶（CK）		□1 U/L □2 其他：____	□1	□2	□3	□0
肌酸激酶同工酶（CK-MB）		□1 U/L □2 其他：____	□1	□2	□3	□0
血尿素氮（BUN）		□1 mmol/L □2 其他：____	□1	□2	□3	□0
尿素（Urea）		□1 mmol/L □2 其他：____	□1	□2	□3	□0

血肌酐（CR）		□1 μmol/L □2 其他：____	□1	□2	□3	□0

尿常规 □0 未查，原因________________________

采样日期：|__|__|__|__|年|__|__|月|__|__|日　　正常值范围编号：|__|__|__|__|

检查项目	测量结果		结果判定			未查
	实测值	单位	正常	异常		
				无临床意义	有临床意义	
尿蛋白		□1 不适用 □2 其他：____	□1	□2	□3	□0
尿葡萄糖		□1 mmol/L □2 其他：____	□1	□2	□3	□0
尿 PH 值		□1 不适用 □2 其他：____	□1	□2	□3	□0
尿红细胞		□1 不适用 □2 其他：____	□1	□2	□3	□0
尿白细胞		□1 不适用 □2 其他：____	□1	□2	□3	□0
尿酮体		□1 不适用 □2 其他：____	□1	□2	□3	□0

凝血功能 □0 未查，原因________________________

采样日期：|__|__|__|__|年|__|__|月|__|__|日　　正常值范围编号：|__|__|__|__|

检查项目	测量结果		结果判定			未查
	实测值	单位	正常	异常		
				无临床意义	有临床意义	
凝血酶原时间（PT）		□1 S □2 其他：____	□1	□2	□3	□0
活化部分凝血活酶时间（APTT）		□1 S □2 其他：____	□1	□2	□3	□0
凝血酶时间（TT）		□1 S □2 其他：____	□1	□2	□3	□0
血浆纤维蛋白原（Fbg）		□1 g/L □2 其他：____	□1	□2	□3	□0
国际标准化比率（INR）		□1 不适用 □2 其他：____	□1	□2	□3	□0

C 反应蛋白（CRP） □0 未查，原因________________________

采样日期：|__|__|__|__|年|__|__|月|__|__|日　　正常值范围编号：|__|__|__|__|

检查项目	测量结果		结果判定		
	实测值	单位	正常	异常	
				无临床意义	有临床意义
C 反应蛋白（CRP）		□1 mg/L □2 其他：____	□1	□2	□3

血沉（ESR） □0 未查，原因________________________

采样日期：|__|__|__|__|年|__|__|月|__|__|日　　正常值范围编号：|__|__|__|__|

检查项目	测量结果		结果判定		
				异常	
	实测值	单位	正常	无临床意义	有临床意义
血沉（ESR）		□1 mm/h □2其他：_____	□1	□2	□3

TNF-α □0 未查，原因________________________________

采样日期：|__|__|__|__|年|__|__|月|__|__|日 正常值范围编号：|__|__|__|__|

检查项目	测量结果		结果判定		
				异常	
	实测值	单位	正常	无临床意义	有临床意义
TNF-α		□1 pg/mL □2其他：_____	□1	□2	□3

IL-6 □0 未查，原因________________________________

采样日期：|__|__|__|__|年|__|__|月|__|__|日 正常值范围编号：|__|__|__|__|

检查项目	测量结果		结果判定		
				异常	
	实测值	单位	正常	无临床意义	有临床意义
IL-6		□1 pg/mL □2其他：_____	□1	□2	□3

尿或血清 HCG（仅适用于育龄女性） □0 未查，原因________________________
□1 不适用（男性或不具有生育能力的女性）

采样日期：|__|__|__|__|年|__|__|月|__|__|日 正常值范围编号：|__|__|__|__|

采样类别：□1 血液 □2 尿液

检查结果：□1 阴性 □2 阳性 □3 其他：____________________

单位： □1 不适用 □2 mIU/mL □3 其他：____________________

12 导联心电图 □0 未查，原因________________________

检查日期：|__|__|__|__|年|__|__|月|__|__|日

检查结果：□1 正常 □2 异常无临床意义 □3 异常有临床意义

若"异常"，请描述：____

____

胸部 CT □0 未查，原因________________________
□1 不适用

检查日期：|__|__|__|__|年|__|__|月|__|__|日

检查结果：□1 正常 □2 异常无临床意义 □3 异常有临床意义

若异常，请描述：____	
是否胸部 CT 病灶吸收较基线≥50%？	□$_0$ 否 □$_1$ 是 □$_2$ 无法判断 □$_3$ 不适用 □$_4$ 其他，请描述：__________

**新型冠状病毒核酸检测**	□$_0$ 未查，原因__________
采样部位（可多选）	□$_0$ 上呼吸道 □$_1$ 下呼吸道
上呼吸道	采样日期：|__|__|__|__|年|__|__|月|__|__|日<br>检查结果：□$_1$ 阴性 □$_2$ 阳性 □$_3$ 无法判断 □$_4$ 其他，请描述__________
下呼吸道	采样日期：|__|__|__|__|年|__|__|月|__|__|日<br>检查结果：□$_1$ 阴性 □$_2$ 阳性 □$_3$ 无法判断 □$_4$ 其他，请描述__________
结果判定	□$_1$ 阴性 □$_2$ 阳性 □$_3$ 无法判断 □$_4$ 其他，请描述__________

**指脉氧饱和度**（不吸氧情况下） □$_0$ 未查，原因__________
检查日期：|__|__|__|__|年|__|__|月|__|__|日
实测值：__________%（静息状态）

**是否转成重型/危重型肺炎**	□$_0$ 未评价	评价日期|__|__|__|__|年|__|__|月|__|__|日
是否转成重型/危重型肺炎：	□$_0$ 否	□$_1$ 是，临床分型： □$_1$ 重型 □$_2$ 危重型
转成重型/危重型肺炎日期：|__|__|__|__|年|__|__|月|__|__|日		

自上次访视以来，受试者如有不良事件发生，请填写“不良事件”表；如有合并药物治疗，请填写“既往/合并药物治疗”表；如有合并非药物治疗，请填写“既往/合并非药物治疗”表；如有计划外检查，请填写“计划外检查”表；如有 IMV/NIV、ECMO、HFNC、HFNC、氧疗，请填写在“IMV/NIV、ECMO、HFNC、氧疗”表中。

治疗结束
受试者是否按研究方案完成了所有治疗？ □0 否 □1 是
治疗结束日期：|__|__|__|__|年|__|__|月|__|__|日
如提前退出治疗，请选择最重要的原因（单选）：<br>□1 研究期间出现疾病恶化，经研究者综合评估后需要改变治疗<br>□2 药物不良事件导致病情加重的出现<br>□3 患者不愿意继续使用研究药物<br>□4 妊娠<br>□5 受试者失访<br>□6 受试者死亡<br>□7 受试者撤回知情同意<br>□8 研究整体中止<br>□9 其他，请描述：______________________________<br>______________________________<br><br>备注（请记录其他需要补充说明的事由）：<br>______________________________<br>______________________________<br>______________________________

**访视类型：**□1 电话随访	□2 医院随访
**本次访视日期：**|__|__|__|__|年|__|__|月|__|__|日	

如受试者已出院，以下内容可不进行，如未进行请留空；如受试者未出院，请进行以下检查。

**生命体征** □0 未查，原因________			
检查日期	|__|__|__|__|年|__|__|月|__|__|日		
日最高体温	|__|__|.|__|℃	测量方式	□1 腋下 □2 口腔 □3 肛门<br>□4 耳朵 □5 未知 □6 其他，请描述______
呼吸频率	|__|__|次/分	脉搏	|__|__|__|次/分
血压	|__|__|__|/|__|__|__| mmHg（收缩压/舒张压）		

**体格检查** □0 未查，原因________					
检查日期：|__|__|__|__|年|__|__|月|__|__|日					
**检查部位**	**检查结果**<br>**1=正常**<br>**2=异常无临床意义**<br>**3=异常有临床意义**<br>**0=未查**				**异常请描述**
整体外观	□1	□2	□3	□0	
头	□1	□2	□3	□0	
眼	□1	□2	□3	□0	
耳	□1	□2	□3	□0	
鼻	□1	□2	□3	□0	
喉	□1	□2	□3	□0	
颈部及甲状腺	□1	□2	□3	□0	
皮肤	□1	□2	□3	□0	
心血管系统	□1	□2	□3	□0	
胸部及肺	□1	□2	□3	□0	
腹部	□1	□2	□3	□0	
神经系统	□1	□2	□3	□0	

骨骼肌系统	□1	□2	□3	□0	
淋巴系统	□1	□2	□3	□0	

有无呼吸道症状 □0 未查，原因________________

检查日期：|__|__|__|__|年|__|__|月|__|__|日

受试者有无呼吸道症状：□0 无 □1 有，请描述：________________

____

血常规 □0 未查，原因________________

采样日期：|__|__|__|__|年|__|__|月|__|__|日 正常值范围编号：|__|__|__|__|

检查项目	测量结果		结果判定			未查
	实测值	单位	正常	异常		
				无临床意义	有临床意义	
红细胞计数（RBC）		□1 10^{12}/L □2 其他：____	□1	□2	□3	□0
白细胞计数（WBC）		□1 10^9/L □2 其他：____	□1	□2	□3	□0
血红蛋白（Hb）		□1 g/L □2 其他：____	□1	□2	□3	□0
血小板计数（PLT）		□1 10^9/L □2 其他：____	□1	□2	□3	□0
中性粒细胞计数（ANC）		□1 10^9/L □2 其他：____	□1	□2	□3	□0
淋巴细胞计数（LY）		□1 10^9/L □2 其他：____	□1	□2	□3	□0
单核细胞计数（MO）		□1 10^9/L □2 其他：____	□1	□2	□3	□0
嗜酸性粒细胞计数（EO）		□1 10^9/L □2 其他：____	□1	□2	□3	□0
嗜碱性粒细胞计数（BA）		□1 10^9/L □2 其他：____	□1	□2	□3	□0

血生化 □0 未查，原因________________

采样日期|__|__|__|__|年|__|__|月|__|__|日 正常值范围编号：|__|__|__|__|

检查项目	测量结果		结果判定			未查
	实测值	单位	正常	异常		
				无临床意义	有临床意义	
谷丙转氨酶（ALT）		□1 U/L □2 其他：____	□1	□2	□3	□0
谷草转氨酶（AST）		□1 U/L □2 其他：____	□1	□2	□3	□0
总胆红素（TBIL）		□1 μmol/L □2 其他：____	□1	□2	□3	□0
白蛋白（ALB）		□1 g/L □2 其他：____	□1	□2	□3	□0
γ-谷氨酰转肽酶（GGT）		□1 U/L □2 其他：____	□1	□2	□3	□0
乳酸脱氢酶（LDH）		□1 U/L □2 其他：____	□1	□2	□3	□0

肌酸激酶（CK）		□1 U/L □2 其他：_____	□1	□2	□3	□0
肌酸激酶同工酶（CK-MB）		□1 U/L □2 其他：_____	□1	□2	□3	□0
血尿素氮（BUN）		□1 mmol/L □2 其他：_____	□1	□2	□3	□0
尿素（Urea）		□1 mmol/L □2 其他：_____	□1	□2	□3	□0
血肌酐（CR）		□1 μmol/L □2 其他：_____	□1	□2	□3	□0

尿常规 □0 未查，原因____

采样日期：|__|__|__|__|年|__|__|月|__|__|日　　正常值范围编号：|__|__|__|__|

检查项目	测量结果		结果判定			未查
	实测值	单位	正常	异常		
				无临床意义	有临床意义	
尿蛋白		□1 不适用 □2 其他：_____	□1	□2	□3	□0
尿葡萄糖		□1 mmol/L □2 其他：_____	□1	□2	□3	□0
尿 PH 值		□1 不适用 □2 其他：_____	□1	□2	□3	□0
尿红细胞		□1 不适用 □2 其他：_____	□1	□2	□3	□0
尿白细胞		□1 不适用 □2 其他：_____	□1	□2	□3	□0
尿酮体		□1 不适用 □2 其他：_____	□1	□2	□3	□0

凝血功能 □0 未查，原因____

采样日期：|__|__|__|__|年|__|__|月|__|__|日　　正常值范围编号：|__|__|__|__|

检查项目	测量结果		结果判定			未查
	实测值	单位	正常	异常		
				无临床意义	有临床意义	
凝血酶原时间（PT）		□1 S □2 其他：_____	□1	□2	□3	□0
活化部分凝血活酶时间（APTT）		□1 S □2 其他：_____	□1	□2	□3	□0
凝血酶时间（TT）		□1 S □2 其他：_____	□1	□2	□3	□0
血浆纤维蛋白原（Fbg）		□1 g/L □2 其他：_____	□1	□2	□3	□0
国际标准化比率（INR）		□1 不适用 □2 其他：_____	□1	□2	□3	□0

C 反应蛋白（CRP） □0 未查，原因____

采样日期：|__|__|__|__|年|__|__|月|__|__|日　　正常值范围编号：|__|__|__|__|

检查项目	测量结果		结果判定	
	实测值	单位	正常	异常

				无临床意义	有临床意义
C 反应蛋白（CRP）		□1 mg/L　□2 其他：_____	□1	□2	□3

血沉（ESR）　□0 未查，原因________________________________

采样日期：|__|__|__|__|年|__|__|月|__|__|日　　正常值范围编号：|__|__|__|__|

检查项目	测量结果		结果判定		
	实测值	单位	正常	异常	
				无临床意义	有临床意义
血沉（ESR）		□1 mm/h　□2 其他：_____	□1	□2	□3

TNF-α　□0 未查，原因________________________________

采样日期：|__|__|__|__|年|__|__|月|__|__|日　　正常值范围编号：|__|__|__|__|

检查项目	测量结果		结果判定		
	实测值	单位	正常	异常	
				无临床意义	有临床意义
TNF-α		□1 pg/mL　□2 其他：_____	□1	□2	□3

IL-6　□0 未查，原因________________________________

采样日期：|__|__|__|__|年|__|__|月|__|__|日　　正常值范围编号：|__|__|__|__|

检查项目	测量结果		结果判定		
	实测值	单位	正常	异常	
				无临床意义	有临床意义
IL-6		□1 pg/mL　□2 其他：_____	□1	□2	□3

尿或血清 HCG（仅适用于育龄女性）　□0 未查，原因________________________
□1 不适用（男性或不具有生育能力的女性）
□2 不适用（方案不涉及）

采样日期：|__|__|__|__|年|__|__|月|__|__|日　　正常值范围编号：|__|__|__|__|

采样类别：□1 血液　□2 尿液

检查结果：□1 阴性　□2 阳性　□3 其他：____________________

单位：□1 不适用　□2 mIU/mL　□3 其他：____________________

12 导联心电图　□0 未查，原因________________________________

检查日期：|__|__|__|__|年|__|__|月|__|__|日

检查结果：□1 正常　□2 异常无临床意义　□3 异常有临床意义

若“异常”，请描述：____

____

**胸部 CT** ☐0 未查，原因______________________ ☐1 不适用
检查日期：|__|__|__|__|年|__|__|月|__|__|日
检查结果：☐1 正常 ☐2 异常无临床意义 ☐3 异常有临床意义
若异常，请描述：______________________
是否胸部 CT 病灶吸收较基线≥50%？ ☐0 否 ☐1 是 ☐2 无法判断 ☐3 不适用 ☐4 其他，请描述：__________

**新型冠状病毒核酸检测**	☐0 未查，原因______________________
采样部位（可多选）	☐0 上呼吸道 ☐1 下呼吸道
上呼吸道	采样日期：|__|__|__|__|年|__|__|月|__|__|日<br>检查结果：☐1 阴性 ☐2 阳性 ☐3 无法判断 ☐4 其他，请描述__________
下呼吸道	采样日期：|__|__|__|__|年|__|__|月|__|__|日<br>检查结果：☐1 阴性 ☐2 阳性 ☐3 无法判断 ☐4 其他，请描述__________
结果判定	☐1 阴性 ☐2 阳性 ☐3 无法判断 ☐4 其他，请描述__________

**指脉氧饱和度**（不吸氧情况下） ☐0 未查，原因______________________
检查日期：|__|__|__|__|年|__|__|月|__|__|日
实测值：____________%（静息状态）

**是否转成重型/危重型肺炎** ☐0 未评价 评价日期|__|__|__|__|年|__|__|月|__|__|日
是否转成重型/危重型肺炎： ☐0 否 ☐1 是，临床分型： ☐1 重型 ☐2 危重型
转成重型/危重型肺炎日期：|__|__|__|__|年|__|__|月|__|__|日

自上次访视以来，受试者如有不良事件发生，请填写“不良事件”表；如有合并药物治疗，请填写“既往/合并药物治疗”表；如有合并非药物治疗，请填写“既往/合并非药物治疗”表；如有计划外检查，请填写“计划外检查”表；如有 IMV/NIV、ECMO、HFNC、HFNC、氧疗，请填写在“IMV/NIV、ECMO、HFNC、氧疗”表中。

**访视类型：**□1 电话随访	□2 医院随访
**本次访视日期：**|__|__|__|__|年|__|__|月|__|__|日	

如受试者已出院，以下内容可不进行，如未进行请留空；如受试者未出院，请进行以下检查。

**生命体征**	□0 未查，原因________________		
检查日期	|__|__|__|__|年|__|__|月|__|__|日		
日最高体温	|__|__|.|__|℃	测量方式	□1 腋下 □2 口腔 □3 肛门<br>□4 耳朵 □5 未知 □6 其他，请描述______
呼吸频率	|__|__|次/分	脉搏	|__|__|__|次/分
血压	|__|__|__|/|__|__|__| mmHg（收缩压/舒张压）		

**体格检查**	□0 未查，原因________________				
检查日期：|__|__|__|__|年|__|__|月|__|__|日					
**检查部位**	**检查结果**<br>**1=正常**<br>**2=异常无临床意义**<br>**3=异常有临床意义**<br>**0=未查**				**异常请描述**
整体外观	□1	□2	□3	□0	
头	□1	□2	□3	□0	
眼	□1	□2	□3	□0	
耳	□1	□2	□3	□0	
鼻	□1	□2	□3	□0	
喉	□1	□2	□3	□0	
颈部及甲状腺	□1	□2	□3	□0	
皮肤	□1	□2	□3	□0	
心血管系统	□1	□2	□3	□0	
胸部及肺	□1	□2	□3	□0	
腹部	□1	□2	□3	□0	
神经系统	□1	□2	□3	□0	

骨骼肌系统	□1	□2	□3	□0	
淋巴系统	□1	□2	□3	□0	

有无呼吸道症状 □0 未查，原因______________________________

检查日期：|__|__|__|__|年|__|__|月|__|__|日

受试者有无呼吸道症状：□0 无 □1 有，请描述：______________________________

____

血常规 □0 未查，原因______________________________

采样日期：|__|__|__|__|年|__|__|月|__|__|日 正常值范围编号：|__|__|__|__|

检查项目	测量结果		结果判定			未查
	实测值	单位	正常	异常		
				无临床意义	有临床意义	
红细胞计数（RBC）		□1 10^{12}/L □2 其他：____	□1	□2	□3	□0
白细胞计数（WBC）		□1 10^{9}/L □2 其他：____	□1	□2	□3	□0
血红蛋白（Hb）		□1 g/L □2 其他：____	□1	□2	□3	□0
血小板计数（PLT）		□1 10^{9}/L □2 其他：____	□1	□2	□3	□0
中性粒细胞计数（ANC）		□1 10^{9}/L □2 其他：____	□1	□2	□3	□0
淋巴细胞计数（LY）		□1 10^{9}/L □2 其他：____	□1	□2	□3	□0
单核细胞计数（MO）		□1 10^{9}/L □2 其他：____	□1	□2	□3	□0
嗜酸性粒细胞计数（EO）		□1 10^{9}/L □2 其他：____	□1	□2	□3	□0
嗜碱性粒细胞计数（BA）		□1 10^{9}/L □2 其他：____	□1	□2	□3	□0

血生化 □0 未查，原因______________________________

采样日期|__|__|__|__|年|__|__|月|__|__|日 正常值范围编号：|__|__|__|__|

检查项目	测量结果		结果判定			未查
	实测值	单位	正常	异常		
				无临床意义	有临床意义	
谷丙转氨酶（ALT）		□1 U/L □2 其他：____	□1	□2	□3	□0
谷草转氨酶（AST）		□1 U/L □2 其他：____	□1	□2	□3	□0
总胆红素（TBIL）		□1 μmol/L □2 其他：____	□1	□2	□3	□0
白蛋白（ALB）		□1 g/L □2 其他：____	□1	□2	□3	□0
γ-谷氨酰转肽酶（GGT）		□1 U/L □2 其他：____	□1	□2	□3	□0
乳酸脱氢酶（LDH）		□1 U/L □2 其他：____	□1	□2	□3	□0

肌酸激酶（CK）		□$_1$ U/L　□$_2$其他：_____	□$_1$	□$_2$	□$_3$	□$_0$
肌酸激酶同工酶（CK-MB）		□$_1$ U/L　□$_2$其他：_____	□$_1$	□$_2$	□$_3$	□$_0$
血尿素氮（BUN）		□$_1$ mmol/L　□$_2$其他：_____	□$_1$	□$_2$	□$_3$	□$_0$
尿素（Urea）		□$_1$ mmol/L　□$_2$其他：_____	□$_1$	□$_2$	□$_3$	□$_0$
血肌酐（CR）		□$_1$ μmol/L　□$_2$其他：_____	□$_1$	□$_2$	□$_3$	□$_0$

尿常规　□$_0$未查，原因________________________________

采样日期：|__|__|__|__|年|__|__|月|__|__|日　　正常值范围编号：|__|__|__|__|

检查项目	测量结果		结果判定			未查
	实测值	单位	正常	异常		
				无临床意义	有临床意义	
尿蛋白		□$_1$不适用　□$_2$其他：_____	□$_1$	□$_2$	□$_3$	□$_0$
尿葡萄糖		□$_1$ mmol/L　□$_2$其他：_____	□$_1$	□$_2$	□$_3$	□$_0$
尿 PH 值		□$_1$不适用　□$_2$其他：_____	□$_1$	□$_2$	□$_3$	□$_0$
尿红细胞		□$_1$不适用　□$_2$其他：_____	□$_1$	□$_2$	□$_3$	□$_0$
尿白细胞		□$_1$不适用　□$_2$其他：_____	□$_1$	□$_2$	□$_3$	□$_0$
尿酮体		□$_1$不适用　□$_2$其他：_____	□$_1$	□$_2$	□$_3$	□$_0$

凝血功能　□$_0$未查，原因________________________________

采样日期：|__|__|__|__|年|__|__|月|__|__|日　　正常值范围编号：|__|__|__|__|

检查项目	测量结果		结果判定			未查
	实测值	单位	正常	异常		
				无临床意义	有临床意义	
凝血酶原时间（PT）		□$_1$ S　□$_2$其他：_____	□$_1$	□$_2$	□$_3$	□$_0$
活化部分凝血活酶时间（APTT）		□$_1$ S　□$_2$其他：_____	□$_1$	□$_2$	□$_3$	□$_0$
凝血酶时间（TT）		□$_1$ S　□$_2$其他：_____	□$_1$	□$_2$	□$_3$	□$_0$
血浆纤维蛋白原（Fbg）		□$_1$ g/L　□$_2$其他：_____	□$_1$	□$_2$	□$_3$	□$_0$
国际标准化比率（INR）		□$_1$不适用　□$_2$其他：_____	□$_1$	□$_2$	□$_3$	□$_0$

C 反应蛋白（CRP）　□$_0$未查，原因________________________________

采样日期：|__|__|__|__|年|__|__|月|__|__|日　　正常值范围编号：|__|__|__|__|

检查项目	测量结果		结果判定	
	实测值	单位	正常	异常

				无临床意义	有临床意义
C 反应蛋白（CRP）		□1 mg/L □2 其他：_____	□1	□2	□3

血沉（ESR） □0 未查，原因____________________

采样日期：|__|__|__|__|年|__|__|月|__|__|日 正常值范围编号：|__|__|__|__|

检查项目	测量结果		结果判定		
	实测值	单位	正常	异常	
				无临床意义	有临床意义
血沉（ESR）		□1 mm/h □2 其他：_____	□1	□2	□3

TNF-α □0 未查，原因____________________

采样日期：|__|__|__|__|年|__|__|月|__|__|日 正常值范围编号：|__|__|__|__|

检查项目	测量结果		结果判定		
	实测值	单位	正常	异常	
				无临床意义	有临床意义
TNF-α		□1 pg/mL □2 其他：_____	□1	□2	□3

IL-6 □0 未查，原因____________________

采样日期：|__|__|__|__|年|__|__|月|__|__|日 正常值范围编号：|__|__|__|__|

检查项目	测量结果		结果判定		
	实测值	单位	正常	异常	
				无临床意义	有临床意义
IL-6		□1 pg/mL □2 其他：_____	□1	□2	□3

12 导联心电图 □0 未查，原因____________________

检查日期：|__|__|__|__|年|__|__|月|__|__|日

检查结果：□1 正常 □2 异常无临床意义 □3 异常有临床意义

若“异常”，请描述：____________________

____

胸部 CT □0 未查，原因____________________
□1 不适用

检查日期：|__|__|__|__|年|__|__|月|__|__|日

检查结果：□1 正常 □2 异常无临床意义 □3 异常有临床意义

若异常，请描述：____
是否胸部 CT 病灶吸收较基线≥50%？ □0 否 □1 是 □2 无法判断 □3 不适用 □4 其他，请描述：______________

**新型冠状病毒核酸检测**	□0 未查，原因______________________
采样部位（可多选）	□0 上呼吸道 □1 下呼吸道
上呼吸道	采样日期：|__|__|__|__|年|__|__|月|__|__|日<br>检查结果：□1 阴性 □2 阳性 □3 无法判断 □4 其他，请描述_________
下呼吸道	采样日期：|__|__|__|__|年|__|__|月|__|__|日<br>检查结果：□1 阴性 □2 阳性 □3 无法判断 □4 其他，请描述_________
结果判定	□1 阴性 □2 阳性 □3 无法判断 □4 其他，请描述__________

**指脉氧饱和度**（不吸氧情况下） □0 未查，原因______________________
检查日期：|__|__|__|__|年|__|__|月|__|__|日
实测值：______________%（静息状态）

**是否转成重型/危重型肺炎** □0 未评价 评价日期|__|__|__|__|年|__|__|月|__|__|日
是否转成重型/危重型肺炎： □0 否 □1 是，临床分型： □1 重型 □2 危重型
转成重型/危重型肺炎日期：|__|__|__|__|年|__|__|月|__|__|日

自上次访视以来，受试者如有不良事件发生，请填写“不良事件”表；如有合并药物治疗，请填写“既往/合并药物治疗”表；如有合并非药物治疗，请填写“既往/合并非药物治疗”表；如有计划外检查，请填写“计划外检查”表。；如有 IMV/NIV、ECMO、HFNC、氧疗，请填写在“IMV/NIV、ECMO、HFNC、氧疗”表中。

受试者是否使用试验药物？ □0 否 □1 是 □2 不适用（对照组）

试验药物使用情况

服药天数	是否用药	用药日期	计划每日给药总剂量（mg）	实际每日给药总剂量（mg）	给药频率（1）	给药情况（2）	如剂量调整或暂停给药，请说明原因	如漏服、多服，或其他情况请说明
第__天	□0 否<br>□1 是	|__|__|__|__|年|__|__|月|__|__|日	|__|__|__|__|__|	|__|__|__|__|__|	|__| ________	|__| ________	□1 不良事件，编号：___<br>□2 其他：________	
第__天	□0 否<br>□1 是	|__|__|__|__|年|__|__|月|__|__|日	|__|__|__|__|__|	|__|__|__|__|__|	|__| ________	|__| ________	□1 不良事件，编号：___<br>□2 其他：________	
第__天	□0 否<br>□1 是	|__|__|__|__|年|__|__|月|__|__|日	|__|__|__|__|__|	|__|__|__|__|__|	|__| ________	|__| ________	□1 不良事件，编号：___<br>□2 其他：________	
第__天	□0 否<br>□1 是	|__|__|__|__|年|__|__|月|__|__|日	|__|__|__|__|__|	|__|__|__|__|__|	|__| ________	|__| ________	□1 不良事件，编号：___<br>□2 其他：________	
第__天	□0 否<br>□1 是	|__|__|__|__|年|__|__|月|__|__|日	|__|__|__|__|__|	|__|__|__|__|__|	|__| ________	|__| ________	□1 不良事件，编号：___<br>□2 其他：________	
第__天	□0 否<br>□1 是	|__|__|__|__|年|__|__|月|__|__|日	|__|__|__|__|__|	|__|__|__|__|__|	|__| ________	|__| ________	□1 不良事件，编号：___<br>□2 其他：________	
第__天	□0 否<br>□1 是	|__|__|__|__|年|__|__|月|__|__|日	|__|__|__|__|__|	|__|__|__|__|__|	|__| ________	|__| ________	□1 不良事件，编号：___<br>□2 其他：________	
第__天	□0 否<br>□1 是	|__|__|__|__|年|__|__|月|__|__|日	|__|__|__|__|__|	|__|__|__|__|__|	|__| ________	|__| ________	□1 不良事件，编号：___<br>□2 其他：________	

（1）给药频率：1=每日三次 2=每日两次 3=每日一次 4=未知 5=其他，请在横线处具体描述

（2）给药情况：1=不适用 2=剂量调整 3=漏服 4=多服 5=暂停给药 6=其他，请在横线处具体描述

试验药物使用情况								
服药天数	是否用药	用药日期	计划每日给药总剂量（mg）	实际每日给药总剂量（mg）	给药频率（1）	给药情况（2）	如剂量调整或暂停给药，请说明原因	如漏服、多服，或其他情况请说明
第__天	□0 否 □1 是	|__|__|__|__|年|__|__|月|__|__|日	|__|__|__|__|__|	|__|__|__|__|__|	|__| ________	|__| ________	□1 不良事件，编号：___ □2 其他：________	
第__天	□0 否 □1 是	|__|__|__|__|年|__|__|月|__|__|日	|__|__|__|__|__|	|__|__|__|__|__|	|__| ________	|__| ________	□1 不良事件，编号：___ □2 其他：________	
第__天	□0 否 □1 是	|__|__|__|__|年|__|__|月|__|__|日	|__|__|__|__|__|	|__|__|__|__|__|	|__| ________	|__| ________	□1 不良事件，编号：___ □2 其他：________	
第__天	□0 否 □1 是	|__|__|__|__|年|__|__|月|__|__|日	|__|__|__|__|__|	|__|__|__|__|__|	|__| ________	|__| ________	□1 不良事件，编号：___ □2 其他：________	
第__天	□0 否 □1 是	|__|__|__|__|年|__|__|月|__|__|日	|__|__|__|__|__|	|__|__|__|__|__|	|__| ________	|__| ________	□1 不良事件，编号：___ □2 其他：________	
第__天	□0 否 □1 是	|__|__|__|__|年|__|__|月|__|__|日	|__|__|__|__|__|	|__|__|__|__|__|	|__| ________	|__| ________	□1 不良事件，编号：___ □2 其他：________	
第__天	□0 否 □1 是	|__|__|__|__|年|__|__|月|__|__|日	|__|__|__|__|__|	|__|__|__|__|__|	|__| ________	|__| ________	□1 不良事件，编号：___ □2 其他：________	
第__天	□0 否 □1 是	|__|__|__|__|年|__|__|月|__|__|日	|__|__|__|__|__|	|__|__|__|__|__|	|__| ________	|__| ________	□1 不良事件，编号：___ □2 其他：________	

（1）给药频率：1=每日三次 2=每日两次 3=每日一次 4=未知 5=其他，请在横线处具体描述

（2）给药情况：1=不适用 2=剂量调整 3=漏服 4=多服 5=暂停给药 6=其他，请在横线处具体描述

受试者是否发生不良事件？ □0 否 □1 是，请参照表格下方的编码详细填写在下表中。

不良事件

编号	不良事件名称	开始日期	研究结束时是否持续？		CTCAE等级（1）	采取的措施		与研究药物的关系（4）	AE转归（5）	是否为SAE？	
			是	否→结束日期		对研究药物的措施（2）	其它措施（3）			否	是，SAE具体类型（6）
1		|__|__|__|__|年<br>|__|__|月|__|__|日	□	|__|__|__|__|年<br>|__|__|月|__|__|日	|__|	|__| ________	|__| ________	|__|	|__|	□	|__|
2		|__|__|__|__|年<br>|__|__|月|__|__|日	□	|__|__|__|__|年<br>|__|__|月|__|__|日	|__|	|__| ________	|__| ________	|__|	|__|	□	|__|
3		|__|__|__|__|年<br>|__|__|月|__|__|日	□	|__|__|__|__|年<br>|__|__|月|__|__|日	|__|	|__| ________	|__| ________	|__|	|__|	□	|__|
4		|__|__|__|__|年<br>|__|__|月|__|__|日	□	|__|__|__|__|年<br>|__|__|月|__|__|日	|__|	|__| ________	|__| ________	|__|	|__|	□	|__|

（1）CTCAE等级	（2）对研究药物的措施	（3）其它措施	（4）与研究药物关系	（5）AE转归	（6）SAE具体类型
1=1级<br>2=2级<br>3=3级<br>4=4级<br>5=5级	1=维持目前治疗<br>2=用药暂停<br>3=用药终止<br>4=减小剂量<br>5=不适用<br>6=其它，请在横线处具体描述	1=无<br>2=合并用药，填写既往/合并药物治疗表<br>3=合并非药物治疗，填写既往/合并非药物治疗表<br>4=其它，请在横线处具体描述	1=肯定有关<br>2=很可能有关<br>3=可能有关<br>4=可能无关<br>5=肯定无关<br>6=待评价<br>7=无法评价	1=未恢复/未治愈<br>2=恢复/治愈<br>3=恢复但留有后遗症<br>4=死亡<br>5=未知	1=危及生命<br>2=导致死亡<br>3=需住院治疗或延长目前住院时间<br>4=导致永久或显著的残疾或功能障碍<br>5=导致先天畸形/出生缺陷<br>6=其它重要的医学事件

不良事件

编号	不良事件名称	开始日期	研究结束时是否持续？		CTCAE 等级（1）	采取的措施		与研究药物的关系（4）	AE 转归（5）	是否为SAE？	
			是	否→结束日期		对研究药物的措施（2）	其它措施（3）			否	是，SAE 具体类型（6）
		|__|__|__|__|年 |__|__|月|__|__|日	☐	|__|__|__|__|年 |__|__|月|__|__|日	|__|	|__| ________	|__| ________	|__|	|__|	☐	|__|
		|__|__|__|__|年 |__|__|月|__|__|日	☐	|__|__|__|__|年 |__|__|月|__|__|日	|__|	|__| ________	|__| ________	|__|	|__|	☐	|__|
		|__|__|__|__|年 |__|__|月|__|__|日	☐	|__|__|__|__|年 |__|__|月|__|__|日	|__|	|__| ________	|__| ________	|__|	|__|	☐	|__|
		|__|__|__|__|年 |__|__|月|__|__|日	☐	|__|__|__|__|年 |__|__|月|__|__|日	|__|	|__| ________	|__| ________	|__|	|__|	☐	|__|
		|__|__|__|__|年 |__|__|月|__|__|日	☐	|__|__|__|__|年 |__|__|月|__|__|日	|__|	|__| ________	|__| ________	|__|	|__|	☐	|__|

（1）CTCAE等级	（2）对研究药物的措施	（3）其它措施	（4）与研究药物关系	（5）AE转归	（6）SAE具体类型
1=1级<br>2=2级<br>3=3级<br>4=4级<br>5=5级	1=维持目前治疗<br>2=用药暂停<br>3=用药终止<br>4=减小剂量<br>5=不适用<br>6=其它，请在横线处具体描述	1=无<br>2=合并用药，填写既往/合并药物治疗表<br>3=合并非药物治疗，填写既往/合并非药物治疗表<br>4=其它，请在横线处具体描述	1=肯定有关<br>2=很可能有关<br>3=可能有关<br>4=可能无关<br>5=肯定无关<br>6=待评价<br>7=无法评价	1=未恢复/未治愈<br>2=恢复/治愈<br>3=恢复但留有后遗症<br>4=死亡<br>5=未知	1=危及生命<br>2=导致死亡<br>3=需住院治疗或延长目前住院时间<br>4=导致永久或显著的残疾或功能障碍<br>5=导致先天畸形/出生缺陷<br>6=其它重要的医学事件

受试者是否有既往/合并药物治疗？　□0 否　□1 **是**，请参照表格下方的编码详细填写在下表中。

<table>
<tr><th colspan="12">既往/合并药物治疗</th></tr>
<tr><th rowspan="2">编号</th><th rowspan="2">药物名称（通用名）</th><th rowspan="2">适应症（1）</th><th rowspan="2">适应症备注</th><th rowspan="2">单次剂量</th><th rowspan="2">剂量单位（2）</th><th rowspan="2">给药频率（3）</th><th rowspan="2">给药途径（4）</th><th rowspan="2">开始日期</th><th colspan="2">研究结束时是否持续？</th><th rowspan="2">是否为指南推荐的标准治疗（5）</th></tr>
<tr><th>是</th><th>否→结束日期</th></tr>
<tr><td>1</td><td></td><td>|__|</td><td></td><td></td><td>|__|
______</td><td>|__|
______</td><td>|__|
______</td><td>|__|__|__|__|年
|__|__|月|__|__|日</td><td>□</td><td>|__|__|__|__|年
|__|__|月|__|__|日</td><td>|__|</td></tr>
<tr><td>2</td><td></td><td>|__|</td><td></td><td></td><td>|__|
______</td><td>|__|
______</td><td>|__|
______</td><td>|__|__|__|__|年
|__|__|月|__|__|日</td><td>□</td><td>|__|__|__|__|年
|__|__|月|__|__|日</td><td>|__|</td></tr>
<tr><td>3</td><td></td><td>|__|</td><td></td><td></td><td>|__|
______</td><td>|__|
______</td><td>|__|
______</td><td>|__|__|__|__|年
|__|__|月|__|__|日</td><td>□</td><td>|__|__|__|__|年
|__|__|月|__|__|日</td><td>|__|</td></tr>
<tr><td>4</td><td></td><td>|__|</td><td></td><td></td><td>|__|
______</td><td>|__|
______</td><td>|__|
______</td><td>|__|__|__|__|年
|__|__|月|__|__|日</td><td>□</td><td>|__|__|__|__|年
|__|__|月|__|__|日</td><td>|__|</td></tr>
<tr><td>5</td><td></td><td>|__|</td><td></td><td></td><td>|__|
______</td><td>|__|
______</td><td>|__|
______</td><td>|__|__|__|__|年
|__|__|月|__|__|日</td><td>□</td><td>|__|__|__|__|年
|__|__|月|__|__|日</td><td>|__|</td></tr>
</table>

（1）适应症	（2）剂量单位		（3）给药频率		（4）给药途径		（5）指南推荐标准治疗
1=本研究疾病<br>2=既往/伴随疾病（请在备注处填写既往/伴随疾病编号）<br>3=不良事件（请在备注处填写不良事件编号）<br>4=其它（请在备注处描述）	1=微克(μg)<br>2=毫克(mg)<br>3=克(g)<br>4=毫升(mL)<br>5=国际单位(IU)	6=单位(U)<br>7=片(Tab)<br>8=胶囊(Cap)<br>9=未知<br>10=其它，请在横线处填写	1=每日一次(qd)<br>2=每日二次(bid)<br>3=每日三次(tid)<br>4=每日四次(qid)<br>5=隔日一次(qod)	6=每周一次(qw)<br>7=睡前(hs)<br>8=按需(prn)<br>9=未知<br>10=其它，请在横线处填写	1=口服(PO)<br>2=静脉滴注(IVgtt)<br>3=静脉注射(IV)<br>4=肌内注射(IM)<br>5=皮下注射(SQ)<br>6=舌下(SL)	7=局部（皮肤）(TOP)<br>8=眼(OPH)<br>9=吸入(INH)<br>10=未知<br>11=其它，请在横线处填写	0=否<br>1=是<br>2=不适用

既往/合并药物治疗									研究结束时是否持续？		
编号	药物名称（通用名）	适应症（1）	适应症备注	单次剂量	剂量单位（2）	给药频率（3）	给药途径（4）	开始日期	是	否→结束日期	是否为指南推荐的标准治疗（5）
		|__|			|__|	|__|	|__|	|__|__|__|__|年 |__|__|月|__|__|日	□	|__|__|__|__|年 |__|__|月|__|__|日	|__|
		|__|			|__|	|__|	|__|	|__|__|__|__|年 |__|__|月|__|__|日	□	|__|__|__|__|年 |__|__|月|__|__|日	|__|
		|__|			|__|	|__|	|__|	|__|__|__|__|年 |__|__|月|__|__|日	□	|__|__|__|__|年 |__|__|月|__|__|日	|__|
		|__|			|__|	|__|	|__|	|__|__|__|__|年 |__|__|月|__|__|日	□	|__|__|__|__|年 |__|__|月|__|__|日	|__|
		|__|			|__|	|__|	|__|	|__|__|__|__|年 |__|__|月|__|__|日	□	|__|__|__|__|年 |__|__|月|__|__|日	|__|

（1）适应症	（2）剂量单位		（3）给药频率		（4）给药途径		（5）指南推荐标准治疗
1=本研究疾病<br>2=既往/伴随疾病（请在备注处填写既往/伴随疾病编号）<br>3=不良事件（请在备注处填写不良事件编号）<br>4=其它（请在备注处描述）	1=微克(μg)<br>2=毫克(mg)<br>3=克(g)<br>4=毫升(mL)<br>5=国际单位(IU)	6=单位(U)<br>7=片(Tab)<br>8=胶囊(Cap)<br>9=未知<br>10=其它，请在横线处填写	1=每日一次(qd)<br>2=每日二次(bid)<br>3=每日三次(tid)<br>4=每日四次(qid)<br>5=隔日一次(qod)	6=每周一次(qw)<br>7=睡前(hs)<br>8=按需(prn)<br>9=未知<br>10=其它，请在横线处填写	1=口服(PO)<br>2=静脉滴注(IVgtt)<br>3=静脉注射(IV)<br>4=肌内注射(IM)<br>5=皮下注射(SQ)<br>6=舌下(SL)	7=局部（皮肤）(TOP)<br>8=眼(OPH)<br>9=吸入(INH)<br>10=未知<br>11=其它，请在横线处填写	0=否<br>1=是<br>2=不适用

受试者是否有既往/合并非药物治疗？ □0 否 □1 是，请继续填写下表。

既往/合并非药物治疗

编号	治疗名称（请用专业的医学术语描述）	适应症（1）	适应症备注	开始日期	研究结束时是否持续？		是否为指南推荐的标准治疗（2）
					是	否→结束日期	
1		|__|		|__|__|__|__|年|__|__|月|__|__|日	□	|__|__|__|__|年|__|__|月|__|__|日	|__|
2		|__|		|__|__|__|__|年|__|__|月|__|__|日	□	|__|__|__|__|年|__|__|月|__|__|日	|__|
3		|__|		|__|__|__|__|年|__|__|月|__|__|日	□	|__|__|__|__|年|__|__|月|__|__|日	|__|
4		|__|		|__|__|__|__|年|__|__|月|__|__|日	□	|__|__|__|__|年|__|__|月|__|__|日	|__|
5		|__|		|__|__|__|__|年|__|__|月|__|__|日	□	|__|__|__|__|年|__|__|月|__|__|日	|__|
6		|__|		|__|__|__|__|年|__|__|月|__|__|日	□	|__|__|__|__|年|__|__|月|__|__|日	|__|
7		|__|		|__|__|__|__|年|__|__|月|__|__|日	□	|__|__|__|__|年|__|__|月|__|__|日	|__|
8		|__|		|__|__|__|__|年|__|__|月|__|__|日	□	|__|__|__|__|年|__|__|月|__|__|日	|__|

（1）适应症：1=本研究疾病 2=既往/伴随疾病（请在备注处填写既往/伴随疾病编号） 3=不良事件（请在备注处填写不良事件编号） 4=其它（请在备注处描述）

（2）是否为指南推荐的标准治疗：0=否 1=是 2=不适用

既往/合并非药物治疗							
编号	治疗名称<br>（请用专业的医学术语描述）	适应症（1）	适应症备注	开始日期	研究结束时是否持续？		是否为指南推荐的标准治疗（2）
					是	否→结束日期	
		|__|		|__|__|__|__|年|__|__|月|__|__|日	☐	|__|__|__|__|年|__|__|月|__|__|日	|__|
		|__|		|__|__|__|__|年|__|__|月|__|__|日	☐	|__|__|__|__|年|__|__|月|__|__|日	|__|
		|__|		|__|__|__|__|年|__|__|月|__|__|日	☐	|__|__|__|__|年|__|__|月|__|__|日	|__|
		|__|		|__|__|__|__|年|__|__|月|__|__|日	☐	|__|__|__|__|年|__|__|月|__|__|日	|__|
		|__|		|__|__|__|__|年|__|__|月|__|__|日	☐	|__|__|__|__|年|__|__|月|__|__|日	|__|
		|__|		|__|__|__|__|年|__|__|月|__|__|日	☐	|__|__|__|__|年|__|__|月|__|__|日	|__|
		|__|		|__|__|__|__|年|__|__|月|__|__|日	☐	|__|__|__|__|年|__|__|月|__|__|日	|__|
		|__|		|__|__|__|__|年|__|__|月|__|__|日	☐	|__|__|__|__|年|__|__|月|__|__|日	|__|
		|__|		|__|__|__|__|年|__|__|月|__|__|日	☐	|__|__|__|__|年|__|__|月|__|__|日	|__|

（1）适应症：1=本研究疾病　2=既往/伴随疾病（请在备注处填写既往/伴随疾病编号）　3=不良事件（请在备注处填写不良事件编号）　4=其它（请在备注处描述）
（2）是否为指南推荐的标准治疗：0=否　1=是　2=不适用

受试者是否有IMV/NIV、ECMO、HFNC、氧疗？ □0 否 □1 是，请继续填写下表。					
**IMV/NIV、ECMO、HFNC、氧疗**					
编号	治疗名称	治疗开始日期	治疗开始时间	治疗结束日期	治疗结束时间
**1**	□1 IMV □3 ECMO<br>□2 NIV □4 HFNC<br>□5 氧疗	|__|__|__|__|年|__|__|月|__|__|日	|__|__|:|__|__|	|__|__|__|__|年|__|__|月|__|__|日	|__|__|:|__|__|
**2**	□1 IMV □3 ECMO<br>□2 NIV □4 HFNC<br>□5 氧疗	|__|__|__|__|年|__|__|月|__|__|日	|__|__|:|__|__|	|__|__|__|__|年|__|__|月|__|__|日	|__|__|:|__|__|
**3**	□1 IMV □3 ECMO<br>□2 NIV □4 HFNC<br>□5 氧疗	|__|__|__|__|年|__|__|月|__|__|日	|__|__|:|__|__|	|__|__|__|__|年|__|__|月|__|__|日	|__|__|:|__|__|
**4**	□1 IMV □3 ECMO<br>□2 NIV □4 HFNC<br>□5 氧疗	|__|__|__|__|年|__|__|月|__|__|日	|__|__|:|__|__|	|__|__|__|__|年|__|__|月|__|__|日	|__|__|:|__|__|
**5**	□1 IMV □3 ECMO<br>□2 NIV □4 HFNC<br>□5 氧疗	|__|__|__|__|年|__|__|月|__|__|日	|__|__|:|__|__|	|__|__|__|__|年|__|__|月|__|__|日	|__|__|:|__|__|
**6**	□1 IMV □3 ECMO<br>□2 NIV □4 HFNC<br>□5 氧疗	|__|__|__|__|年|__|__|月|__|__|日	|__|__|:|__|__|	|__|__|__|__|年|__|__|月|__|__|日	|__|__|:|__|__|
**7**	□1 IMV □3 ECMO<br>□2 NIV □4 HFNC<br>□5 氧疗	|__|__|__|__|年|__|__|月|__|__|日	|__|__|:|__|__|	|__|__|__|__|年|__|__|月|__|__|日	|__|__|:|__|__|

IMV/NIV、ECMO、HFNC、氧疗

编号	治疗名称	治疗开始日期	治疗开始时间	治疗结束日期	治疗结束时间
	□1 IMV □3 ECMO<br>□2 NIV □4 HFNC<br>□5 氧疗	|__|__|__|__|年|__|__|月|__|__|日	|__|__|:|__|__|	|__|__|__|__|年|__|__|月|__|__|日	|__|__|:|__|__|
	□1 IMV □3 ECMO<br>□2 NIV □4 HFNC<br>□5 氧疗	|__|__|__|__|年|__|__|月|__|__|日	|__|__|:|__|__|	|__|__|__|__|年|__|__|月|__|__|日	|__|__|:|__|__|
	□1 IMV □3 ECMO<br>□2 NIV □4 HFNC<br>□5 氧疗	|__|__|__|__|年|__|__|月|__|__|日	|__|__|:|__|__|	|__|__|__|__|年|__|__|月|__|__|日	|__|__|:|__|__|
	□1 IMV □3 ECMO<br>□2 NIV □4 HFNC<br>□5 氧疗	|__|__|__|__|年|__|__|月|__|__|日	|__|__|:|__|__|	|__|__|__|__|年|__|__|月|__|__|日	|__|__|:|__|__|
	□1 IMV □3 ECMO<br>□2 NIV □4 HFNC<br>□5 氧疗	|__|__|__|__|年|__|__|月|__|__|日	|__|__|:|__|__|	|__|__|__|__|年|__|__|月|__|__|日	|__|__|:|__|__|
	□1 IMV □3 ECMO<br>□2 NIV □4 HFNC<br>□5 氧疗	|__|__|__|__|年|__|__|月|__|__|日	|__|__|:|__|__|	|__|__|__|__|年|__|__|月|__|__|日	|__|__|:|__|__|
	□1 IMV □3 ECMO<br>□2 NIV □4 HFNC<br>□5 氧疗	|__|__|__|__|年|__|__|月|__|__|日	|__|__|:|__|__|	|__|__|__|__|年|__|__|月|__|__|日	|__|__|:|__|__|

受试者是否有其他检查需要记录？ □0 否 □1 是，请填写下表。

其他检查							
**检查项目**	**采样/检查日期**	**检查结果**	**单位**	**结果判定**<br>**1=正常**<br>**2=异常无临床意义**<br>**3=异常有临床意义**			**备注**
	|__|__|__|__|年|__|__|月|__|__|日			□1	□2	□3	
	|__|__|__|__|年|__|__|月|__|__|日			□1	□2	□3	
	|__|__|__|__|年|__|__|月|__|__|日			□1	□2	□3	
	|__|__|__|__|年|__|__|月|__|__|日			□1	□2	□3	
	|__|__|__|__|年|__|__|月|__|__|日			□1	□2	□3	
	|__|__|__|__|年|__|__|月|__|__|日			□1	□2	□3	
	|__|__|__|__|年|__|__|月|__|__|日			□1	□2	□3	

其他检查							
检查项目	采样/检查日期	检查结果	单位	结果判定<br>**1=正常**<br>**2=异常无临床意义**<br>**3=异常有临床意义**			备注
	|__|__|__|__|年|__|__|月|__|__|日			□1	□2	□3	
	|__|__|__|__|年|__|__|月|__|__|日			□1	□2	□3	
	|__|__|__|__|年|__|__|月|__|__|日			□1	□2	□3	
	|__|__|__|__|年|__|__|月|__|__|日			□1	□2	□3	
	|__|__|__|__|年|__|__|月|__|__|日			□1	□2	□3	
	|__|__|__|__|年|__|__|月|__|__|日			□1	□2	□3	
	|__|__|__|__|年|__|__|月|__|__|日			□1	□2	□3	

研究总结
受试者是否按研究方案完成了研究？<br>□1 是，完成日期：|__|__|__|__|年|__|__|月|__|__|日<br>□0 否，终止日期：|__|__|__|__|年|__|__|月|__|__|日
如提前退出研究，请选择最重要的原因（单选）：<br>□1 研究期间出现疾病恶化，经研究者综合评估后需要改变治疗<br>□2 药物不良事件导致病情加重的出现<br>□3 患者不愿意继续使用研究药物<br>□4 妊娠<br>□5 受试者失访<br>□6 受试者死亡<br>□7 受试者撤回知情同意<br>□8 研究整体中止<br>□9 其他，请描述：____<br>____<br><br>备注（请记录其他需要补充说明的事由）：<br>____<br>____<br>____

住院情况
受试者是否因新型冠状病毒肺炎住院？ □0 否 □1 是
如是，入院日期：|__|__|__|__|年|__|__|月|__|__|日
研究结束时是否出院：□0 否 □1 是
如是，出院日期：|__|__|__|__|年|__|__|月|__|__|日

死亡
受试者是否死亡？ □$_0$ 否 □$_1$ 是，请填写。 □$_2$ 未知
死亡日期：|__|__|__|__|年|__|__|月|__|__|日
死亡原因：□$_1$ 不良事件<br>□$_2$ 未知<br>□$_3$ 其他，请描述：________________________________<br>____<br>____

研究者声明
我已审核并确认此病例报告表中记录的所有数据均真实、准确、可靠、完整无误，并与原始资料一致，所有错误或遗漏均已得到更正或注明。<br><br>**主要研究者签字：______________________　　签字日期：|__|__|__|__|年|__|__|月|__|__|日**

CRA 声明
我已核对并确认此病例报告表中记录的所有数据均真实、准确、可靠、完整无误，并与原始资料一致。<br><br>**CRA 签字：__________________________　　签字日期：|__|__|__|__|年|__|__|月|__|__|日**

生命体征	□0 未查，原因________________		
检查日期	|__|__|__|__|年|__|__|月|__|__|日		
日最高体温	|__|__|.|__|℃	测量方式	□1 腋下 □2 口腔 □3 肛门<br>□4 耳朵 □5 未知 □6 其他，请描述______
呼吸频率	|__|__|次/分	脉搏	|__|__|__|次/分
血压	|__|__|__|/|__|__|__| mmHg（收缩压/舒张压）		

生命体征	□0 未查，原因________________		
检查日期	|__|__|__|__|年|__|__|月|__|__|日		
日最高体温	|__|__|.|__|℃	测量方式	□1 腋下 □2 口腔 □3 肛门<br>□4 耳朵 □5 未知 □6 其他，请描述______
呼吸频率	|__|__|次/分	脉搏	|__|__|__|次/分
血压	|__|__|__|/|__|__|__| mmHg（收缩压/舒张压）		

生命体征	□0 未查，原因________________		
检查日期	|__|__|__|__|年|__|__|月|__|__|日		
日最高体温	|__|__|.|__|℃	测量方式	□1 腋下 □2 口腔 □3 肛门<br>□4 耳朵 □5 未知 □6 其他，请描述______
呼吸频率	|__|__|次/分	脉搏	|__|__|__|次/分
血压	|__|__|__|/|__|__|__| mmHg（收缩压/舒张压）		

生命体征	□0 未查，原因________________		
检查日期	|__|__|__|__|年|__|__|月|__|__|日		
日最高体温	|__|__|.|__|℃	测量方式	□1 腋下 □2 口腔 □3 肛门<br>□4 耳朵 □5 未知 □6 其他，请描述______
呼吸频率	|__|__|次/分	脉搏	|__|__|__|次/分
血压	|__|__|__|/|__|__|__| mmHg（收缩压/舒张压）		

**体格检查** ☐$_0$ 未查，原因________________					
检查日期：|__|__|__|__|年|__|__|月|__|__|日					
**检查部位**	**检查结果**<br>**1=正常**<br>**2=异常无临床意义**<br>**3=异常有临床意义**<br>**0=未查**				**异常请描述**
整体外观	☐$_1$	☐$_2$	☐$_3$	☐$_0$	
头	☐$_1$	☐$_2$	☐$_3$	☐$_0$	
眼	☐$_1$	☐$_2$	☐$_3$	☐$_0$	
耳	☐$_1$	☐$_2$	☐$_3$	☐$_0$	
鼻	☐$_1$	☐$_2$	☐$_3$	☐$_0$	
喉	☐$_1$	☐$_2$	☐$_3$	☐$_0$	
颈部及甲状腺	☐$_1$	☐$_2$	☐$_3$	☐$_0$	
皮肤	☐$_1$	☐$_2$	☐$_3$	☐$_0$	
心血管系统	☐$_1$	☐$_2$	☐$_3$	☐$_0$	
胸部及肺	☐$_1$	☐$_2$	☐$_3$	☐$_0$	
腹部	☐$_1$	☐$_2$	☐$_3$	☐$_0$	
神经系统	☐$_1$	☐$_2$	☐$_3$	☐$_0$	
骨骼肌系统	☐$_1$	☐$_2$	☐$_3$	☐$_0$	
淋巴系统	☐$_1$	☐$_2$	☐$_3$	☐$_0$	

**有无呼吸道症状**　□0 未查，原因________
检查日期：|__|__|__|__|年|__|__|月|__|__|日
受试者有无呼吸道症状：□0 无　□1 有，请描述：________

**有无呼吸道症状**　□0 未查，原因________
检查日期：|__|__|__|__|年|__|__|月|__|__|日
受试者有无呼吸道症状：□0 无　□1 有，请描述：________

**有无呼吸道症状**　□0 未查，原因________
检查日期：|__|__|__|__|年|__|__|月|__|__|日
受试者有无呼吸道症状：□0 无　□1 有，请描述：________

**有无呼吸道症状**　□0 未查，原因________
检查日期：|__|__|__|__|年|__|__|月|__|__|日
受试者有无呼吸道症状：□0 无　□1 有，请描述：________

**有无呼吸道症状**　□0 未查，原因________
检查日期：|__|__|__|__|年|__|__|月|__|__|日
受试者有无呼吸道症状：□0 无　□1 有，请描述：________

**有无呼吸道症状**　□0 未查，原因________
检查日期：|__|__|__|__|年|__|__|月|__|__|日
受试者有无呼吸道症状：□0 无　□1 有，请描述：________

**有无呼吸道症状**　□0 未查，原因________
检查日期：|__|__|__|__|年|__|__|月|__|__|日
受试者有无呼吸道症状：□0 无　□1 有，请描述：________

血常规 □$_0$ 未查，原因________________

采样日期：|__|__|__|__|年|__|__|月|__|__|日 正常值范围编号：|__|__|__|__|

检查项目	测量结果		结果判定			未查
	实测值	单位	正常	异常		
				无临床意义	有临床意义	
红细胞计数（RBC）		□$_1$ 10^{12}/L □$_2$ 其他：_____	□$_1$	□$_2$	□$_3$	□$_0$
白细胞计数（WBC）		□$_1$ 10^9/L □$_2$ 其他：_____	□$_1$	□$_2$	□$_3$	□$_0$
血红蛋白（Hb）		□$_1$ g/L □$_2$ 其他：_____	□$_1$	□$_2$	□$_3$	□$_0$
血小板计数（PLT）		□$_1$ 10^9/L □$_2$ 其他：_____	□$_1$	□$_2$	□$_3$	□$_0$
中性粒细胞计数（ANC）		□$_1$ 10^9/L □$_2$ 其他：_____	□$_1$	□$_2$	□$_3$	□$_0$
淋巴细胞计数（LY）		□$_1$ 10^9/L □$_2$ 其他：_____	□$_1$	□$_2$	□$_3$	□$_0$
单核细胞计数（MO）		□$_1$ 10^9/L □$_2$ 其他：_____	□$_1$	□$_2$	□$_3$	□$_0$
嗜酸性粒细胞计数（EO）		□$_1$ 10^9/L □$_2$ 其他：_____	□$_1$	□$_2$	□$_3$	□$_0$
嗜碱性粒细胞计数（BA）		□$_1$ 10^9/L □$_2$ 其他：_____	□$_1$	□$_2$	□$_3$	□$_0$

血常规 □$_0$ 未查，原因________________

采样日期：|__|__|__|__|年|__|__|月|__|__|日 正常值范围编号：|__|__|__|__|

检查项目	测量结果		结果判定			未查
	实测值	单位	正常	异常		
				无临床意义	有临床意义	
红细胞计数（RBC）		□$_1$ 10^{12}/L □$_2$ 其他：_____	□$_1$	□$_2$	□$_3$	□$_0$
白细胞计数（WBC）		□$_1$ 10^9/L □$_2$ 其他：_____	□$_1$	□$_2$	□$_3$	□$_0$
血红蛋白（Hb）		□$_1$ g/L □$_2$ 其他：_____	□$_1$	□$_2$	□$_3$	□$_0$
血小板计数（PLT）		□$_1$ 10^9/L □$_2$ 其他：_____	□$_1$	□$_2$	□$_3$	□$_0$
中性粒细胞计数（ANC）		□$_1$ 10^9/L □$_2$ 其他：_____	□$_1$	□$_2$	□$_3$	□$_0$
淋巴细胞计数（LY）		□$_1$ 10^9/L □$_2$ 其他：_____	□$_1$	□$_2$	□$_3$	□$_0$
单核细胞计数（MO）		□$_1$ 10^9/L □$_2$ 其他：_____	□$_1$	□$_2$	□$_3$	□$_0$
嗜酸性粒细胞计数（EO）		□$_1$ 10^9/L □$_2$ 其他：_____	□$_1$	□$_2$	□$_3$	□$_0$
嗜碱性粒细胞计数（BA）		□$_1$ 10^9/L □$_2$ 其他：_____	□$_1$	□$_2$	□$_3$	□$_0$

血生化　　　　□0 未查，原因________________

采样日期|__|__|__|__|年|__|__|月|__|__|日　　　　正常值范围编号：|__|__|__|__|

检查项目	测量结果		结果判定			未查
	实测值	单位	正常	异常		
				无临床意义	有临床意义	
谷丙转氨酶（ALT）		□1 U/L　□2 其他：_____	□1	□2	□3	□0
谷草转氨酶（AST）		□1 U/L　□2 其他：_____	□1	□2	□3	□0
总胆红素（TBIL）		□1 μmol/L　□2 其他：_____	□1	□2	□3	□0
白蛋白（ALB）		□1 g/L　□2 其他：_____	□1	□2	□3	□0
γ-谷氨酰转肽酶（GGT）		□1 U/L　□2 其他：_____	□1	□2	□3	□0
乳酸脱氢酶（LDH）		□1 U/L　□2 其他：_____	□1	□2	□3	□0
肌酸激酶（CK）		□1 U/L　□2 其他：_____	□1	□2	□3	□0
肌酸激酶同工酶（CK-MB）		□1 U/L　□2 其他：_____	□1	□2	□3	□0
血尿素氮（BUN）		□1 mmol/L　□2 其他：_____	□1	□2	□3	□0
尿素（Urea）		□1 mmol/L　□2 其他：_____	□1	□2	□3	□0
血肌酐（CR）		□1 μmol/L　□2 其他：_____	□1	□2	□3	□0

血生化　　　　□0 未查，原因________________

采样日期|__|__|__|__|年|__|__|月|__|__|日　　　　正常值范围编号：|__|__|__|__|

检查项目	测量结果		结果判定			未查
	实测值	单位	正常	异常		
				无临床意义	有临床意义	
谷丙转氨酶（ALT）		□1 U/L　□2 其他：_____	□1	□2	□3	□0
谷草转氨酶（AST）		□1 U/L　□2 其他：_____	□1	□2	□3	□0
总胆红素（TBIL）		□1 μmol/L　□2 其他：_____	□1	□2	□3	□0
白蛋白（ALB）		□1 g/L　□2 其他：_____	□1	□2	□3	□0
γ-谷氨酰转肽酶（GGT）		□1 U/L　□2 其他：_____	□1	□2	□3	□0
乳酸脱氢酶（LDH）		□1 U/L　□2 其他：_____	□1	□2	□3	□0
肌酸激酶（CK）		□1 U/L　□2 其他：_____	□1	□2	□3	□0
肌酸激酶同工酶（CK-MB）		□1 U/L　□2 其他：_____	□1	□2	□3	□0
血尿素氮（BUN）		□1 mmol/L　□2 其他：_____	□1	□2	□3	□0
尿素（Urea）		□1 mmol/L　□2 其他：_____	□1	□2	□3	□0
血肌酐（CR）		□1 μmol/L　□2 其他：_____	□1	□2	□3	□0

尿常规 □0 未查，原因____

采样日期：|__|__|__|__|年|__|__|月|__|__|日 正常值范围编号：|__|__|__|__|

检查项目	测量结果		结果判定			未查
	实测值	单位	正常	异常		
				无临床意义	有临床意义	
尿蛋白		□1 不适用 □2 其他：____	□1	□2	□3	□0
尿葡萄糖		□1 mmol/L □2 其他：____	□1	□2	□3	□0
尿 PH 值		□1 不适用 □2 其他：____	□1	□2	□3	□0
尿红细胞		□1 不适用 □2 其他：____	□1	□2	□3	□0
尿白细胞		□1 不适用 □2 其他：____	□1	□2	□3	□0
尿酮体		□1 不适用 □2 其他：____	□1	□2	□3	□0

尿常规 □0 未查，原因____

采样日期：|__|__|__|__|年|__|__|月|__|__|日 正常值范围编号：|__|__|__|__|

检查项目	测量结果		结果判定			未查
	实测值	单位	正常	异常		
				无临床意义	有临床意义	
尿蛋白		□1 不适用 □2 其他：____	□1	□2	□3	□0
尿葡萄糖		□1 mmol/L □2 其他：____	□1	□2	□3	□0
尿 PH 值		□1 不适用 □2 其他：____	□1	□2	□3	□0
尿红细胞		□1 不适用 □2 其他：____	□1	□2	□3	□0
尿白细胞		□1 不适用 □2 其他：____	□1	□2	□3	□0
尿酮体		□1 不适用 □2 其他：____	□1	□2	□3	□0

尿常规 □0 未查，原因____

采样日期：|__|__|__|__|年|__|__|月|__|__|日 正常值范围编号：|__|__|__|__|

检查项目	测量结果		结果判定			未查
	实测值	单位	正常	异常		
				无临床意义	有临床意义	
尿蛋白		□1 不适用 □2 其他：____	□1	□2	□3	□0
尿葡萄糖		□1 mmol/L □2 其他：____	□1	□2	□3	□0
尿 PH 值		□1 不适用 □2 其他：____	□1	□2	□3	□0
尿红细胞		□1 不适用 □2 其他：____	□1	□2	□3	□0
尿白细胞		□1 不适用 □2 其他：____	□1	□2	□3	□0
尿酮体		□1 不适用 □2 其他：____	□1	□2	□3	□0

凝血功能 □0 未查，原因________________________________

采样日期：|__|__|__|__|年|__|__|月|__|__|日 正常值范围编号：|__|__|__|__|

检查项目	测量结果		结果判定			未查
	实测值	单位	正常	异常		
				无临床意义	有临床意义	
凝血酶原时间（PT）		□1 S □2 其他：_____	□1	□2	□3	□0
活化部分凝血活酶时间（APTT）		□1 S □2 其他：_____	□1	□2	□3	□0
凝血酶时间（TT）		□1 S □2 其他：_____	□1	□2	□3	□0
血浆纤维蛋白原（Fbg）		□1 g/L □2 其他：_____	□1	□2	□3	□0
国际标准化比率（INR）		□1 不适用 □2 其他：_____	□1	□2	□3	□0

凝血功能 □0 未查，原因________________________________

采样日期：|__|__|__|__|年|__|__|月|__|__|日 正常值范围编号：|__|__|__|__|

检查项目	测量结果		结果判定			未查
	实测值	单位	正常	异常		
				无临床意义	有临床意义	
凝血酶原时间（PT）		□1 S □2 其他：_____	□1	□2	□3	□0
活化部分凝血活酶时间（APTT）		□1 S □2 其他：_____	□1	□2	□3	□0
凝血酶时间（TT）		□1 S □2 其他：_____	□1	□2	□3	□0
血浆纤维蛋白原（Fbg）		□1 g/L □2 其他：_____	□1	□2	□3	□0
国际标准化比率（INR）		□1 不适用 □2 其他：_____	□1	□2	□3	□0

凝血功能 □0 未查，原因________________________________

采样日期：|__|__|__|__|年|__|__|月|__|__|日 正常值范围编号：|__|__|__|__|

检查项目	测量结果		结果判定			未查
	实测值	单位	正常	异常		
				无临床意义	有临床意义	
凝血酶原时间（PT）		□1 S □2 其他：_____	□1	□2	□3	□0
活化部分凝血活酶时间（APTT）		□1 S □2 其他：_____	□1	□2	□3	□0
凝血酶时间（TT）		□1 S □2 其他：_____	□1	□2	□3	□0
血浆纤维蛋白原（Fbg）		□1 g/L □2 其他：_____	□1	□2	□3	□0
国际标准化比率（INR）		□1 不适用 □2 其他：_____	□1	□2	□3	□0

C 反应蛋白（CRP） □$_0$未查，原因________________________________

采样日期：|__|__|__|__|年|__|__|月|__|__|日 正常值范围编号：|__|__|__|__|

检查项目	测量结果		结果判定		
C 反应蛋白（CRP）	实测值	单位	正常	异常	
				无临床意义	有临床意义
		□$_1$ mg/L □$_2$其他：_____	□$_1$	□$_2$	□$_3$

C 反应蛋白（CRP） □$_0$未查，原因________________________________

采样日期：|__|__|__|__|年|__|__|月|__|__|日 正常值范围编号：|__|__|__|__|

检查项目	测量结果		结果判定		
C 反应蛋白（CRP）	实测值	单位	正常	异常	
				无临床意义	有临床意义
		□$_1$ mg/L □$_2$其他：_____	□$_1$	□$_2$	□$_3$

C 反应蛋白（CRP） □$_0$未查，原因________________________________

采样日期：|__|__|__|__|年|__|__|月|__|__|日 正常值范围编号：|__|__|__|__|

检查项目	测量结果		结果判定		
C 反应蛋白（CRP）	实测值	单位	正常	异常	
				无临床意义	有临床意义
		□$_1$ mg/L □$_2$其他：_____	□$_1$	□$_2$	□$_3$

C 反应蛋白（CRP） □$_0$未查，原因________________________________

采样日期：|__|__|__|__|年|__|__|月|__|__|日 正常值范围编号：|__|__|__|__|

检查项目	测量结果		结果判定		
C 反应蛋白（CRP）	实测值	单位	正常	异常	
				无临床意义	有临床意义
		□$_1$ mg/L □$_2$其他：_____	□$_1$	□$_2$	□$_3$

C 反应蛋白（CRP） □$_0$未查，原因________________________________

采样日期：|__|__|__|__|年|__|__|月|__|__|日 正常值范围编号：|__|__|__|__|

检查项目	测量结果		结果判定		
C 反应蛋白（CRP）	实测值	单位	正常	异常	
				无临床意义	有临床意义
		□$_1$ mg/L □$_2$其他：_____	□$_1$	□$_2$	□$_3$

血沉（ESR） □0 未查，原因________________________________

采样日期：|__|__|__|__|年|__|__|月|__|__|日 正常值范围编号：|__|__|__|__|

检查项目	测量结果		结果判定		
				异常	
	实测值	单位	正常	无临床意义	有临床意义
血沉（ESR）		□1 mm/h □2其他：_____	□1	□2	□3

血沉（ESR） □0 未查，原因________________________________

采样日期：|__|__|__|__|年|__|__|月|__|__|日 正常值范围编号：|__|__|__|__|

检查项目	测量结果		结果判定		
				异常	
	实测值	单位	正常	无临床意义	有临床意义
血沉（ESR）		□1 mm/h □2其他：_____	□1	□2	□3

血沉（ESR） □0 未查，原因________________________________

采样日期：|__|__|__|__|年|__|__|月|__|__|日 正常值范围编号：|__|__|__|__|

检查项目	测量结果		结果判定		
				异常	
	实测值	单位	正常	无临床意义	有临床意义
血沉（ESR）		□1 mm/h □2其他：_____	□1	□2	□3

血沉（ESR） □0 未查，原因________________________________

采样日期：|__|__|__|__|年|__|__|月|__|__|日 正常值范围编号：|__|__|__|__|

检查项目	测量结果		结果判定		
				异常	
	实测值	单位	正常	无临床意义	有临床意义
血沉（ESR）		□1 mm/h □2其他：_____	□1	□2	□3

血沉（ESR） □0 未查，原因________________________________

采样日期：|__|__|__|__|年|__|__|月|__|__|日 正常值范围编号：|__|__|__|__|

检查项目	测量结果		结果判定		
				异常	
	实测值	单位	正常	无临床意义	有临床意义
血沉（ESR）		□1 mm/h □2其他：_____	□1	□2	□3

TNF-α	□0 未查，原因________________					
采样日期：|__|__|__|__|年|__|__|月|__|__|日			正常值范围编号：|__|__|__|__|			
检查项目	测量结果		结果判定			
TNF-α	实测值	单位	正常	异常		
				无临床意义	有临床意义	
		□1 pg/mL □2其他：_____	□1	□2	□3	

TNF-α	□0 未查，原因________________					
采样日期：|__|__|__|__|年|__|__|月|__|__|日			正常值范围编号：|__|__|__|__|			
检查项目	测量结果		结果判定			
TNF-α	实测值	单位	正常	异常		
				无临床意义	有临床意义	
		□1 pg/mL □2其他：_____	□1	□2	□3	

TNF-α	□0 未查，原因________________					
采样日期：|__|__|__|__|年|__|__|月|__|__|日			正常值范围编号：|__|__|__|__|			
检查项目	测量结果		结果判定			
TNF-α	实测值	单位	正常	异常		
				无临床意义	有临床意义	
		□1 pg/mL □2其他：_____	□1	□2	□3	

TNF-α	□0 未查，原因________________					
采样日期：|__|__|__|__|年|__|__|月|__|__|日			正常值范围编号：|__|__|__|__|			
检查项目	测量结果		结果判定			
TNF-α	实测值	单位	正常	异常		
				无临床意义	有临床意义	
		□1 pg/mL □2其他：_____	□1	□2	□3	

TNF-α	□0 未查，原因________________					
采样日期：|__|__|__|__|年|__|__|月|__|__|日			正常值范围编号：|__|__|__|__|			
检查项目	测量结果		结果判定			
TNF-α	实测值	单位	正常	异常		
				无临床意义	有临床意义	
		□1 pg/mL □2其他：_____	□1	□2	□3	

IL-6 □0 未查，原因________________

采样日期：|__|__|__|__|年|__|__|月|__|__|日 正常值范围编号：|__|__|__|__|

检查项目	测量结果		结果判定		
	实测值	单位	正常	异常	
				无临床意义	有临床意义
IL-6		□1 pg/mL □2其他：_____	□1	□2	□3

IL-6 □0 未查，原因________________

采样日期：|__|__|__|__|年|__|__|月|__|__|日 正常值范围编号：|__|__|__|__|

检查项目	测量结果		结果判定		
	实测值	单位	正常	异常	
				无临床意义	有临床意义
IL-6		□1 pg/mL □2其他：_____	□1	□2	□3

IL-6 □0 未查，原因________________

采样日期：|__|__|__|__|年|__|__|月|__|__|日 正常值范围编号：|__|__|__|__|

检查项目	测量结果		结果判定		
	实测值	单位	正常	异常	
				无临床意义	有临床意义
IL-6		□1 pg/mL □2其他：_____	□1	□2	□3

IL-6 □0 未查，原因________________

采样日期：|__|__|__|__|年|__|__|月|__|__|日 正常值范围编号：|__|__|__|__|

检查项目	测量结果		结果判定		
	实测值	单位	正常	异常	
				无临床意义	有临床意义
IL-6		□1 pg/mL □2其他：_____	□1	□2	□3

IL-6 □0 未查，原因________________

采样日期：|__|__|__|__|年|__|__|月|__|__|日 正常值范围编号：|__|__|__|__|

检查项目	测量结果		结果判定		
	实测值	单位	正常	异常	
				无临床意义	有临床意义
IL-6		□1 pg/mL □2其他：_____	□1	□2	□3

**尿或血清 HCG（仅适用于育龄女性）**	☐0 未查，原因________________<br>☐1 不适用（男性或不具有生育能力的女性）
采样日期：|__|__|__|__|年|__|__|月|__|__|日	正常值范围编号：|__|__|__|__|
采样类别：☐1 血液　☐2 尿液	
检查结果：☐1 阴性　☐2 阳性　☐3 其他：________________	
单位：☐1 不适用　☐2 mIU/mL　☐3 其他：________________	

**尿或血清 HCG（仅适用于育龄女性）**	☐0 未查，原因________________<br>☐1 不适用（男性或不具有生育能力的女性）
采样日期：|__|__|__|__|年|__|__|月|__|__|日	正常值范围编号：|__|__|__|__|
采样类别：☐1 血液　☐2 尿液	
检查结果：☐1 阴性　☐2 阳性　☐3 其他：________________	
单位：☐1 不适用　☐2 mIU/mL　☐3 其他：________________	

**尿或血清 HCG（仅适用于育龄女性）**	☐0 未查，原因________________<br>☐1 不适用（男性或不具有生育能力的女性）
采样日期：|__|__|__|__|年|__|__|月|__|__|日	正常值范围编号：|__|__|__|__|
采样类别：☐1 血液　☐2 尿液	
检查结果：☐1 阴性　☐2 阳性　☐3 其他：________________	
单位：☐1 不适用　☐2 mIU/mL　☐3 其他：________________	

**尿或血清 HCG（仅适用于育龄女性）**	☐0 未查，原因________________<br>☐1 不适用（男性或不具有生育能力的女性）
采样日期：|__|__|__|__|年|__|__|月|__|__|日	正常值范围编号：|__|__|__|__|
采样类别：☐1 血液　☐2 尿液	
检查结果：☐1 阴性　☐2 阳性　☐3 其他：________________	
单位：☐1 不适用　☐2 mIU/mL　☐3 其他：________________	

**12 导联心电图** □0 未查，原因______________________________
检查日期：|__|__|__|__|年|__|__|月|__|__|日
检查结果：□1 正常 □2 异常无临床意义 □3 异常有临床意义
若“异常”，请描述：______________________________

**12 导联心电图** □0 未查，原因______________________________
检查日期：|__|__|__|__|年|__|__|月|__|__|日
检查结果：□1 正常 □2 异常无临床意义 □3 异常有临床意义
若“异常”，请描述：______________________________

**12 导联心电图** □0 未查，原因______________________________
检查日期：|__|__|__|__|年|__|__|月|__|__|日
检查结果：□1 正常 □2 异常无临床意义 □3 异常有临床意义
若“异常”，请描述：______________________________

**12 导联心电图** □0 未查，原因______________________________
检查日期：|__|__|__|__|年|__|__|月|__|__|日
检查结果：□1 正常 □2 异常无临床意义 □3 异常有临床意义
若“异常”，请描述：______________________________

**12 导联心电图** □0 未查，原因______________________________
检查日期：|__|__|__|__|年|__|__|月|__|__|日
检查结果：□1 正常 □2 异常无临床意义 □3 异常有临床意义
若“异常”，请描述：______________________________

**12 导联心电图** □0 未查，原因______________________________
检查日期：|__|__|__|__|年|__|__|月|__|__|日
检查结果：□1 正常 □2 异常无临床意义 □3 异常有临床意义
若“异常”，请描述：______________________________

<table>
<tr><td colspan="2">新型冠状病毒核酸检测　　□$_{0}$ 未查，原因____</td></tr>
<tr><td>采样部位（可多选）</td><td>□$_{0}$ 上呼吸道　　□$_{1}$ 下呼吸道</td></tr>
<tr><td>上呼吸道</td><td>采样日期：|__|__|__|__|年|__|__|月|__|__|日
检查结果：□$_{1}$ 阴性 □$_{2}$ 阳性 □$_{3}$ 无法判断 □$_{4}$ 其他，请描述________</td></tr>
<tr><td>下呼吸道</td><td>采样日期：|__|__|__|__|年|__|__|月|__|__|日
检查结果：□$_{1}$ 阴性 □$_{2}$ 阳性 □$_{3}$ 无法判断 □$_{4}$ 其他，请描述________</td></tr>
<tr><td>结果判定</td><td>□$_{1}$ 阴性 □$_{2}$ 阳性 □$_{3}$ 无法判断 □$_{4}$ 其他，请描述________________</td></tr>
</table>

<table>
<tr><td colspan="2">新型冠状病毒核酸检测　　□$_{0}$ 未查，原因____</td></tr>
<tr><td>采样部位（可多选）</td><td>□$_{0}$ 上呼吸道　　□$_{1}$ 下呼吸道</td></tr>
<tr><td>上呼吸道</td><td>采样日期：|__|__|__|__|年|__|__|月|__|__|日
检查结果：□$_{1}$ 阴性 □$_{2}$ 阳性 □$_{3}$ 无法判断 □$_{4}$ 其他，请描述________</td></tr>
<tr><td>下呼吸道</td><td>采样日期：|__|__|__|__|年|__|__|月|__|__|日
检查结果：□$_{1}$ 阴性 □$_{2}$ 阳性 □$_{3}$ 无法判断 □$_{4}$ 其他，请描述________</td></tr>
<tr><td>结果判定</td><td>□$_{1}$ 阴性 □$_{2}$ 阳性 □$_{3}$ 无法判断 □$_{4}$ 其他，请描述________________</td></tr>
</table>

<table>
<tr><td colspan="2">新型冠状病毒核酸检测　　□$_{0}$ 未查，原因____</td></tr>
<tr><td>采样部位（可多选）</td><td>□$_{0}$ 上呼吸道　　□$_{1}$ 下呼吸道</td></tr>
<tr><td>上呼吸道</td><td>采样日期：|__|__|__|__|年|__|__|月|__|__|日
检查结果：□$_{1}$ 阴性 □$_{2}$ 阳性 □$_{3}$ 无法判断 □$_{4}$ 其他，请描述________</td></tr>
<tr><td>下呼吸道</td><td>采样日期：|__|__|__|__|年|__|__|月|__|__|日
检查结果：□$_{1}$ 阴性 □$_{2}$ 阳性 □$_{3}$ 无法判断 □$_{4}$ 其他，请描述________</td></tr>
<tr><td>结果判定</td><td>□$_{1}$ 阴性 □$_{2}$ 阳性 □$_{3}$ 无法判断 □$_{4}$ 其他，请描述________________</td></tr>
</table>

<table>
<tr><td colspan="2">新型冠状病毒核酸检测　　□$_{0}$ 未查，原因____</td></tr>
<tr><td>采样部位（可多选）</td><td>□$_{0}$ 上呼吸道　　□$_{1}$ 下呼吸道</td></tr>
<tr><td>上呼吸道</td><td>采样日期：|__|__|__|__|年|__|__|月|__|__|日
检查结果：□$_{1}$ 阴性 □$_{2}$ 阳性 □$_{3}$ 无法判断 □$_{4}$ 其他，请描述________</td></tr>
<tr><td>下呼吸道</td><td>采样日期：|__|__|__|__|年|__|__|月|__|__|日
检查结果：□$_{1}$ 阴性 □$_{2}$ 阳性 □$_{3}$ 无法判断 □$_{4}$ 其他，请描述________</td></tr>
<tr><td>结果判定</td><td>□$_{1}$ 阴性 □$_{2}$ 阳性 □$_{3}$ 无法判断 □$_{4}$ 其他，请描述________________</td></tr>
</table>

**指脉氧饱和度**（不吸氧情况下） □0 未查，原因____________________
检查日期：|__|__|__|__|年|__|__|月|__|__|日
实测值：______________%（静息状态）

**指脉氧饱和度**（不吸氧情况下） □0 未查，原因____________________
检查日期：|__|__|__|__|年|__|__|月|__|__|日
实测值：______________%（静息状态）

**指脉氧饱和度**（不吸氧情况下） □0 未查，原因____________________
检查日期：|__|__|__|__|年|__|__|月|__|__|日
实测值：______________%（静息状态）

**指脉氧饱和度**（不吸氧情况下） □0 未查，原因____________________
检查日期：|__|__|__|__|年|__|__|月|__|__|日
实测值：______________%（静息状态）

**指脉氧饱和度**（不吸氧情况下） □0 未查，原因____________________
检查日期：|__|__|__|__|年|__|__|月|__|__|日
实测值：______________%（静息状态）

**指脉氧饱和度**（不吸氧情况下） □0 未查，原因____________________
检查日期：|__|__|__|__|年|__|__|月|__|__|日
实测值：______________%（静息状态）

**指脉氧饱和度**（不吸氧情况下） □0 未查，原因____________________
检查日期：|__|__|__|__|年|__|__|月|__|__|日
实测值：______________%（静息状态）

**是否转成重型/危重型肺炎**	□$_0$ 未评价	评价日期|__|__|__|__|年|__|__|月|__|__|日
是否转成重型/危重型肺炎：	□$_0$ 否 □$_1$ 是，临床分型：	□$_1$ 重型 □$_2$ 危重型
转成重型/危重型肺炎日期：|__|__|__|__|年|__|__|月|__|__|日		

**是否转成重型/危重型肺炎**	□$_0$ 未评价	评价日期|__|__|__|__|年|__|__|月|__|__|日
是否转成重型/危重型肺炎：	□$_0$ 否 □$_1$ 是，临床分型：	□$_1$ 重型 □$_2$ 危重型
转成重型/危重型肺炎日期：|__|__|__|__|年|__|__|月|__|__|日		

**是否转成重型/危重型肺炎**	□$_0$ 未评价	评价日期|__|__|__|__|年|__|__|月|__|__|日
是否转成重型/危重型肺炎：	□$_0$ 否 □$_1$ 是，临床分型：	□$_1$ 重型 □$_2$ 危重型
转成重型/危重型肺炎日期：|__|__|__|__|年|__|__|月|__|__|日		

**是否转成重型/危重型肺炎**	□$_0$ 未评价	评价日期|__|__|__|__|年|__|__|月|__|__|日
是否转成重型/危重型肺炎：	□$_0$ 否 □$_1$ 是，临床分型：	□$_1$ 重型 □$_2$ 危重型
转成重型/危重型肺炎日期：|__|__|__|__|年|__|__|月|__|__|日		

**是否转成重型/危重型肺炎**	□$_0$ 未评价	评价日期|__|__|__|__|年|__|__|月|__|__|日
是否转成重型/危重型肺炎：	□$_0$ 否 □$_1$ 是，临床分型：	□$_1$ 重型 □$_2$ 危重型
转成重型/危重型肺炎日期：|__|__|__|__|年|__|__|月|__|__|日		

**是否转成重型/危重型肺炎**	□$_0$ 未评价	评价日期|__|__|__|__|年|__|__|月|__|__|日
是否转成重型/危重型肺炎：	□$_0$ 否 □$_1$ 是，临床分型：	□$_1$ 重型 □$_2$ 危重型
转成重型/危重型肺炎日期：|__|__|__|__|年|__|__|月|__|__|日		

**是否转成重型/危重型肺炎**	□$_0$ 未评价	评价日期|__|__|__|__|年|__|__|月|__|__|日
是否转成重型/危重型肺炎：	□$_0$ 否 □$_1$ 是，临床分型：	□$_1$ 重型 □$_2$ 危重型
转成重型/危重型肺炎日期：|__|__|__|__|年|__|__|月|__|__|日		

**是否转成重型/危重型肺炎**	□$_0$ 未评价	评价日期|__|__|__|__|年|__|__|月|__|__|日
是否转成重型/危重型肺炎：	□$_0$ 否 □$_1$ 是，临床分型：	□$_1$ 重型 □$_2$ 危重型
转成重型/危重型肺炎日期：|__|__|__|__|年|__|__|月|__|__|日		

**胸部 CT** ☐$_0$ 未查，原因______________ ☐$_1$ 不适用
检查日期：|__|__|__|__|年|__|__|月|__|__|日
检查结果：☐$_1$ 正常　☐$_2$ 异常无临床意义　☐$_3$ 异常有临床意义
若异常，请描述：______________
是否胸部 CT 病灶吸收较基线≥50%？　☐$_0$ 否　☐$_1$ 是　☐$_2$ 无法判断　☐$_3$ 不适用　☐$_4$ 其他，请描述：__________

**胸部 CT** ☐$_0$ 未查，原因______________ ☐$_1$ 不适用
检查日期：|__|__|__|__|年|__|__|月|__|__|日
检查结果：☐$_1$ 正常　☐$_2$ 异常无临床意义　☐$_3$ 异常有临床意义
若异常，请描述：______________
是否胸部 CT 病灶吸收较基线≥50%？　☐$_0$ 否　☐$_1$ 是　☐$_2$ 无法判断　☐$_3$ 不适用　☐$_4$ 其他，请描述：__________

**胸部 CT** ☐$_0$ 未查，原因______________ ☐$_1$ 不适用
检查日期：|__|__|__|__|年|__|__|月|__|__|日
检查结果：☐$_1$ 正常　☐$_2$ 异常无临床意义　☐$_3$ 异常有临床意义
若异常，请描述：______________
是否胸部 CT 病灶吸收较基线≥50%？　☐$_0$ 否　☐$_1$ 是　☐$_2$ 无法判断　☐$_3$ 不适用　☐$_4$ 其他，请描述：__________

动脉血气分析 □0 未查，原因________________

是否吸氧：□0 否 □1 是，吸氧浓度（FiO2）：__________%

采样日期：|__|__|__|__|年|__|__|月|__|__|日 正常值范围编号：|__|__|__|__|

检查项目	测量结果		结果判定			未查
	实测值	单位	正常	异常		
				无临床意义	有临床意义	
动脉血氧分压（PaO2）		□1 mmHg □2 其他：____	□1	□2	□3	□0
血氧饱和度（SaO2）		□1 % □2 其他：____	□1	□2	□3	□0

动脉血气分析 □0 未查，原因________________

是否吸氧：□0 否 □1 是，吸氧浓度（FiO2）：__________%

采样日期：|__|__|__|__|年|__|__|月|__|__|日 正常值范围编号：|__|__|__|__|

检查项目	测量结果		结果判定			未查
	实测值	单位	正常	异常		
				无临床意义	有临床意义	
动脉血氧分压（PaO2）		□1 mmHg □2 其他：____	□1	□2	□3	□0
血氧饱和度（SaO2）		□1 % □2 其他：____	□1	□2	□3	□0

动脉血气分析 □0 未查，原因________________

是否吸氧：□0 否 □1 是，吸氧浓度（FiO2）：__________%

采样日期：|__|__|__|__|年|__|__|月|__|__|日 正常值范围编号：|__|__|__|__|

检查项目	测量结果		结果判定			未查
	实测值	单位	正常	异常		
				无临床意义	有临床意义	
动脉血氧分压（PaO2）		□1 mmHg □2 其他：____	□1	□2	□3	□0
血氧饱和度（SaO2）		□1 % □2 其他：____	□1	□2	□3	□0

**临床状态**	□$_0$ 未评价，原因____
评价日期	|__|__|__|__|年|__|__|月|__|__|日
评价结果	□$_1$ 1级：出院<br>□$_2$ 2级：住院，不需要氧疗<br>□$_3$ 3级：住院，需要氧疗（非NIV/HFNC）<br>□$_4$ 4级：ICU/住院，需要无创机械通气（NIV）/高流量鼻导管氧疗（HFNC）<br>□$_5$ 5级：ICU，需要体外膜肺氧合（ECMO）和/或有创机械通气（IMV）<br>□$_6$ 6级：死亡

**临床状态**	□$_0$ 未评价，原因____
评价日期	|__|__|__|__|年|__|__|月|__|__|日
评价结果	□$_1$ 1级：出院<br>□$_2$ 2级：住院，不需要氧疗<br>□$_3$ 3级：住院，需要氧疗（非NIV/HFNC）<br>□$_4$ 4级：ICU/住院，需要无创机械通气（NIV）/高流量鼻导管氧疗（HFNC）<br>□$_5$ 5级：ICU，需要体外膜肺氧合（ECMO）和/或有创机械通气（IMV）<br>□$_6$ 6级：死亡

**临床状态**	□$_0$ 未评价，原因____
评价日期	|__|__|__|__|年|__|__|月|__|__|日
评价结果	□$_1$ 1级：出院<br>□$_2$ 2级：住院，不需要氧疗<br>□$_3$ 3级：住院，需要氧疗（非NIV/HFNC）<br>□$_4$ 4级：ICU/住院，需要无创机械通气（NIV）/高流量鼻导管氧疗（HFNC）<br>□$_5$ 5级：ICU，需要体外膜肺氧合（ECMO）和/或有创机械通气（IMV）<br>□$_6$ 6级：死亡

**临床状态**	□$_0$ 未评价，原因____
评价日期	|__|__|__|__|年|__|__|月|__|__|日
评价结果	□$_1$ 1级：出院<br>□$_2$ 2级：住院，不需要氧疗<br>□$_3$ 3级：住院，需要氧疗（非NIV/HFNC）<br>□$_4$ 4级：ICU/住院，需要无创机械通气（NIV）/高流量鼻导管氧疗（HFNC）<br>□$_5$ 5级：ICU，需要体外膜肺氧合（ECMO）和/或有创机械通气（IMV）<br>□$_6$ 6级：死亡

签署知情同意书	
签署知情同意书日期	|__|__|__|__|年|__|__|月|__|__|日
知情同意书版本号	____________________
签署知情同意书时适用方案版本号	|__|__|.|__|

签署知情同意书	
签署知情同意书日期	|__|__|__|__|年|__|__|月|__|__|日
知情同意书版本号	____________________
签署知情同意书时适用方案版本号	|__|__|.|__|

签署知情同意书	
签署知情同意书日期	|__|__|__|__|年|__|__|月|__|__|日
知情同意书版本号	____________________
签署知情同意书时适用方案版本号	|__|__|.|__|

签署知情同意书	
签署知情同意书日期	|__|__|__|__|年|__|__|月|__|__|日
知情同意书版本号	____________________
签署知情同意书时适用方案版本号	|__|__|.|__|

签署知情同意书	
签署知情同意书日期	|__|__|__|__|年|__|__|月|__|__|日
知情同意书版本号	____________________
签署知情同意书时适用方案版本号	|__|__|.|__|

硫酸羟氯喹治疗新型冠状病毒肺炎的一项随机、对照、开放的多中心研究

病例报告表

（Case Report Form）

中 心 编 号： |__|__|

受 试 者 筛 选 号 ： |__|__|__|__|__|

受试者姓名缩写： |__|__|__|__|

研究中心名称（正楷）： ______________________

研究医生姓名（正楷）： ______________________

版 本 号 及 日 期： 3.0 版/2020 年 2 月 25 日

适 用 临 床 分 型 ： 重型

保密声明

本文件包含的相关信息涉及商业机密和商业信息，这些商业信息术语保密内容或专有信息，若非法规要求，任何人不得擅自公开这些保密内容。任何接触到该文件的个人应当了解文件的专有性或机密性，并不得擅自公开文件。以上文件保密方面的限制条款将适用于未来提供给你的任何注明具有专有性或机密性的资料。

填表指南

1. 病例报告表须由授权人员负责观察记录，并对CRF中的每一页认真检查并签字。

2. 试验医师应熟悉试验方案，并掌握GCP原则。

3. 请用蓝色或黑色签字笔填写，字迹应清晰，易于辨认，英文最好用大写字母，例如：CASE REPORT FORM。

4. 在开放的方格中填写数字时，每一格填写一个数字；如果数字位数少于相应的格子数时请以零填充，不可出现空格。例如：56Kg，填入空格中为：| 0 | 5 | 6 |. | 0 | 0 |Kg。

5. 日期采用国际ISO8601日期格式：yyyymmdd。例如2007年12月21日写成| 2 |0 | 0 | 7 |年| 1 | 2 |月| 2 | 1 |日。

6. 表中凡有“□”的项目，请在符合的方框内打“×”，或填写相关数字，如果未做或数据无法获得，请在相应空格上填写ND（未完成）或NK（无法获知）；不适用填写NA。

7. 每项填写内容务必准确，不得随便涂改，禁止使用涂改液或以其他方式掩盖原始数据。如发现填写内容有误，请用单横线划去错误的记录，使原有记录仍可辨认，在右上方填写正确记录，并注明修改者的姓名缩写及修改日期。如：~~58.6~~ 56.8 ZH 2007-12-21。

8. 患者姓名拼音缩写四格应填满，两字姓名按每个字前两个字母填写，三字姓名按三字的首字母和第三个字的第二个字母填写；四字姓名填写每一个字的首字母。每位患者姓名的拼音缩写应在其CRF中统一。举例：张红| Z | H | H | 0 |，李淑明| L | S |M | I |，欧阳小惠| O | Y | X | H |。

9. 不要改变病例报告表的格式，如发现表中没有填写位置填写记录者希望记录的资料时，请联系负责的CRA。

10. 病例报告表填写完成后，主要研究者应检查病例报告表的每一页记录，并在病例报告表的研究者声明处签字。

本次访视日期：|__|__|__|__|年|__|__|月|__|__|日

**签署知情同意书**	
是否签署知情同意书	□$_0$ 否 □$_1$ 是
签署知情同意书日期	|__|__|__|__|年|__|__|月|__|__|日
知情同意书版本号	____________________
签署知情同意书时适用方案版本号	|__|__|.|__|

**确认入选/排除标准**	
受试者是否满足所有入选标准且不满足所有排除标准？ □$_1$ 是 □$_0$ 否，请填写。	
□$_1$ 不满足入选标准，入选标准编号（可多选）	□ 1 □ 2 □ 3 □ 4 □ 5 □ 6 □ 7<br>□ 其他，请描述：________________
□$_2$ 满足排除标准，排除标准编号（可多选）	□ 1 □ 2 □ 3 □ 4 □ 5 □ 6 □ 7<br>□ 8 □ 9 □ 其他，请描述：___________

**人口学资料**	
性 别	□$_1$ 男 □$_2$ 女
出 生 日 期	|__|__|__|__|年|__|__|月|__|__|日 如不能获得，请填写年龄：|__|__| 岁
民 族	□$_1$ 汉 □$_2$ 其他，请描述：____________________

**新型冠状病毒肺炎病史**	
首次发病日期	|__|__|__|__|年|__|__|月|__|__|日
发病前 14 天内有无相关病源接触史	□$_0$ 无 □$_1$ 有 □$_2$ 未知
接触类型（可多选）	□$_1$ 接触湖北地区<br>□$_2$ 接触新型冠状病毒感染者<br>□$_3$ 未知<br>□$_4$ 其他，请描述 ：____
既往有无进行抗新型冠状病毒肺炎治疗	□$_0$ 无 □$_1$ 有
首次核酸检测阳性报告日期	|__|__|__|__|年|__|__|月|__|__|日
入组时临床分型	□$_1$ 轻型 □$_2$ 普通型 □$_3$ 重型 □$_4$ 危重型

注：如进行抗新型冠状病毒肺炎治疗，请在“既往/合并药物治疗”和“既往/合并非药物治疗” 表中填写。

药物过敏史

受试者有无药物过敏史？

□0 无　　□1 有，请描述 ：____

____

既往/伴随疾病

受试者是否有除新型冠状病毒肺炎之外的既往/伴随疾病？　□0 否　□1 是，请填写下表。

编号	疾病名称	确诊日期	目前是否持续？	结束日期	是否接受治疗
1		|__|__|__|__|年 |__|__|月|__|__|日	□0 否 □1 是	|__|__|__|__|年 |__|__|月|__|__|日	□0 否 □1 是
2		|__|__|__|__|年 |__|__|月|__|__|日	□0 否 □1 是	|__|__|__|__|年 |__|__|月|__|__|日	□0 否 □1 是
3		|__|__|__|__|年 |__|__|月|__|__|日	□0 否 □1 是	|__|__|__|__|年 |__|__|月|__|__|日	□0 否 □1 是
4		|__|__|__|__|年 |__|__|月|__|__|日	□0 否 □1 是	|__|__|__|__|年 |__|__|月|__|__|日	□0 否 □1 是
5		|__|__|__|__|年 |__|__|月|__|__|日	□0 否 □1 是	|__|__|__|__|年 |__|__|月|__|__|日	□0 否 □1 是
6		|__|__|__|__|年 |__|__|月|__|__|日	□0 否 □1 是	|__|__|__|__|年 |__|__|月|__|__|日	□0 否 □1 是
7		|__|__|__|__|年 |__|__|月|__|__|日	□0 否 □1 是	|__|__|__|__|年 |__|__|月|__|__|日	□0 否 □1 是
8		|__|__|__|__|年 |__|__|月|__|__|日	□0 否 □1 是	|__|__|__|__|年 |__|__|月|__|__|日	□0 否 □1 是
9		|__|__|__|__|年 |__|__|月|__|__|日	□0 否 □1 是	|__|__|__|__|年 |__|__|月|__|__|日	□0 否 □1 是
10		|__|__|__|__|年 |__|__|月|__|__|日	□0 否 □1 是	|__|__|__|__|年 |__|__|月|__|__|日	□0 否 □1 是

生命体征　□0 未查，原因________________________________

检查日期	|__|__|__|__|年|__|__|月|__|__|日		
日最高体温	|__|__|.|__|℃	测量方式	□1 腋下　□2 口腔　□3 肛门<br>□4 耳朵　□5 未知　□6 其他，请描述______
呼吸频率	|__|__|次/分	脉搏	|__|__|__|次/分

血压	|__|__|__|/|__|__|__| mmHg（收缩压/舒张压）

**体格检查** □0 未查，原因________________________					
检查日期：|__|__|__|__|年|__|__|月|__|__|日					
**检查部位**	**检查结果**<br>**1=正常**<br>**2=异常无临床意义**<br>**3=异常有临床意义**<br>**0=未查**				**异常请描述**
整体外观	□1	□2	□3	□0	
头	□1	□2	□3	□0	
眼	□1	□2	□3	□0	
耳	□1	□2	□3	□0	
鼻	□1	□2	□3	□0	
喉	□1	□2	□3	□0	
颈部及甲状腺	□1	□2	□3	□0	
皮肤	□1	□2	□3	□0	
心血管系统	□1	□2	□3	□0	
胸部及肺	□1	□2	□3	□0	
腹部	□1	□2	□3	□0	
神经系统	□1	□2	□3	□0	
骨骼肌系统	□1	□2	□3	□0	
淋巴系统	□1	□2	□3	□0	

**有无呼吸道症状** □0 未查，原因________________________
检查日期：|__|__|__|__|年|__|__|月|__|__|日
受试者有无呼吸道症状：□0 无 □1 有，请描述：________________________<br>____

血常规 □0 未查，原因______________________________

采样日期：|__|__|__|__|年|__|__|月|__|__|日 正常值范围编号：|__|__|__|__|__|

检查项目	测量结果		结果判定			未查
	实测值	单位	正常	异常		
				无临床意义	有临床意义	
红细胞计数（RBC）		□1 10^{12}/L □2 其他：_____	□1	□2	□3	□0
白细胞计数（WBC）		□1 10^9/L □2 其他：_____	□1	□2	□3	□0
血红蛋白（Hb）		□1 g/L □2 其他：_____	□1	□2	□3	□0
血小板计数（PLT）		□1 10^9/L □2 其他：_____	□1	□2	□3	□0
中性粒细胞计数（ANC）		□1 10^9/L □2 其他：_____	□1	□2	□3	□0
淋巴细胞计数（LY）		□1 10^9/L □2 其他：_____	□1	□2	□3	□0
单核细胞计数（MO）		□1 10^9/L □2 其他：_____	□1	□2	□3	□0
嗜酸性粒细胞计数（EO）		□1 10^9/L □2 其他：_____	□1	□2	□3	□0
嗜碱性粒细胞计数（BA）		□1 10^9/L □2 其他：_____	□1	□2	□3	□0

血生化 □0 未查，原因______________________________

采样日期|__|__|__|__|年|__|__|月|__|__|日 正常值范围编号：|__|__|__|__|__|

检查项目	测量结果		结果判定			未查
	实测值	单位	正常	异常		
				无临床意义	有临床意义	
谷丙转氨酶（ALT）		□1 U/L □2 其他：_____	□1	□2	□3	□0
谷草转氨酶（AST）		□1 U/L □2 其他：_____	□1	□2	□3	□0
总胆红素（TBIL）		□1 μmol/L □2 其他：_____	□1	□2	□3	□0
白蛋白（ALB）		□1 g/L □2 其他：_____	□1	□2	□3	□0
γ-谷氨酰转肽酶（GGT）		□1 U/L □2 其他：_____	□1	□2	□3	□0
乳酸脱氢酶（LDH）		□1 U/L □2 其他：_____	□1	□2	□3	□0
肌酸激酶（CK）		□1 U/L □2 其他：_____	□1	□2	□3	□0
肌酸激酶同工酶（CK-MB）		□1 U/L □2 其他：_____	□1	□2	□3	□0
血尿素氮（BUN）		□1 mmol/L □2 其他：_____	□1	□2	□3	□0
尿素（Urea）		□1 mmol/L □2 其他：_____	□1	□2	□3	□0
血肌酐（CR）		□1 μmol/L □2 其他：_____	□1	□2	□3	□0

尿常规 □0 未查，原因________________

采样日期：|__|__|__|__|__|年|__|__|月|__|__|日 正常值范围编号：|__|__|__|__|__|

检查项目	测量结果		结果判定			未查
	实测值	单位	正常	异常		
				无临床意义	有临床意义	
尿蛋白		□1 不适用 □2 其他：_____	□1	□2	□3	□0
尿葡萄糖		□1 mmol/L □2 其他：_____	□1	□2	□3	□0
尿 PH 值		□1 不适用 □2 其他：_____	□1	□2	□3	□0
尿红细胞		□1 不适用 □2 其他：_____	□1	□2	□3	□0
尿白细胞		□1 不适用 □2 其他：_____	□1	□2	□3	□0
尿酮体		□1 不适用 □2 其他：_____	□1	□2	□3	□0

凝血功能 □0 未查，原因________________

采样日期：|__|__|__|__|__|年|__|__|月|__|__|日 正常值范围编号：|__|__|__|__|__|

检查项目	测量结果		结果判定			未查
	实测值	单位	正常	异常		
				无临床意义	有临床意义	
凝血酶原时间（PT）		□1 S □2 其他：_____	□1	□2	□3	□0
活化部分凝血活酶时间（APTT）		□1 S □2 其他：_____	□1	□2	□3	□0
凝血酶时间（TT）		□1 S □2 其他：_____	□1	□2	□3	□0
血浆纤维蛋白原（Fbg）		□1 g/L □2 其他：_____	□1	□2	□3	□0
国际标准化比率（INR）		□1 不适用 □2 其他：_____	□1	□2	□3	□0

C 反应蛋白（CRP） □0 未查，原因________________

采样日期：|__|__|__|__|__|年|__|__|月|__|__|日 正常值范围编号：|__|__|__|__|__|

检查项目	测量结果		结果判定		
	实测值	单位	正常	异常	
				无临床意义	有临床意义
C 反应蛋白（CRP）		□1 mg/L □2 其他：_____	□1	□2	□3

血沉（ESR） □0 未查，原因________________

采样日期：|__|__|__|__|__|年|__|__|月|__|__|日 正常值范围编号：|__|__|__|__|__|

检查项目	测量结果		结果判定	
	实测值	单位	正常	异常

				无临床意义	有临床意义
血沉（ESR）		□1 mm/h □2其他：_____	□1	□2	□3

TNF-α □0 未查，原因________________

采样日期：\|__\|__\|__\|__\|年\|__\|__\|月\|__\|__\|日 正常值范围编号：\|__\|__\|__\|__\|

检查项目	测量结果		结果判定		
	实测值	单位	正常	异常	
				无临床意义	有临床意义
TNF-α		□1 pg/mL □2其他：_____	□1	□2	□3

IL-6 □0 未查，原因________________

采样日期：\|__\|__\|__\|__\|年\|__\|__\|月\|__\|__\|日 正常值范围编号：\|__\|__\|__\|__\|

检查项目	测量结果		结果判定		
	实测值	单位	正常	异常	
				无临床意义	有临床意义
IL-6		□1 pg/mL □2其他：_____	□1	□2	□3

尿或血清 HCG（仅适用于育龄女性） □0 未查，原因________________
□1 不适用（男性或不具有生育能力的女性）

采样日期：\|__\|__\|__\|__\|年\|__\|__\|月\|__\|__\|日 正常值范围编号：\|__\|__\|__\|__\|

采样类别：□1 血液 □2 尿液

检查结果：□1 阴性 □2 阳性 □3 其他：________________

单位： □1 不适用 □2 mIU/mL □3 其他：________________

12 导联心电图 □0 未查，原因________________

检查日期：\|__\|__\|__\|__\|年\|__\|__\|月\|__\|__\|日

检查结果：□1 正常 □2 异常无临床意义 □3 异常有临床意义

若“异常”，请描述：________________________________

____

胸部 CT □0 未查，原因________________

检查日期：\|__\|__\|__\|__\|年\|__\|__\|月\|__\|__\|日

检查结果：□1 正常 □2 异常无临床意义 □3 异常有临床意义

若异常，请描述：____

____

新型冠状病毒核酸检测	□0 未查，原因________________
采样部位（可多选）	□0 上呼吸道　□1 下呼吸道
上呼吸道	采样日期：|__|__|__|__|年|__|__|月|__|__|日<br>检查结果：□1 阴性 □2 阳性 □3 无法判断 □4 其他，请描述________
下呼吸道	采样日期：|__|__|__|__|年|__|__|月|__|__|日<br>检查结果：□1 阴性 □2 阳性 □3 无法判断 □4 其他，请描述________
结果判定	□1 阴性 □2 阳性 □3 无法判断 □4 其他，请描述________________

指脉氧饱和度（不吸氧情况下） □0 未查，原因________________
检查日期：|__|__|__|__|年|__|__|月|__|__|日
实测值：______________%（静息状态）

动脉血气分析 □0 未查，原因________________						
是否吸氧：□0 否　□1 是，吸氧浓度（FiO2）：______________%						
采样日期：|__|__|__|__|年|__|__|月|__|__|日　正常值范围编号：|__|__|__|__|						
检查项目	测量结果		结果判定			未查
				异常		
	实测值	单位	正常	无临床意义	有临床意义	
动脉血氧分压（PaO2）		□1 mmHg　□2 其他：____	□1	□2	□3	□0
血氧饱和度（SaO2）		□1 %　□2 其他：____	□1	□2	□3	□0

临床状态	□0 未评价，原因________________
评价日期	|__|__|__|__|年|__|__|月|__|__|日
评价结果	□1 1级：出院<br>□2 2级：住院，不需要氧疗<br>□3 3级：住院，需要氧疗（非NIV/HFNC）<br>□4 4级：ICU/住院，需要无创机械通气（NIV）/高流量鼻导管氧疗（HFNC）<br>□5 5级：ICU，需要体外膜肺氧合（ECMO）和/或有创机械通气（IMV）<br>□6 6级：死亡

**筛选结果**
受试者是否筛选成功？<br>□0 否，筛选失败的主要原因：________________<br>□1 是，请进行随机化。
随机日期：|__|__|__|__|年|__|__|月|__|__|日
随机编号：________________
随机分组：□1 试验组　　　　　□2 对照组

自签署知情同意书以来，如有不良事件发生，请填写“不良事件”表；如有合并药物治疗，请填写“既往/合并药物治疗”表；如有合并非药物治疗，请填写“既往/合并非药物治疗”表；如有计划外检查，请填写“计划外检查”表。；如有 IMV/NIV、ECMO、HFNC、氧疗，请填写在“IMV/NIV、ECMO、 HFNC、氧疗”表中。

本次访视日期：|__|__|__|__|年|__|__|月|__|__|日

**生命体征** □0 未查，原因________			
检查日期	|__|__|__|__|年|__|__|月|__|__|日		
日最高体温	|__|__|.|__|℃	测量方式	□1 腋下 □2 口腔 □3 肛门<br>□4 耳朵 □5 未知 □6 其他，请描述______
呼吸频率	|__|__|次/分	脉搏	|__|__|__|次/分
血压	|__|__|__|/|__|__|__| mmHg（收缩压/舒张压）		

**有无呼吸道症状** □0 未查，原因________
检查日期：|__|__|__|__|年|__|__|月|__|__|日
受试者有无呼吸道症状：□0 无 □1 有，请描述：________

**血常规** □0 未查，原因________						
采样日期：|__|__|__|__|年|__|__|月|__|__|日			正常值范围编号：|__|__|__|__|			
检查项目	测量结果		结果判定			未查
	实测值	单位	正常	异常：无临床意义	异常：有临床意义	
红细胞计数（RBC）		□1 10^{12}/L □2 其他：____	□1	□2	□3	□0
白细胞计数（WBC）		□1 10^9/L □2 其他：____	□1	□2	□3	□0
血红蛋白（Hb）		□1 g/L □2 其他：____	□1	□2	□3	□0
血小板计数（PLT）		□1 10^9/L □2 其他：____	□1	□2	□3	□0
中性粒细胞计数（ANC）		□1 10^9/L □2 其他：____	□1	□2	□3	□0
淋巴细胞计数（LY）		□1 10^9/L □2 其他：____	□1	□2	□3	□0
单核细胞计数（MO）		□1 10^9/L □2 其他：____	□1	□2	□3	□0
嗜酸性粒细胞计数（EO）		□1 10^9/L □2 其他：____	□1	□2	□3	□0
嗜碱性粒细胞计数（BA）		□1 10^9/L □2 其他：____	□1	□2	□3	□0

**血生化** □0 未查，原因________						
采样日期|__|__|__|__|年|__|__|月|__|__|日			正常值范围编号：|__|__|__|__|			
检查项目	测量结果		结果判定			未查
	实测值	单位	正常	异常：无临床意义	异常：有临床意义	

谷丙转氨酶（ALT）		□1 U/L □2其他：_____	□1	□2	□3	□0
谷草转氨酶（AST）		□1 U/L □2其他：_____	□1	□2	□3	□0
总胆红素（TBIL）		□1 μmol/L □2其他：_____	□1	□2	□3	□0
白蛋白（ALB）		□1 g/L □2其他：_____	□1	□2	□3	□0
γ-谷氨酰转肽酶（GGT）		□1 U/L □2其他：_____	□1	□2	□3	□0
乳酸脱氢酶（LDH）		□1 U/L □2其他：_____	□1	□2	□3	□0
肌酸激酶（CK）		□1 U/L □2其他：_____	□1	□2	□3	□0
肌酸激酶同工酶（CK-MB）		□1 U/L □2其他：_____	□1	□2	□3	□0
血尿素氮（BUN）		□1 mmol/L □2其他：_____	□1	□2	□3	□0
尿素（Urea）		□1 mmol/L □2其他：_____	□1	□2	□3	□0
血肌酐（CR）		□1 μmol/L □2其他：_____	□1	□2	□3	□0

凝血功能 □0未查，原因____

采样日期：|__|__|__|__|年|__|__|月|__|__|日 正常值范围编号：|__|__|__|__|

检查项目	测量结果		结果判定			未查
	实测值	单位	正常	异常		
				无临床意义	有临床意义	
凝血酶原时间（PT）		□1 S □2其他：_____	□1	□2	□3	□0
活化部分凝血活酶时间（APTT）		□1 S □2其他：_____	□1	□2	□3	□0
凝血酶时间（TT）		□1 S □2其他：_____	□1	□2	□3	□0
血浆纤维蛋白原（Fbg）		□1 g/L □2其他：_____	□1	□2	□3	□0
国际标准化比率（INR）		□1不适用 □2其他：_____	□1	□2	□3	□0

C 反应蛋白（CRP） □0未查，原因____

采样日期：|__|__|__|__|年|__|__|月|__|__|日 正常值范围编号：|__|__|__|__|

检查项目	测量结果		结果判定		
	实测值	单位	正常	异常	
				无临床意义	有临床意义
C 反应蛋白（CRP）		□1 mg/L □2其他：_____	□1	□2	□3

血沉（ESR） □0未查，原因____

采样日期：|__|__|__|__|年|__|__|月|__|__|日 正常值范围编号：|__|__|__|__|

检查项目	测量结果		结果判定		
				异常	
	实测值	单位	正常	无临床意义	有临床意义
血沉（ESR）		□1 mm/h □2其他：_____	□1	□2	□3

TNF-α □0 未查，原因________________________________

采样日期：\|__\|__\|__\|__\|年\|__\|__\|月\|__\|__\|日 正常值范围编号：\|__\|__\|__\|__\|

检查项目	测量结果		结果判定		
				异常	
	实测值	单位	正常	无临床意义	有临床意义
TNF-α		□1 pg/mL □2其他：_____	□1	□2	□3

IL-6 □0 未查，原因________________________________

采样日期：\|__\|__\|__\|__\|年\|__\|__\|月\|__\|__\|日 正常值范围编号：\|__\|__\|__\|__\|

检查项目	测量结果		结果判定		
				异常	
	实测值	单位	正常	无临床意义	有临床意义
IL-6		□1 pg/mL □2其他：_____	□1	□2	□3

**新型冠状病毒核酸检测**	□0 未查，原因________________________
采样部位（可多选）	□0 上呼吸道 □1 下呼吸道
上呼吸道	采样日期：|__|__|__|__|年|__|__|月|__|__|日<br>检查结果：□1 阴性 □2 阳性 □3 无法判断 □4 其他，请描述_________
下呼吸道	采样日期：|__|__|__|__|年|__|__|月|__|__|日<br>检查结果：□1 阴性 □2 阳性 □3 无法判断 □4 其他，请描述_________
结果判定	□1 阴性 □2 阳性 □3 无法判断 □4 其他，请描述__________________

指脉氧饱和度（不吸氧情况下） □0 未查，原因________________________

检查日期：\|__\|__\|__\|__\|年\|__\|__\|月\|__\|__\|日

实测值：_______________%（静息状态）

动脉血气分析 □0 未查，原因________________________________
□1 不适用

是否吸氧：□0 否 □1 是，吸氧浓度（FiO2）：_______________%

采样日期：\|__\|__\|__\|__\|年\|__\|__\|月\|__\|__\|日 正常值范围编号：\|__\|__\|__\|__\|

检查项目	测量结果		结果判定			未查
	实测值	单位	正常	异常		
				无临床意义	有临床意义	
动脉血氧分压（PaO2）		□1 mmHg □2 其他：_____	□1	□2	□3	□0
血氧饱和度（SaO2）		□1 % □2 其他：_____	□1	□2	□3	□0

自上次访视以来，受试者如有不良事件发生，请填写“不良事件”表；如有合并药物治疗，请填写“既往/合并药物治疗”表；如有合并非药物治疗，请填写“既往/合并非药物治疗”表；如有计划外检查，请填写“计划外检查”表。如有 IMV/NIV、ECMO、HFNC、氧疗，请填写在“IMV/NIV、ECMO、HFNC、氧疗”表中。

本次访视日期：|__|__|__|__|年|__|__|月|__|__|日

**生命体征** □0 未查，原因________________			
检查日期	|__|__|__|__|年|__|__|月|__|__|日		
日最高体温	|__|__|.|__|℃	测量方式	□1 腋下 □2 口腔 □3 肛门<br>□4 耳朵 □5 未知 □6 其他，请描述______
呼吸频率	|__|__|次/分	脉搏	|__|__|__|次/分
血压	|__|__|__|/|__|__|__| mmHg（收缩压/舒张压）		

**有无呼吸道症状** □0 未查，原因________________
检查日期：|__|__|__|__|年|__|__|月|__|__|日
受试者有无呼吸道症状：□0 无 □1 有，请描述：________________<br>________________

血常规 □0 未查，原因________________

采样日期：|__|__|__|__|年|__|__|月|__|__|日　　正常值范围编号：|__|__|__|__|

检查项目	测量结果		结果判定			未查
	实测值	单位	正常	异常：无临床意义	异常：有临床意义	
红细胞计数（RBC）		□1 10^{12}/L □2 其他：____	□1	□2	□3	□0
白细胞计数（WBC）		□1 10^9/L □2 其他：____	□1	□2	□3	□0
血红蛋白（Hb）		□1 g/L □2 其他：____	□1	□2	□3	□0
血小板计数（PLT）		□1 10^9/L □2 其他：____	□1	□2	□3	□0
中性粒细胞计数（ANC）		□1 10^9/L □2 其他：____	□1	□2	□3	□0
淋巴细胞计数（LY）		□1 10^9/L □2 其他：____	□1	□2	□3	□0
单核细胞计数（MO）		□1 10^9/L □2 其他：____	□1	□2	□3	□0
嗜酸性粒细胞计数（EO）		□1 10^9/L □2 其他：____	□1	□2	□3	□0
嗜碱性粒细胞计数（BA）		□1 10^9/L □2 其他：____	□1	□2	□3	□0

血生化 □0 未查，原因________________

采样日期|__|__|__|__|年|__|__|月|__|__|日　　正常值范围编号：|__|__|__|__|

检查项目	测量结果		结果判定			未查
	实测值	单位	正常	异常：无临床意义	异常：有临床意义	

谷丙转氨酶（ALT）		□1 U/L □2 其他：_____	□1	□2	□3	□0
谷草转氨酶（AST）		□1 U/L □2 其他：_____	□1	□2	□3	□0
总胆红素（TBIL）		□1 μmol/L □2 其他：_____	□1	□2	□3	□0
白蛋白（ALB）		□1 g/L □2 其他：_____	□1	□2	□3	□0
γ-谷氨酰转肽酶（GGT）		□1 U/L □2 其他：_____	□1	□2	□3	□0
乳酸脱氢酶（LDH）		□1 U/L □2 其他：_____	□1	□2	□3	□0
肌酸激酶（CK）		□1 U/L □2 其他：_____	□1	□2	□3	□0
肌酸激酶同工酶（CK-MB）		□1 U/L □2 其他：_____	□1	□2	□3	□0
血尿素氮（BUN）		□1 mmol/L □2 其他：_____	□1	□2	□3	□0
尿素（Urea）		□1 mmol/L □2 其他：_____	□1	□2	□3	□0
血肌酐（CR）		□1 μmol/L □2 其他：_____	□1	□2	□3	□0

凝血功能 □0 未查，原因________________________________

采样日期：|__|__|__|__|年|__|__|月|__|__|日 正常值范围编号：|__|__|__|__|

检查项目	测量结果		结果判定			未查
	实测值	单位	正常	异常		
				无临床意义	有临床意义	
凝血酶原时间（PT）		□1 S □2 其他：_____	□1	□2	□3	□0
活化部分凝血活酶时间（APTT）		□1 S □2 其他：_____	□1	□2	□3	□0
凝血酶时间（TT）		□1 S □2 其他：_____	□1	□2	□3	□0
血浆纤维蛋白原（Fbg）		□1 g/L □2 其他：_____	□1	□2	□3	□0
国际标准化比率（INR）		□1 不适用 □2 其他：_____	□1	□2	□3	□0

C 反应蛋白（CRP） □0 未查，原因________________________________

采样日期：|__|__|__|__|年|__|__|月|__|__|日 正常值范围编号：|__|__|__|__|

检查项目	测量结果		结果判定		
	实测值	单位	正常	异常	
				无临床意义	有临床意义
C 反应蛋白（CRP）		□1 mg/L □2 其他：_____	□1	□2	□3

血沉（ESR） □0 未查，原因________________________________

采样日期：|__|__|__|__|年|__|__|月|__|__|日 正常值范围编号：|__|__|__|__|

检查项目	测量结果		结果判定		
				异常	
血沉（ESR）	实测值	单位	正常	无临床意义	有临床意义
		□1 mm/h　□2其他：_____	□1	□2	□3

TNF-α　　□0 未查，原因________________________________

采样日期：|__|__|__|__|年|__|__|月|__|__|日　　正常值范围编号：|__|__|__|__|

检查项目	测量结果		结果判定		
				异常	
TNF-α	实测值	单位	正常	无临床意义	有临床意义
		□1 pg/mL　□2其他：_____	□1	□2	□3

IL-6　　□0 未查，原因________________________________

采样日期：|__|__|__|__|年|__|__|月|__|__|日　　正常值范围编号：|__|__|__|__|

检查项目	测量结果		结果判定		
				异常	
IL-6	实测值	单位	正常	无临床意义	有临床意义
		□1 pg/mL　□2其他：_____	□1	□2	□3

**新型冠状病毒核酸检测**	□0 未查，原因________________________
采样部位（可多选）	□0 上呼吸道　　□1 下呼吸道
上呼吸道	采样日期：|__|__|__|__|年|__|__|月|__|__|日<br>检查结果：□1 阴性　□2 阳性　□3 无法判断　□4 其他，请描述_________
下呼吸道	采样日期：|__|__|__|__|年|__|__|月|__|__|日<br>检查结果：□1 阴性　□2 阳性　□3 无法判断　□4 其他，请描述_________
结果判定	□1 阴性　□2 阳性　□3 无法判断　□4 其他，请描述__________________

指脉氧饱和度（不吸氧情况下）　□0 未查，原因________________________

检查日期：|__|__|__|__|年|__|__|月|__|__|日

实测值：_______________%（静息状态）

动脉血气分析　□0 未查，原因________________________________
□1 不适用

是否吸氧：□0 否　　□1 是，吸氧浓度（FiO2）：_______________%

采样日期：|__|__|__|__|年|__|__|月|__|__|日　　正常值范围编号：|__|__|__|__|

检查项目	测量结果		结果判定			未查
	实测值	单位	正常	异常		
				无临床意义	有临床意义	
动脉血氧分压（PaO2）		□1 mmHg □2 其他：____	□1	□2	□3	□0
血氧饱和度（SaO2）		□1 % □2 其他：____	□1	□2	□3	□0

**临床状态**	□0 未评价，原因________________
评价日期	|__|__|__|__|年|__|__|月|__|__|日
评价结果	□1 1级：出院<br>□2 2级：住院，不需要氧疗<br>□3 3级：住院，需要氧疗（非NIV/HFNC）<br>□4 4级：ICU/住院，需要无创机械通气（NIV）/高流量鼻导管氧疗（HFNC）<br>□5 5级：ICU，需要体外膜肺氧合（ECMO）和/或有创机械通气（IMV）<br>□6 6级：死亡

自上次访视以来，受试者如有不良事件发生，请填写“不良事件”表；如有合并药物治疗，请填写“既往/合并药物治疗”表；如有合并非药物治疗，请填写“既往/合并非药物治疗”表；如有计划外检查，请填写“计划外检查”表。如有 IMV/NIV、ECMO、HFNC、氧疗，请填写在“IMV/NIV、ECMO、HFNC、氧疗”表中。

本次访视日期：|__|__|__|__|年|__|__|月|__|__|日

**生命体征**　　□0 未查，原因________________			
检查日期	|__|__|__|__|年|__|__|月|__|__|日		
日最高体温	|__|__|.|__|℃	测量方式	□1 腋下　□2 口腔　□3 肛门<br>□4 耳朵　□5 未知　□6 其他，请描述______
呼吸频率	|__|__|次/分	脉搏	|__|__|__|次/分
血压	|__|__|__|/|__|__|__| mmHg（收缩压/舒张压）		

**有无呼吸道症状**　　□0 未查，原因________________
检查日期：|__|__|__|__|年|__|__|月|__|__|日
受试者有无呼吸道症状：□0 无　　□1 有，请描述：________________<br>________________

血常规　　□0 未查，原因________________

采样日期：|__|__|__|__|年|__|__|月|__|__|日　　正常值范围编号：|__|__|__|__|

检查项目	测量结果		结果判定			未查
	实测值	单位	正常	异常		
				无临床意义	有临床意义	
红细胞计数（RBC）		□1 10^{12}/L　□2 其他：____	□1	□2	□3	□0
白细胞计数（WBC）		□1 10^9/L　□2 其他：____	□1	□2	□3	□0
血红蛋白（Hb）		□1 g/L　□2 其他：____	□1	□2	□3	□0
血小板计数（PLT）		□1 10^9/L　□2 其他：____	□1	□2	□3	□0
中性粒细胞计数（ANC）		□1 10^9/L　□2 其他：____	□1	□2	□3	□0
淋巴细胞计数（LY）		□1 10^9/L　□2 其他：____	□1	□2	□3	□0
单核细胞计数（MO）		□1 10^9/L　□2 其他：____	□1	□2	□3	□0
嗜酸性粒细胞计数（EO）		□1 10^9/L　□2 其他：____	□1	□2	□3	□0
嗜碱性粒细胞计数（BA）		□1 10^9/L　□2 其他：____	□1	□2	□3	□0

血生化　　□0 未查，原因________________

采样日期|__|__|__|__|年|__|__|月|__|__|日　　正常值范围编号：|__|__|__|__|

检查项目	测量结果		结果判定			未查
	实测值	单位	正常	异常		
				无临床意义	有临床意义	

谷丙转氨酶（ALT）		□1 U/L □2 其他：_____	□1	□2	□3	□0
谷草转氨酶（AST）		□1 U/L □2 其他：_____	□1	□2	□3	□0
总胆红素（TBIL）		□1 μmol/L □2 其他：_____	□1	□2	□3	□0
白蛋白（ALB）		□1 g/L □2 其他：_____	□1	□2	□3	□0
γ-谷氨酰转肽酶（GGT）		□1 U/L □2 其他：_____	□1	□2	□3	□0
乳酸脱氢酶（LDH）		□1 U/L □2 其他：_____	□1	□2	□3	□0
肌酸激酶（CK）		□1 U/L □2 其他：_____	□1	□2	□3	□0
肌酸激酶同工酶（CK-MB）		□1 U/L □2 其他：_____	□1	□2	□3	□0
血尿素氮（BUN）		□1 mmol/L □2 其他：_____	□1	□2	□3	□0
尿素（Urea）		□1 mmol/L □2 其他：_____	□1	□2	□3	□0
血肌酐（CR）		□1 μmol/L □2 其他：_____	□1	□2	□3	□0

凝血功能 □0 未查，原因________________

采样日期：|__|__|__|__|年|__|__|月|__|__|日 正常值范围编号：|__|__|__|__|

检查项目	测量结果		结果判定			未查
	实测值	单位	正常	异常		
				无临床意义	有临床意义	
凝血酶原时间（PT）		□1 S □2 其他：_____	□1	□2	□3	□0
活化部分凝血活酶时间（APTT）		□1 S □2 其他：_____	□1	□2	□3	□0
凝血酶时间（TT）		□1 S □2 其他：_____	□1	□2	□3	□0
血浆纤维蛋白原（Fbg）		□1 g/L □2 其他：_____	□1	□2	□3	□0
国际标准化比率（INR）		□1 不适用 □2 其他：_____	□1	□2	□3	□0

C 反应蛋白（CRP） □0 未查，原因________________

采样日期：|__|__|__|__|年|__|__|月|__|__|日 正常值范围编号：|__|__|__|__|

检查项目	测量结果		结果判定		
	实测值	单位	正常	异常	
				无临床意义	有临床意义
C 反应蛋白（CRP）		□1 mg/L □2 其他：_____	□1	□2	□3

血沉（ESR） □0 未查，原因________________

采样日期：|__|__|__|__|年|__|__|月|__|__|日 正常值范围编号：|__|__|__|__|

检查项目	测量结果		结果判定		
血沉（ESR）	实测值	单位	正常	异常	
				无临床意义	有临床意义
		□1 mm/h □2其他：_____	□1	□2	□3

TNF-α □0 未查，原因____

采样日期：|__|__|__|__|年|__|__|月|__|__|日 正常值范围编号：|__|__|__|__|

检查项目	测量结果		结果判定		
TNF-α	实测值	单位	正常	异常	
				无临床意义	有临床意义
		□1 pg/mL □2其他：_____	□1	□2	□3

IL-6 □0 未查，原因____

采样日期：|__|__|__|__|年|__|__|月|__|__|日 正常值范围编号：|__|__|__|__|

检查项目	测量结果		结果判定		
IL-6	实测值	单位	正常	异常	
				无临床意义	有临床意义
		□1 pg/mL □2其他：_____	□1	□2	□3

新型冠状病毒核酸检测 □0 未查，原因______________________________

采样部位（可多选）	□0 上呼吸道 □1 下呼吸道
上呼吸道	采样日期：|__|__|__|__|年|__|__|月|__|__|日<br>检查结果：□1 阴性 □2 阳性 □3 无法判断 □4 其他，请描述_________
下呼吸道	采样日期：|__|__|__|__|年|__|__|月|__|__|日<br>检查结果：□1 阴性 □2 阳性 □3 无法判断 □4 其他，请描述_________
结果判定	□1 阴性 □2 阳性 □3 无法判断 □4 其他，请描述__________________

指脉氧饱和度（不吸氧情况下） □0 未查，原因______________________________

检查日期：|__|__|__|__|年|__|__|月|__|__|日

实测值：_______________%（静息状态）

动脉血气分析 □0 未查，原因____
□1 不适用

是否吸氧：□0 否 □1 是，吸氧浓度（FiO2）：_______________%

采样日期：|__|__|__|__|年|__|__|月|__|__|日 正常值范围编号：|__|__|__|__|

检查项目	测量结果		结果判定			未查
	实测值	单位	正常	异常		
				无临床意义	有临床意义	
动脉血氧分压（PaO2）		□1 mmHg　□2 其他：_____	□1	□2	□3	□0
血氧饱和度（SaO2）		□1 %　□2 其他：_____	□1	□2	□3	□0

自上次访视以来，受试者如有不良事件发生，请填写“不良事件”表；如有合并药物治疗，请填写“既往/合并药物治疗”表；如有合并非药物治疗，请填写“既往/合并非药物治疗”表；如有计划外检查，请填写“计划外检查”表。如有 IMV/NIV、ECMO、HFNC、氧疗，请填写在“IMV/NIV、ECMO、HFNC、氧疗”表中。

本次访视日期：|__|__|__|__|年|__|__|月|__|__|日

**生命体征**	□0 未查，原因________		
检查日期	|__|__|__|__|年|__|__|月|__|__|日		
日最高体温	|__|__|.|__|℃	测量方式	□1 腋下 □2 口腔 □3 肛门<br>□4 耳朵 □5 未知 □6 其他，请描述______
呼吸频率	|__|__|次/分	脉搏	|__|__|__|次/分
血压	|__|__|__|/|__|__|__| mmHg（收缩压/舒张压）		

**有无呼吸道症状** □0 未查，原因________
检查日期：|__|__|__|__|年|__|__|月|__|__|日
受试者有无呼吸道症状：□0 无 □1 有，请描述：________<br>________

血常规 □0 未查，原因________

采样日期：|__|__|__|__|年|__|__|月|__|__|日 正常值范围编号：|__|__|__|__|

检查项目	测量结果		结果判定			未查
	实测值	单位	正常	异常		
				无临床意义	有临床意义	
红细胞计数（RBC）		□1 10^{12}/L □2 其他：____	□1	□2	□3	□0
白细胞计数（WBC）		□1 10^{9}/L □2 其他：____	□1	□2	□3	□0
血红蛋白（Hb）		□1 g/L □2 其他：____	□1	□2	□3	□0
血小板计数（PLT）		□1 10^{9}/L □2 其他：____	□1	□2	□3	□0
中性粒细胞计数（ANC）		□1 10^{9}/L □2 其他：____	□1	□2	□3	□0
淋巴细胞计数（LY）		□1 10^{9}/L □2 其他：____	□1	□2	□3	□0
单核细胞计数（MO）		□1 10^{9}/L □2 其他：____	□1	□2	□3	□0
嗜酸性粒细胞计数（EO）		□1 10^{9}/L □2 其他：____	□1	□2	□3	□0
嗜碱性粒细胞计数（BA）		□1 10^{9}/L □2 其他：____	□1	□2	□3	□0

血生化 □0 未查，原因________

采样日期|__|__|__|__|年|__|__|月|__|__|日 正常值范围编号：|__|__|__|__|

检查项目	测量结果		结果判定			未查
	实测值	单位	正常	异常		
				无临床意义	有临床意义	

谷丙转氨酶（ALT）		□1 U/L □2 其他：_____	□1	□2	□3	□0
谷草转氨酶（AST）		□1 U/L □2 其他：_____	□1	□2	□3	□0
总胆红素（TBIL）		□1 μmol/L □2 其他：_____	□1	□2	□3	□0
白蛋白（ALB）		□1 g/L □2 其他：_____	□1	□2	□3	□0
γ-谷氨酰转肽酶（GGT）		□1 U/L □2 其他：_____	□1	□2	□3	□0
乳酸脱氢酶（LDH）		□1 U/L □2 其他：_____	□1	□2	□3	□0
肌酸激酶（CK）		□1 U/L □2 其他：_____	□1	□2	□3	□0
肌酸激酶同工酶（CK-MB）		□1 U/L □2 其他：_____	□1	□2	□3	□0
血尿素氮（BUN）		□1 mmol/L □2 其他：_____	□1	□2	□3	□0
尿素（Urea）		□1 mmol/L □2 其他：_____	□1	□2	□3	□0
血肌酐（CR）		□1 μmol/L □2 其他：_____	□1	□2	□3	□0

凝血功能 □0 未查，原因______________________________

采样日期：\|__\|__\|__\|__\|年\|__\|__\|月\|__\|__\|日 正常值范围编号：\|__\|__\|__\|__\|

检查项目	测量结果		结果判定			未查
	实测值	单位	正常	异常		
				无临床意义	有临床意义	
凝血酶原时间（PT）		□1 S □2 其他：_____	□1	□2	□3	□0
活化部分凝血活酶时间（APTT）		□1 S □2 其他：_____	□1	□2	□3	□0
凝血酶时间（TT）		□1 S □2 其他：_____	□1	□2	□3	□0
血浆纤维蛋白原（Fbg）		□1 g/L □2 其他：_____	□1	□2	□3	□0
国际标准化比率（INR）		□1 不适用 □2 其他：_____	□1	□2	□3	□0

C 反应蛋白（CRP） □0 未查，原因______________________________

采样日期：\|__\|__\|__\|__\|年\|__\|__\|月\|__\|__\|日 正常值范围编号：\|__\|__\|__\|__\|

检查项目	测量结果		结果判定		
C 反应蛋白（CRP）	实测值	单位	正常	异常	
				无临床意义	有临床意义
		□1 mg/L □2 其他：_____	□1	□2	□3

血沉（ESR） □0 未查，原因______________________________

采样日期：\|__\|__\|__\|__\|年\|__\|__\|月\|__\|__\|日 正常值范围编号：\|__\|__\|__\|__\|

检查项目	测量结果		结果判定	
	实测值	单位	正常	异常

				无临床意义	有临床意义
血沉（ESR）		□1 mm/h □2其他：_____	□1	□2	□3

TNF-α □0 未查，原因______________________________

采样日期：|__|__|__|__|年|__|__|月|__|__|日 正常值范围编号：|__|__|__|__|

检查项目	测量结果		结果判定		
				异常	
TNF-α	实测值	单位	正常	无临床意义	有临床意义
		□1 pg/mL □2其他：_____	□1	□2	□3

IL-6 □0 未查，原因______________________________

采样日期：|__|__|__|__|年|__|__|月|__|__|日 正常值范围编号：|__|__|__|__|

检查项目	测量结果		结果判定		
				异常	
IL-6	实测值	单位	正常	无临床意义	有临床意义
		□1 pg/mL □2其他：_____	□1	□2	□3

**新型冠状病毒核酸检测**	□0 未查，原因______________________________
采样部位（可多选）	□0 上呼吸道 □1 下呼吸道
上呼吸道	采样日期：|__|__|__|__|年|__|__|月|__|__|日<br>检查结果：□1 阴性 □2 阳性 □3 无法判断 □4 其他，请描述_________
下呼吸道	采样日期：|__|__|__|__|年|__|__|月|__|__|日<br>检查结果：□1 阴性 □2 阳性 □3 无法判断 □4 其他，请描述_________
结果判定	□1 阴性 □2 阳性 □3 无法判断 □4 其他，请描述__________________

指脉氧饱和度（不吸氧情况下） □0 未查，原因______________________________

检查日期：|__|__|__|__|年|__|__|月|__|__|日

实测值：_______________%（静息状态）

动脉血气分析 □0 未查，原因______________________________
□1 不适用

是否吸氧：□0 否 □1 是，吸氧浓度（FiO2）：_______________%

采样日期：|__|__|__|__|年|__|__|月|__|__|日 正常值范围编号：|__|__|__|__|

检查项目	测量结果	结果判定	未查

	实测值	单位	正常	异常		
				无临床意义	有临床意义	
动脉血氧分压（PaO2）		□1 mmHg □2 其他：____	□1	□2	□3	□0
血氧饱和度（SaO2）		□1 % □2 其他：____	□1	□2	□3	□0

**临床状态**	□0 未评价，原因________________
评价日期	|__|__|__|__|年|__|__|月|__|__|日
评价结果	□1 1级：出院<br>□2 2级：住院，不需要氧疗<br>□3 3级：住院，需要氧疗（非NIV/HFNC）<br>□4 4级：ICU/住院，需要无创机械通气（NIV）/高流量鼻导管氧疗（HFNC）<br>□5 5级：ICU，需要体外膜肺氧合（ECMO）和/或有创机械通气（IMV）<br>□6 6级：死亡

自上次访视以来，受试者如有不良事件发生，请填写“不良事件”表；如有合并药物治疗，请填写“既往/合并药物治疗”表；如有合并非药物治疗，请填写“既往/合并非药物治疗”表；如有计划外检查，请填写“计划外检查”表。如有 IMV/NIV、ECMO、HFNC、氧疗，请填写在“IMV/NIV、ECMO、HFNC、氧疗”表中。

本次访视日期：|__|__|__|__|年|__|__|月|__|__|日

**生命体征**	□$_0$ 未查，原因________________________		
检查日期	|__|__|__|__|年|__|__|月|__|__|日		
日最高体温	|__|__|.|__|℃	测量方式	□$_1$ 腋下 □$_2$ 口腔 □$_3$ 肛门<br>□$_4$ 耳朵 □$_5$ 未知 □$_6$ 其他，请描述_______
呼吸频率	|__|__|次/分	脉搏	|__|__|__|次/分
血压	|__|__|__|/|__|__|__| mmHg（收缩压/舒张压）		

**体格检查** □$_0$ 未查，原因________________________					
检查日期：|__|__|__|__|年|__|__|月|__|__|日					
**检查部位**	**检查结果**<br>**1=正常**<br>**2=异常无临床意义**<br>**3=异常有临床意义**<br>**0=未查**				**异常请描述**
整体外观	□$_1$	□$_2$	□$_3$	□$_0$	
头	□$_1$	□$_2$	□$_3$	□$_0$	
眼	□$_1$	□$_2$	□$_3$	□$_0$	
耳	□$_1$	□$_2$	□$_3$	□$_0$	
鼻	□$_1$	□$_2$	□$_3$	□$_0$	
喉	□$_1$	□$_2$	□$_3$	□$_0$	
颈部及甲状腺	□$_1$	□$_2$	□$_3$	□$_0$	
皮肤	□$_1$	□$_2$	□$_3$	□$_0$	
心血管系统	□$_1$	□$_2$	□$_3$	□$_0$	
胸部及肺	□$_1$	□$_2$	□$_3$	□$_0$	
腹部	□$_1$	□$_2$	□$_3$	□$_0$	
神经系统	□$_1$	□$_2$	□$_3$	□$_0$	
骨骼肌系统	□$_1$	□$_2$	□$_3$	□$_0$	
淋巴系统	□$_1$	□$_2$	□$_3$	□$_0$	

有无呼吸道症状 □0 未查，原因________________

检查日期：|__|__|__|__|年|__|__|月|__|__|日

受试者有无呼吸道症状：□0 无 □1 有，请描述：________________

____

血常规 □0 未查，原因________________

采样日期：|__|__|__|__|年|__|__|月|__|__|日 正常值范围编号：|__|__|__|__|

检查项目	测量结果		结果判定			未查
	实测值	单位	正常	异常		
				无临床意义	有临床意义	
红细胞计数（RBC）		□1 10^{12}/L □2 其他：_____	□1	□2	□3	□0
白细胞计数（WBC）		□1 10^{9}/L □2 其他：_____	□1	□2	□3	□0
血红蛋白（Hb）		□1 g/L □2 其他：_____	□1	□2	□3	□0
血小板计数（PLT）		□1 10^{9}/L □2 其他：_____	□1	□2	□3	□0
中性粒细胞计数（ANC）		□1 10^{9}/L □2 其他：_____	□1	□2	□3	□0
淋巴细胞计数（LY）		□1 10^{9}/L □2 其他：_____	□1	□2	□3	□0
单核细胞计数（MO）		□1 10^{9}/L □2 其他：_____	□1	□2	□3	□0
嗜酸性粒细胞计数（EO）		□1 10^{9}/L □2 其他：_____	□1	□2	□3	□0
嗜碱性粒细胞计数（BA）		□1 10^{9}/L □2 其他：_____	□1	□2	□3	□0

血生化 □0 未查，原因________________

采样日期|__|__|__|__|年|__|__|月|__|__|日 正常值范围编号：|__|__|__|__|

检查项目	测量结果		结果判定			未查
	实测值	单位	正常	异常		
				无临床意义	有临床意义	
谷丙转氨酶（ALT）		□1 U/L □2 其他：_____	□1	□2	□3	□0
谷草转氨酶（AST）		□1 U/L □2 其他：_____	□1	□2	□3	□0
总胆红素（TBIL）		□1 μmol/L □2 其他：_____	□1	□2	□3	□0
白蛋白（ALB）		□1 g/L □2 其他：_____	□1	□2	□3	□0
γ-谷氨酰转肽酶（GGT）		□1 U/L □2 其他：_____	□1	□2	□3	□0
乳酸脱氢酶（LDH）		□1 U/L □2 其他：_____	□1	□2	□3	□0
肌酸激酶（CK）		□1 U/L □2 其他：_____	□1	□2	□3	□0
肌酸激酶同工酶（CK-MB）		□1 U/L □2 其他：_____	□1	□2	□3	□0

血尿素氮（BUN）		□1 mmol/L □2 其他：_____	□1	□2	□3	□0
尿素（Urea）		□1 mmol/L □2 其他：_____	□1	□2	□3	□0
血肌酐（CR）		□1 μmol/L □2 其他：_____	□1	□2	□3	□0

尿常规 □0 未查，原因____

采样日期：|__|__|__|__|年|__|__|月|__|__|日 正常值范围编号：|__|__|__|__|

检查项目	测量结果		结果判定			未查
	实测值	单位	正常	异常		
				无临床意义	有临床意义	
尿蛋白		□1 不适用 □2 其他：_____	□1	□2	□3	□0
尿葡萄糖		□1 mmol/L □2 其他：_____	□1	□2	□3	□0
尿 PH 值		□1 不适用 □2 其他：_____	□1	□2	□3	□0
尿红细胞		□1 不适用 □2 其他：_____	□1	□2	□3	□0
尿白细胞		□1 不适用 □2 其他：_____	□1	□2	□3	□0
尿酮体		□1 不适用 □2 其他：_____	□1	□2	□3	□0

凝血功能 □0 未查，原因____

采样日期：|__|__|__|__|年|__|__|月|__|__|日 正常值范围编号：|__|__|__|__|

检查项目	测量结果		结果判定			未查
	实测值	单位	正常	异常		
				无临床意义	有临床意义	
凝血酶原时间（PT）		□1 S □2 其他：_____	□1	□2	□3	□0
活化部分凝血活酶时间（APTT）		□1 S □2 其他：_____	□1	□2	□3	□0
凝血酶时间（TT）		□1 S □2 其他：_____	□1	□2	□3	□0
血浆纤维蛋白原（Fbg）		□1 g/L □2 其他：_____	□1	□2	□3	□0
国际标准化比率（INR）		□1 不适用 □2 其他：_____	□1	□2	□3	□0

C 反应蛋白（CRP） □0 未查，原因____

采样日期：|__|__|__|__|年|__|__|月|__|__|日 正常值范围编号：|__|__|__|__|

检查项目	测量结果		结果判定		
C 反应蛋白（CRP）	实测值	单位	正常	异常	
				无临床意义	有临床意义
		□1 mg/L □2 其他：_____	□1	□2	□3

血沉（ESR）　☐0 未查，原因____________________

采样日期：|__|__|__|__|年|__|__|月|__|__|日　　正常值范围编号：|__|__|__|__|

检查项目	测量结果		结果判定		
	实测值	单位	正常	异常：无临床意义	异常：有临床意义
血沉（ESR）		☐1 mm/h　☐2 其他：_____	☐1	☐2	☐3

TNF-α　☐0 未查，原因____________________

采样日期：|__|__|__|__|年|__|__|月|__|__|日　　正常值范围编号：|__|__|__|__|

检查项目	测量结果		结果判定		
	实测值	单位	正常	异常：无临床意义	异常：有临床意义
TNF-α		☐1 pg/mL　☐2 其他：_____	☐1	☐2	☐3

IL-6　☐0 未查，原因____________________

采样日期：|__|__|__|__|年|__|__|月|__|__|日　　正常值范围编号：|__|__|__|__|

检查项目	测量结果		结果判定		
	实测值	单位	正常	异常：无临床意义	异常：有临床意义
IL-6		☐1 pg/mL　☐2 其他：_____	☐1	☐2	☐3

尿或血清 HCG（仅适用于育龄女性）　☐0 未查，原因____________________
☐1 不适用（男性或不具有生育能力的女性）

采样日期：|__|__|__|__|年|__|__|月|__|__|日　　正常值范围编号：|__|__|__|__|

采样类别：☐1 血液　☐2 尿液

检查结果：☐1 阴性　☐2 阳性　☐3 其他：____________

单位：☐1 不适用　☐2 mIU/mL　☐3 其他：____________

12 导联心电图　☐0 未查，原因____________________

检查日期：|__|__|__|__|年|__|__|月|__|__|日

检查结果：☐1 正常　☐2 异常无临床意义　☐3 异常有临床意义

若“异常”，请描述：____________________

____

**胸部 CT** □0 未查，原因______________ □1 不适用
检查日期：|__|__|__|__|年|__|__|月|__|__|日
检查结果：□1 正常 □2 异常无临床意义 □3 异常有临床意义
若异常，请描述：______________
是否胸部 CT 病灶吸收较基线≥50%？ □0 否 □1 是 □2 无法判断 □3 不适用 □4 其他，请描述：______________

**新型冠状病毒核酸检测** □0 未查，原因______________	
采样部位（可多选）	□0 上呼吸道 □1 下呼吸道
上呼吸道	采样日期：|__|__|__|__|年|__|__|月|__|__|日<br>检查结果：□1 阴性 □2 阳性 □3 无法判断 □4 其他，请描述__________
下呼吸道	采样日期：|__|__|__|__|年|__|__|月|__|__|日<br>检查结果：□1 阴性 □2 阳性 □3 无法判断 □4 其他，请描述__________
结果判定	□1 阴性 □2 阳性 □3 无法判断 □4 其他，请描述__________

**指脉氧饱和度**（不吸氧情况下） □0 未查，原因______________
检查日期：|__|__|__|__|年|__|__|月|__|__|日
实测值：______________%（静息状态）

**动脉血气分析** □0 未查，原因______________ □1 不适用
是否吸氧：□0 否 □1 是，吸氧浓度（FiO2）：______________%
采样日期：|__|__|__|__|年|__|__|月|__|__|日 正常值范围编号：|__|__|__|__|__|

检查项目	测量结果		结果判定			未查
	实测值	单位	正常	异常		
				无临床意义	有临床意义	
动脉血氧分压（PaO2）		□1 mmHg □2 其他：_____	□1	□2	□3	□0
血氧饱和度（SaO2）		□1 % □2 其他：_____	□1	□2	□3	□0

**临床状态**	□0 未评价，原因________
评价日期	|__|__|__|__|年|__|__|月|__|__|日
评价结果	□1 1级：出院<br>□2 2级：住院，不需要氧疗<br>□3 3级：住院，需要氧疗（非NIV/HFNC）<br>□4 4级：ICU/住院，需要无创机械通气（NIV）/高流量鼻导管氧疗（HFNC）<br>□5 5级：ICU，需要体外膜肺氧合（ECMO）和/或有创机械通气（IMV）<br>□6 6级：死亡

自上次访视以来，受试者如有不良事件发生，请填写“不良事件”表；如有合并药物治疗，请填写“既往/合并药物治疗”表；如有合并非药物治疗，请填写“既往/合并非药物治疗”表；如有计划外检查，请填写“计划外检查”表。如有 IMV/NIV、ECMO、HFNC、氧疗，请填写在“IMV/NIV、ECMO、HFNC、氧疗”表中。

治疗结束
受试者是否按研究方案完成了所有治疗？ □0 否 □1 是
治疗结束日期：|__|__|__|__|年|__|__|月|__|__|日
如提前退出治疗，请选择最重要的原因（单选）：<br>□1 研究期间出现疾病恶化，经研究者综合评估后需要改变治疗<br>□2 药物不良事件导致病情加重的出现<br>□3 患者不愿意继续使用研究药物<br>□4 妊娠<br>□5 受试者失访<br>□6 受试者死亡<br>□7 受试者撤回知情同意<br>□8 研究整体中止<br>□9 其他，请描述：________________<br>________________<br><br>备注（请记录其他需要补充说明的事由）：<br>________________<br>________________<br>________________

**访视类型：**□1 电话随访	□2 医院随访
**本次访视日期：**|__|__|__|__|年|__|__|月|__|__|日	

如受试者已出院，以下内容可不进行，如未进行请留空；如受试者未出院，请进行以下检查。

**生命体征**	□0 未查，原因______		
检查日期	|__|__|__|__|年|__|__|月|__|__|日		
日最高体温	|__|__|.|__|℃	测量方式	□1 腋下 □2 口腔 □3 肛门<br>□4 耳朵 □5 未知 □6 其他，请描述______
呼吸频率	|__|__|次/分	脉搏	|__|__|__|次/分
血压	|__|__|__|/|__|__|__| mmHg（收缩压/舒张压）		

**体格检查**	□0 未查，原因______				
检查日期：|__|__|__|__|年|__|__|月|__|__|日					
**检查部位**	**检查结果**<br>**1=正常**<br>**2=异常无临床意义**<br>**3=异常有临床意义**<br>**0=未查**				**异常请描述**
整体外观	□1	□2	□3	□0	
头	□1	□2	□3	□0	
眼	□1	□2	□3	□0	
耳	□1	□2	□3	□0	
鼻	□1	□2	□3	□0	
喉	□1	□2	□3	□0	
颈部及甲状腺	□1	□2	□3	□0	
皮肤	□1	□2	□3	□0	
心血管系统	□1	□2	□3	□0	
胸部及肺	□1	□2	□3	□0	
腹部	□1	□2	□3	□0	
神经系统	□1	□2	□3	□0	

骨骼肌系统	□1	□2	□3	□0	
淋巴系统	□1	□2	□3	□0	

有无呼吸道症状 □0 未查，原因________________

检查日期：|__|__|__|__|年|__|__|月|__|__|日

受试者有无呼吸道症状：□0 无 □1 有，请描述：________________

____

血常规 □0 未查，原因________________

采样日期：|__|__|__|__|年|__|__|月|__|__|日 正常值范围编号：|__|__|__|__|

检查项目	测量结果		结果判定			未查
	实测值	单位	正常	异常		
				无临床意义	有临床意义	
红细胞计数（RBC）		□1 10^{12}/L □2 其他：____	□1	□2	□3	□0
白细胞计数（WBC）		□1 10^9/L □2 其他：____	□1	□2	□3	□0
血红蛋白（Hb）		□1 g/L □2 其他：____	□1	□2	□3	□0
血小板计数（PLT）		□1 10^9/L □2 其他：____	□1	□2	□3	□0
中性粒细胞计数（ANC）		□1 10^9/L □2 其他：____	□1	□2	□3	□0
淋巴细胞计数（LY）		□1 10^9/L □2 其他：____	□1	□2	□3	□0
单核细胞计数（MO）		□1 10^9/L □2 其他：____	□1	□2	□3	□0
嗜酸性粒细胞计数（EO）		□1 10^9/L □2 其他：____	□1	□2	□3	□0
嗜碱性粒细胞计数（BA）		□1 10^9/L □2 其他：____	□1	□2	□3	□0

血生化 □0 未查，原因________________

采样日期|__|__|__|__|年|__|__|月|__|__|日 正常值范围编号：|__|__|__|__|

检查项目	测量结果		结果判定			未查
	实测值	单位	正常	异常		
				无临床意义	有临床意义	
谷丙转氨酶（ALT）		□1 U/L □2 其他：____	□1	□2	□3	□0
谷草转氨酶（AST）		□1 U/L □2 其他：____	□1	□2	□3	□0
总胆红素（TBIL）		□1 μmol/L □2 其他：____	□1	□2	□3	□0
白蛋白（ALB）		□1 g/L □2 其他：____	□1	□2	□3	□0
γ-谷氨酰转肽酶（GGT）		□1 U/L □2 其他：____	□1	□2	□3	□0
乳酸脱氢酶（LDH）		□1 U/L □2 其他：____	□1	□2	□3	□0

肌酸激酶（CK）		□$_1$ U/L　□$_2$其他：_____	□$_1$	□$_2$	□$_3$	□$_0$
肌酸激酶同工酶（CK-MB）		□$_1$ U/L　□$_2$其他：_____	□$_1$	□$_2$	□$_3$	□$_0$
血尿素氮（BUN）		□$_1$ mmol/L　□$_2$其他：_____	□$_1$	□$_2$	□$_3$	□$_0$
尿素（Urea）		□$_1$ mmol/L　□$_2$其他：_____	□$_1$	□$_2$	□$_3$	□$_0$
血肌酐（CR）		□$_1$ μmol/L　□$_2$其他：_____	□$_1$	□$_2$	□$_3$	□$_0$

尿常规　　□$_0$未查，原因____

采样日期：\|__\|__\|__\|__\|年\|__\|__\|月\|__\|__\|日　　正常值范围编号：\|__\|__\|__\|__\|

检查项目	测量结果		结果判定			未查
	实测值	单位	正常	异常		
				无临床意义	有临床意义	
尿蛋白		□$_1$不适用　□$_2$其他：_____	□$_1$	□$_2$	□$_3$	□$_0$
尿葡萄糖		□$_1$ mmol/L　□$_2$其他：_____	□$_1$	□$_2$	□$_3$	□$_0$
尿 PH 值		□$_1$不适用　□$_2$其他：_____	□$_1$	□$_2$	□$_3$	□$_0$
尿红细胞		□$_1$不适用　□$_2$其他：_____	□$_1$	□$_2$	□$_3$	□$_0$
尿白细胞		□$_1$不适用　□$_2$其他：_____	□$_1$	□$_2$	□$_3$	□$_0$
尿酮体		□$_1$不适用　□$_2$其他：_____	□$_1$	□$_2$	□$_3$	□$_0$

凝血功能　　□$_0$未查，原因____

采样日期：\|__\|__\|__\|__\|年\|__\|__\|月\|__\|__\|日　　正常值范围编号：\|__\|__\|__\|__\|

检查项目	测量结果		结果判定			未查
	实测值	单位	正常	异常		
				无临床意义	有临床意义	
凝血酶原时间（PT）		□$_1$ S　□$_2$其他：_____	□$_1$	□$_2$	□$_3$	□$_0$
活化部分凝血活酶时间（APTT）		□$_1$ S　□$_2$其他：_____	□$_1$	□$_2$	□$_3$	□$_0$
凝血酶时间（TT）		□$_1$ S　□$_2$其他：_____	□$_1$	□$_2$	□$_3$	□$_0$
血浆纤维蛋白原（Fbg）		□$_1$ g/L　□$_2$其他：_____	□$_1$	□$_2$	□$_3$	□$_0$
国际标准化比率（INR）		□$_1$不适用　□$_2$其他：_____	□$_1$	□$_2$	□$_3$	□$_0$

C 反应蛋白（CRP）　　□$_0$未查，原因____________________________________

采样日期：\|__\|__\|__\|__\|年\|__\|__\|月\|__\|__\|日　　正常值范围编号：\|__\|__\|__\|__\|

检查项目	测量结果		结果判定	
	实测值	单位	正常	异常

C 反应蛋白（CRP）				无临床意义	有临床意义
		□1 mg/L　□2 其他：_____	□1	□2	□3

血沉（ESR）　□0 未查，原因________________

采样日期：|__|__|__|__|年|__|__|月|__|__|日　　正常值范围编号：|__|__|__|__|

检查项目	测量结果		结果判定		
	实测值	单位	正常	异常	
				无临床意义	有临床意义
血沉（ESR）		□1 mm/h　□2 其他：_____	□1	□2	□3

TNF-α　□0 未查，原因________________

采样日期：|__|__|__|__|年|__|__|月|__|__|日　　正常值范围编号：|__|__|__|__|

检查项目	测量结果		结果判定		
	实测值	单位	正常	异常	
				无临床意义	有临床意义
TNF-α		□1 pg/mL　□2 其他：_____	□1	□2	□3

IL-6　□0 未查，原因________________

采样日期：|__|__|__|__|年|__|__|月|__|__|日　　正常值范围编号：|__|__|__|__|

检查项目	测量结果		结果判定		
	实测值	单位	正常	异常	
				无临床意义	有临床意义
IL-6		□1 pg/mL　□2 其他：_____	□1	□2	□3

12 导联心电图　□0 未查，原因________________

检查日期：|__|__|__|__|年|__|__|月|__|__|日

检查结果：□1 正常　□2 异常无临床意义　□3 异常有临床意义

若“异常”，请描述：________________

____

胸部 CT　□0 未查，原因________________
□1 不适用

检查日期：|__|__|__|__|年|__|__|月|__|__|日

检查结果：□1 正常　□2 异常无临床意义　□3 异常有临床意义

若异常，请描述：____
是否胸部 CT 病灶吸收较基线≥50%？ □$_0$ 否 □$_1$ 是 □$_2$ 无法判断 □$_3$ 不适用<br>□$_4$ 其他，请描述：________________

**新型冠状病毒核酸检测**	□$_0$ 未查，原因________________________
采样部位（可多选）	□$_0$ 上呼吸道 □$_1$ 下呼吸道
上呼吸道	采样日期：|__|__|__|__|年|__|__|月|__|__|日<br>检查结果：□$_1$ 阴性 □$_2$ 阳性 □$_3$ 无法判断 □$_4$ 其他，请描述_________
下呼吸道	采样日期：|__|__|__|__|年|__|__|月|__|__|日<br>检查结果：□$_1$ 阴性 □$_2$ 阳性 □$_3$ 无法判断 □$_4$ 其他，请描述_________
结果判定	□$_1$ 阴性 □$_2$ 阳性 □$_3$ 无法判断 □$_4$ 其他，请描述_______________

**指脉氧饱和度**（不吸氧情况下） □$_0$ 未查，原因________________________
检查日期：|__|__|__|__|年|__|__|月|__|__|日
实测值：_____________%（静息状态）

**动脉血气分析** □$_0$ 未查，原因________________________<br>□$_1$ 不适用
是否吸氧：□$_0$ 否 □$_1$ 是，吸氧浓度（FiO2）：_____________%
采样日期：|__|__|__|__|年|__|__|月|__|__|日 正常值范围编号：|__|__|__|__|

检查项目	测量结果		结果判定			未查
	实测值	单位	正常	异常		
				无临床意义	有临床意义	
动脉血氧分压（PaO2）		□$_1$ mmHg □$_2$ 其他：____	□$_1$	□$_2$	□$_3$	□$_0$
血氧饱和度（SaO2）		□$_1$ % □$_2$ 其他：____	□$_1$	□$_2$	□$_3$	□$_0$

**临床状态**	□$_0$ 未评价，原因________________________
评价日期	|__|__|__|__|年|__|__|月|__|__|日
评价结果	□$_1$ 1级：出院<br>□$_2$ 2级：住院，不需要氧疗<br>□$_3$ 3级：住院，需要氧疗（非NIV/HFNC）<br>□$_4$ 4级：ICU/住院，需要无创机械通气（NIV）/高流量鼻导管氧疗（HFNC）

	□$_5$ 5级：ICU，需要体外膜肺氧合（ECMO）和/或有创机械通气（IMV）<br>□$_6$ 6级：死亡

自上次访视以来，受试者如有不良事件发生，请填写“不良事件”表；如有合并药物治疗，请填写“既往/合并药物治疗”表；如有合并非药物治疗，请填写“既往/合并非药物治疗”表；如有计划外检查，请填写“计划外检查”表。如有 IMV/NIV、ECMO、HFNC、氧疗，请填写在“IMV/NIV、ECMO、HFNC、氧疗”表中。

受试者是否使用试验药物？ □0 否 □1 是 □2 不适用（对照组）

试验药物使用情况

服药天数	是否用药	用药日期	计划每日给药总剂量（mg）	实际每日给药总剂量（mg）	给药频率（1）	给药情况（2）	如剂量调整或暂停给药，请说明原因	如漏服、多服，或其他情况请说明
第__天	□0 否<br>□1 是	|__|__|__|__|年|__|__|月|__|__|日	|__|__|__|__|__|	|__|__|__|__|__|	|__| __________	|__| __________	□1 不良事件，编号：___<br>□2 其他：__________	
第__天	□0 否<br>□1 是	|__|__|__|__|年|__|__|月|__|__|日	|__|__|__|__|__|	|__|__|__|__|__|	|__| __________	|__| __________	□1 不良事件，编号：___<br>□2 其他：__________	
第__天	□0 否<br>□1 是	|__|__|__|__|年|__|__|月|__|__|日	|__|__|__|__|__|	|__|__|__|__|__|	|__| __________	|__| __________	□1 不良事件，编号：___<br>□2 其他：__________	
第__天	□0 否<br>□1 是	|__|__|__|__|年|__|__|月|__|__|日	|__|__|__|__|__|	|__|__|__|__|__|	|__| __________	|__| __________	□1 不良事件，编号：___<br>□2 其他：__________	
第__天	□0 否<br>□1 是	|__|__|__|__|年|__|__|月|__|__|日	|__|__|__|__|__|	|__|__|__|__|__|	|__| __________	|__| __________	□1 不良事件，编号：___<br>□2 其他：__________	
第__天	□0 否<br>□1 是	|__|__|__|__|年|__|__|月|__|__|日	|__|__|__|__|__|	|__|__|__|__|__|	|__| __________	|__| __________	□1 不良事件，编号：___<br>□2 其他：__________	
第__天	□0 否<br>□1 是	|__|__|__|__|年|__|__|月|__|__|日	|__|__|__|__|__|	|__|__|__|__|__|	|__| __________	|__| __________	□1 不良事件，编号：___<br>□2 其他：__________	
第__天	□0 否<br>□1 是	|__|__|__|__|年|__|__|月|__|__|日	|__|__|__|__|__|	|__|__|__|__|__|	|__| __________	|__| __________	□1 不良事件，编号：___<br>□2 其他：__________	

（1）给药频率：1=每日三次 2=每日两次 3=每日一次 4=未知 5=其他，请在横线处具体描述
（2）给药情况：1=不适用 2=剂量调整 3=漏服 4=多服 5=暂停给药 6=其他，请在横线处具体描述

试验药物使用情况								
服药天数	是否用药	用药日期	计划每日给药总剂量（mg）	实际每日给药总剂量（mg）	给药频率（1）	给药情况（2）	如剂量调整或暂停给药，请说明原因	如漏服、多服，或其他情况请说明
第__天	□0 否 □1 是	|__|__|__|__|年|__|__|月 |__|__|日	|__|__|__|__|__|	|__|__|__|__|__|	|__| ________	|__| ________	□1 不良事件，编号：__ □2 其他：________	
第__天	□0 否 □1 是	|__|__|__|__|年|__|__|月 |__|__|日	|__|__|__|__|__|	|__|__|__|__|__|	|__| ________	|__| ________	□1 不良事件，编号：__ □2 其他：________	
第__天	□0 否 □1 是	|__|__|__|__|年|__|__|月 |__|__|日	|__|__|__|__|__|	|__|__|__|__|__|	|__| ________	|__| ________	□1 不良事件，编号：__ □2 其他：________	
第__天	□0 否 □1 是	|__|__|__|__|年|__|__|月 |__|__|日	|__|__|__|__|__|	|__|__|__|__|__|	|__| ________	|__| ________	□1 不良事件，编号：__ □2 其他：________	
第__天	□0 否 □1 是	|__|__|__|__|年|__|__|月 |__|__|日	|__|__|__|__|__|	|__|__|__|__|__|	|__| ________	|__| ________	□1 不良事件，编号：__ □2 其他：________	
第__天	□0 否 □1 是	|__|__|__|__|年|__|__|月 |__|__|日	|__|__|__|__|__|	|__|__|__|__|__|	|__| ________	|__| ________	□1 不良事件，编号：__ □2 其他：________	
第__天	□0 否 □1 是	|__|__|__|__|年|__|__|月 |__|__|日	|__|__|__|__|__|	|__|__|__|__|__|	|__| ________	|__| ________	□1 不良事件，编号：__ □2 其他：________	
第__天	□0 否 □1 是	|__|__|__|__|年|__|__|月 |__|__|日	|__|__|__|__|__|	|__|__|__|__|__|	|__| ________	|__| ________	□1 不良事件，编号：__ □2 其他：________	

（1）给药频率： 1=每日三次 2=每日两次 3=每日一次 4=未知 5=其他，请在横线处具体描述

（2）给药情况： 1=不适用 2=剂量调整 3=漏服 4=多服 5=暂停给药 6=其他，请在横线处具体描述

受试者是否发生不良事件？　　□0 否　　□1 **是**，请参照表格下方的编码详细填写在下表中。

不良事件											
**编号**	**不良事件名称**	**开始日期**	**研究结束时是否持续？**		**CTCAE 等级（1）**	**采取的措施**		**与研究药物的关系（4）**	**AE 转归（5）**	**是否为SAE？**	
			**是**	**否→结束日期**		**对研究药物的措施（2）**	**其它措施（3）**			**否**	**是，SAE 具体类型（6）**
**1**		|__|__|__|__|年 |__|__|月|__|__|日	□	|__|__|__|__|年 |__|__|月|__|__|日	|__|	|__| ________	|__| ________	|__|	|__|	□	|__|
**2**		|__|__|__|__|年 |__|__|月|__|__|日	□	|__|__|__|__|年 |__|__|月|__|__|日	|__|	|__| ________	|__| ________	|__|	|__|	□	|__|
**3**		|__|__|__|__|年 |__|__|月|__|__|日	□	|__|__|__|__|年 |__|__|月|__|__|日	|__|	|__| ________	|__| ________	|__|	|__|	□	|__|
**4**		|__|__|__|__|年 |__|__|月|__|__|日	□	|__|__|__|__|年 |__|__|月|__|__|日	|__|	|__| ________	|__| ________	|__|	|__|	□	|__|

**（1）CTCAE等级**	**（2）对研究药物的措施**	**（3）其它措施**	**（4）与研究药物关系**	**（5）AE转归**	**（6）SAE具体类型**
1=1级<br>2=2级<br>3=3级<br>4=4级<br>5=5级	1=维持目前治疗<br>2=用药暂停<br>3=用药终止<br>4=减小剂量<br>5=不适用<br>6=其它，请在横线处具体描述	1=无<br>2=合并用药，填写既往/合并药物治疗表<br>3=合并非药物治疗，填写既往/合并非药物治疗表<br>4=其它，请在横线处具体描述	1=肯定有关<br>2=很可能有关<br>3=可能有关<br>4=可能无关<br>5=肯定无关<br>6=待评价<br>7=无法评价	1=未恢复/未治愈<br>2=恢复/治愈<br>3=恢复但留有后遗症<br>4=死亡<br>5=未知	1=危及生命<br>2=导致死亡<br>3=需住院治疗或延长目前住院时间<br>4=导致永久或显著的残疾或功能障碍<br>5=导致先天畸形/出生缺陷<br>6=其它重要的医学事件

不良事件											
编号	不良事件名称	开始日期	研究结束时是否持续？		CTCAE 等级（1）	采取的措施		与研究药物的关系（4）	AE 转归（5）	是否为SAE？	
			是	否→结束日期		对研究药物的措施（2）	其它措施（3）			否	是，SAE 具体类型（6）
		|__|__|__|__|年<br>|__|__|月|__|__|日	☐	|__|__|__|__|年<br>|__|__|月|__|__|日	|__|	|__| ________	|__| ________	|__|	|__|	☐	|__|
		|__|__|__|__|年<br>|__|__|月|__|__|日	☐	|__|__|__|__|年<br>|__|__|月|__|__|日	|__|	|__| ________	|__| ________	|__|	|__|	☐	|__|
		|__|__|__|__|年<br>|__|__|月|__|__|日	☐	|__|__|__|__|年<br>|__|__|月|__|__|日	|__|	|__| ________	|__| ________	|__|	|__|	☐	|__|
		|__|__|__|__|年<br>|__|__|月|__|__|日	☐	|__|__|__|__|年<br>|__|__|月|__|__|日	|__|	|__| ________	|__| ________	|__|	|__|	☐	|__|
		|__|__|__|__|年<br>|__|__|月|__|__|日	☐	|__|__|__|__|年<br>|__|__|月|__|__|日	|__|	|__| ____	|__| ____	|__|	|__|	☐	|__|

（1）CTCAE等级	（2）对研究药物的措施	（3）其它措施	（4）与研究药物关系	（5）AE转归	（6）SAE具体类型
1=1级<br>2=2级<br>3=3级<br>4=4级<br>5=5级	1=维持目前治疗<br>2=用药暂停<br>3=用药终止<br>4=减小剂量<br>5=不适用<br>6=其它，请在横线处具体描述	1=无<br>2=合并用药，填写既往/合并药物治疗表<br>3=合并非药物治疗，填写既往/合并非药物治疗表<br>4=其它，请在横线处具体描述	1=肯定有关<br>2=很可能有关<br>3=可能有关<br>4=可能无关<br>5=肯定无关<br>6=待评价<br>7=无法评价	1=未恢复/未治愈<br>2=恢复/治愈<br>3=恢复但留有后遗症<br>4=死亡<br>5=未知	1=危及生命<br>2=导致死亡<br>3=需住院治疗或延长目前住院时间<br>4=导致永久或显著的残疾或功能障碍<br>5=导致先天畸形/出生缺陷<br>6=其它重要的医学事件

受试者是否有既往/合并药物治疗？ □0 否 □1 是，请参照表格下方的编码详细填写在下表中。

既往/合并药物治疗

编号	药物名称（通用名）	适应症（1）	适应症备注	单次剂量	剂量单位（2）	给药频率（3）	给药途径（4）	开始日期	研究结束时是否持续？是	研究结束时是否持续？否→结束日期	是否为指南推荐的标准治疗（5）
1		|__|			|__| ____	|__| ____	|__| ____	|__|__|__|__|年 |__|__|月|__|__|日	□	|__|__|__|__|年 |__|__|月|__|__|日	|__|
2		|__|			|__| ____	|__| ____	|__| ____	|__|__|__|__|年 |__|__|月|__|__|日	□	|__|__|__|__|年 |__|__|月|__|__|日	|__|
3		|__|			|__| ____	|__| ____	|__| ____	|__|__|__|__|年 |__|__|月|__|__|日	□	|__|__|__|__|年 |__|__|月|__|__|日	|__|
4		|__|			|__| ____	|__| ____	|__| ____	|__|__|__|__|年 |__|__|月|__|__|日	□	|__|__|__|__|年 |__|__|月|__|__|日	|__|
5		|__|			|__| ____	|__| ____	|__| ____	|__|__|__|__|年 |__|__|月|__|__|日	□	|__|__|__|__|年 |__|__|月|__|__|日	|__|

（1）适应症	（2）剂量单位		（3）给药频率		（4）给药途径		（5）指南推荐标准治疗
1=本研究疾病	1=微克(μg)	6=单位(U)	1=每日一次(qd)	6=每周一次(qw)	1=口服(PO)	7=局部（皮肤）(TOP)	0=否
2=既往/伴随疾病（请在备注处填写既往/伴随疾病编号）	2=毫克(mg)	7=片(Tab)	2=每日二次(bid)	7=睡前(hs)	2=静脉滴注(IVgtt)	8=眼(OPH)	1=是
3=不良事件（请在备注处填写不良事件编号）	3=克(g)	8=胶囊(Cap)	3=每日三次(tid)	8=按需(prn)	3=静脉注射(IV)	9=吸入(INH)	2=不适用
4=其它（请在备注处描述）	4=毫升(mL)	9=未知	4=每日四次(qid)	9=未知	4=肌内注射(IM)	10=未知	
	5=国际单位(IU)	10=其它，请在横线处填写	5=隔日一次(qod)	10=其它，请在横线处填写	5=皮下注射(SQ)	11=其它，请在横线处填写	
					6=舌下(SL)		

既往/合并药物治疗									研究结束时是否持续？		是否为指南推荐的标准治疗（5）
编号	药物名称（通用名）	适应症（1）	适应症备注	单次剂量	剂量单位（2）	给药频率（3）	给药途径（4）	开始日期	是	否→结束日期	
		|__|			|__| ______	|__| ______	|__| ______	|__|__|__|__|年 |__|__|月|__|__|日	□	|__|__|__|__|年 |__|__|月|__|__|日	|__|
		|__|			|__| ______	|__| ______	|__| ______	|__|__|__|__|年 |__|__|月|__|__|日	□	|__|__|__|__|年 |__|__|月|__|__|日	|__|
		|__|			|__| ______	|__| ______	|__| ______	|__|__|__|__|年 |__|__|月|__|__|日	□	|__|__|__|__|年 |__|__|月|__|__|日	|__|
		|__|			|__| ______	|__| ______	|__| ______	|__|__|__|__|年 |__|__|月|__|__|日	□	|__|__|__|__|年 |__|__|月|__|__|日	|__|
		|__|			|__| ______	|__| ______	|__| ______	|__|__|__|__|年 |__|__|月|__|__|日	□	|__|__|__|__|年 |__|__|月|__|__|日	|__|

（1）适应症	（2）剂量单位		（3）给药频率		（4）给药途径		（5）指南推荐标准治疗
1=本研究疾病<br>2=既往/伴随疾病（请在备注处填写既往/伴随疾病编号）<br>3=不良事件（请在备注处填写不良事件编号）<br>4=其它（请在备注处描述）	1=微克(μg)<br>2=毫克(mg)<br>3=克(g)<br>4=毫升(mL)<br>5=国际单位(IU)	6=单位(U)<br>7=片(Tab)<br>8=胶囊(Cap)<br>9=未知<br>10=其它，请在横线处填写	1=每日一次(qd)<br>2=每日二次(bid)<br>3=每日三次(tid)<br>4=每日四次(qid)<br>5=隔日一次(qod)	6=每周一次(qw)<br>7=睡前(hs)<br>8=按需(prn)<br>9=未知<br>10=其它，请在横线处填写	1=口服(PO)<br>2=静脉滴注(IVgtt)<br>3=静脉注射(IV)<br>4=肌内注射(IM)<br>5=皮下注射(SQ)<br>6=舌下(SL)	7=局部（皮肤）(TOP)<br>8=眼(OPH)<br>9=吸入(INH)<br>10=未知<br>11=其它，请在横线处填写	0=否<br>1=是<br>2=不适用

<table>
<tr><td colspan="9">受试者是否有既往/合并非药物治疗？ □0 否 □1 是，请继续填写下表。</td></tr>
<tr><th colspan="9">既往/合并非药物治疗</th></tr>
<tr><th rowspan="2">编号</th><th rowspan="2">治疗名称
（请用专业的医学术语描述）</th><th rowspan="2">适应症
（1）</th><th rowspan="2">适应症备注</th><th rowspan="2">开始日期</th><th colspan="2">研究结束时是否持续？</th><th rowspan="2">是否为指南推荐的标准治疗
（2）</th></tr>
<tr><th>是</th><th>否→结束日期</th></tr>
<tr><td>1</td><td></td><td>|__|</td><td></td><td>|__|__|__|__|年|__|__|月|__|__|日</td><td>□</td><td>|__|__|__|__|年|__|__|月|__|__|日</td><td>|__|</td></tr>
<tr><td>2</td><td></td><td>|__|</td><td></td><td>|__|__|__|__|年|__|__|月|__|__|日</td><td>□</td><td>|__|__|__|__|年|__|__|月|__|__|日</td><td>|__|</td></tr>
<tr><td>3</td><td></td><td>|__|</td><td></td><td>|__|__|__|__|年|__|__|月|__|__|日</td><td>□</td><td>|__|__|__|__|年|__|__|月|__|__|日</td><td>|__|</td></tr>
<tr><td>4</td><td></td><td>|__|</td><td></td><td>|__|__|__|__|年|__|__|月|__|__|日</td><td>□</td><td>|__|__|__|__|年|__|__|月|__|__|日</td><td>|__|</td></tr>
<tr><td>5</td><td></td><td>|__|</td><td></td><td>|__|__|__|__|年|__|__|月|__|__|日</td><td>□</td><td>|__|__|__|__|年|__|__|月|__|__|日</td><td>|__|</td></tr>
<tr><td>6</td><td></td><td>|__|</td><td></td><td>|__|__|__|__|年|__|__|月|__|__|日</td><td>□</td><td>|__|__|__|__|年|__|__|月|__|__|日</td><td>|__|</td></tr>
<tr><td>7</td><td></td><td>|__|</td><td></td><td>|__|__|__|__|年|__|__|月|__|__|日</td><td>□</td><td>|__|__|__|__|年|__|__|月|__|__|日</td><td>|__|</td></tr>
<tr><td>8</td><td></td><td>|__|</td><td></td><td>|__|__|__|__|年|__|__|月|__|__|日</td><td>□</td><td>|__|__|__|__|年|__|__|月|__|__|日</td><td>|__|</td></tr>
<tr><td colspan="9">（1）适应症：1=本研究疾病 2=既往/伴随疾病（请在备注处填写既往/伴随疾病编号） 3=不良事件（请在备注处填写不良事件编号） 4=其它（请在备注处描述）
（2）是否为指南推荐的标准治疗：0=否 1=是 2=不适用</td></tr>
</table>

既往/合并非药物治疗							
**编号**	**治疗名称**<br>（请用专业的医学术语描述）	**适应症（1）**	**适应症备注**	**开始日期**	**研究结束时是否持续？**		**是否为指南推荐的标准治疗（2）**
					**是**	**否→结束日期**	
		|__|		|__|__|__|__|年|__|__|月|__|__|日	☐	|__|__|__|__|年|__|__|月|__|__|日	|__|
		|__|		|__|__|__|__|年|__|__|月|__|__|日	☐	|__|__|__|__|年|__|__|月|__|__|日	|__|
		|__|		|__|__|__|__|年|__|__|月|__|__|日	☐	|__|__|__|__|年|__|__|月|__|__|日	|__|
		|__|		|__|__|__|__|年|__|__|月|__|__|日	☐	|__|__|__|__|年|__|__|月|__|__|日	|__|
		|__|		|__|__|__|__|年|__|__|月|__|__|日	☐	|__|__|__|__|年|__|__|月|__|__|日	|__|
		|__|		|__|__|__|__|年|__|__|月|__|__|日	☐	|__|__|__|__|年|__|__|月|__|__|日	|__|
		|__|		|__|__|__|__|年|__|__|月|__|__|日	☐	|__|__|__|__|年|__|__|月|__|__|日	|__|
		|__|		|__|__|__|__|年|__|__|月|__|__|日	☐	|__|__|__|__|年|__|__|月|__|__|日	|__|
		|__|		|__|__|__|__|年|__|__|月|__|__|日	☐	|__|__|__|__|年|__|__|月|__|__|日	|__|

（1）适应症：1=本研究疾病　2=既往/伴随疾病（请在备注处填写既往/伴随疾病编号）　3=不良事件（请在备注处填写不良事件编号）　4=其它（请在备注处描述）
（2）是否为指南推荐的标准治疗：0=否　1=是　2=不适用

受试者是否有IMV/NIV、ECMO、HFNC、氧疗？ □0 否 □1 是，请继续填写下表。

IMV/NIV、ECMO、HFNC、氧疗

编号	治疗名称	治疗开始日期	治疗开始时间	治疗结束日期	治疗结束时间
**1**	□1 IMV □3 ECMO<br>□2 NIV □4 HFNC<br>□5 氧疗	|__|__|__|__|年|__|__|月|__|__|日	|__|__|:|__|__|	|__|__|__|__|年|__|__|月|__|__|日	|__|__|:|__|__|
**2**	□1 IMV □3 ECMO<br>□2 NIV □4 HFNC<br>□5 氧疗	|__|__|__|__|年|__|__|月|__|__|日	|__|__|:|__|__|	|__|__|__|__|年|__|__|月|__|__|日	|__|__|:|__|__|
**3**	□1 IMV □3 ECMO<br>□2 NIV □4 HFNC<br>□5 氧疗	|__|__|__|__|年|__|__|月|__|__|日	|__|__|:|__|__|	|__|__|__|__|年|__|__|月|__|__|日	|__|__|:|__|__|
**4**	□1 IMV □3 ECMO<br>□2 NIV □4 HFNC<br>□5 氧疗	|__|__|__|__|年|__|__|月|__|__|日	|__|__|:|__|__|	|__|__|__|__|年|__|__|月|__|__|日	|__|__|:|__|__|
**5**	□1 IMV □3 ECMO<br>□2 NIV □4 HFNC<br>□5 氧疗	|__|__|__|__|年|__|__|月|__|__|日	|__|__|:|__|__|	|__|__|__|__|年|__|__|月|__|__|日	|__|__|:|__|__|
**6**	□1 IMV □3 ECMO<br>□2 NIV □4 HFNC<br>□5 氧疗	|__|__|__|__|年|__|__|月|__|__|日	|__|__|:|__|__|	|__|__|__|__|年|__|__|月|__|__|日	|__|__|:|__|__|
**7**	□1 IMV □3 ECMO<br>□2 NIV □4 HFNC<br>□5 氧疗	|__|__|__|__|年|__|__|月|__|__|日	|__|__|:|__|__|	|__|__|__|__|年|__|__|月|__|__|日	|__|__|:|__|__|

IMV/NIV、ECMO、HFNC、氧疗					
编号	治疗名称	治疗开始日期	治疗开始时间	治疗结束日期	治疗结束时间
	□₁ IMV □₃ ECMO<br>□₂ NIV □₄ HFNC<br>□₅ 氧疗	|__|__|__|__|年|__|__|月|__|__|日	|__|__|:|__|__|	|__|__|__|__|年|__|__|月|__|__|日	|__|__|:|__|__|
	□₁ IMV □₃ ECMO<br>□₂ NIV □₄ HFNC<br>□₅ 氧疗	|__|__|__|__|年|__|__|月|__|__|日	|__|__|:|__|__|	|__|__|__|__|年|__|__|月|__|__|日	|__|__|:|__|__|
	□₁ IMV □₃ ECMO<br>□₂ NIV □₄ HFNC<br>□₅ 氧疗	|__|__|__|__|年|__|__|月|__|__|日	|__|__|:|__|__|	|__|__|__|__|年|__|__|月|__|__|日	|__|__|:|__|__|
	□₁ IMV □₃ ECMO<br>□₂ NIV □₄ HFNC<br>□₅ 氧疗	|__|__|__|__|年|__|__|月|__|__|日	|__|__|:|__|__|	|__|__|__|__|年|__|__|月|__|__|日	|__|__|:|__|__|
	□₁ IMV □₃ ECMO<br>□₂ NIV □₄ HFNC<br>□₅ 氧疗	|__|__|__|__|年|__|__|月|__|__|日	|__|__|:|__|__|	|__|__|__|__|年|__|__|月|__|__|日	|__|__|:|__|__|
	□₁ IMV □₃ ECMO<br>□₂ NIV □₄ HFNC<br>□₅ 氧疗	|__|__|__|__|年|__|__|月|__|__|日	|__|__|:|__|__|	|__|__|__|__|年|__|__|月|__|__|日	|__|__|:|__|__|
	□₁ IMV □₃ ECMO<br>□₂ NIV □₄ HFNC<br>□₅ 氧疗	|__|__|__|__|年|__|__|月|__|__|日	|__|__|:|__|__|	|__|__|__|__|年|__|__|月|__|__|日	|__|__|:|__|__|

受试者是否有其他检查需要记录？　□0 否　□1 是，请填写下表。							
**其他检查**							
**检查项目**	**采样/检查日期**	**检查结果**	**单位**	**结果判定**<br>**1=正常**<br>**2=异常无临床意义**<br>**3=异常有临床意义**			**备注**
	|__|__|__|__|年|__|__|月|__|__|日			□1	□2	□3	
	|__|__|__|__|年|__|__|月|__|__|日			□1	□2	□3	
	|__|__|__|__|年|__|__|月|__|__|日			□1	□2	□3	
	|__|__|__|__|年|__|__|月|__|__|日			□1	□2	□3	
	|__|__|__|__|年|__|__|月|__|__|日			□1	□2	□3	
	|__|__|__|__|年|__|__|月|__|__|日			□1	□2	□3	
	|__|__|__|__|年|__|__|月|__|__|日			□1	□2	□3	

其他检查							
检查项目	采样/检查日期	检查结果	单位	结果判定<br>1=正常<br>2=异常无临床意义<br>3=异常有临床意义			备注
	|__|__|__|__|年|__|__|月|__|__|日			□1	□2	□3	
	|__|__|__|__|年|__|__|月|__|__|日			□1	□2	□3	
	|__|__|__|__|年|__|__|月|__|__|日			□1	□2	□3	
	|__|__|__|__|年|__|__|月|__|__|日			□1	□2	□3	
	|__|__|__|__|年|__|__|月|__|__|日			□1	□2	□3	
	|__|__|__|__|年|__|__|月|__|__|日			□1	□2	□3	
	|__|__|__|__|年|__|__|月|__|__|日			□1	□2	□3	

研究总结
受试者是否按研究方案完成了研究？<br>□$_1$ 是，完成日期：|__|__|__|__|年|__|__|月|__|__|日<br>□$_0$ 否，终止日期：|__|__|__|__|年|__|__|月|__|__|日
如提前退出研究，请选择最重要的原因（单选）：<br>□$_1$ 研究期间出现疾病恶化，经研究者综合评估后需要改变治疗<br>□$_2$ 药物不良事件导致病情加重的出现<br>□$_3$ 患者不愿意继续使用研究药物<br>□$_4$ 妊娠<br>□$_5$ 受试者失访<br>□$_6$ 受试者死亡<br>□$_7$ 受试者撤回知情同意<br>□$_8$ 研究整体中止<br>□$_9$ 其他，请描述：________________________________<br>____<br><br>备注（请记录其他需要补充说明的事由）：<br>____<br>____<br>____

住院情况
受试者是否因新型冠状病毒肺炎住院？ □0 否 □1 是
如是，入院日期：|__|__|__|__|年|__|__|月|__|__|日
研究结束时是否出院：□0 否 □1 是
如是，出院日期：|__|__|__|__|年|__|__|月|__|__|日

死亡
受试者是否死亡？ □$_0$ 否 □$_1$ 是，请填写。 □$_2$ 未知
死亡日期：|__|__|__|__|年|__|__|月|__|__|日
死亡原因：□$_1$ 不良事件<br>□$_2$ 未知<br>□$_3$ 其他，请描述：________________________<br>________________________<br>________________________

研究者声明
我已审核并确认此病例报告表中记录的所有数据均真实、准确、可靠、完整无误，并与原始资料一致，所有错误或遗漏均已得到更正或注明。<br><br>**主要研究者签字：________________________ 签字日期：|__|__|__|__|年|__|__|月|__|__|日**

CRA 声明
我已核对并确认此病例报告表中记录的所有数据均真实、准确、可靠、完整无误，并与原始资料一致。<br><br>**CRA 签字：_____________________________ 签字日期：|__|__|__|__|年|__|__|月|__|__|日**

生命体征	□0 未查，原因____		
检查日期	|__|__|__|__|年|__|__|月|__|__|日		
日最高体温	|__|__|.|__|℃	测量方式	□1 腋下 □2 口腔 □3 肛门<br>□4 耳朵 □5 未知 □6 其他，请描述______
呼吸频率	|__|__|次/分	脉搏	|__|__|__|次/分
血压	|__|__|__|/|__|__|__| mmHg（收缩压/舒张压）		

生命体征	□0 未查，原因____		
检查日期	|__|__|__|__|年|__|__|月|__|__|日		
日最高体温	|__|__|.|__|℃	测量方式	□1 腋下 □2 口腔 □3 肛门<br>□4 耳朵 □5 未知 □6 其他，请描述______
呼吸频率	|__|__|次/分	脉搏	|__|__|__|次/分
血压	|__|__|__|/|__|__|__| mmHg（收缩压/舒张压）		

生命体征	□0 未查，原因____		
检查日期	|__|__|__|__|年|__|__|月|__|__|日		
日最高体温	|__|__|.|__|℃	测量方式	□1 腋下 □2 口腔 □3 肛门<br>□4 耳朵 □5 未知 □6 其他，请描述______
呼吸频率	|__|__|次/分	脉搏	|__|__|__|次/分
血压	|__|__|__|/|__|__|__| mmHg（收缩压/舒张压）		

生命体征	□0 未查，原因____		
检查日期	|__|__|__|__|年|__|__|月|__|__|日		
日最高体温	|__|__|.|__|℃	测量方式	□1 腋下 □2 口腔 □3 肛门<br>□4 耳朵 □5 未知 □6 其他，请描述______
呼吸频率	|__|__|次/分	脉搏	|__|__|__|次/分
血压	|__|__|__|/|__|__|__| mmHg（收缩压/舒张压）		

**体格检查**	□0 未查，原因________________				
检查日期：|__|__|__|__|年|__|__|月|__|__|日					
**检查部位**	**检查结果**<br>**1=正常**<br>**2=异常无临床意义**<br>**3=异常有临床意义**<br>**0=未查**				**异常请描述**
整体外观	□1	□2	□3	□0	
头	□1	□2	□3	□0	
眼	□1	□2	□3	□0	
耳	□1	□2	□3	□0	
鼻	□1	□2	□3	□0	
喉	□1	□2	□3	□0	
颈部及甲状腺	□1	□2	□3	□0	
皮肤	□1	□2	□3	□0	
心血管系统	□1	□2	□3	□0	
胸部及肺	□1	□2	□3	□0	
腹部	□1	□2	□3	□0	
神经系统	□1	□2	□3	□0	
骨骼肌系统	□1	□2	□3	□0	
淋巴系统	□1	□2	□3	□0	

**有无呼吸道症状** □0 未查，原因________________________________
检查日期：|__|__|__|__|__|年|__|__|月|__|__|日
受试者有无呼吸道症状：□0 无 □1 有，请描述：________________________________ ________________________________

**有无呼吸道症状** □0 未查，原因________________________________
检查日期：|__|__|__|__|__|年|__|__|月|__|__|日
受试者有无呼吸道症状：□0 无 □1 有，请描述：________________________________ ________________________________

**有无呼吸道症状** □0 未查，原因________________________________
检查日期：|__|__|__|__|__|年|__|__|月|__|__|日
受试者有无呼吸道症状：□0 无 □1 有，请描述：________________________________ ________________________________

**有无呼吸道症状** □0 未查，原因________________________________
检查日期：|__|__|__|__|__|年|__|__|月|__|__|日
受试者有无呼吸道症状：□0 无 □1 有，请描述：________________________________ ________________________________

**有无呼吸道症状** □0 未查，原因________________________________
检查日期：|__|__|__|__|__|年|__|__|月|__|__|日
受试者有无呼吸道症状：□0 无 □1 有，请描述：________________________________ ________________________________

**有无呼吸道症状** □0 未查，原因________________________________
检查日期：|__|__|__|__|__|年|__|__|月|__|__|日
受试者有无呼吸道症状：□0 无 □1 有，请描述：________________________________ ________________________________

**有无呼吸道症状** □0 未查，原因________________________________
检查日期：|__|__|__|__|__|年|__|__|月|__|__|日
受试者有无呼吸道症状：□0 无 □1 有，请描述：________________________________ ________________________________

研究方案编号：KY2020-29		附表-计划外检查
研究中心编号 |__|__|	受试者筛选号 |__|__|__|__|__|__|	

血常规 □$_0$未查，原因________________________________

采样日期：|__|__|__|__|年|__|__|月|__|__|日 正常值范围编号：|__|__|__|__|

检查项目	测量结果		结果判定			未查
	实测值	单位	正常	异常		
				无临床意义	有临床意义	
红细胞计数（RBC）		□$_1$$10^{12}$/L □$_2$其他：_____	□$_1$	□$_2$	□$_3$	□$_0$
白细胞计数（WBC）		□$_1$ 10^9/L □$_2$其他：_____	□$_1$	□$_2$	□$_3$	□$_0$
血红蛋白（Hb）		□$_1$ g/L □$_2$其他：_____	□$_1$	□$_2$	□$_3$	□$_0$
血小板计数（PLT）		□$_1$ 10^9/L □$_2$其他：_____	□$_1$	□$_2$	□$_3$	□$_0$
中性粒细胞计数（ANC）		□$_1$ 10^9/L □$_2$其他：_____	□$_1$	□$_2$	□$_3$	□$_0$
淋巴细胞计数（LY）		□$_1$ 10^9/L □$_2$其他：_____	□$_1$	□$_2$	□$_3$	□$_0$
单核细胞计数（MO）		□$_1$ 10^9/L □$_2$其他：_____	□$_1$	□$_2$	□$_3$	□$_0$
嗜酸性粒细胞计数（EO）		□$_1$ 10^9/L □$_2$其他：_____	□$_1$	□$_2$	□$_3$	□$_0$
嗜碱性粒细胞计数（BA）		□$_1$ 10^9/L □$_2$其他：_____	□$_1$	□$_2$	□$_3$	□$_0$

血常规 □$_0$未查，原因________________________________

采样日期：|__|__|__|__|年|__|__|月|__|__|日 正常值范围编号：|__|__|__|__|

检查项目	测量结果		结果判定			未查
	实测值	单位	正常	异常		
				无临床意义	有临床意义	
红细胞计数（RBC）		□$_1$$10^{12}$/L □$_2$其他：_____	□$_1$	□$_2$	□$_3$	□$_0$
白细胞计数（WBC）		□$_1$ 10^9/L □$_2$其他：_____	□$_1$	□$_2$	□$_3$	□$_0$
血红蛋白（Hb）		□$_1$ g/L □$_2$其他：_____	□$_1$	□$_2$	□$_3$	□$_0$
血小板计数（PLT）		□$_1$ 10^9/L □$_2$其他：_____	□$_1$	□$_2$	□$_3$	□$_0$
中性粒细胞计数（ANC）		□$_1$ 10^9/L □$_2$其他：_____	□$_1$	□$_2$	□$_3$	□$_0$
淋巴细胞计数（LY）		□$_1$ 10^9/L □$_2$其他：_____	□$_1$	□$_2$	□$_3$	□$_0$
单核细胞计数（MO）		□$_1$ 10^9/L □$_2$其他：_____	□$_1$	□$_2$	□$_3$	□$_0$
嗜酸性粒细胞计数（EO）		□$_1$ 10^9/L □$_2$其他：_____	□$_1$	□$_2$	□$_3$	□$_0$
嗜碱性粒细胞计数（BA）		□$_1$ 10^9/L □$_2$其他：_____	□$_1$	□$_2$	□$_3$	□$_0$

血生化 □0 未查，原因________________

采样日期|__|__|__|__|年|__|__|月|__|__|日 正常值范围编号：|__|__|__|__|__|

检查项目	测量结果		结果判定			未查
	实测值	单位	正常	异常		
				无临床意义	有临床意义	
谷丙转氨酶（ALT）		□1 U/L □2 其他：_____	□1	□2	□3	□0
谷草转氨酶（AST）		□1 U/L □2 其他：_____	□1	□2	□3	□0
总胆红素（TBIL）		□1 μmol/L □2 其他：_____	□1	□2	□3	□0
白蛋白（ALB）		□1 g/L □2 其他：_____	□1	□2	□3	□0
γ-谷氨酰转肽酶（GGT）		□1 U/L □2 其他：_____	□1	□2	□3	□0
乳酸脱氢酶（LDH）		□1 U/L □2 其他：_____	□1	□2	□3	□0
肌酸激酶（CK）		□1 U/L □2 其他：_____	□1	□2	□3	□0
肌酸激酶同工酶（CK-MB）		□1 U/L □2 其他：_____	□1	□2	□3	□0
血尿素氮（BUN）		□1 mmol/L □2 其他：_____	□1	□2	□3	□0
尿素（Urea）		□1 mmol/L □2 其他：_____	□1	□2	□3	□0
血肌酐（CR）		□1 μmol/L □2 其他：_____	□1	□2	□3	□0

血生化 □0 未查，原因________________

采样日期|__|__|__|__|年|__|__|月|__|__|日 正常值范围编号：|__|__|__|__|__|

检查项目	测量结果		结果判定			未查
	实测值	单位	正常	异常		
				无临床意义	有临床意义	
谷丙转氨酶（ALT）		□1 U/L □2 其他：_____	□1	□2	□3	□0
谷草转氨酶（AST）		□1 U/L □2 其他：_____	□1	□2	□3	□0
总胆红素（TBIL）		□1 μmol/L □2 其他：_____	□1	□2	□3	□0
白蛋白（ALB）		□1 g/L □2 其他：_____	□1	□2	□3	□0
γ-谷氨酰转肽酶（GGT）		□1 U/L □2 其他：_____	□1	□2	□3	□0
乳酸脱氢酶（LDH）		□1 U/L □2 其他：_____	□1	□2	□3	□0
肌酸激酶（CK）		□1 U/L □2 其他：_____	□1	□2	□3	□0
肌酸激酶同工酶（CK-MB）		□1 U/L □2 其他：_____	□1	□2	□3	□0
血尿素氮（BUN）		□1 mmol/L □2 其他：_____	□1	□2	□3	□0
尿素（Urea）		□1 mmol/L □2 其他：_____	□1	□2	□3	□0
血肌酐（CR）		□1 μmol/L □2 其他：_____	□1	□2	□3	□0

**尿常规**	☐₀ 未查，原因________					
采样日期：|__|__|__|__|年|__|__|月|__|__|日				正常值范围编号：|__|__|__|__|		
检查项目	测量结果		结果判定			未查
	实测值	单位	正常	异常		
				无临床意义	有临床意义	
尿蛋白		☐1 不适用　☐2 其他：_____	☐1	☐2	☐3	☐0
尿葡萄糖		☐1 mmol/L　☐2 其他：_____	☐1	☐2	☐3	☐0
尿 PH 值		☐1 不适用　☐2 其他：_____	☐1	☐2	☐3	☐0
尿红细胞		☐1 不适用　☐2 其他：_____	☐1	☐2	☐3	☐0
尿白细胞		☐1 不适用　☐2 其他：_____	☐1	☐2	☐3	☐0
尿酮体		☐1 不适用　☐2 其他：_____	☐1	☐2	☐3	☐0

**尿常规**	☐₀ 未查，原因________					
采样日期：|__|__|__|__|年|__|__|月|__|__|日				正常值范围编号：|__|__|__|__|		
检查项目	测量结果		结果判定			未查
	实测值	单位	正常	异常		
				无临床意义	有临床意义	
尿蛋白		☐1 不适用　☐2 其他：_____	☐1	☐2	☐3	☐0
尿葡萄糖		☐1 mmol/L　☐2 其他：_____	☐1	☐2	☐3	☐0
尿 PH 值		☐1 不适用　☐2 其他：_____	☐1	☐2	☐3	☐0
尿红细胞		☐1 不适用　☐2 其他：_____	☐1	☐2	☐3	☐0
尿白细胞		☐1 不适用　☐2 其他：_____	☐1	☐2	☐3	☐0
尿酮体		☐1 不适用　☐2 其他：_____	☐1	☐2	☐3	☐0

**尿常规**	☐₀ 未查，原因________					
采样日期：|__|__|__|__|年|__|__|月|__|__|日				正常值范围编号：|__|__|__|__|		
检查项目	测量结果		结果判定			未查
	实测值	单位	正常	异常		
				无临床意义	有临床意义	
尿蛋白		☐1 不适用　☐2 其他：_____	☐1	☐2	☐3	☐0
尿葡萄糖		☐1 mmol/L　☐2 其他：_____	☐1	☐2	☐3	☐0
尿 PH 值		☐1 不适用　☐2 其他：_____	☐1	☐2	☐3	☐0
尿红细胞		☐1 不适用　☐2 其他：_____	☐1	☐2	☐3	☐0
尿白细胞		☐1 不适用　☐2 其他：_____	☐1	☐2	☐3	☐0
尿酮体		☐1 不适用　☐2 其他：_____	☐1	☐2	☐3	☐0

凝血功能 □$_0$ 未查，原因________________________________

采样日期：|__|__|__|__|年|__|__|月|__|__|日 正常值范围编号：|__|__|__|__|

检查项目	测量结果		结果判定			未查
	实测值	单位	正常	异常		
				无临床意义	有临床意义	
凝血酶原时间（PT）		□$_1$ S □$_2$ 其他：_____	□$_1$	□$_2$	□$_3$	□$_0$
活化部分凝血活酶时间（APTT）		□$_1$ S □$_2$ 其他：_____	□$_1$	□$_2$	□$_3$	□$_0$
凝血酶时间（TT）		□$_1$ S □$_2$ 其他：_____	□$_1$	□$_2$	□$_3$	□$_0$
血浆纤维蛋白原（Fbg）		□$_1$ g/L □$_2$ 其他：_____	□$_1$	□$_2$	□$_3$	□$_0$
国际标准化比率（INR）		□$_1$ 不适用 □$_2$ 其他：_____	□$_1$	□$_2$	□$_3$	□$_0$

凝血功能 □$_0$ 未查，原因________________________________

采样日期：|__|__|__|__|年|__|__|月|__|__|日 正常值范围编号：|__|__|__|__|

检查项目	测量结果		结果判定			未查
	实测值	单位	正常	异常		
				无临床意义	有临床意义	
凝血酶原时间（PT）		□$_1$ S □$_2$ 其他：_____	□$_1$	□$_2$	□$_3$	□$_0$
活化部分凝血活酶时间（APTT）		□$_1$ S □$_2$ 其他：_____	□$_1$	□$_2$	□$_3$	□$_0$
凝血酶时间（TT）		□$_1$ S □$_2$ 其他：_____	□$_1$	□$_2$	□$_3$	□$_0$
血浆纤维蛋白原（Fbg）		□$_1$ g/L □$_2$ 其他：_____	□$_1$	□$_2$	□$_3$	□$_0$
国际标准化比率（INR）		□$_1$ 不适用 □$_2$ 其他：_____	□$_1$	□$_2$	□$_3$	□$_0$

凝血功能 □$_0$ 未查，原因________________________________

采样日期：|__|__|__|__|年|__|__|月|__|__|日 正常值范围编号：|__|__|__|__|

检查项目	测量结果		结果判定			未查
	实测值	单位	正常	异常		
				无临床意义	有临床意义	
凝血酶原时间（PT）		□$_1$ S □$_2$ 其他：_____	□$_1$	□$_2$	□$_3$	□$_0$
活化部分凝血活酶时间（APTT）		□$_1$ S □$_2$ 其他：_____	□$_1$	□$_2$	□$_3$	□$_0$
凝血酶时间（TT）		□$_1$ S □$_2$ 其他：_____	□$_1$	□$_2$	□$_3$	□$_0$
血浆纤维蛋白原（Fbg）		□$_1$ g/L □$_2$ 其他：_____	□$_1$	□$_2$	□$_3$	□$_0$
国际标准化比率（INR）		□$_1$ 不适用 □$_2$ 其他：_____	□$_1$	□$_2$	□$_3$	□$_0$

C 反应蛋白（CRP）　☐0 未查，原因________________

采样日期：|__|__|__|__|年|__|__|月|__|__|日　　正常值范围编号：|__|__|__|__|

检查项目	测量结果		结果判定		
				异常	
	实测值	单位	正常	无临床意义	有临床意义
C 反应蛋白（CRP）		☐1 mg/L　☐2 其他：_____	☐1	☐2	☐3

C 反应蛋白（CRP）　☐0 未查，原因________________

采样日期：|__|__|__|__|年|__|__|月|__|__|日　　正常值范围编号：|__|__|__|__|

检查项目	测量结果		结果判定		
				异常	
	实测值	单位	正常	无临床意义	有临床意义
C 反应蛋白（CRP）		☐1 mg/L　☐2 其他：_____	☐1	☐2	☐3

C 反应蛋白（CRP）　☐0 未查，原因________________

采样日期：|__|__|__|__|年|__|__|月|__|__|日　　正常值范围编号：|__|__|__|__|

检查项目	测量结果		结果判定		
				异常	
	实测值	单位	正常	无临床意义	有临床意义
C 反应蛋白（CRP）		☐1 mg/L　☐2 其他：_____	☐1	☐2	☐3

C 反应蛋白（CRP）　☐0 未查，原因________________

采样日期：|__|__|__|__|年|__|__|月|__|__|日　　正常值范围编号：|__|__|__|__|

检查项目	测量结果		结果判定		
				异常	
	实测值	单位	正常	无临床意义	有临床意义
C 反应蛋白（CRP）		☐1 mg/L　☐2 其他：_____	☐1	☐2	☐3

C 反应蛋白（CRP）　☐0 未查，原因________________

采样日期：|__|__|__|__|年|__|__|月|__|__|日　　正常值范围编号：|__|__|__|__|

检查项目	测量结果		结果判定		
				异常	
	实测值	单位	正常	无临床意义	有临床意义
C 反应蛋白（CRP）		☐1 mg/L　☐2 其他：_____	☐1	☐2	☐3

血沉（ESR）	□0 未查，原因______					
采样日期：|__|__|__|__|年|__|__|月|__|__|日			正常值范围编号：|__|__|__|__|			
检查项目	测量结果		结果判定			
血沉（ESR）	实测值	单位	正常	异常		
				无临床意义	有临床意义	
		□1 mm/h □2其他：_____	□1	□2	□3	

血沉（ESR）	□0 未查，原因______					
采样日期：|__|__|__|__|年|__|__|月|__|__|日			正常值范围编号：|__|__|__|__|			
检查项目	测量结果		结果判定			
血沉（ESR）	实测值	单位	正常	异常		
				无临床意义	有临床意义	
		□1 mm/h □2其他：_____	□1	□2	□3	

血沉（ESR）	□0 未查，原因______					
采样日期：|__|__|__|__|年|__|__|月|__|__|日			正常值范围编号：|__|__|__|__|			
检查项目	测量结果		结果判定			
血沉（ESR）	实测值	单位	正常	异常		
				无临床意义	有临床意义	
		□1 mm/h □2其他：_____	□1	□2	□3	

血沉（ESR）	□0 未查，原因______					
采样日期：|__|__|__|__|年|__|__|月|__|__|日			正常值范围编号：|__|__|__|__|			
检查项目	测量结果		结果判定			
血沉（ESR）	实测值	单位	正常	异常		
				无临床意义	有临床意义	
		□1 mm/h □2其他：_____	□1	□2	□3	

血沉（ESR）	□0 未查，原因______					
采样日期：|__|__|__|__|年|__|__|月|__|__|日			正常值范围编号：|__|__|__|__|			
检查项目	测量结果		结果判定			
血沉（ESR）	实测值	单位	正常	异常		
				无临床意义	有临床意义	
		□1 mm/h □2其他：_____	□1	□2	□3	

TNF-α	□0 未查，原因____________				
采样日期：|__|__|__|__|年|__|__|月|__|__|日			正常值范围编号：|__|__|__|__|		
检查项目	测量结果		结果判定		
				异常	
	实测值	单位	正常	无临床意义	有临床意义
TNF-α		□1 pg/mL □2其他：_____	□1	□2	□3

TNF-α	□0 未查，原因____________				
采样日期：|__|__|__|__|年|__|__|月|__|__|日			正常值范围编号：|__|__|__|__|		
检查项目	测量结果		结果判定		
				异常	
	实测值	单位	正常	无临床意义	有临床意义
TNF-α		□1 pg/mL □2其他：_____	□1	□2	□3

TNF-α	□0 未查，原因____________				
采样日期：|__|__|__|__|年|__|__|月|__|__|日			正常值范围编号：|__|__|__|__|		
检查项目	测量结果		结果判定		
				异常	
	实测值	单位	正常	无临床意义	有临床意义
TNF-α		□1 pg/mL □2其他：_____	□1	□2	□3

TNF-α	□0 未查，原因____________				
采样日期：|__|__|__|__|年|__|__|月|__|__|日			正常值范围编号：|__|__|__|__|		
检查项目	测量结果		结果判定		
				异常	
	实测值	单位	正常	无临床意义	有临床意义
TNF-α		□1 pg/mL □2其他：_____	□1	□2	□3

TNF-α	□0 未查，原因____________				
采样日期：|__|__|__|__|年|__|__|月|__|__|日			正常值范围编号：|__|__|__|__|		
检查项目	测量结果		结果判定		
				异常	
	实测值	单位	正常	无临床意义	有临床意义
TNF-α		□1 pg/mL □2其他：_____	□1	□2	□3

IL-6　　☐0 未查，原因________________

采样日期：|__|__|__|__|年|__|__|月|__|__|日　　正常值范围编号：|__|__|__|__|

检查项目	测量结果		结果判定		
				异常	
	实测值	单位	正常	无临床意义	有临床意义
IL-6		☐1 pg/mL　☐2其他：_____	☐1	☐2	☐3

IL-6　　☐0 未查，原因________________

采样日期：|__|__|__|__|年|__|__|月|__|__|日　　正常值范围编号：|__|__|__|__|

检查项目	测量结果		结果判定		
				异常	
	实测值	单位	正常	无临床意义	有临床意义
IL-6		☐1 pg/mL　☐2其他：_____	☐1	☐2	☐3

IL-6　　☐0 未查，原因________________

采样日期：|__|__|__|__|年|__|__|月|__|__|日　　正常值范围编号：|__|__|__|__|

检查项目	测量结果		结果判定		
				异常	
	实测值	单位	正常	无临床意义	有临床意义
IL-6		☐1 pg/mL　☐2其他：_____	☐1	☐2	☐3

IL-6　　☐0 未查，原因________________

采样日期：|__|__|__|__|年|__|__|月|__|__|日　　正常值范围编号：|__|__|__|__|

检查项目	测量结果		结果判定		
				异常	
	实测值	单位	正常	无临床意义	有临床意义
IL-6		☐1 pg/mL　☐2其他：_____	☐1	☐2	☐3

IL-6　　☐0 未查，原因________________

采样日期：|__|__|__|__|年|__|__|月|__|__|日　　正常值范围编号：|__|__|__|__|

检查项目	测量结果		结果判定		
				异常	
	实测值	单位	正常	无临床意义	有临床意义
IL-6		☐1 pg/mL　☐2其他：_____	☐1	☐2	☐3

**尿或血清 HCG（仅适用于育龄女性）**	□0 未查，原因________________<br>□1 不适用（男性或不具有生育能力的女性）
采样日期：|__|__|__|__|年|__|__|月|__|__|日	正常值范围编号：|__|__|__|__|__|
采样类别：□1 血液　□2 尿液	
检查结果：□1 阴性　□2 阳性　□3 其他：________________	
单位：　□1 不适用　□2 mIU/mL　□3 其他：________________	

**尿或血清 HCG（仅适用于育龄女性）**	□0 未查，原因________________<br>□1 不适用（男性或不具有生育能力的女性）
采样日期：|__|__|__|__|年|__|__|月|__|__|日	正常值范围编号：|__|__|__|__|__|
采样类别：□1 血液　□2 尿液	
检查结果：□1 阴性　□2 阳性　□3 其他：________________	
单位：　□1 不适用　□2 mIU/mL　□3 其他：________________	

**尿或血清 HCG（仅适用于育龄女性）**	□0 未查，原因________________<br>□1 不适用（男性或不具有生育能力的女性）
采样日期：|__|__|__|__|年|__|__|月|__|__|日	正常值范围编号：|__|__|__|__|__|
采样类别：□1 血液　□2 尿液	
检查结果：□1 阴性　□2 阳性　□3 其他：________________	
单位：　□1 不适用　□2 mIU/mL　□3 其他：________________	

**尿或血清 HCG（仅适用于育龄女性）**	□0 未查，原因________________<br>□1 不适用（男性或不具有生育能力的女性）
采样日期：|__|__|__|__|年|__|__|月|__|__|日	正常值范围编号：|__|__|__|__|__|
采样类别：□1 血液　□2 尿液	
检查结果：□1 阴性　□2 阳性　□3 其他：________________	
单位：　□1 不适用　□2 mIU/mL　□3 其他：________________	

**12 导联心电图**　☐0 未查，原因________
检查日期：|__|__|__|__|年|__|__|月|__|__|日
检查结果：☐1 正常　☐2 异常无临床意义　☐3 异常有临床意义
若“异常”，请描述：________

**12 导联心电图**　☐0 未查，原因________
检查日期：|__|__|__|__|年|__|__|月|__|__|日
检查结果：☐1 正常　☐2 异常无临床意义　☐3 异常有临床意义
若“异常”，请描述：________

**12 导联心电图**　☐0 未查，原因________
检查日期：|__|__|__|__|年|__|__|月|__|__|日
检查结果：☐1 正常　☐2 异常无临床意义　☐3 异常有临床意义
若“异常”，请描述：________

**12 导联心电图**　☐0 未查，原因________
检查日期：|__|__|__|__|年|__|__|月|__|__|日
检查结果：☐1 正常　☐2 异常无临床意义　☐3 异常有临床意义
若“异常”，请描述：________

**12 导联心电图**　☐0 未查，原因________
检查日期：|__|__|__|__|年|__|__|月|__|__|日
检查结果：☐1 正常　☐2 异常无临床意义　☐3 异常有临床意义
若“异常”，请描述：________

**12 导联心电图**　☐0 未查，原因________
检查日期：|__|__|__|__|年|__|__|月|__|__|日
检查结果：☐1 正常　☐2 异常无临床意义　☐3 异常有临床意义
若“异常”，请描述：________

**新型冠状病毒核酸检测**	□$_0$ 未查，原因________________________________
采样部位（可多选）	□$_0$ 上呼吸道　　□$_1$ 下呼吸道
上呼吸道	采样日期：|__|__|__|__|年|__|__|月|__|__|日<br>检查结果：□$_1$ 阴性 □$_2$ 阳性 □$_3$ 无法判断 □$_4$ 其他，请描述_________
下呼吸道	采样日期：|__|__|__|__|年|__|__|月|__|__|日<br>检查结果：□$_1$ 阴性 □$_2$ 阳性 □$_3$ 无法判断 □$_4$ 其他，请描述_________
结果判定	□$_1$ 阴性 □$_2$ 阳性 □$_3$ 无法判断 □$_4$ 其他，请描述__________________

**新型冠状病毒核酸检测**	□$_0$ 未查，原因________________________________
采样部位（可多选）	□$_0$ 上呼吸道　　□$_1$ 下呼吸道
上呼吸道	采样日期：|__|__|__|__|年|__|__|月|__|__|日<br>检查结果：□$_1$ 阴性 □$_2$ 阳性 □$_3$ 无法判断 □$_4$ 其他，请描述_________
下呼吸道	采样日期：|__|__|__|__|年|__|__|月|__|__|日<br>检查结果：□$_1$ 阴性 □$_2$ 阳性 □$_3$ 无法判断 □$_4$ 其他，请描述_________
结果判定	□$_1$ 阴性 □$_2$ 阳性 □$_3$ 无法判断 □$_4$ 其他，请描述__________________

**新型冠状病毒核酸检测**	□$_0$ 未查，原因________________________________
采样部位（可多选）	□$_0$ 上呼吸道　　□$_1$ 下呼吸道
上呼吸道	采样日期：|__|__|__|__|年|__|__|月|__|__|日<br>检查结果：□$_1$ 阴性 □$_2$ 阳性 □$_3$ 无法判断 □$_4$ 其他，请描述_________
下呼吸道	采样日期：|__|__|__|__|年|__|__|月|__|__|日<br>检查结果：□$_1$ 阴性 □$_2$ 阳性 □$_3$ 无法判断 □$_4$ 其他，请描述_________
结果判定	□$_1$ 阴性 □$_2$ 阳性 □$_3$ 无法判断 □$_4$ 其他，请描述__________________

**新型冠状病毒核酸检测**	□$_0$ 未查，原因________________________________
采样部位（可多选）	□$_0$ 上呼吸道　　□$_1$ 下呼吸道
上呼吸道	采样日期：|__|__|__|__|年|__|__|月|__|__|日<br>检查结果：□$_1$ 阴性 □$_2$ 阳性 □$_3$ 无法判断 □$_4$ 其他，请描述_________
下呼吸道	采样日期：|__|__|__|__|年|__|__|月|__|__|日<br>检查结果：□$_1$ 阴性 □$_2$ 阳性 □$_3$ 无法判断 □$_4$ 其他，请描述_________
结果判定	□$_1$ 阴性 □$_2$ 阳性 □$_3$ 无法判断 □$_4$ 其他，请描述__________________

**指脉氧饱和度**（不吸氧情况下） □0 未查，原因________________________
检查日期：|__|__|__|__|年|__|__|月|__|__|日
实测值：________________%（静息状态）

**指脉氧饱和度**（不吸氧情况下） □0 未查，原因________________________
检查日期：|__|__|__|__|年|__|__|月|__|__|日
实测值：________________%（静息状态）

**指脉氧饱和度**（不吸氧情况下） □0 未查，原因________________________
检查日期：|__|__|__|__|年|__|__|月|__|__|日
实测值：________________%（静息状态）

**指脉氧饱和度**（不吸氧情况下） □0 未查，原因________________________
检查日期：|__|__|__|__|年|__|__|月|__|__|日
实测值：________________%（静息状态）

**指脉氧饱和度**（不吸氧情况下） □0 未查，原因________________________
检查日期：|__|__|__|__|年|__|__|月|__|__|日
实测值：________________%（静息状态）

**指脉氧饱和度**（不吸氧情况下） □0 未查，原因________________________
检查日期：|__|__|__|__|年|__|__|月|__|__|日
实测值：________________%（静息状态）

**指脉氧饱和度**（不吸氧情况下） □0 未查，原因________________________
检查日期：|__|__|__|__|年|__|__|月|__|__|日
实测值：________________%（静息状态）

**胸部 CT** ☐0 未查，原因__________ ☐1 不适用
检查日期：|__|__|__|__|年|__|__|月|__|__|日
检查结果：☐1 正常 ☐2 异常无临床意义 ☐3 异常有临床意义
若异常，请描述：__________
是否胸部 CT 病灶吸收较基线≥50%？ ☐0 否 ☐1 是 ☐2 无法判断 ☐3 不适用 ☐4 其他，请描述：__________

**胸部 CT** ☐0 未查，原因__________ ☐1 不适用
检查日期：|__|__|__|__|年|__|__|月|__|__|日
检查结果：☐1 正常 ☐2 异常无临床意义 ☐3 异常有临床意义
若异常，请描述：__________
是否胸部 CT 病灶吸收较基线≥50%？ ☐0 否 ☐1 是 ☐2 无法判断 ☐3 不适用 ☐4 其他，请描述：__________

**胸部 CT** ☐0 未查，原因__________ ☐1 不适用
检查日期：|__|__|__|__|年|__|__|月|__|__|日
检查结果：☐1 正常 ☐2 异常无临床意义 ☐3 异常有临床意义
若异常，请描述：__________
是否胸部 CT 病灶吸收较基线≥50%？ ☐0 否 ☐1 是 ☐2 无法判断 ☐3 不适用 ☐4 其他，请描述：__________

动脉血气分析 □0未查，原因________________________________

是否吸氧：□0否 □1是，吸氧浓度（FiO2）：______________%

采样日期：|__|__|__|__|年|__|__|月|__|__|日 正常值范围编号：|__|__|__|__|

检查项目	测量结果		结果判定			未查
	实测值	单位	正常	异常		
				无临床意义	有临床意义	
动脉血氧分压（PaO2）		□1 mmHg □2其他：____	□1	□2	□3	□0
血氧饱和度（SaO2）		□1 % □2其他：____	□1	□2	□3	□0

动脉血气分析 □0未查，原因________________________________

是否吸氧：□0否 □1是，吸氧浓度（FiO2）：______________%

采样日期：|__|__|__|__|年|__|__|月|__|__|日 正常值范围编号：|__|__|__|__|

检查项目	测量结果		结果判定			未查
	实测值	单位	正常	异常		
				无临床意义	有临床意义	
动脉血氧分压（PaO2）		□1 mmHg □2其他：____	□1	□2	□3	□0
血氧饱和度（SaO2）		□1 % □2其他：____	□1	□2	□3	□0

动脉血气分析 □0未查，原因________________________________

是否吸氧：□0否 □1是，吸氧浓度（FiO2）：______________%

采样日期：|__|__|__|__|年|__|__|月|__|__|日 正常值范围编号：|__|__|__|__|

检查项目	测量结果		结果判定			未查
	实测值	单位	正常	异常		
				无临床意义	有临床意义	
动脉血氧分压（PaO2）		□1 mmHg □2其他：____	□1	□2	□3	□0
血氧饱和度（SaO2）		□1 % □2其他：____	□1	□2	□3	□0

临床状态	□0 未评价，原因________________
评价日期	|__|__|__|__|年|__|__|月|__|__|日
评价结果	□1 1级：出院<br>□2 2级：住院，不需要氧疗<br>□3 3级：住院，需要氧疗（非NIV/HFNC）<br>□4 4级：ICU/住院，需要无创机械通气（NIV）/高流量鼻导管氧疗（HFNC）<br>□5 5级：ICU，需要体外膜肺氧合（ECMO）和/或有创机械通气（IMV）<br>□6 6级：死亡

临床状态	□0 未评价，原因________________
评价日期	|__|__|__|__|年|__|__|月|__|__|日
评价结果	□1 1级：出院<br>□2 2级：住院，不需要氧疗<br>□3 3级：住院，需要氧疗（非NIV/HFNC）<br>□4 4级：ICU/住院，需要无创机械通气（NIV）/高流量鼻导管氧疗（HFNC）<br>□5 5级：ICU，需要体外膜肺氧合（ECMO）和/或有创机械通气（IMV）<br>□6 6级：死亡

临床状态	□0 未评价，原因________________
评价日期	|__|__|__|__|年|__|__|月|__|__|日
评价结果	□1 1级：出院<br>□2 2级：住院，不需要氧疗<br>□3 3级：住院，需要氧疗（非NIV/HFNC）<br>□4 4级：ICU/住院，需要无创机械通气（NIV）/高流量鼻导管氧疗（HFNC）<br>□5 5级：ICU，需要体外膜肺氧合（ECMO）和/或有创机械通气（IMV）<br>□6 6级：死亡

临床状态	□0 未评价，原因________________
评价日期	|__|__|__|__|年|__|__|月|__|__|日
评价结果	□1 1级：出院<br>□2 2级：住院，不需要氧疗<br>□3 3级：住院，需要氧疗（非NIV/HFNC）<br>□4 4级：ICU/住院，需要无创机械通气（NIV）/高流量鼻导管氧疗（HFNC）<br>□5 5级：ICU，需要体外膜肺氧合（ECMO）和/或有创机械通气（IMV）<br>□6 6级：死亡

**签署知情同意书**	
签署知情同意书日期	|__|__|__|__|年|__|__|月|__|__|日
知情同意书版本号	____________________
签署知情同意书时适用方案版本号	|__|__|.|__|

**签署知情同意书**	
签署知情同意书日期	|__|__|__|__|年|__|__|月|__|__|日
知情同意书版本号	____________________
签署知情同意书时适用方案版本号	|__|__|.|__|

**签署知情同意书**	
签署知情同意书日期	|__|__|__|__|年|__|__|月|__|__|日
知情同意书版本号	____________________
签署知情同意书时适用方案版本号	|__|__|.|__|

**签署知情同意书**	
签署知情同意书日期	|__|__|__|__|年|__|__|月|__|__|日
知情同意书版本号	____________________
签署知情同意书时适用方案版本号	|__|__|.|__|

**签署知情同意书**	
签署知情同意书日期	|__|__|__|__|年|__|__|月|__|__|日
知情同意书版本号	____________________
签署知情同意书时适用方案版本号	|__|__|.|__|

Final Patient Consent Form: KY2020-29

Version date. 13 February 2020

This is the translated version of the Patient Consent Form

in English.

HYDROXYCHLOROQUINE SULFATE IN THE TREATMENT OF COVID-19 INFECTION -A RANDOMIZED, OPEN MULTICENTER STUDY

INFORMED CONSENT

Dear Sir or Madame:

We invite you to participate in a randomized, controlled, open multicenter study of hydroxychloroquine sulfate in the treatment of COVID-19 infection clinical research. Before you agree to participate in this study, please read the following message carefully. You may also discuss it with your relatives and ask your doctor or researcher any questions you wish to know until you get a satisfied answer. If you agree to participate in this study, please sign the informed consent and keep a copy of the informed consent signed by both parties.

I. RESEARCH BACKGROUND AND PURPOSE:

Since December 2019, a number of cases of COVID-19 pneumonia have been found in wuhan, Hubei province. With the spread of the epidemic, such cases have also been found in other parts of China and abroad. So far, there is still a lack of effective and specific anti-coronavirus drugs in the treatment of COVID-19 pneumonia. Using drugs with potential antiviral effects and already been clinically proven safety in humans is one of the fastest solutions. Hydroxychloroquine (HCQ) sulfate is an antimalarial and immunoregulatory drug that has been clinically used for decades. It was first used in the treatment of systemic lupus erythematosus (SLE) in 1955 and rheumatoid arthritis in 1988. Hydroxychloroquine sulfate has multiple mechanisms of action such as immune regulation, anti-inflammatory, inhibition of cell proliferation and mutagenicity, reduction of blood lipid, anti-thrombotic, and reduction of diabetes risk, etc. The indications of hydroxychloroquine sulfate in clinical practice extends from rheumatic immune diseases to dermatology, oncology and

other fields. Recently, clinical studies have confirmed hydroxychloroquine sulfate possible antiviral effect of high doses in various respiratory viruses (including coronavirus SARS and Middle East respiratory virus). The drug safety of hydroxychloroquine sulfate has been widely recognized, and the drug's tissue distribution concentration is also high.

Therefore, we conducted a national multi-center clinical study to investigate the effect of hydroxychloroquine sulfate on the viral negative conversion rate, clinical symptom remission, improvement of laboratory examination result, and prevention of increased progression. It is expected that hydroxychloroquine sulfate can be confirmed as effective drug in treating patients with COVID-19 infection.

II. PROCEDURES

This study was carried out in 20 centers across the country. After you participate in this study, we will follow up and observe your condition for 28 days, including 14 days or 21days according to the disease severity after treatment and 7 days after treatment. You will receive standard care according to Chinese guideline of COVID-19 pneumonia edition 6 or standard care and hydroxychloroquine sulfate orally. Routine consultation and physical examination, chest HRCT, vital signs monitoring, relevant blood examination and viral nucleic acid sampling will be done according to the protocol.

III. WHAT DO YOU NEED TO DO TO PARTICIPATE IN THIS STUDY

To participate in this study, the doctor will treat your disease according to the current COVID - 19 pneumonia Chinese guideline. Give fully active treatment, follow-up, and randomly with or without use of hydroxychloroquine sulfate orally (for 14-21 days according to the disease severity).

Investigators will collect your demographic information, evaluate your clinical condition before the treatment, 4 days, 7 days, 10 days, 14 days and 21 days (for severe pneumonia) during the

treatment. Laboratory examination and safety of the treatment were followed up along with the treatment and 7 days after treatment.

IV. POSSIBLE BENEFITS OF PARTICIPATING IN THIS STUDY

Your participation in this study may contribute to your recovery from COVID 19 infection.

V. POSSIBLE ADVERSE REACTIONS, RISKS AND RISK PREVENTION MEASURES IN THE STUDY

You will not be at additional risk if you are enrolled in a routine medical program where all examinations are for diseases.

VI. DESCRIPTION OF EXPENSES

The required examination and treatment in this study are completed according to the routine of diagnosis and treatment, and there will be no additional cost for you to participate in this study.

VII. COMPENSATION FOR PARTICIPATION IN THE STUDY, INCLUDING COMPENSATION FOR INJURY

This study is related to diseases of public health emergencies. There is no corresponding financial compensation. At the same time, the treatment you received after participating in this study is carried out in accordance with the standard management of diseases. The additional hydroxychloroquine sulfate tablets you received are commonly used as a routine clinical medication, but individual patients may still have individual adverse drug reactions. However, since the drug has been on the market for many years, investigators are familiar with the type and severity of adverse reactions, and will take corresponding treatment according to the condition if it happens. Corresponding measures will include stopping the use of the drug according to the severity of the adverse reactions. Your participation will make an important contribution to the treatment of covid-

19 infection and provide help for the future treatment of similar patients. We appreciate your participation very much.

VIII. ALTERNATIVE PLAN

This is a clinical observation study, if you are not willing to participate in this study, according to the routine management of the disease, will not affect your clinical treatment.

IX. CONFIDENTIALITY OF YOUR PERSONAL INFORMATION

Your medical records (including research medical records and physical and laboratory examination reports) will be kept in the hospital as required. In addition to investigators, ethics committees, supervision, auditing, pharmaceutical administration departments and other relevant personnel will be allowed to access your medical records, other personnel not related to the study will not have access to your medical records without permission. Public reports of the results of this study will not disclose your personal identity. We will do everything we can to protect the privacy of your personal medical information.

X. TERMINATION OF STUDY

Participation in the study is entirely voluntary. You may refuse to participate in the study or withdraw from the study without reason at any time during the study. This will not affect your relationship with your doctor, nor will it affect the loss of your medical or other benefits. In addition, your participation in this study may be terminated for the following reasons:

1. You did not follow the study protocol.

2. You fail to conduct follow-up observation on time.

XI. ETHICS COMMITTEE

This study was reported to the human research ethics committee of Ruijin hospital affiliated to the school of medicine of Shanghai Jiao tong university, and was approved after a comprehensive review by the committee, including risk assessment of the subjects.

I confirm that I have read and understood the informed consent of this study, voluntarily accepted the treatment in this study, and agreed to use my medical data for the publication of this study.

Subject signature: contact: date:

Proxy signature (if necessary): relationship with subject: date:

Witness (if required): relationship with subject: date:

I confirm that I have explained to the patient the details of the study, including its rights and possible benefits and risks, and given him a copy of the signed informed consent.

Signature of investigator:

Contact information: (mobile phone) date:

Final Patient Consent Form: KY2020-29

Version date. 13 February 2020

This is the original version of the Patient Consent Form

in Chinese.

上海交通大学医学院附属瑞金医院人体研究知情同意书

硫酸羟氯喹治疗新型冠状病毒肺炎的一项随机对照、开放的多中心研究

知情同意书

尊敬的____________患者：您好！

我们将邀请您参加一项“硫酸羟氯喹治疗新型冠状病毒肺炎的一项随机对照、开放的多中心研究”的临床研究。在您同意参加这项研究之前，请仔细阅读以下内容，您也可以和您的亲属一起讨论，并向您的医生或研究人员提出任何您希望了解的问题，直至得到满意的答复。如果您同意参加本研究，请您签署知情同意书，并且保存一份双方签字的知情同意书的复印件。

一、　研究背景和研究目的

2019 年 12 月以来，湖北省武汉市陆续发现了多例新型冠状病毒感染的肺炎患者，随着疫情的蔓延，我国其他地区及境外也相继发现了此类病例。有效控制病毒复制可以改善疾病进展和临床转归，但迄今为止，目前对 2019-nCoV 感染的肺炎的诊治方案中仍缺乏有效特异的抗冠状病毒药物[3]。尽早使用有潜在抗病毒效应且临床已经证明其在人体使用中有良好安全性的药物是解决这一问题的最快方案之一。硫酸羟氯喹（hydroxychloroquine，HCQ）是目前已经在临床应用数十年的抗疟疾和免疫调节药物，1955 年开始用于治疗系统性红斑狼疮（ SLE ），1988 年开始用于治疗类风湿关节炎，其调节免疫的作用及安全性和有效性已经得到了临床的广泛认可。硫酸羟氯喹具有免疫调节、抗炎、抑制细胞增生和诱变、降低血脂以及抗血栓、降低糖尿病风险等多重作用机制，在临床上羟氯喹应用范围从风湿免疫疾病延伸到皮肤、肿瘤等领域。同时，在抗病毒方面，硫酸羟氯喹也有明显的治疗作用，有临床研究证实对各种呼吸道病毒（包括冠状病毒 SARS 和中东呼吸道病毒）高剂量使用可能存在的抗病毒疗效。其药物的安全性和组织中的分布浓度也有很好的表现。

因此，我们开展了一个全国多中心的临床研究，拟对该药物在轻型/普通型新型冠状病毒肺炎患者的病毒转阴率、临床症状缓解、实验室检查指标的改善、预防疾病严重度增加的作用，预期可证实该药物可作为新型冠状病毒肺炎的轻中度患者抗病毒治疗用药。

二、具体程序和流程

本次研究在全国多家单位开展，您参加本项目后，我们会对您的病情跟踪观察 21 天其中治疗 14 天，停药后观察 7 天。期间，按照疾病诊治的流程，接受常规的问诊和体格检查、胸部 HRCT、生命体征监测、相关血液检查、病毒核酸采样检查。

研究过程中治疗原则参照国家卫健委发布的第五版新型冠状病毒感染的肺炎诊疗方案进行。

三、如果参加研究您需要做什么

参与本项目医生会对您的病情按照目前新型冠状病毒肺炎的诊疗方案给予积极治疗、随访，并随机加用或不加用硫酸羟氯喹片的口服药物治疗（疗程 14 天），治疗开始前、第 4 天、第 7 天、第 10 天和第 14 天会进行相应的临床症状的评估和血液学疗效的评估检查。研究第 14 天会进行胸部 CT 的复查，治疗结束后 7 天再次随访治疗的疗效和安全性。

四、参加本研究可能给您带来的受益

您参加本次研究可能有助于您本次新型冠状病毒肺炎的好转康复。

五、参加本研究可能发生的不良反应、风险以及风险防范措施

您参加本研究所需检查均为疾病的常规诊疗项目，因此不会额外增加您的风险。

六、费用情况说明

本研究的所需检查、治疗都是根据诊疗常规完成，不会因参加本项目而额外增加您的费用。

七、参与研究的补偿，包括损伤的赔偿

本项目是一项突发公共卫生事件相关的疾病研究，没有相应的经济补偿。同时，您参与本项目后所接受诊疗按照疾病常规管理进行，所接受的额外硫酸羟氯喹片治疗，作为临床常用药物，仍存在个别患者可能出现个体化的药物不良反应。但因该药物上市多年，不良反应的类型及严重

程度研究者均熟悉，将会根据病情采取相应的治疗措施，并根据不良反应的严重程度采取包括停止使用该药物使用在内的相应措施，不会因参与本项目而造成您的损伤。您的参与将为新型冠状病毒肺炎的诊治做出重要的贡献，为同类患者未来的诊断和治疗提供帮助，再次我们向您表示感谢。

八、替代方案

这是一项临床观察研究，若您不愿意参加本研究，就按照疾病的常规管理，不会影响您的临床治疗。

九、您个人信息的保密

您的医疗记录（包括研究病历及理化检查报告等）将按规定保存在医院。除研究者、伦理委员会、监查、稽查、药政管理部门等相关人员将被允许查阅您的医疗记录外，其他与研究无关的人员在未得到允许的情况下，无权查阅您的医疗记录。本研究结果的公开报告将不会披露您的个人身份。我们将在允许的范围内，尽一切努力保护您个人医疗资料的隐私。

十、终止参加研究

是否参加本项研究完全取决于您的自愿。您可以拒绝参加此项研究，或在研究过程中的任何时间无理由退出研究，这都不会影响您和医生的关系，都不会影响对您的医疗或有其他方面利益的损失。考虑到本疾病属于国家关注的突发性公共卫生事件，如果您退出研究后，研究者将继续收集您的诊疗信息，以便科研人员及临床医生日后更好的了解病毒疫情爆发时紧急情况下的治疗方案，使整个社会以及国家受益。

此外，由于以下原因，可能会终止您参与本研究：

1、您未遵从研究医生的医嘱。

2、您未按时进行随访观察。

十一、伦理委员会

本研究已向上海交通大学医学院附属瑞金医院人体研究伦理委员会报告，经委员会的全面审查和包括对受试者的风险评估，并获得了批准。

我确认已阅读并理解了本研究的知情同意书，自愿接受本研究中的治疗方法，并同意将我的医疗数据用于本研究的发表。

受试者签名：__________　　联系方式：______________　　日期：______________

代理人签名：______________与受试者关系______________联系方式_____________ 日期__

（如果需要）

见证人（如果需要）：______________联系方式：______________ 日期：______________

我确认已向患者解释了本研究的详细情况，包括其权利以及可能的受益和风险，并给其一份签署过的知情同意书副本。

研究者签名：　_____________

联系方式：　______________（手机）　　　　　日期：________________
