# Supplementary material for: Hydroxychloroquine in patients with mainly mild to moderate coronavirus disease 2019: open label, randomised controlled trial
Source: BMJ. 2020 May 14;369:m1849. doi: 10.1136/bmj.m1849 (PMC7221473; doi:10.1136/bmj.m1849)
Supplement: Supplementary file 2 — Web appendix: Supplementary tables [file weit056844.ww2.pdf]

Supplementary Table A. Probability of Negative conversion by the specific time*

Outcome	SOC plus HCQ (N=75)	SOC (N=75)	Probability difference
Day 4, % (95%CI)	14.5 (8.1 to 25.2)	26.3 (17.7 to 38.1)	-11.9 (-25.1 to 13.8)
Day 7, % (95%CI)	43.8 (33.0 to 56.4)	57.3 (46.0 to 69.1)	-13.5 (-30.8 to 3.9)
Day 10, % (95%CI)	65.1 (53.4 to 76.5)	72.0 (61.1 to 82.0)	-6.9 (-23.3 to 9.5)
Day 14, % (95%CI)	79.8 (68.7 to 88.9)	81.3 (71.2 to 89.6)	-1.6 (-15.9 to 12.8)
Day 21, % (95%CI)	85.4 (73.8 to 93.8)	81.3 (71.2 to 89.6)	4.1 (-10.3 to 18.5)

* Negative conversion probability and its 95% CI was estimated by Kaplan-Meire method. 95%CI of probability difference was by an approximate normal distribution and the standard error was estimated by the bootstrap method (N=1000). SOC=standard-of-care, HCQ= hydroxychloroquine.

Supplementary Table B. Updated results on probability of negative conversion by the specific time*

Outcome	SOC plus HCQ (N=75)	SOC (N=75)
Day 4, % (95%CI)	14.4 (8.0 to 25.1)	27.7 (18.9 to 39.6)
Day 7, % (95%CI)	46.4 (35.5 to 58.9)	61.0 (49.8 to 72.3)
Day 10, % (95%CI)	67.9 (56.6 to 78.7)	72.5 (61.8 to 82.4)
Day 14, % (95%CI)	80.9 (70.5 to 89.4)	81.7 (71.7 to 89.8)
Day 21, % (95%CI)	86.9 (76.9 to 94.0)	81.7 (71.7 to 89.8)
Day 28, % (95%CI)	NE (NE to NE)	100 (100 to 100)

* The probabilities of negative conversion between the two groups were re-analyzed using follow-up data by April 27, 2020 to show the up-to-date results on our primary outcome since our manuscript submission. Negative conversion probability and its 95% CI were estimated by Kaplan-Meire method.

The probabilities of negative conversion in the HCQ plus SOC group were not evaluable due to no additional event or censoring after day 23 (by day 23, the probability was 90.2, 95%CI (79.7, 96.6)). In the SOC group, all the patients reached negative conversion or censored before day 22 and the negative conversion probability is 100% by day 22 and therefore remained 100 (100 to 100) by day 28.

SOC=standard-of-care, HCQ= hydroxychloroquine. NE=not evaluable
